# Relating movement markers of schizophrenia to self-experience—a mixed-methods study

**DOI:** 10.3389/fpsyt.2023.1212508

**Published:** 2023-06-21

**Authors:** Lily A. L. Martin, David Melchert, Monika Knack, Thomas Fuchs

**Affiliations:** ^1^Department of Psychology, Faculty of Behavioral and Cultural Studies, University of Heidelberg, Heidelberg, Germany; ^2^Department of General Psychiatry, Centre for Psychosocial Medicine, Academic Medical Center, University of Heidelberg, Heidelberg, Germany; ^3^Research Institute for Creative Arts Therapies (RIArT), Department of Therapy Sciences, Alanus University of Arts and Social Sciences, Alfter, Germany; ^4^Department of Educational Science and Psychology, Free University Berlin, Berlin, Germany; ^5^Department of Philosophy, Faculty of Philosophy, University of Heidelberg, Heidelberg, Germany

**Keywords:** self-disorders, movement markers, schizophrenia, examination of anomalous self-experience (EASE), mixed-methods

## Abstract

**Introduction:**

Basic self-disorders on the one hand and motor symptoms on the other hand are discussed as endophenotypes of schizophrenia psychopathology. However, the systematic interaction between motor symptoms and the self-experience of patients is rarely studied.

**Methods:**

In a previous study we defined motor markers of schizophrenia via a data-driven analysis of patients' gait patterns. In this study, we related the movement markers to measures of basic self-disorder obtained with EASE interviews. We substantiated the correlations with a qualitative content analysis of the interviews of a subset of four patients. We related qualitative and quantitative data on an intra- and interpersonal level.

**Results:**

Our results suggest an association between the previously defined, theory-independent movement markers and basic self-disorders, specifically in the domain of cognition, self-experience and bodily experiences. While movement marker manifestation was not precisely reflected in the individuals' descriptions of anomalous self- and body experience, we found clear trends of more and more intense descriptions with increasing movement marker scores, when looking at specific experiences, such as hyper reflexivity.

**Discussion:**

These results foster an integrated view of the patient and could stimulate therapeutic approaches aiming at an improvement of self- and body-experience of patients with schizophrenia.

## 1. Introduction

Schizophrenia is among the most incapacitating psychiatric conditions and manifests in a variety of symptoms which scientists struggle to subsume under one coherent concept (1). Although basic self-disorders (SDs) have been reported as a characteristic feature since the first definition of schizophrenia, they have only been discussed as the pathogenetic core of schizophrenic psychoses within the past two decades (2–9). SDs are observed in the majority of people with schizophrenia and often occur in prodromal stages of the illness, long before positive symptoms arise and schizophrenia is diagnosed (8). Recent reviews found strong evidence for SDs being a phenotypic marker of vulnerability to the illness: SDs not only selectively aggregated within schizophrenia spectrum disorders (SSD) but also occurred in patient groups with clinical high risk and ultra-high risk for psychosis (7, 8). Moreover, SDs gradually increase from non-psychotic disorders over clinical and ultra-high-risk patients to patients with SSD (8), and elevated SD levels in adolescence can predict the diagnosis of SSD later in life (10). A recent meta-analysis concludes that SDs are not just another symptom domain occurring in parallel with other symptom clusters but rather a generative condition of altered experience that is necessary for certain symptoms to arise (delusion, hallucinations, and social withdrawal) [(7), p. 1013].

Basic self-disorders are subtle but specific disruptions of the so-called minimal self—the tacit, pre-reflective feeling of being the owner or creator of one's experiences, thoughts, and actions (11–13). The minimal, basic, or core self describes the integral first-person perspective of our consciousness: “I am the one looking at the tree,” without having to make this certainty explicitly conscious by active introspection (12). Phenomenologists also speak of “ipseity” when describing the unconscious feeling of mineness of all our experiences (13). Moreover, seeing or touching the tree always comprises the background feeling of the body, or the implicit awareness of being an *embodied* subject of action, including interoceptive, proprioceptive, and kinesthetic awareness as well as a basic auto-affection or “feeling of being alive” (11, 13, 14). Neuropsychological research supports the assumption that an intact basic sense of self arises from two components: (a) the sense of body ownership and (b) the sense of body agency. While multisensory afferent signals are considered sufficient to generate a sense of ownership, recent studies suggest that efferent motor signals (self-initiated movement) and afferent sensory feedback (sensorimotor integration) are necessary to generate a sense of agency and an integrated bodily self-experience (15–17). Hence, correctly attributed body and movement experiences are considered essential prerequisites for the emergence of an integrated sense of self (17).

SDs in schizophrenia present as a subtle feeling of alienation, inner emptiness or inanimation, and loss of natural self-evidence (3, 9, 18, 19). The affected no longer feel at home “in their own bodies and worlds (3, 20, 21). The self-evidence with which the body mediates sensory perception, action processes, emotional expression, and social interactions is primarily disturbed or increasingly lost (3, 12, 19). Subtle feelings of depersonalization are accompanied by altered body and movement perception (disautomation of movement sequences, hyper-reflexive self-observation) and pronounced interaction difficulties (reduced emotional expression in gestures and facial expressions, lack of non-verbal resonance) (22). Against this background SDs are often subsumed under the term of *disembodiment* (3, 12, 23). In acute psychotic states, the subtle experiences can increase to an existentially threatening “ego dissolution” (22). People lose the sense of agency for their own emotions or actions, leading to delusions of manipulation or alien control (positive symptoms) (9, 24, 25).

In 2005, a collaboration between Danish, German, and Norwegian psychiatrists and phenomenologists lead to the publication of the Examination of Anomalous Self-Experience (EASE), a semi-structured, qualitative interview, which explores experiential or subjective anomalies of individuals with schizophrenia. The interview was constructed by Parnas et al. on the basis of self-reports of patients, has a strong descriptive, diagnostic and differential-diagnostic value, and is now considered the most established assessment tool for SDs (26). With its 57 items, which are structured into five subdomains: (1) cognition and stream of consciousness, (2) self-awareness and presence, (3) bodily experiences, (4) demarcation/transitivism, and (5) existential reorientation—it incorporates all aspects of a disturbed basic sense of self. Fuchs et al. focus on a disruption of pre-reflective bodily self-awareness and the implicit sensorimotor functions of the body and thus consider a disembodiment and anomalous bodily and self-experiences (EASE domain 2 and 3) as the core disturbance of schizophrenia and SDs (3, 20, 24, 27–30).

Most people with schizophrenia not only present with altered body and movement perception but also with externally observable general motor abnormalities (GMAs), such as neurological soft signs (NSSs), abnormal involuntary movements (AIMs), or psychomotor slowing [see Ref. (31–33) for an overview]. In fact, subtle impairments in motor coordination, sensory integration, balance, and sequencing of complex motor acts (all NSS) are among the most established findings in schizophrenia research (11, 31, 34). Similar to SDs, GMA have been observed in up to 80% of people with SSD and can be found in individuals with a high risk of psychosis as well as in first-degree relatives with a genetic risk for the illness. Hence, they can be considered a sign or an endophenotype for schizophrenia vulnerability (31, 35). Despite their high prevalence, there is no single definition of GMA. Categorizations of associated symptom clusters vary with conceptual frameworks and assessment instruments of the respective studies and researchers (31, 35, 36). In a previous study, we identified theory-independent movement markers (MMs) of people with schizophrenia: *via* data-driven analysis of full-body motion patterns in gait, we extracted medication- and weight-independent limitations in posture, velocity, regularity of gait as well as sway, flexibility, and integration of body parts. Specifically, the adjustment of body sides, limbs, and movement direction were affected [see Ref. (31) for study details].

While it is beyond debate that postures or movement are in a constant bidirectional interplay with cognition and emotion (37–39) and that subtle or intense changes in movement ability and behavior are related to changes in self-experience and subjective wellbeing of patients (40), the systematic interaction between motor symptoms and the self-experience of patients with schizophrenia is rarely studied. Only recently, there have been attempts to systematically relate phenomenological concepts, such as SDs, to underlying neurocognitive mechanisms (41, 42) or GMA (11). Tonna et al. (11) identified postural patterns (motor rigidity or instability) and a general disruption of the gait cycle as a marker for the underlying neurodevelopmental disturbance in early-onset schizophrenia and found associations of the specific motor pattern with subjective bodily experiences (mainly the loss of bodily integrity, cohesion, and demarcation). As a result, the researchers hypothesized motor symptoms and SDs to be “two poles of the same underlying perturbation of the structure of consciousness, […] first and foremost rooted in motor enactment” [11, p. 12].

Taking into account the role of the moving body in the development of a coherent sense of self [sense of body ownership and agency; see above and (15)], in the following study, we aimed at relating the previously identified MM to subtle changes in the self- and body experience of the participants measured by the phenomenological examination of anomalous self-experience (EASE). By adding an in-depth, qualitative analysis of a subset of EASE interviews, we chose a mixed-methods approach, which aims at relating qualitative and quantitative data. We are not aware of any resembling study.

We addressed the following hypotheses:

H1. The previously defined MMs are correlated with SD measurements: Specifically, higher scores of the MM (a more severe impairment) are associated with higher ratings in the EASE subdomains 2 “Self-awareness and presence” and 3 “Bodily Experiences.”

H2. Specific anomalous self-experiences, (specific EASE items, including but not restricted to “diminished sense of self,” “distorted first-person perspective,” “somatic depersonalization”) are correlated with stronger MM manifestations.

H3. Individual manifestations of MM (movement profiles) are reflected in the participants' qualitative descriptions of self- and body experience, in the sense that higher MM manifestations are associated with more and more intense descriptions of limitations in self- and body experience.

## 2. Materials and methods

The study was part of the collaborative research project “Schizophrenia and the Moving Body” (Center for Psychosocial Medicine (CPM), University Hospital Heidelberg; Heidelberg Center for Motion Research (HCMR), University of Heidelberg; BioMotionLab, York University). It was conducted in accordance with the Declaration of Helsinki (2013) and the code of ethics for doctors of the state chamber of physicians of Baden-Württemberg and approved by the local ethics committee of the Medical Faculty of Heidelberg University (S-629/2018).

### 2.1. Recruitment and assessment

Participants with a diagnosis of schizophrenia were recruited between 2019 and 2020 among patients who were admitted to one of four wards of the CPM. All patient files of the wards were screened according to the following selection criteria: Inclusion: (1) able to consent, (2) between 18 and 60 years old, and (3) diagnosed with a schizophrenia spectrum disorder (ICD-10: F20.0 – F20.9) prior to study inclusion by senior psychiatrist unrelated to the study, (4) stable on antipsychotic medication for at least 2 weeks; exclusion: (1) acute psychosis (ICD-10: F23), (2) diagnosis of a catatonic or schizoaffective subtype (ICD-10: F20.2, F25.0-F25.9), (3) history of brain trauma, neurological or internal diseases, heavy fractions, or prostheses, (4) visible tremor, (5) strong visual impairment, (6) alcohol/substance abuse or dependency within the past 12 months or substance-induced psychosis (ICD-10: F19.5), (7) IQ < 70, (8) SAS score above 4, and (9) pronounced language barriers. With the help of flyers and through personal approach by the leading physicians, eligible patients were referred to the study. Since the control group is not actively analyzed in this publication but only functions as a point of reference, recruitment procedure and selection criteria of the control group are described elsewhere (31). All participants, who met the inclusion criteria, were informed in great detail about the course of the study and gave informed consent prior to participation. They were interviewed at the CPM using the EASE (26) and then invited to the HCMR to take part in the movement assessment. EASE interviews were conducted by the first and the last author (LM and TF) with the help of a semi-structured interview guide (43) and audio-taped [Check (21) for details on EASE documentation and analysis]. Both interviewers were trained in the usage of the interview within official workshops conducted by the interview's author group. Details of the movement assessment are given in another publication (31). Depending on the participants' schedules, the assessments took place on the same day or on different days. After completion of the study, participants were reimbursed with an expense allowance of 10 € per participation hour.

### 2.2. Data analysis

MMs of Schizophrenia were defined in a previous study by an algorithm-driven analysis of the MoCap data comparing patients and controls. See Martin et al. (31) for a detailed description of the respective analysis. While walking, patients with schizophrenia displayed (1) a hanging *head posture*, (2) a reduced *gait regularity*, (3) an increased *variation of gait regularity*, (4) a reduced *stride length*, (5) *arm sway*, (6) *elbow sway*, (7) and *knee sway*, (8) an increased *lateral body sway*, (9) a reduced *gait velocity*, (10) *arm sway velocity*, (11) and *elbow sway velocity*, (12) less *adjustment of body sides*, (13) less *goal directedness of movement*, (14) less *flexibility of limb movement*, (15) less *adjustment of limb movement*, (16) and a decreased *adjustment of sway velocities*. Calculations for the publication at hand were done using the free software environment for statistical computing and graphics R (Version 4.0.2) and RStudio (Version 1.2.5019) (44) as well as IBM SPSS (Version 27.0.0.0).

After defining sample characteristics, we performed five steps of analysis.

#### 2.2.1. Interview rating and inter-rater reliability

All interviews were rated according to an adapted rating scale by the first author of this article (45). To ensure a certain level of objectivity, half of the interviews (randomly chosen) were rated by a second rater who was not involved in the study but trained in EASE rating. Rater training took place over the course of 2 days at CPM and was conducted by the last author (TF). The second rating was used to calculate inter-rater reliabilities (IRRs). IRRs were calculated following a tutorial by Hallgren (46). We computed intra-class correlations (ICCs) for the overall EASE score and for all five subdomains, respectively. Except for subdomain four, the ICCs of the individual subdomains were sufficient: (1) cognition and stream of consciousness: ICC_1_ = 0.84, (2) self-awareness and presence: ICC_2_ = 0.85, (3) bodily experiences: ICC_3_ = 0.66, (4) demarcation/transitivism: ICC_4_ = −0.20, and (5) existential reorientation: ICC_5_ = 0.61. The low ICC of subdomain four can be explained by a misunderstanding of the second rater, which led to a complete omission of ratings in subdomain four. Because the overall IRR was considerably high (ICC_Total_ = 0.89), a satisfactory amount of objectivity can be assumed. Therefore, the EASE scores of the first rater were used in all further steps of the analysis.

#### 2.2.2. Correlations with EASE scores

We correlated the defined MM (31) with EASE total scores and with the five subdomains of the interview. Correlations followed Pearson (47) and were two-tailed. Normal distribution of the variables was determined with the Kolmogorov–Smirnov test (48, 49). The EASE subdomains are a collection of experiences, which share a common ground but are very heterogenous in their experiential nature. Hence, to get an idea of the correlations' origin (which exact experience is related to the respective measurable MM?), we also correlated the MM with specific EASE Items. Following Streiner and Norman (50), who advice against overcorrecting with the Bonferroni method in explorative studies, which might find promising leads, we deliberately chose not to apply a correction method for multiple testing. Therefore, we understand the following results as a preliminary base for further hypothesis generation. Furthermore, due to the explorative nature of our study, we understood non-significant but moderate correlations (above 0.3) as pointing toward a correlational trend.

#### 2.2.3. Movement index and movement profiles

Because every quantitative analysis inevitably comprises a data reduction, we decided to enrich the correlational clusters with idiosyncratic qualitative information. To enable an in-depth analysis of the qualitative interview data and relate the textual information to the quantitative MM, we extracted a subsample of four patients. Since a targeted selection of particularly interesting interviews is associated with the risk of a confirmation bias, we determined a selection criterion based on the quantitative movement data: The *movement index* (MI) was computed on the basis of the abovementioned 16 MM. It is defined as the patient's mean of absolute z-deviations across all 16 MM and can be interpreted as the individual's prototypicality of movement markers within the schizophrenia sample. To be exact, we first transferred all individuals' MM values to absolute z-values, indicating the deviation of the respective patient's MM manifestation from the mean values of the other patients. Absolute values were used to prevent positive and negative values from averaging out in the following step. Then, we calculated the individuals' mean across all 16 transferred MM (MI = mean of the absolute MM z-values). Using the MI, we identified three patients who covered the entire spectrum of MM manifestation: (a) the person with the lowest movement index and, hence, the smallest deviation from the other patients, representing the prototypical patient of the schizophrenia sample, (b) one person with an average movement index, representing a medium deviation of the schizophrenia sample, and (c) the person with the highest movement index, representing a high deviation from the other patients or the most atypical movement profile regarding the patient group. Because we used absolute z-values, the abovementioned deviation includes both negative and positive differences of MM manifestation to the schizophrenia sample. However, the selection procedure provided a subsample containing exclusively male patients. To reflect the entire sample's gender distribution within the subsample, one more female patient with an average MI was selected. To get an overview of the manifestation of the 16 individual MM within the subsample individuals and make the quantitative data relatable to the qualitative interview data, we created four *movement profiles* (MPs). For this, we computed and plotted two z-values of each MM for each of the four subsample patients using the mean of and thus relating the movement markers to the (a) schizophrenia sample and (b) the control group. While (a) allowed the assessment of the prototypicality of schizophrenia-characteristic MMs, (b) represented a more disease-oriented dimension regarding the deviation from the control group. Finally, we identified remarkable movement markers of subsample patients, which exhibited z-scores higher or lower than 1 regarding the control group. These remarkable movement markers were related to the individuals' categories of subjective self- and body experiences, hence the qualitative data from the EASE interviews.

#### 2.2.4. Qualitative analysis of interview data

The qualitative analysis of the subsample's interview data followed a consensual research approach (51, 52). It was implemented by an analysis committee (first, second, and fourth author of the publication coming from the fields of psychology and psychiatry) who within regular focus groups aimed at a consensus on the suitability of procedures as well as the analysis of the interview data. For the interpretation of the interview data, we invited an independent expert (third author, phenomenologist) to ensure a certain level of interpretation objectivity. We used the software MAXQDA 2020 (53) for all qualitative analysis steps. After the transcription of the interview audio-files [guidelines of McLellan et al. (54)], we conducted a qualitative content analysis following Kuckartz (55). The transcripts of the interviews can be found in the Supplementary material. All identifying names and places were anonymized. To account for the diverse expressions and descriptions of individual experiences and to make the qualitative analysis as reproducible as possible, we deliberately chose to ignore the factorial and item structure of the original EASE interview and to analyze the interviews “as a whole.” To enable an open and unbiased analysis of the textual material, we chose to search for salubrious and pathological expressions of self- and body experience rather than SDs specifically. We performed five steps of qualitative analysis: (1) deductive development of the main thematic categories and codes of self- and body experience using theory and literature (15, 20, 26, 56–61); (2) coding of the interview material; (3) compilation of all text passages coded within a category; (4) inductive enrichment of the main thematic categories and formation of lacking categories using the interview material; and (5) coding of the complete material with the final category system. Coding was continued until data saturation was reached, hence until found categories recurred and no novel variations were found.

#### 2.2.5. Relating interview data to MMs

As stated above, only remarkable MMs (exhibiting SDs > +/-1 than the control mean) were related to the qualitative data. The integration of qualitative and quantitative data was done on two levels: (a) On an intrapersonal level, we related the number of coding of a respective experiential category as well its subjective severity (taken from the individual descriptions) to the individual's MM manifestations (intensity of manifestation and kind of MM). (b) On an interpersonal level, we searched and compiled trends: Are more and more intense descriptions of aberrations in self- and body experience related to higher manifestations in remarkable MMs?

## 3. Results

### 3.1. Sample characteristics

We interviewed 22 patients with schizophrenia. Due to rigorous inclusion criteria and dropouts, we analyzed the interview data of 20 patients only−14 men and 6 women. See Table 1 for detailed sample characteristics.

**Table 1:** Sample characteristics.

	**Patients with schizophrenia (*N =* 20)**
**Gender**	
Male	14 (70.0%)
Female	6 (30.0%)
**Age (Years)**	
Mean (SD) [Min, Max]	39.0 (11.8) [20.0, 59.0]
**Body Weight (kg)**	
Mean (SD) [Min, Max]	91.4 (16.1) [60.9, 125]
**Height (cm)**	
Mean (SD) [Min, Max]	177 (9.87) [156, 197]
**BMI**	
Mean (SD) [Min, Max]	29.0 (3.36) [23.2, 36.2]
**Handedness**	
right	17 (85.0%)
left	3 (15.0%)
**Nationality**	
German	19 (95.0%)
Other	1 (5.0%)
**Mother tongue**	
German	18 (90.0%)
Other	2 (10.0%)
**Family status**	
single	17 (85.0%)
married	2 (10.0%)
divorced	1 (5.0%)
**Years of education**	
Mean (SD) [Min, Max]	14.7 (4.19) [9.00, 26.0]
Missing	7 (35.0%)
**Job**	
In training	1 (5.0%)
Employed	7 (35.0%)
Self employed	0 (0%)
Retired	2 (10.0%)
Unemployed	7 (35.0%)
On sick leave	2 (10.0%)
Other	1 (5.0%)
**Olanzapine equivalents**	
Mean (SD) [Min, Max]	17.2 (8.30) [5.00, 32.3]
**Years of illness**	
Mean (SD) [Min, Max]	12.6 (11.5) [0.0800, 38.5]
**Number of psychotic episodes**	
Mean (SD) [Min, Max]	5.05 (4.86) [1.00, 20.0]

### 3.2. Correlations of MM with self-experience

Tables 2, 3 display the correlations of the MM with the patients' SD measurements by the EASE. Table 2 gives an overview of correlations with EASE total and subscores, and Table 3 displays correlations with certain single EASE items.

**Table 2:** Correlations of movement markers with the patients' self- and body experience: EASE subscores.

		**Movement markers**							
	**EASE Subscores**	**Head posture**	**Gait regularity**	**Variation of gait regularity**	**Stride length**	**Arm sway**	**Elbow sway**	**Knee sway**	**Lateral body sway**
**Examination of Self-Experience (EASE)**	**Total**	−0.319	0.080	0.295	−0.263	−0.247	−0.099	−0.189	0.331
	**1. Cognition**	−0.336	0.182	0.442	−0.376	−0.224	−0.205	−0.272	0.324
	**2. Self-Awareness**	−0.253	0.058	0.109	−0.069	−0.153	0.056	−0.019	**0.462** ^*^
	**3. Bodily Experiences**	−0.205	−0.022	0.057	−0.327	−0.256	−0.185	−0.212	0.013
	**4. Demarcation**	−0.271	0.155	−0.050	−0.094	−0.003	0.061	−0.214	−0.132
	**5. Existential Reorientation**	0.001	−0.181	0.413	−0.030	−0.230	−0.069	0.011	0.151
	**EASE Subscores**	**Gait velocity**	**Arm sway velocity**	**Elbow sway velocity**	**Adjustment of body sides**	**Goal directedness of movement**	**Flexibility of limb movement**	**Adjustment** **of limb movement**	**Adjustment of sway velocities**
	**Total**	−0.379	−0.085	0.010	0.199	−0.169	−0.369	−0.015	−0.174
	**1. Cognition**	−0.436	−0.001	0.010	0.083	−0.184	−0.121	−0.198	−0.205
	**2. Self-Awareness**	−0.304	−0.038	0.066	0.338	−0.286	−0.416	0.080	−0.167
	**3. Bodily Experiences**	−0.223	−0.070	0.001	0.417	−0.223	−0.388	0.303	0.039
	**4. Demarcation**	−0.044	−0.101	−0.060	−0.112	0.100	−0.133	−0.065	−0.058
	**5. Existential Reorientation**	−0.175	−0.209	−0.069	−0.242	0.285	−0.263	−0.147	−0.145

Higher EASE values signify increased disturbances in self- and body experience. Correlations follow Pearson and are two-tailed. Statistically significant correlations are marked bold. ^*^p < 0.05, ^**^p < 0.01.

**Table 3:** Correlations of movement markers with the patients' self- and body experience: EASE single items.

		**Movement markers**							
	**EASE Items**	**Head posture**	**Gait regularity**	**Variation of gait regularity**	**Stride length**	**Arm sway**	**Elbow sway**	**Knee sway**	**Lateral body sway**
**Examination of self-experience (EASE)**	**2.1: Dim. sense of basic self**	**−0.337**	**−0.081**	**0.15**	**0.29**	**−0.125**	**−0.153**	**0.39**	**−0.216**
	**2.2: Dist. first-person perspective**	**−0.092**	**−0.411**	**0.24**	**−0.275**	−0.492^*^	**−0.330**	**−0.144**	**0.25**
	**2.3: Depersonalization**	**−0.090**	**−0.095**	**−0.055**	**0.1**	**−0.150**	**0.04**	**0.11**	**−0.026**
	**2.4: Diminished presence**	**−0.112**	**−0.228**	**−0.304**	**−0.002**	**−0.093**	**−0.004**	**−0.046**	**0.07**
	**2.6: Hyperreflexivity**	**−0.022**	**−0.072**	**−0.260**	**−0.182**	**−0.003**	**0.05**	**−0.121**	**0.35**
	**3.1: Morphological change**	**−0.044**	**−0.056**	**−0.253**	**−0.047**	**−0.135**	**−0.129**	**−0.221**	**−0.196**
	**3.3: Somatic Depersonalization**	**−0.117**	**−0.121**	**0.11**	**−0.294**	**−0.224**	**−0.228**	**0.02**	**−0.150**
	**3.4: Psychophysical misfit**	**0.15**	**−0.275**	**−0.011**	**−0.136**	**−0.440**	**−0.478**	**−0.086**	**−0.097**
	**3.5: Bodily disintegration**	**−0.357**	**0.18**	**0.02**	**−0.291**	**−0.037**	**−0.011**	**0.01**	**0.11**
	**3.6: Spat. of bodily experiences**	**0.2**	**0.2**	**0.1**	**−0.057**	**0.1**	**0.2**	**0.23**	**0.41**
	**3.7: Cenesthetic experiences**	**−0.141**	**0.16**	**0.08**	**−0.350**	**0.04**	**0.17**	**−0.262**	**0.24**
	**3.8: Motor disturbances**	**−0.260**	**0.31**	**0.17**	**−0.381**	**0**	**−0.003**	**−0.377**	**0.11**
	**EASE Items**	**Gait Velocity**	**Arm sway velocity**	**Elbow sway velocity**	**Adjustment of body sides**	**Goal directedness of movement**	**Flexibility of limb movement**	**Adjustment of limb movement**	**Adjustment of sway velocities**
	**2.1: Dim. Sense of basic self**	**0.21**	**−0.035**	**−0.043**	**0.19**	**−0.096**	**−0.086**	**0.22**	**0.13**
	**2.2: Dist. First-person perspective**	**−0.402**	**−0.336**	**−0.249**	0.450^*^	**−0.268**	−0.619^**^	**0.34**	**0.21**
	**2.3: Depersonalization**	**0.08**	**−0.061**	**0.06**	**0.34**	**−0.123**	**−0.375**	**0.27**	**0.09**
	**2.4: Diminished presence**	**−0.023**	**−0.144**	**−0.089**	**0.08**	**0.02**	**−0.224**	**0.15**	**0.07**
	**2.6: Hyperreflexivity**	**−0.145**	**0.12**	**0.15**	**0.32**	**−0.438**	**−0.174**	**0.16**	**−0.119**
	**3.1: Morphological change**	**0.11**	**−0.047**	**−0.047**	**0.25**	**−0.033**	**−0.164**	**0.32**	**0.09**
	**3.3: Somatic depersonalization**	**−0.199**	**−0.016**	**−0.017**	0.580^**^	**−0.363**	**−0.315**	**0.44**	**0.24**
	**3.4: Psychophysical misfit**	**−0.067**	**−0.272**	**−0.307**	**0.46**	**−0.057**	**−0.428**	0.557^*^	**0.41**
	**3.5: Bodily disintegration**	**−0.170**	**0.23**	**0.25**	**0.25**	−0.516^*^	**−0.113**	**−0.008**	**−0.093**
	**3.6: Spat. Of Bodily Experiences**	**−0.240**	**0.24**	**0.23**	**0.52**	**−0.465**	**−0.043**	**−0.171**	**−0.062**
	**3.7: Cenesthetic experiences**	**−0.354**	**0.08**	**0.19**	**−0.094**	**−0.138**	**−0.126**	**−0.224**	**−0.382**
	**3.8: Motor disturbances**	−0.419	0.02	0.03	0.02	0.06	−0.016	−0.147	−0.265

Higher EASE values signify increased disturbances in self- and body experience. Correlations follow Pearson and are two-tailed. Statistically significant correlations are marked bold. Shortages: 2.1 Dim. Diminished, 2.2 Dist. = Distorted, 3.6 Spat. = Spatialization. ^*^p < 0.05, ^**^p < 0.01.

#### 3.2.1. Correlations of MM with EASE total and subscores (H1)

Except for LBS, we could not find statistically meaningful correlations of MM with overall SDs or any of the EASE subscales. An increased lateral body sway (LBS) was associated with higher ratings in cognition or self-awareness disturbances. The association of a higher LBS with changes in self-awareness (EASE subscore 2) is statistically significant on an alpha-level of 0.05.

However, we found many correlational trends: While some MM moderately correlated with subscale 3 (stride length, adjustment of body sides, flexibility of limb movement, and adjustment of limb movement), many also displayed a moderate correlational trend with deficits in *self-awareness and presence* (gait velocity, lateral body sway, adjustment of body sides, and flexibility of limb movement). Furthermore, many MMs were associated with disturbances in *cognition and consciousness* (EASE subscale 1: gait velocity, stride length, variation of gait regularity, and lateral body sway). This seems logical, because, as stated above, not only SDs and movement but also movement and cognition are in a constant bidirectional interplay with each other.

We expected the MM to mainly be correlated with the amount of *anomalous bodily experiences (EASE subscale 3) and disturbances in self-awareness and presence (EASE subscale 2)*. Taken together, despite the lack of statistically significant correlations, most kinematic MM displayed a correlational trend with either the SD total score, or one or many of the first three subscores of the EASE. For instance, the gait velocity was inversely correlated with the amount of SDs in general (*r* = −0.379), with disturbances in *cognition and consciousness* (*r*= −0.436) as well as with disturbances in *self-awareness* in particular (*r* = −0.304). A higher manifestations of the respective movement marker—a slower walk—were related to increased SDs regarding cognition, self-awareness, and body perception. We could not find moderate correlations of SDs with the sway of specific limbs (arm, elbow, and knee sway). These MM might be too specific to display a correlation with overall experiences of self-disturbance in such a small sample size. The fact that we could find moderate correlations with the overall gait velocity but not with specific limb velocities supports this notion.

Looking at the relational MM, we can find a clear correlational trend with changes in body perception: Most relational MMs are moderately correlated with the amount of anomalous bodily experiences (adjustment of body sides, flexibility of limb movement, and adjustment of limb movement). Many of them are also moderately correlated with disturbances in self-awareness and presence. The adjustment of left and right arm movement (adjustment of body sides), for example, is not only positively correlated with EASE subscore 3 (*anomalous bodily experiences: r* = 0.417.*)* but also with subscore 2 (amount of disturbances in *self-awareness and presence*: *r*_RatioWSLR1, EASE:Self − Awareness_ = 0.338). Both associations make sense since a higher value in the respective MM stands for less of an adjustment between the body sides. Hence, increased disadjustment was related to increased SDs considering self-awareness and body perception. Other relational MMs, such as the flexibility or the adjustment of limb movement, show the same correlation properties. Considering the small sample size of 20 patients, the abovementioned correlations are comparably high. All correlational patterns but the one of the postural marker (head posture) are in line with our hypothesis. Correlations of head posture with EASE total and subscore 1 are moderate and point toward a correlational trend but contradict hypothesis 1 in the sense that, e.g., increased disturbances of cognition are related to a more upright posture.

#### 3.2.2. Correlations of MM with specific EASE items (H2)

We expected specific anomalous experiences to be associated with higher MM manifestations. We found six statistically significant associations: (1) a reduced arm sway was related to feelings of a distorted first-person perspective (significant inverse correlation: *r* = −0.429^*^), (2/3) lacking adjustment of body sides while walking was correlated with disturbances in the first-person perspective (*r* = 0.450^*^) as well as with experiences of somatic depersonalization (*r* = 0.580^**^; significant positive correlations; higher values in the adjustment ratio stand for less of an adjustment), (4) a reduced goal directedness of the gait was related to experiences of bodily disintegration (significant inverse correlation: *r* = −0.516^*^), a (5) reduced flexibility of limb movement was related to disturbances in the first-person perspective (significant inverse correlation: *r* = −0.619^**^), (6) and a reduced adjustment of limb movement during gait was related to descriptions of psychophysical misfit (significant positive correlation: *r* = −0.557^*^, see explanation of adjustment variable above).

In addition to the statistically significant correlations, we found many correlational trends: In general, almost all MMs were associated with one or many specific anomalous self- or bodily experiences. Mostly, people who displayed increased MM also reported at least one anomalous bodily experience, such as somatic depersonalization, psychophysical misfit, bodily disintegration, coenesthetic experiences, or general motor disturbances.

Furthermore, half of the MM were at least moderately correlated with the experience of a distorted first-person perspective. Other anomalous experiences, such as feelings of general depersonalization, were moderately associated with a reduced adjustment of body sides and less flexibility in the limb movement. In addition, increased experiences of hyperreflexivity were moderately associated with many relational MM (lateral body sway, adjustment of body sides, and goal directness of movement). Overall, the adjustment-related MM (adjustment of body sides, goal directedness of movement, flexibility of limb movement, and adjustment of limb movement) displayed the most correlational trends with specific self- and body-related anomalous experiences. Again, the correlations of specific anomalous experiences with head posture contradicted our hypotheses. Increased anomalous experiences were associated with more upright walking.

### 3.3. Self- and body experience of subgroup patients

Table 4 gives an overview of the subgroup's demographic data.

**Table 4:** Movement index, demographic, and medical data of the subsample.

	**Patient**			
	**RM05**	**IH12**	**AB04**	**SF03a**
Movement index	Low	Middle	Middle	High
Gender	Male	Female	Male	Male
Age (years)	30	46	25	24
Mass (kg)	98,6	90,3	105	93,4
Height (cm)	178	175,6	189	178,5
BMI	31,12	29,28	29,39	29,31
Handedness	Left	Right	Right	Right
Olanzapine equivalents	30,64	6,53	13,5	5,32
Years of illness	2,25	26,5	6,5	9,5
Number of psychoses	2	3	6	5

The final categories of the coding system and detailed case vignettes of the individual interviews can be found in the Supplementary material. Due to space limitations, we focus on reporting pathological experiences and give textual examples for those experiential categories, which were mentioned by all patients. Table 5 explains and summarizes the experiential categories mentioned by the four subgroup patients. Patient quotes were translated from German by the first and second author.

**Table 5:** Descriptions and frequencies of the pathological categories mentioned by the subsample.

	**RM05**	**IH12**	**AB04**	**SF03a**		
**Category**	**Frequency of category mentioning**				**Total**	**Brief description of category**
**Self-experience**						
**Feeling of self-insecurity**	**4**	**4**	**12**	**9**	**29**	Worrying about being yourself and being a person.
Feeling of anxiety	3	9	4	0	16	Includes panic attacks, psychic-mental anxiety, phobic anxiety, social anxiety, diffuse anxiety, and paranoid anxiety.
Distorted first-person perspective	0	1	1	0	2	Thoughts, perceptions, or feelings appear as deprived of the tag of mineness.
**Feeling of a loss of control**	**10**	**7**	**9**	**3**	**29**	The feeling of being dependent or to lose control.
Feeling of being absent/not present	1	0	2	2	5	The feeling of not fully participating in the world or not being entirely present in the world.
**Feeling of being unlively/devitalized**	**3**	**1**	**3**	**2**	**9**	A diminished vitality, diminished initiative, hypohedonia or (in extreme cases) the feeling of being a liveless, dead object/body.
**Hyperreflexivity**	**2**	**3**	**3**	**5**	**13**	The tendency to exaggeratedly monitor one's own sensations, emotions and thoughts; The attempt to volitionally steer otherwise tacit processes.
Mirror-related phenomena	0	0	0	1	1	An unusually frequent, and intense looking in the mirror or avoiding one's specular image or looking only occasionally but perceiving a facial or bodily change.
Sense of change in relation to age	0	0	1	0	1	A fundamental feeling of being considerably older or younger than the actual age, not related to social relations/interactions.
Fragmentation of experience	0	1	1	0	2	Stimuli and experiences, which usually tacitly appear to the subject as a unity, are perceived as fragmented.
**Body experience**						
Somatic depersonalization/bodily estrangement	0	0	0	1	1	Estrangement of the body and its movement.
Loss of body agency	0	0	3	0	3	The sense of losing control of one's own body movement.
Motor blocking	0	2	0	0	2	Sudden weakness (paresis), impediment or complete blockage of intended motor actions.
**Disautomation**	**1**	**1**	**2**	**1**	**4**	The tendency to exaggeratedly monitor one's own actions. Patients try to volitionally steer the otherwise tacit actions.
Rigidness/choppiness	0	0	0	1	1	Perception of own movements as rigid and choppy.
Feeling of lost body ownership	0	0	0	1	1	Disturbances in the sense of being the subject /owner of the movement or the body.
Psychophysical misfit and psychophysical split	0	0	0	2	2	The body or individual parts of the body are experienced as being isolated and separated from one another, as not being present at all or as not fitting to the owner.
Coenesthesia	0	1	1	0	2	Unusual bodily feelings, which cannot be explained medically; Also termed body hallucinations.
Motor slowdown	1	0	0	0	1	Own movement is perceived as being slowed down.
Thoughts linked to movements	0	0	1	0	1	Single or repetitive movements are perceived as related to thoughts.
	25	30	43	28		

Note: Categories of self- and body experience are marked bold, if they were mentioned by all of the four patients. Frequencies refer to the entire interview.

All patients describe various examples of existential *feelings of self-insecurity*, which surface in social or work situations or are expressed as the feeling of being fundamentally different to other people. *RM05* even suspects that his self-doubts marked the beginning of his illness: “*I talked about this a lot with my mother. She said, it already started in elementary school. There, a teacher wrote about me: ‘He is able to do a lot but does not dare to do it'. This describes me very well; Until today, I somehow always think, what I do is not good enough.”* His self-questioning leads to a feeling of otherness and loneliness: “*I feel clearly different from other people*.” In his first job, confronted with stressful situations and work pressure, the insecurity escalated into a persistent conviction that people in his work environment would speak badly of him and that he would therefore never find a job again. Similarly, IH12 experiences a multitude of problems in social and work-related situations that have a common basis of self-insecurity: “*I think I did something wrong at work. And then I keep thinking about it. […] Still, there is something I do wrong every day. […] At least one thing.”* Due to the feeling of not being right or doing things the right way, she too often reassures herself by asking for advice about the smallest things (e.g., which company stamp to use). The feelings interfere with daily life to such a degree that she feels compelled to devise a coping routine: “*That's why I always go to church on Sundays, simply because I keep making mistakes, and just to let go of that and know that I'm going to start all over again.”* AB04 presents with the most and strongest descriptions of self-insecurity. In the course of the conversation, they surface as deep uncertainties about what is right or wrong, whether he is right or wrong as a person and which parts of him, including thoughts and perceptions, are healthy or sick: “*My thinking and my behavior are very different from people who are not so sick. […] I can't at all say whether I feel [the thoughts] normally, or whether it is already, uh, abnormal, because it was so long ago that I wasn't sick that I forgot how it should be. And now I don't really know whether that's right, or isn't that right?”*. This existential uncertainty is associated with (a) the alienation of the own self, its thoughts, and actions and leads to a (b) profound feeling of worthlessness and even the questioning of the own right to exist: (a) “*That sounds really weird. My thoughts are clearly mine because I think them. But I don't have the feeling that they are just mine. […] Something is added to the normal structure of thoughts.”; (b)* “*For years I thought I had to die. […] You just have the feeling that you are worth nothing if you are supposed to be killed.”*

Furthermore, all patients experience a loss of control. Loss of control manifests as cognitive phenomena such as pressured thinking, decision ambivalence, or concentration problems and sometimes is associated with body-related phenomena or even has an influence on social interaction. SF03a summarizes his experience from child- and adulthood as follows: “*As a child I said to my mother: The thoughts don't stop. I can't stop thinking. […] It is such a rush of thoughts, such a storm. […] I can no longer filter properly. I can no longer classify the thoughts, allocate the right meaning to them. […] When the thoughts flow in on you, then I would say the filter is broken. There is a processor on the computer, a task scheduler, which assigns to the various processes what is important, what will be carried out first. That one is broken in the brain.”* AB04 compulsively and unintentionally broods over the course of the day: “*I don't go to bed and think: now you're thinking about today again. Not like that, it comes automatically.”* The pressure he experiences through his thoughts is evident as an electrical feeling in his head: “*I have no rest with my thoughts. Many people, I think, can sometimes relax, just think about nothing for a moment or only think about simple things […] And with me it's always [makes loud electric noises with his tongue].”* The feeling is so intense and regular that he initially thought every patient with schizophrenia experiences this sort of pressure. After reporting his sensation to another patient, he was surprised that the other did not experience the phenomenon at all. Naturally moments of loss of control impact daily life. RM05 describes how he sometimes loses track of conversations due to disappearing, fading and suddenly dissipating thoughts or even finds himself somewhere on the street without remembering how he got there: “*[During conversations] I hop from topic to topic. In one moment I think about my e-mail account, in the next I wonder if I closed the window, then a completely different topic comes up. My thoughts are erratic. […] Sometimes the other person looks at me and asks: äh, how did you get there now?”*

Another feeling that is prevalent in all subgroup patients is the *feeling of being unlively or devitalized*. It often is accompanied by a *feeling of being absent or not present* and is expressed in descriptions of being lost in thought, of a gray and unlively environment, strong anhedonia, and an intense lack of initiative or energy. RM05, for instance, sinks into thoughts that come automatically and feel very lively. He then appears absent-minded and subsequently has to be actively brought back into the conversation by other people who snap their fingers and say his name several times. AB04 confirms the question of whether the world sometimes seems dead and gray to him and adds: “*Everything works, but it works like a movie, where you are totally powerless, somehow totally second row, like that.”* In addition, he described strong feelings of anhedonia: “*The whole time you just had the thought: I have to die, I have to die, I have to die. And then everything somehow passes you by, the whole rest. […] You can't enjoy anything anymore, and so on. […] I didn't take a shower for weeks, I didn't go out, and everything. Nothing at all.”* For SF03a, the feeling of being absent escalates into an objectification of the environment. He reports that he usually does not perceive lively objects in the environment with which he can interact. Instead, he feels as an observing subject detached from the world: “*That corresponds a bit to the Cartesian image of man, from Descartes, where you perceive yourself as detached from the world. And just watch. And then the world consists only of things and not of tools*.” As a result, he increasingly withdraws, lacks initiative, and feels fundamentally devitalized: “*Sometimes I feel an emptiness inside. Before I got to [name of psychiatric department], I wasn't interested in anything, enjoyed nothing. I just didn't want to do anything. I just laid in bed and did nothing. […] I was completely disinterested and lacking in drive. […] I even found it difficult to answer the ringing phone next to the bed, which I knew my mother would call to help. But to pick up the phone myself and then talk, somehow I couldn't muster the energy for it.”*

Additionally, all patients report hyperreflexivity, meaning the tendency to exaggeratedly monitor one's own sensations, emotions, and thoughts (1, 13, 20). AB04 reflects intensively on various aspects of the environment, questioning otherwise tacit aspects of everyday life. “*Why do we have money and not exchange [things]? […] With the carbon dioxide [in mineral water]: why does it bubble? […].”* His intense hyperreflexivity is not only related to a *loss of common sense* but also to a fragmentation of his cognition, body perception, and movement (see also descriptions of *disautomation* below). For instance, when reading texts, he is able to understand single words but has difficulties to grasp the content of the entire sentence or text: “*I read a sentence, the second sentence, then I have already forgotten the first one, then I skip one because it is so exhausting for me and so I go through the whole text. […] [To understand] the whole sentence, the context, and then the next one after it, this mass, or something like that, that's too much.” Or*, when standing at traffic lights, he reflects: “*I stand there and then I don't know how to stand. And then someone starts walking and then I start walking too and then I think: Mh, the other person is long gone while I still think about the traffic lights.”* The effects of hyperreflexivity seem to have the greatest effect on SF03a. This is also reflected in the frequency of category mentioning (see Table 5). In daily live, he (a) continuously reflects on social interactions and (b) sometimes even stops perceiving his environment due to intense reflection: (a) “*[When shopping] I always get out of the way of people, worry a lot about being in the way or so. I am very careful with this.”*, (b) “*I am always told that I sometimes stroll through the area, lost in thought. That is true. Sometimes I don't notice when people greet me because I'm so in my thoughts. […] I really like to think when I walk, and then it can happen that I just walk somewhere [without noticing].”* Similarly to AB04, SF03's hyperreflexivity is connected to a fragmentation of movement perception: “*How do I experience my movements? Yes, a little awkward at times. That I don't know how to move properly, like that.”* It once even escalated in the alienation from his own body perception, when he experienced an intense *psychophysical misfit* and with this a *somatic depersonalization or a bodily estrangement*. “*[…] Somehow, body and mind were now totally separate and that was a very strange bodily feeling. […] That was very unusual, like having new shoes that your feet have not yet got used to but related to the whole body. […] I was yet in the body, but yet not. […] The spirit was in the body, but it didn't quite fit. Like, when you try - I don't know - to put Windows on an Apple computer or something.”*

Finally, all subgroup patients report phenomena of *disautomation*, a category of experiences which is closely intertwined with hyperreflexivity. RM05, for instance, not only perceives his own movements as very slow but also experiences the loss of the natural self-givenness in routine actions, such as tying his shoe laces: “*Sometimes I find myself having to think about how to tie the knot. That I thought to myself: Okay, now you have to think about it somehow, even though you really don't have to.”* Similarly, IH12 experiences physical black-outs while making music, and SF03a perceives his movements as clumsy, rigid, or choppy. AB04 experiences disautomation phenomena most frequently. He reflects on the heights of stairs and reports that he no longer is able to climb stairs automatically without introspection: “*The stairs seem different heights to me. As if the levels are different, as if I had to climb higher to overcome the next stair than the other. Strange. And yet, it is the same [height] everywhere. […] All over the world it is the same [the height of the stairs], if you consider how close we go over them. It's the same everywhere, stairs are always the same, otherwise you would stumble.”* For all subgroup patients, the own movements become an object of intense reflection (hyperreflexivity) and hence do not take place automatically anymore.

### 3.4. Movement profiles and their relation to self- and body experience (H3)

#### 3.4.1. Intrapersonal comparison of MM manifestation and self- and body experiences

**Figure 1:**
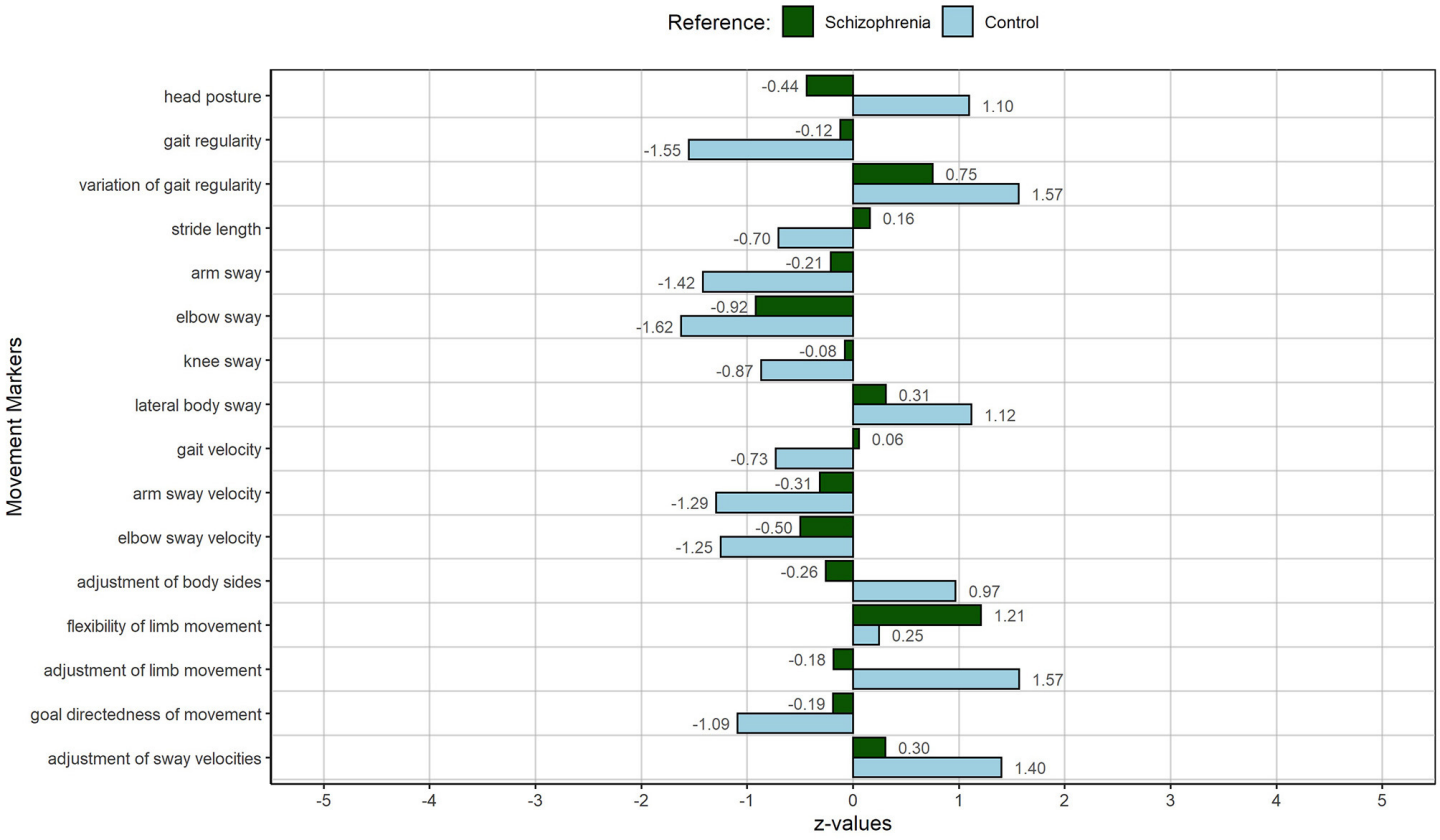
MP of RM05: The person with the lowest MI, hence, the smallest deviation from the other patients, representing the prototypical patient of the schizophrenia sample. The figure displays deviations of individual MM from the schizophrenia sample (green) and the control sample (light blue).

Considering his MM pattern, RM05 represented the prototypical patient in our study. Except one MM (flexibility of limb movement), which differs from other patients more than one SD, his movement pattern is close to the schizophrenia mean (deviation in the range of −1 to 1 SD). Compared to the mean of the study's control sample, the following MMs exceed a deviation of +/– 1 SD and hence are considered remarkable: head posture, gait regularity, variation of gait regularity, arm and elbow sway, lateral body sway, arm and elbow sway velocity, the adjustment of limb movement, the goal directedness of movement, and the adjustment of sway velocities. See Figure 1 for the exact SD values. Compared to the average control individual, RM05 walks with an increasingly slumped posture, his gait is less regular and varies more in its regularity, his arms and elbows sway less and slower while walking, and his gait is characterized by more sideways sway. Finally, during gait, the movements of his limbs and sway velocities are less adjusted (higher values in the ratios stand for less of an adjustment).

Compared to the other subset patients, RM05 presents with the lowest number of mentioned SD experiences (see Table 5). This is in line with his MM manifestation, due to which he was selected as the prototypal patient regarding his gait pattern (Figure 1). Overall, his own movement perception corresponds to the manifestation of the MM in his gait. Compared to the average control person, he presents with a reduced arm and elbow sway velocity, which is in line with his statement, that he generally moves more slowly than others. Furthermore, he experiences hyperreflexivity and disautomation in usually tacit actions, such as tying shoelaces. Although not specifically mentioned in relation to gait, disautomation is also reflected in RM05's gait: His experiences can be linked to a reduced adjustment of limbs and body sides during walking as well as to his reduced gait regularity. The reduced arm and elbow sway, the increased lateral body sway, and the loss of goal directedness can be interpreted in the same light. We can hypothesize that experiences of hyperreflexivity are intertwined with a fragmentation of successive movements, which lose their relatedness, smooth transition, and “grace” (31). In the case of RM05, less movement or a certain stiffness in limbs and diminished goal directedness during gait might be an indication of that connection. Naturally, such experiences contribute to the experience of loss of control, which, RM05 in comparison with the other subgroup patients mentions a lot.

IH12 and AB04 were selected to represent a medium deviation of MM manifestation of the schizophrenia sample. While AB04 represents a rather adverse medium deviation and hence a more pathological movement pattern than the other patients with schizophrenia, IH12 represents a rather favorable medium deviation, a healthier movement pattern than the average patient with schizophrenia. Compared to the average patient with schizophrenia in our study, AB04 presents with a slumped gait posture, an increased variation of gait regularity, an increased lateral body sway, and a decreased adjustment of limb movements (high values = less of an adjustment). IH12, in contrast, presents with an increased gait regularity compared to the other patients with schizophrenia and a decreased variation of gait regularity, hence with an overall more regular gait pattern. She displays more arm and elbow sway and a higher flexibility of limb movement than the average patient with schizophrenia in our study. Only her lateral body sway is increased in relation to the other patients.

**Figure 2:**
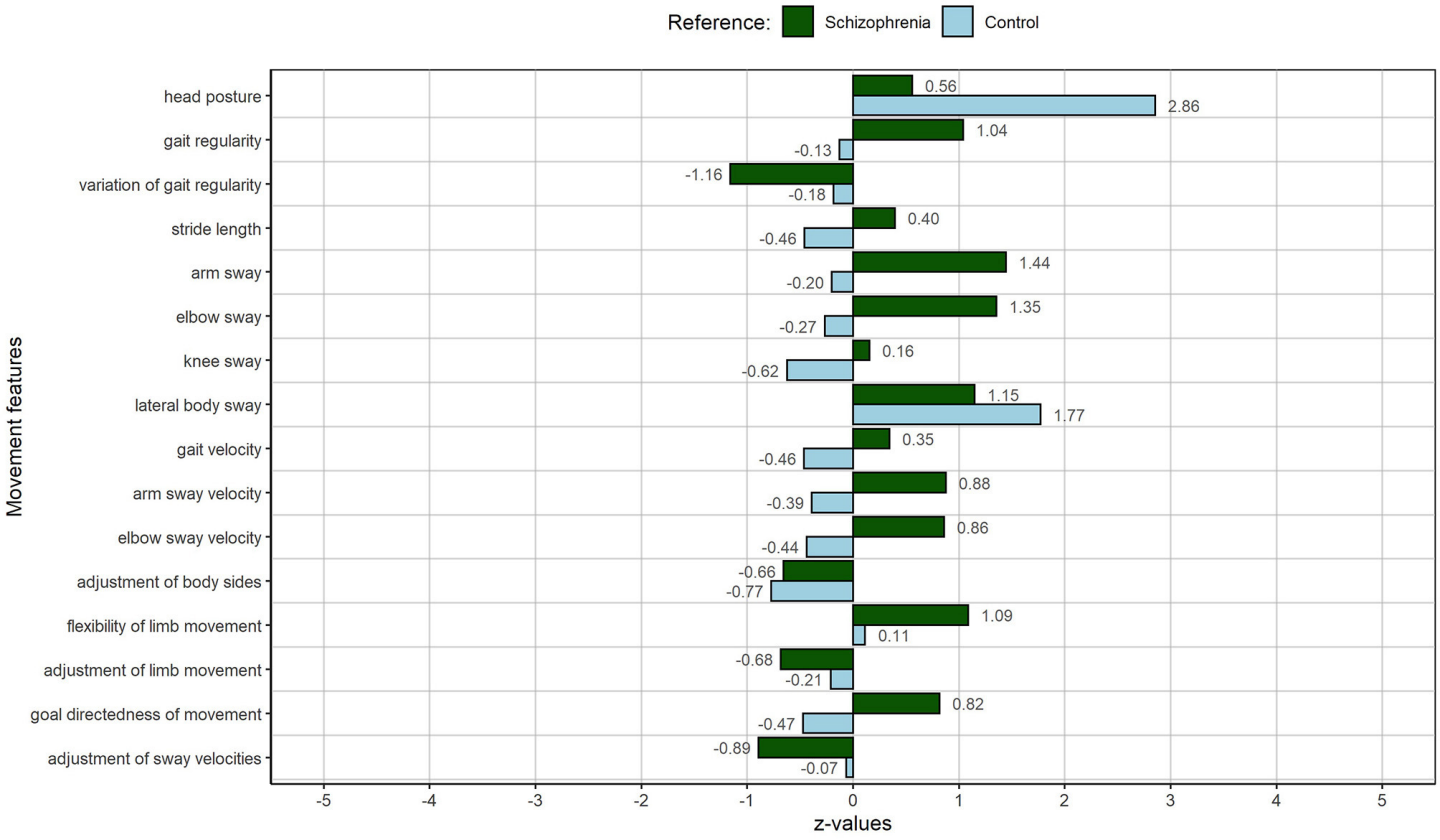
MP of IH12: The female person with an average movement index, representing a medium deviation of the schizophrenia sample. The figure displays deviations of individual MM from the schizophrenia sample (green) and the control sample (light blue).

This also applies when comparing IH12 to the average control individual of our study. Regarding most MM, she deviates very little from the average control person. Only her head posture and lateral body sway during gait are affected. See Figure 2 for the exact SD values. Compared to the average control individual of our sample, IH12 presented with a definitely increased bowing of the posture and an increased lateral body sway while walking. In addition, considering the total number of mentioned self- and body experiences, IH12 is located in the middle of the schizophrenia range. However, the total numbers of Table 5 only take into account pathological categories which are named a lot considering the statement that IH12 presents with a rather favorable movement pattern. Especially various descriptions of anxiety increase the number of mentioned pathological self- and body experiences. Likewise, the relation of MM and descriptions of anomalous experiences are not as straightforward as for RM05.

On the one hand, the increased hanging of the head and the lateral body sway—as a sign of a reduced frontal direction of the gait—can be associated with descriptions of hyperreflexivity and disautomation by IH12. As stated above, hyperreflexivity is the tendency to excessively monitor own actions. It can lead to disautomation of usually tacit actions and movements. The constant downward directedness of the gaze can be associated with a monitoring of the own steps and hence has an influence on the head posture during the gate and the directedness of it. On the other hand, it is remarkable that subjective experiences of severe anxiety, loss of control, feelings of being devitalized, experiences of disautomation, and motor blocking seem to have little effects on IH12's MM manifestation. We hypothesize that the variety of body- and movement-oriented hobbies (tennis, swimming, playing instruments, and singing), which she describes in the interview, have beneficial effects on her body and movement perception as well as her actions and movements.

**Figure 3:**
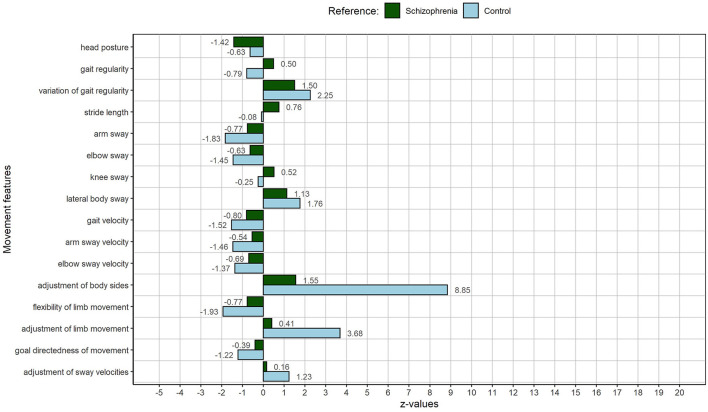
MP of AB04: The male person with an average movement index, representing a medium deviation of the schizophrenia sample. The figure displays deviations of individual MM from the schizophrenia sample (green) and the control sample (light blue).

As stated above, AB04 represents a rather adverse medium deviation of MM manifestation from the other patients. Compared to the average control participant, however, AB04 presents with many strong movement limitations during gait: higher variations of gait regularity, less arm and elbow sway, increased lateral body sway, slower overall gait, and slower movement of the limbs while walking. Especially adjustment-related MMs are impaired: He presents with a dramatically decreased adjustment of body sides, a decreased adjustment of limb movement, and his limb movement is less flexible than that of an average control person. See Figure 3 for exact SD values.

The number and intensity of remarkable MM are in line with the overall amount and content of SD descriptions by AB04. With a total number of 43 (see Table 5), he mentions the most pathological self- and body experiences during the course of the interview. Descriptions of hyperreflexivity and disautomation are directly linked to gait (standing and walking at traffic lights, and climbing stairs) and are reflected in an overall slower gait, slower movements of the limbs as well as in a dramatically decreased adjustment of body sides and limb movement, as well as less limb flexibility during gait. MM and experiences are not only directly linked, and they also relate content wise: AB04 describes to be slower than others, when crossing the street at a traffic light, due to an increased cognitive load. Higher variations of gait regularity, less arm and elbow sway, and an increased lateral body sway can be interpreted in the same light. Like in RM05, increased hyperreflexivity is accompanied by a fragmentation of gait. Not only climbing stairs but also simple walking is affected. Parts of the body, such as the arms, are not involved in the execution of the gait, and body parts are used less and less coordinated. Again, the conscious experiences and subconscious movement changes contribute to an existential self-insecurity (also here AB05 mentions the category the most), a lot of anxiety, and a feeling of lost control.

**Figure 4:**
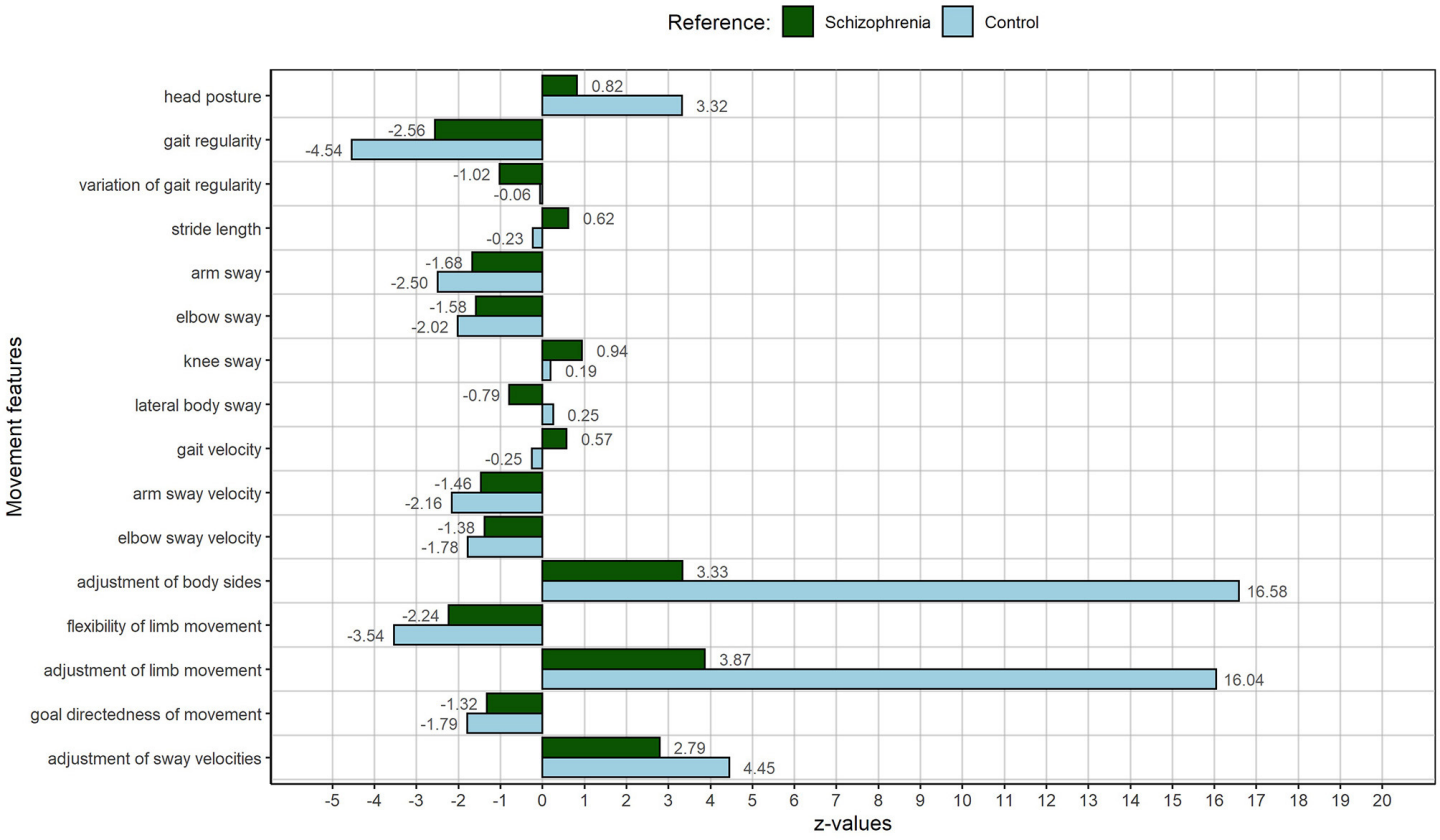
MP of SF03a: The person with the highest movement index, representing a high deviation from the other patients or the most atypical movement profile regarding the patient group. The figure displays deviations of individual MM from the schizophrenia sample (green) and the control sample (light blue).

SF03a was selected as the person with the highest deviation from the average person with schizophrenia in our study, hence as the person with the most atypical MM manifestation in relation to the other patients. He deviates strongly and adversely from the other patients in almost all MMs (see Figure 4).

SF03a's following MM manifestations are remarkable in relation to the control sample of our study: the head posture, the gait regularity, arm and elbow sway, arm and elbow sway velocities, the adjustment of body sides and of limb movement, the flexibility of limb movement, the goal directedness of movement during gait, and the adjustment of sway velocities. SF03a presents with a highly slumped posture, a strongly decreased gait regularity, and with reduced arm and elbow sway and slower usage of these limbs. Especially the adjustment of body sides and limb movement is dramatically impaired in SF03a. In addition, limb movement is less flexible and limb velocities are not well adjusted to each other during gait. Although deviating strongly in MM manifestation from the schizophrenia and control sample in our study, SF03a does not present with the most descriptions of pathological self- and body experiences during the interview, but he presents with the most severe descriptions of bodily experiences, such as a loss of body agency, a psychophysical misfit, and disautomation. He also mentions hyperreflexivity the most often. His strongly decreased gait regularity and the dramatically impaired adjustment of body sides and limb movement (fragmentation of usually tacit walking) can be associated with many hyperreflexivity experiences. Like RM05 and AB04, he also displays reduced arm and elbow sway and a slower and less flexible usage of these limbs during gait. Strong feelings of self-insecurity might be reflected in a highly slumped posture.

#### 3.4.2. Interpersonal analysis of trends

We could not find an overall trend toward more descriptions of SD experiences when MM manifestation was increased. SF03a as the person with the highest manifestation of MM in relation to the control sample did not present with the highest total mentioning of SD experiences.

However, looking at certain categories of SD experiences, we can see clear trends. For instance, the more hyperreflexivity the individuals experienced, the higher the impairment in adjustment and flexibility related MM. As stated above, hyperreflexivity seems to be intertwined with a fragmentation of successive movements (such as gait). These movements lose their relatedness, smooth transition, and “grace” (31). Often less movement of limbs or a certain stiffness in them and a reduced goal directedness during gait coincides with reduced adjustment. Not the entire body is involved in the execution of a certain movement, some body parts are used less and less coordinated, and the body is not utilized as a unity. Naturally, such experiences promote existential feelings of self-insecurity, a loss of control, and even feelings of being devitalized.

This trend can be supported by our correlational analysis of the entire sample. As stated above, many relational MM displayed a correlational trend with increased experiences of hyperreflexivity.

Furthermore, the most intense bodily experiences (loss of body agency, psychophysical misfit, and somatic depersonalization) only appear in the patient with the highest MM manifestation: SF03a. Again, this is in line with our correlational analysis: As stated above, people who displayed increased MM mostly reported body-related SD experiences, such as somatic depersonalization, psychophysical misfit, bodily disintegration, cenesthetic experiences, or general motor disturbances. Looking at correlations within the entire sample, a reduced goal directedness of limb movement, for instance, is significantly related to experiences of somatic depersonalization, a reduced flexibility of limb movement, or to feelings of bodily disintegration.

Finally, although the four subgroup patients vary in their MM manifestation, they share a common basis of anomalous self- and bodily experiences. All report existential feelings of self-insecurity, loss of control, feelings of devitalization, hyperreflexivity, and instances of disautomation.

## 4. Discussion

### 4.1. Key findings

Our study revealed the following key findings. First, the correlational analysis suggests an association of the theory-independent MM with SDs, specifically in the domain of cognition, self-experience, and bodily experiences. Stronger manifestations of the MM, hence an increased motor impairment, were related to more severe SDs (H1). Second, many MMs, primarily relational and adjustment-related ones, were associated with specific anomalous experiences. A stiffened, less coordinated and goal-directed gait is intertwined with, e.g., subjective experiences of somatic depersonalization, or the perturbation of the first-person perspective (H2). Third, MM manifestations are not precisely reflected in the individuals' descriptions of anomalous self- and body experience. However, we found clear trends of more and more intense descriptions with increasing MM manifestation, when looking at specific experiences, such as hyperreflexivity (H3).

Our findings conform to the results of the few previous studies which described associations between SDs or self-experience and motor or neurological symptoms. Hasselwander (62) found associations of balance and gait parameters with perceived self-efficacy and global cognition in people with schizophrenia spectrum disorder. Likewise, many of our MM displayed correlational trends not only with body- and self-experience but also with cognition-related anomalous experiences (EASE subdomain 1). Tonna et al. (11), who performed a stabilometric analysis with force platforms and a gait analysis with wearable sensors, found preliminary associations between SDs (EASE total scores, and domains 1, 3, and 4) and both postural and dynamic motor parameters. Gait parameters, pointing to an overall loss of gait fluidity and naturalness, were related to cognitive impairment (EASE domain 1), abnormal bodily experiences (EASE domain 3), and a loss in self demarcation (EASE domain 4). While our MMs confirm a general disruption of the gait cycle with less coordination and flexibility of limb movement, we not only found correlational trends with cognitive impairment (EASE subdomain 1) and disturbed body experience (EASE subdomain 3) but also with anomalous self-experiences (EASE subdomain 2) of the patients. Unlike Tonna et al. (11), we could not find meaningful correlations of postural markers with SDs. Associations of the head posture (our only significant postural marker) with SDs contradict our hypothesis and raise the question, of whether we may have found a spurious correlation influenced by confounding variables, such as body physicality (BMI) and medication load, or by our small sample size. Content-wise, however, the correlational patterns of postural conditions with SDs in Tonna's study resemble our SD associations with relational and adjustment-related MM. Tonna et al. (11) found that a stiffened posture (decreased sway area) with increased compensatory balancing movements was intertwined with an impairment in the subjective experience of the body (EASE subdomain 3), whereas a greater postural instability (increased sway area) with reduced compensatory movement was associated with a higher permeability of self-boundaries (EASE subdomain 4). Similarly, we found that a stiffened (less limb flexibility), less coordinated and less goal-directed gait was not only related to an overall increase in anomalous bodily experiences or a reduction in self-awareness but also to specific anomalous experiences, such as somatic depersonalization and a diminished first-person perspective. Missing reports of MM associations with specific transitivistic experiences (EASE subdomain 4) in our study are due to the fact, that we, according to our hypothesis, focused on specific experiences of EASE subdomain two and three. Future analyses might shed light on the association of our MM with experiences of pathological permeability.

Nelson et al. (41, 42) highlight hyperreflexivity as one of the cardinal facets of basic self-disturbances and propose (1) source monitoring deficits and (2) aberrant salience (including associated disturbances of memory, prediction and attention processes) as its neurological underpinnings. (1) Source monitoring deficits describe the difficulty to distinguish between endogenous (self-generated) and exogenous (externally generated) stimuli, namely efference copies or corollary discharges (CDs) (11, 41, 63, 64). An important difference between self- and externally generated stimuli is their predictability. Self-generated actions usually are highly predictable. Hence, they involve neural signals (efference copies), which are used to predict and suppress or “dampen” the sensory activity usually arising from the following proprioceptive input (65, 66). However, when self-generated stimuli are not effectively “dampened” in perception, they can be experienced as coming from the outside. For example, if tacit thoughts or sensations come to the fore, they are not automatically identified as one's own and become the center of an objectifying awareness (hyperreflexivity) (41). (2) Similarly, the term aberrant salience describes the excessive attention to information of the environment that is normally irrelevant or highly familiar (67, 68). One neurologically-based concept often mentioned within the context of aberrant salience is “latent inhibition” (69, 70). It characterizes a gating mechanism that allows organisms with complex nervous systems to cease responding to stimuli upon repeated exposure, hence known stimuli with no apparent or emotional value. A breakdown of the gating mechanisms leads to a direction of attention to fragmentary stimuli that normally go unnoticed (aspects of the environment, but also self- or bodily experiences) at the cost of the perception of larger meaningful Gestalts. Again, hyperreflexivity and disautomated perception and action are the consequence.

Supporting Nelson's statements, we found various descriptions of intense hyperreflexivity in all our subsample individuals. Often the symptom was experienced to intrude self- and bodily perception as well as the perception of the environment. Hyperreflexivity not only consistently compromised the structure of consciousness of all our subgroup patients but also systematically intensified with greater impairment in adjustment and flexibility related MM.

This also corresponds to phenomenological concepts of schizophrenia as a disturbance of the pre-reflexive temporal constitution of self-experience, as we described in a previous article (1).

### 4.2. Phenomenological classification of the results

Within the current phenomenological discourse, there are different explanatory approaches to the core disturbance of schizophrenia (1, 3, 20, 23, 24, 27, 28, 71). One group of researchers locates a disturbance of the basal self-experience (SDs) at the heart of schizophrenia psychopathology (71), and another group focuses on a disruption of the implicit bodily functioning in perception and action (a disembodiment) as the core disturbance of the illness (3, 20, 24, 27–30). In our study, the participants reported a common basis of anomalous self- and bodily experiences but presented with different manifestations of motor impairment (MM). Specific anomalous self-experiences, such as hyperreflexivity, clearly intensified with greater motor aberrations but others did not. Unconscious MM manifestations were not directly reflected in descriptions of anomalous experiences. In line with Tonna et al. (11) and Knack et al. (1), instead of understanding SDs or motor impairment as endophenotypes or core disturbances of schizophrenia symptomatology, we hypothesize them to be two consequences of a disturbed pre-reflexive, embodied experience. In fact, corollary discharges (CDs) not only differentiate between self-generated and externally generated sensations but monitor and integrate sensory-motor feedback on the actual body state and hence are responsible for the fluidity and coordination of motor flow (11, 72, 73). Understanding schizophrenia as a disturbance of embodied pre-reflexive experience means to understand the illness as an impairment of processes that constitute our consciousness *before* all (deliberate) reflection (1). Only the automatic and implicit experience and usage of the lived body [the body as tacit medium of all our experiences (74)] enables a relief of the intentional and reflexive consciousness (1, 75). I can type this text and focus on the message of the sentence without thinking about the movement of my fingers or the meaning of each individual word. The embodied, pre-reflexive experience is the basis of and comprises the entire spectrum of processes in our consciousness: Feeling, thinking, perceiving, and moving. As Knack et al. (1) points out: Thinking has, against first intuitions, a large pre-reflexive part. Seldom, we consciously put together a thought but rather follow a flow of thoughts. We do not need to ask ourselves *how* we think but can focus on *what* we think [ibid.]. It is the same with perception and movement. When seeing a clock, we do not have to put together clockface, digits, and hands of the clock but we can focus on the time. Tying shoelaces, we tie the knot without thinking about how to do it (1). According to Fuchs (76), the self-evidence of automatic processes originates from their repetitive execution. It is acquired by experience—watching, training, and practice—and stored in our bodily memory (“Leibgedächtnis”) (77). Actions, perceptions, and movements become embodied, insofar as the lived body is used as a tacit medium of experience, not as a “machine,” which has to be directed and controlled (1). Using textual examples of our subgroup patient AB04, we can demonstrate how a disturbance of the embodied pre-reflexive experience has an impact on all levels of consciousness—feeling, thinking, perceiving including self-perception, and moving. Starting with existential experiences of hyperreflexivity as a tangible and easy to describe symptom, AB04 reveals experiences of fragmented perception in reading (“*[To understand] the whole sentence, the context, and then the next one after it, this mass, or something like that, that's too much”)*, fragmented movement when walking stairs (“*The stairs seem different heights to me. As if the levels are different, as if I had to climb higher to overcome the next stair than the other.”)*, an alienation of the own thoughts and actions (“*That sounds really weird. My thoughts are clearly mine because I think them. But I don't have the feeling that they are just mine.”)*, a long-standing insecurity in self-perception and a feeling of worthlessness (“*For years I thought I had to die. […] You just have the feeling that you are worth nothing if you are supposed to be killed.”)*, and finally even a questioning of his own situatedness in the social world (“*I stand there and then I don't know how to stand. And then someone starts walking and then I start walking too and then I think: Mh, the other person is long gone while I still think about the traffic lights.”*), as well as a questioning of common sense (“*Why do we have money and not* exchange [things]? *[…] With the carbon dioxide [in mineral water]: why does it bubble? […]”)*. Taking these extensive experiences, it is no surprise that all our subgroup patients present with intense feelings of loss of control over their lives, plans, and even own bodies. The fact that MMs are related to but are not exactly reflected in the self-experience of the individuals supports the pre-reflexive nature of the disturbance underlying the schizophrenic illness. Moreover, the fact that our MMs correlate with the first three EASE subscales (cognition, self-experience and bodily experiences) further supports the idea of an underlying disturbance of the pre-reflexive experience that has an effect on all levels of consciousness.

Coming back to the neurodevelopmental underpinnings of schizophrenia psychopathology, we hypothesize that the disruption of an embodied pre-reflexive experience is rooted in the early impairment of the integration of multisensory and motor signals, a process which also has been emphasized for the development of a coherent self-experience (15, 78, 79). Chen et al. (80) identified a decreased functional activity pattern between visual areas and the primary sensorimotor area in people with schizophrenia and concluded that the sense of self relies on the spatial and temporal integration of sensory-motor signals. Similarly, Ehrsson (81) induced disturbances in self-experience (out-of-body experiences) in healthy controls when creating a mismatch between visual perception and proprioceptive signals. Early neurodevelopmental impairments in sensory-motor integration might manifest as pervasive changes in the structure of consciousness, appearing as cognitive, affective, and motor symptoms as well as changes in overall perception and as self-disorders (SDs). Ultimately, we can confirm Henriksen's (8) finding: SDs are not just another symptom cluster but part of an early alteration of the pre-reflexive experience, which gives rise to common symptoms, such as delusion, hallucination, and social withdrawal.

### 4.3. Limitations and future directions

Due to the complexity of our data assessment (movement assessment, and long and detailed interviews), we ended up with a comparably small sample size. As described in our previous publication, the sample size was an interdisciplinary compromise taking into account effect sizes of previous studies, power calculations, and the availability of the motion laboratory (31). A priori power analyses (g^*^power) suggested a total sample size between 10 and 29 for the detection of medium to large effects (*d* = 0.5–0.8), when assuming an alpha-level of *p* < 0.05 and a power of 0.8 for a correlation analysis. Because the few existing previous studies (47) found large effect sizes, and because our variables were distributed normally, we chose to calculate parametric correlations despite our small sample size. However, correlational analyses are specifically affected by small sample sizes. De Winter et al. (82), therefore, suggest the usage of different correlation coefficients across distributions and samples. Hence, in addition to the reproduction of our study with a greater sample size, future studies on the association of movement and SDs might invest some time to single out the most appropriate correlation coefficient. Furthermore, to not cover up correlational trends and hints toward associations, which might be more pronounced in bigger samples, we chose not to correct for multiple testing. In future studies, which build on hypotheses formulated on the basis of the preliminary findings of this study and operate on larger sample sizes, we ought to replicate the correlational trends with rigorous correction methods (50). Moreover, multi-methodological research poses well-known challenges, particularly in the integration of qualitative and quantitative data (83–85). Because there are hardly any standardized procedures for the integration of such different data sets, we first analyzed the data separately and afterward related the respective findings to each other on a rather exploratory or descriptive level. Future studies could benefit from the ongoing development of analysis strategies for mixed data (85). Moreover, to prevent a confirmation bias, for the composition of our subsample, we developed the movement index as a selection criterion based on the individuals' MM marker manifestation. We intended to depict the entire spectrum of MM manifestation of our schizophrenia sample. However, due to the use of absolute values, information on the direction of the MM manifestation (positive or negative deviation of the average participant with schizophrenia) was lost. This might be the reason, why IH12 and AB04, although both being selected as examples of a medium deviation of the average person with schizophrenia, differ significantly in their MM manifestation. In future studies, we would suggest using an adapted selection criterion, if in need of a subsample. One option is to reverse the polarity of certain MMs, so that all MM values point toward the same direction (e.g., higher values = more impairment). Finally, due to space limitations and to not overwhelm the reader with too much information, we chose to focus on pathological descriptions of self-experiences. The one subsample individual who described many salutogenic experiences (specifically movement-related hobbies which were associated with feelings of sovereignty), however, presents with a favorable MM manifestation in relation to the schizophrenia sample. For the purpose of a holistic research approach, in future studies we would like to systematically answer the question whether and how movement-related activities improve motor symptoms and SDs. Since instruments such as the EASE interview only assess the pathological side of self-experience and come with a relatively rigid interview structure, the question remains if data gained from their implementation is suitable for an open qualitative analysis like the one we did. Overall, our study shows that the connection of self-experience, body experience, and quantitative movement data should be considered within research, clinical examinations, and treatment of schizophrenia.

## Data availability statement

The raw data supporting the conclusions of this article will be made available by the authors, without undue reservation.

## Ethics statement

The studies involving human participants were reviewed and approved by the local Ethics Committee of the Medical Faculty of Heidelberg University (S-629/2018). The patients/participants provided their written informed consent to participate in this study. Written informed consent was obtained from the individual(s) for the publication of any potentially identifiable images or data included in this article.

## Author contributions

LM planned and conducted the study (recruitment of participants and assessment of data) and wrote the manuscript. DM transcribed the subgroup interviews. DM and LM analyzed the qualitative and quantitative data. TF supervised the study. LM, DM, and TF formed a focus group, which met regularly to discuss data assessment and analysis. LM, DM, and MK interpreted and discussed the results. LM, MK, and TF integrated the results into the current phenomenological discourse. All authors contributed to the article and approved the submitted version.
