# Supplementary material for: Relating movement markers of schizophrenia to self-experience—a mixed-methods study
Source: Front Psychiatry. 2023 Jun 21;14:1212508. doi: 10.3389/fpsyt.2023.1212508 (PMC10319999; doi:10.3389/fpsyt.2023.1212508)
Supplement: Supplementary file 1 [file Data_Sheet_1.docx]

Relating Movement Markers of Schizophrenia to Self-Experience – a Mixed-Methods Study: Supplementary Material

**Lily Martin ^1,2,5^, David Melchert^3^, Monika Knack^4^, Thomas Fuchs^2^**

1 Department Psychology, Faculty of Behavioural and Cultural Studies, University of Heidelberg, Heidelberg, Germany

2 Department of General Psychiatry, Centre for Psychosocial Medicine, Academic Medical Center, University of Heidelberg, Heidelberg, Germany

3 Department of Educational Science and Psychology, Free University Berlin, Berlin, Germany

4 Department Philosophy, Faculty of Philosophy, University of Heidelberg, Heidelberg, Germany

5 Research Institute for Creative Arts Therapies (RIArT), Department of Therapy Sciences, Alanus University of Arts and Social Sciences, Alfter, Germany

Keywords: self-disorders, movement markers, schizophrenia, Examination of Anomalous Self-Experience (EASE), mixed-methods

***Correspondence:**

Lily Martin

Hoher Wallgraben 26

10318 Berlin

+49 176 70740755

[lily.martin@uni-heidelberg.de](mailto:lily.martin@uni-heidelberg.de)

ORCID: 0000-0001-6379-3325

1. **Transcripts of EASE Interviews**

RM05_EASE_anonymized_1

| 1 | Interview RM05 |
| --- | --- |
| 2 | **I2:** Die man in äh bestimmten psychischen Erkrankungen- wir können es auch schon hier (I: Ja). Das stelle ich dann noch mal. Äh die man in ähm es geht um Erlebnisse, die man in bestimmten psychischen Erkrankungen haben kann, die meist nicht so genau Gegenstand der ersten Befragungen sind oder der ersten Interviews sozusagen und die auch in der Behandlung nicht- oft nicht so genauer zur Sprache kommen (RM05: Hm (zustimmend)), weil Sie mehr so im Hintergrund sind, so Erlebnisweisen, die man manchmal auch schon früher gehabt hat. Und die wir mithilfe eines Interviews so etwas genauer zu erfahren versuchen. (RM05: Okay.). Ähm dieses äh Interviews, ach wie das heißt, spielt letztendlich keine große Rolle oder (I: Habe ich schon erklärt, ja.) Ja. Ähm. Wir nehmen das Gespräch auf Band auf. Ich hoffe, Sie sind damit einverstanden? |
| 3 | **RM05:** Ja, das ist in Ordnung. Video war mir nicht so recht. |
| 4 | **I2:** Ja. Ist ganz in Ordnung. Äh das ist immer alles, wie- wie überhaupt das ganze Interview, vollkommen freiwillig. Und ähm es ist nur so, dass wir es gerne verwenden, wenn wir das dann auswerten, dann ist es einfach besser, man hat es noch mal äh zum Nachhören und kann es auch noch mal abschreiben (RM05: Hm (zustimmend)). Wird aber nicht irgendwo äh auswärts oder verwendet oder weitergeleitet, sondern es ist nur für unsere eigene wissenschaftliche Arbeit (RM05: Ja, alles gut) und es wird nicht mit einem Namen versehen, sondern da kommt ein anonymer- anonymes Kürzel kommt da drauf, das hat dann nichts mehr mit Ihnen zu tun (RM05: Hm (zustimmend)). Als- mit Ihrem Namen zu tun (RM05: Ja). Gut. Nur, dass Sie da ganz sicher sind. Okay. Müssen wir noch 'was klären? Ich glaube, du hast anscheinend schon alles vor- (I: Genau) besprochen, gut (I: Jederzeit Fragen stellen). Jederzeit Fragen stellen, genau. Und noch eins ist wichtig: Das Interviews kann mal ein bisschen länger dauern, und mal kürzer, weiß man vorher nicht, je nachdem, was Sie so, was Sie (RM05: so erzählen) was Ihnen so genau so an Erlebnissen einfällt (I2: lacht). Äh wenn es Ihnen genug ist, dann sagen Sie einfach Bescheid (RM05: ja). Können wir auch (RM05: alles klar) können wir auch ein anderes Mal weitermachen oder je nachdem, ja? Okay? Gut. So. Also, es geht um Themen, die- und-und Erlebnisse, die mit Veränderung zu tun haben, die im Bereich des Denkens, der Gefühle, der Körperempfindungen liegen können, aber auch so mal grundsätzlich mit dem wie man sich so im eigenen leben fühlt (RM05: Hm (zustimmend)) wie man so in der äh Welt und mit den anderen so äh sich erlebt und zurechtkommt. Also wundern Sie sich nicht, wenn die Fragen manchmal ein bisschen komisch sein können, dann sagen Sie einfach: Nee, spielt bei mir keine Rolle (RM05: Hm (zustimmend)). Ja? Und wenn Sie aber den Eindruck haben: Ja, da ist vielleicht bei mir 'was so gewesen, dann sprechen wir halt einfach mal ein bisschen dadrüber. |
| 5 | **RM05:** Alles klar. |
| 6 | **I2:** Okay? Gut. Das erste betrifft die ähm Bereich von äh Gedanken, die mal so innerlich an Gedanken und Bildern in einem abläuft (RM05: Hm (zustimmend)) äh ob man sich so im Allgemeinen gut konzentrieren kann, seinen Gedanken folgen kann, die man äh so im Kopf hat. Oder ob's da manchmal so äh Veränderungen oder Störungen oder Durcheinander gibt. Das wäre jetzt die Frage: Ob das mit Ihrem Denken, so nach Ihrem Eindruck, immer einigermaßen klar oder flüssig läuft, oder ob's da manchmal, ja, Durcheinander gibt. Brüche, dass Gedanken dazwischen kommen, die Sie eigentlich so richtig denken wollten. (RM05: Hm (nachdenklich)). Vielleicht verstehen Sie jetzt so ein bisschen schon worauf ich hinaus wil. |
| 7 | **RM05:** Also ich bin- [LACHT] ich habe das öfter so ein Kreisen von Gedanken um ein bestimmtes Thema, das mich nicht mehr loslässt (RM05: Okay, ja, ja). So'was habe ich (I2: das gehört auch dazu, ja, genau, hm) und wenn man in der ersten psychotischen Episode war, ist so, dass ich dachte, dass meine Gedanken gelesen werden können. (2I: Okay?) Also dass die frei zugänglich sind für andere Menschen. |
| 8 | **I2:** Das kennen Sie noch aus der richtigen psychotischen Zeit (RM05: Ja), sozusagen? |
| 9 | **RM05:** Genau, aus der ersten psychotischen (I2: ersten) Episode. |
| 10 | **I2:** Wann war das, wenn ich fragen darf? |
| 11 | **RM05:** ich bin gerade echt nicht mehr sicher. Ich meine so (I2: Naja, so genau jetzt nicht, also vor kurzem oder) drei, vier Jahre. |
| 12 | **I2:** Drei, vier Jahren, so'was, ja. Mh-h. Kam denn dieser Eindruck irgendwie so meine- das, was ich da denke, ist so für andere vielleicht erkenntbar oder sichtbar oder äh können das irgendwie mitbekommen? Kam das auch außerhalb der Psychose dann mal vor? |
| 13 | **RM05:** Nee, außerhalb der Psychose nicht. |
| 14 | **I2:** Nie? |
| 15 | **RM05:** Ja. |
| 16 | **I2:** Also da war immer klare Grenze, das, was Sie denken, das kann kein anderer mitdenken oder mit- mitbekommen? |
| 17 | **RM05:** Also aktuell, wenn ich jetzt im deutlich gesünderem Zustand bin, denke ich das nicht. |
| 18 | **I2:** Mh-h. Mh-h (zustimmend) aber in der Psychose? |
| 19 | **RM05:** In der Psychose, definitiv. |
| 20 | **I2:** Ähm. Das ist auch wichtig: Wir sprechen aber hier eigentlich durchaus oder fast überwiegend über Zeiten, in denen Sie jetzt nicht akut (RM05: Okay) waren, gel (RM05: Ja). Das können Sie ruhig er-ergänzen, oder (RM05: Hm (zustimmend)) erzählen auch, das ist ganz in Ordnung, aber uns interessiert auch gerade Zeiten- äh uns interessieren gerade auch Zeiten, die vorauslagen, oder mal dazwischen sind, wo Sie (RM05: Hm (zustimmend)) eigentlich jetzt nicht akut in der Erkrankung waren. |
| 21 | **RM05:** Ja, okay, alles, klar. |
| 22 | **I2:** Aber da sagten Sie jetzt vorhin, so: Grübeln, Denken, dass das Kreisen- (RM05: Kreisen um ein Thema) um ein Thema (RM05: genau.). Können Sie ein Beispiel dafür nennen? |
| 23 | **RM05:** Ähm ich mache mir zum Beispiel wahnsinnige Sorgen darüber, dass mein äh E-Mail-Account gehackt werden könnte (I2: Hm (zustimmend)). Weil das auch schon mal passiert ist (I2: Hm (zustimmend)) und ich habe den jetzt extrem abgesichert, mit Zweistufen-Verifizierung und weiß ich was. Und jetzt mache ich mir einerseits Sorgen, dass mein E-Mail-Konto gehackt wird und andererseits mache ich mir Sorgen, dass ich das Ding so sicher gemacht habe, dass ich äh quasi selbst irgendwann nicht mehr Zugriff (I2: selber nicht mehr reinkommen [LACHT]) Ja, genau. (I2: Hm (zustimmend)). Das dann so- das ist so ein Gedanke, der mich beschäftigt. |
| 24 | **I2:** Beschäftigt, ja. Ja. Also, wo Sie immer wieder darauf zurückkommen und (RM05: Genau) sich dadrum so Sorgen machen und grübeln dadrüber (RM05: Genau). Hm. Ähm. Dass Sie sich um ganz banale oder sinnlose Dinge so Gedanken machen, immer wieder darum drehen, das- das kommt eher weniger vor? |
| 25 | **RM05:** Definieren Sie sinnlos? |
| 26 | **I2:** Ja, gute Frage. Also, dass Ihr E-Mail-Account gehackt worden sein könnte, ist ja etwas, was einen schon auch wirklich beunruhigen können- (RM05: Hm (zustimmend), ja) könnte. Aber wenn Sie sich jetzt darüber Gedanken machen, äh was weiß ich, äh haben Sie heute früh die äh Fenster zugemacht? Fenster in der Wohnung zugemacht, oder nicht, oder äh- |
| 27 | **RM05:** Das kommt auch ab und an. |
| 28 | **I2:** Oder da war irgendwo im Geschäft und hat Sie angeschaut, und Sie grübeln die ganze Zeit darüber nach, wer- wer könnte das gewesen sein? |
| 29 | **RM05:** Das kommt auch ab und an vor, gerade (I2: Ja.) mit dem Fenster, oder mit 'ner Tür, die ich eventuell vergessen habe zu schließen. Das kommt ab und an vor, ja. |
| 30 | **I2:** Dass Sie dann da auch nicht mehr so richtig loskommen, davon? |
| 31 | **RM05:** Ja, genau. |
| 32 | **I2:** Oder kann es sein, dass Sie manchmal einfach den Tag so immer wieder durchgehen müssen: Was ist denn da jetzt gewesen, die letzte Stunde? Da war das, und das, und das, und dann kommen Sie immer wieder darauf zurück? |
| 33 | **RM05:** Nee, so nicht (I2: Das nicht.) So würde ich es nicht beschreiben. |
| 34 | **I2:** Also so- so sinnlos ist es nicht. |
| 35 | **RM05:** Nee, so sinnlos ist es nicht (RM05 und I2: [LACHEN]) |
| 36 | **I2:** Dass Sie so ins Grübeln kommen. Manchmal kann man auch so Gedanken haben, die sich wirklich um völlig leere Dinge drehen (RM05: Hm (zustimmend), aber das eher weniger. Sondern es sind so Sorgen: Habe ich alles zugemacht, ist alles in Ordnung? (RM05: Ja, genau) oder hat jemand den Account gehackt, und so weiter? |
| 37 | **RM05:** Genau, ja. |
| 38 | **I2:** Ja. Müssen Sie dann manchmal auch Dinge tun, die so das- die Sie dann beruhigen? Zum Beispiel das immer wieder nachkontrollieren oder immer wieder- |
| 39 | **RM05:** Ich logge mich ab und an dann einfach in mein E-Mail-Konto ein um zu (I2: Hm (zustimmend)) um mich zu vergewissern, dass alles in Ordnung ist. |
| 40 | **I2:** Hm, ja. Aber das genügt dann auch wieder? |
| 41 | **RM05:** Das genügt dann auch wieder. |
| 42 | **I2:** Das müssen Sie nicht immer wieder äh mehrmals am Tag machen? |
| 43 | **RM05:** Ich mach's nicht mehrmals am Tag. |
| 44 | **I2:** Ja, ja, ja, ja, okay. Ja. Da war also das Grübeln, und das Sichdrehen in Gedanken um solche Dinge. Äh und jetzt hatte ich vorhin noch mal gefragt: Sind- sind denn die Gedanken aber dabei sozusagen noch geordnet, oder geht das auch mal durcheinander? Wenn- oder auch sonst, wenn Sie denken? Irgendetwas nachdenken? (RM05: Mh (nachdenklich)) Sondern da so ein bisschen Salat in den Kopf kommt, wie man so sagen könnte. |
| 45 | **RM05:** Bisschen Salat im Kopf kommt ab und an, ja. (I2: [LACHT]). Könnte- würde ich durchaus so sagen, ja. |
| 46 | **I2:** Können Sie das so ein bisschen beschreiben, wie sich das anfühlt? |
| 47 | **RM05:** Dass ich quasi von Thema zu Thema springe (I2: Hm (zustimmend)). Also in einen Moment mache ich mir Gedanken über meinen E-Mail-Account (I2: Hm (zustimmend)). Äh dann mache ich mir Gedanken darüber, ob das Fenster wirklich zu ist [LACHT]. Und dann kommt wieder ein ganz anderes Thema (I2: Hm (zustimmend)). Also es äh meine Gedanken sind sehr sprunghaft (I2: Hm (zustimmend)). Und das äh merkt man auch manchmal in Gesprächen mit mir (I2: Ja.), dass ich von Thema zu Thema hüpfe (I2: Ja, Okay.). Und die andere Person guckt mich an und fragt sich: "Äh, [ANONYMISIERT, NAME DER INTERVIEWTEN PERSON], wie bist du denn jetzt da hingekommen?" |
| 48 | **I2:** Ja, ja, ja. Äh. Gibt's für Sie noch einen Zusammenhang zwischen dem einen oder anderen Thema? Sodass Sie dann irgendwie so einen, so etwas- (RM05: Es gibt einen Zu-) das eine mit dem anderen verbindet, oder? |
| 49 | **RM05:** Es gibt meistens irgendeinen Zusammenhang, der allerdings (I2: Schon noch) so abwegig ist, dass (I2: Hm (zustimmend)) man den nicht so richtig (I2: von außen erkennen könnte). Von außen könnte man- |
| 50 | **I2:** Ja, aber Sie können es noch irgendwie? |
| 51 | **RM05:** Ich kann es noch irgendwie ordnen, ja. |
| 52 | **I2:** Jeden- jede mitbeziehen- mit verbinden, ja. Ähm. Also das Sprunghafte habe ich verstanden. Aber dass Ihnen aber Gedanken jetzt wirklich mal völlig in die Quere kommen? Und Sie gar nicht wissen, wo der jetzt auf einmal herkommt? Äh irgendein äh komischer äh sich- sich dazwischen schiebender Gedanke, während Sie eigentlich gerade an etwas ganz anderes denken? |
| 53 | **RM05:** So etwas (I2: So'was nicht?) eher nicht (I2: So krass?). Nee, so krass nicht |
| 54 | **I2:** Also wirklich irgendetwas aufblitzt in Ihnen, wo Sie gar nicht- wo Sie gar nicht wissen, wo das jetzt herkommt (RM05: Hm (verneinend)) als Gedanken? |
| 55 | **RM05:** Nee. |
| 56 | **I2:** Oder dass die Gedanken mal so ein bisschen schneller geworden sind? So sich immer mehr beschleunigt haben? Dass die wie so ein Gedankenrasen entwickelt hat? Oder, nicht? Dass Sie ähm mehrere Gedanken gleichzeitig oder in schneller Fahrt, in schneller Folge, eins nach dem anderen sich da aufgedrängt hat? Dass Sie dann (RM05: Nee) nicht richtig stoppen konnten? |
| 57 | **RM05:** Nee. |
| 58 | **I2:** Das gab es auch nicht? |
| 59 | **RM05:** Das nicht. |
| 60 | **I2:** Auch nicht, ja. Das Umgekehrte: Dass manchmal Gedanken einfach plötzlich wegwaren, wie- wie so ein Filmriss, wie so ein- |
| 61 | **RM05:** Wenn ich von etwas abgelenkt wurde, zum Beispiel? |
| 62 | **I2:** Ja, Ablenken meine ich gar nicht mal nur so, dass man plötzlich von etwas anderem ähm, irgendwas, was einen beschäftigt, ablenkt. Das ist ja nachvollziehbar (RM05: Hm (zustimmend)), oder so. Ohne solchen konkreten äußeren Anlass Ihnen die Gedanken einfach gestoppt wurden, oder dass die blockiert waren plötzlich? |
| 63 | **RM05:** Eher nicht. |
| 64 | **I2:** Das auch nicht, hm. Oder- oder sich plötzlich so verflüss- verflüchtigt haben? So irgendwie verblasst? |
| 65 | **RM05:** Das kenne ich. |
| 66 | **I2:** Dass Sie es nicht mehr fassen konnten? |
| 67 | **RM05:** Das kenne ich (I2: Ja?). Dass so ein Gedanke einfach mal nach und nach einfach verschwindet (I2: Ja.), meinen Sie? |
| 68 | **I2:** Ja. |
| 69 | **RM05:** Ja, ja, ja. |
| 70 | **I2:** Genau (RM05: Ja), so etwas meine ich, ja. Dass irgendwie so- dass man das Gefühl hat: Das verliert sich jetzt. Also, das geht- das kann ich nicht mehr fassen. |
| 71 | **RM05:** Hm, ja, das kenne ich. |
| 72 | **I2:** Das kennen Sie? Aha. Kann man schwer beschreiben, gel? |
| 73 | **RM05:** Kann man sehr schwer beschreiben (RM05 und I2: [LACHEN]). |
| 74 | **I2:** Oder können Sie es nochmal versuchen, das zu beschreiben? |
| 75 | **RM05:** Äh naja, es ist halt so, dass manchmal ein Gedanke da ist und dann verblasst er, geht in den Hintergrund. Und ich weiß dann nicht mehr so recht ähm was ich gerade- oder ich frage mich dann, worüber ich gerade nachgedacht habe (I2: Ja, so'was). Verstehen Sie, was (I2: Mh-h, mh-h (zustimmend)) ich meine? |
| 76 | **I2:** Ja, ja, ja, genau. |
| 77 | **RM05:** Was hast du- worüber hast du jetzt eigentlich die letzten zehn Sekunden gegrübelt? |
| 78 | **I2:** Ja, ja, ja, okay. (RM05: Dass das-) Das kann also relativ schnell dann, wenn Sie zehn Sekunden sagen (RM05: Ja, genau) ähm dass man plötzlich nicht mehr weiß: Was war jetzt das eigentlich gerade? |
| 79 | **RM05:** Ja, genau. |
| 80 | **I2:** Okay. So etwas kennen Sie schon, ja. Und äh auch ein bisschen ungewöhnlich, selten, aber kommt vor, dass die Gedanken so sich äh so bissl wiederholt haben, aber nicht so, dass Sie es wie bei dem Gedankenkreisen immer wieder durchdenken, sondern dass Sie das Gefühl hatten, der Gedanke, den hören Sie fast noch mal? Oder der kommt noch mal nachgesprochen? Oder so etwas Ähnliches? |
| 81 | **RM05:** Hm ich habe so etwas (I2: In Ihrem Kopf so nachklingt?) in die Richtung, also, so'n Nachklingen, also dass ich tatsächlich was höre, nicht (I2: Hm (zustimmend)). Aber zum Beispiel wiederhole ich relativ oft äh so völlig banale Textzeilen aus Liedern in meinem Kopf. |
| 82 | **I2: Mh-h** (zustimmend), wie ein Ohrwurm? |
| 83 | **RM05:** Nee (I2: Sozusagen), kein Ohrwurm (I2: Das ist nicht-), kein Ohrwurm (I2: Mh-h (zustimmend)), sondern einfach nur die Textzeilen, ohne die Musik dazu. |
| 84 | **I2:** Mh-h, mh-h (zustimmend). Kommt immer wieder dann? |
| 85 | **RM05:** Genau, dann sage ich das so. |
| 86 | **I2:** Zehn Mal, zwanzig Mal? |
| 87 | **RM05:** Genau. |
| 88 | **I2:** Dreißig Mal? |
| 89 | **RM05:** Genau. |
| 90 | **I2:** Aha. Und können Sie es dann auch wieder stoppen, oder? |
| 91 | **RM05:** Ich kann es irgendwann wieder stoppen (I2: Schon.), wenn ich mich (I2: Ja.) fokussiere. Aber ich habe dann wirklich Phasen, wo ich dasitze und vor mich hingrüble und ich habe immer wieder diese Textzeile aus dem einen Lied, dass ich die wiederhole (I2: Mh-h (zustimmend)) und die wiederholt sich und wiederholt sich in meinem Kopf (I2: Hm (zustimmend)) und ich denk mir: Komm, kannst du mal- was denkst du da eigentlich darüber nach? |
| 92 | **I2:** Kratzer in der Schallplatte, hat man früher gesagt, aber (RM05: Ja.) Schallplatten kennt man heute kaum noch [LACHT]. Also wo Sie so eine (RM05: Ja.)- wo so was hängenbleibt. |
| 93 | **RM05:** Ah, Schallplatten sind was Schönes (I: [LACHT]). |
| 94 | **I2:** Sie kennen Sie schon noch, ja? |
| 95 | **RM05:** Ja. (I2: [LACHT] okay.) Ja, im Vergleich zu CD's: Die Dinger sind einfach unzerstörbar (I2: Ja.). Die sind für die Ewigkeit gebaut. |
| 96 | **I2:** Ja gut, aber es gab eben die Kratzer, nicht? Und dann (RM05: Ja, ja, ja) und dann konnte es mal sein, dass das- eine Rille hängenbleibt und dass man plötzlich dasselbe immer wieder gehört hat (RM05: Ja.). So deswegen habe ich das jetzt erinnert, ja? Mh-h. Also das kennen Sie so ein bisschen, so. Das wiederholt sich. Gedanken, die Ihnen fremd vorkamen? Ich habe so etwas Ähnliches schon mal gefragt. Die, wo Sie das Gefühl, die kommen von woanders? Oder sind Ihnen von jemand einge- hinein- äh in den Kopf geschickt, oder? |
| 97 | **RM05:** Nee, nee (I2: Das gab es auch nicht), so was nicht. |
| 98 | **I2:** Das kennen Sie nicht. Ahm. Ja. Sehr unangenehme, die Sie mal- also das, was Sie beschrieben haben, ist ja gut, dass Sie über das- diesen ähm, diesen E-Mail-Account nachdenken, ja, okay, das ist nachvollziehbar. Oder dass Sie diese Liedzeilen immer wieder haben. Aber gab's manchmal, dass Ihnen Gedanken oder sehr unangenehme Gedanken in den Kopf kommen, die Sie schnell loswerden wollen? Irgendwas Scheußliches, was Ihnen immer wieder als Bild oder Gedanke in den Kopf kommt? |
| 99 | **RM05:** Ja, definitiv. |
| 100 | **I2:** Zwangsgedanke sagt man, äh was man- was man eigentlich loswerden möchte, schnell. Aber es geht nicht so schnell weg. |
| 101 | **RM05:** Seit meiner ersten psych- psychotischen Episode habe ich den Gedanken, dass mein alter Arbeitgeber mich verklagen wird (I2: Mh-h, mh-h (zustimmend)), weil ich bin sehr lange mit starker Negativsymptomatik arbeiten gegangen (I2: Mh-h (zustimmend), ja.). Ich denke halt immer wieder, dass ich aufgezeichnet werde, wenn- in den Dingen, die ich sage und tue (I2: Hm (zustimmend)). Das kommt hin und wieder vor und dass ich dann eben quasi- jetzt habe ich den Faden verloren [LACHT]. |
| 102 | **I2:** [LACHT]. Das hatten wir ja vorher als (RM05: das hatten wir vorher) als eine der (RM05: als eine der ja) Symptome, oder Phänomene, die man da erleben kann. Nee, Sie waren dabei zu sagen, dass Sie denn äh da Angst haben, dass der Arbeitgeber Sie (RM05: mich verklagen könnte) Sie verklagen könnte, genau. Weil Sie? |
| 103 | **RM05:** Weil ich im Endeffekt ähm, ich kanns nicht so richtig (I2: weil, wegen der Negativsymptomatik, dann), wegen der Negativsymptomatik (I2: gut arbeiten konnten, damals) nicht gut arbeiten konnte. Und ich habe auch schon mit meinem Arbeitgeber telefoniert und die haben gesagt: "[ANONYMISIERT, NAME DER INTERVIEWTEN PERSON], es ist alles in Ordnung. Gute Genesung" (I2: Mh-h (zustimmend)), aber es kommen trotzdem immer wieder diese negativen Gedanken (I2: Hm (zustimmend)), dass was sein könnte (I2: Ja, ja.), weil ich auch der Meinung bin, dass es einzeilne Personen in dem Unternehmen gibt, die mir nicht wohlgesonnen sind (I2: Mh-h, mh-h (zustimmend)). Und äh das ist so ein Gedanke, den kriege ich nicht weg (I2: Kriegen Sie auch nicht weg, ja). Den kriege ich nicht weg. Also da helfen die Medikamente nix (I2: Mh-h (zustimmend)), da komme was wolle (I2: Mh-h (zustimmend)). Ich könnte noch zehn Mal meinen Arbeitgeber anrufen und der könnte mir auch noch zehn Mal sagen, dass alles in Ordnung ist. |
| 104 | **I2:** Das wissen Sie eigentlich? |
| 105 | **RM05:** Genau. |
| 106 | **I2:** Dass das in Ordnung ist (RM05: Genau, aber-). Also der Gedanke ist eigentlich unsinnig, oder? |
| 107 | **RM05:** Der Gedanke ist komplett unsinnig (I2: unsinnig, ja), aber er kommt und er macht mir Panik. |
| 108 | **I2:** Ja, genau. Mh-h. Also schon so ein Zwangsgedanke, wie man- wie wir es nennen. Etwas, das man unsinnig findet, aber trotzdem quälend immer wieder kommt. |
| 109 | **RM05:** Genau. |
| 110 | **I2:** Ja, mh-h. Mh-h. Rituale, Rit- also so äh was- was Sie immer wiederholen müssen, tun müssen? Jetzt nicht das Denken, sondern etwas, was so im Alltag, Sie- |
| 111 | **RM05:** Rauchen. |
| 112 | **I2:** Gut, das ist kein- das ist kein Zwang, sondern eine Sucht [LACHT]. Gut, okay, also rauchen. |
| 113 | **RM05:** Also was ich- was ich ab und an mache, ist ähm mir die Hände reiben [MACHT REIBENDES GERÄUSCH MIT DEM HÄNDEN]. Das ist so- das muss ich immer mal wieder ab und an machen (I2: Hm (zustimmend)), insbesondere, wenn ich nervös bin (I2: Ja, gut, das ist). Und ich merke, ich nehme das gar nicht wahr, dass ich (I2: Hm (zustimmend)) das noch mache. |
| 114 | **I2:** Hm, okay. Ist jetzt auch noch etwas relativ Harmloses. |
| 115 | **RM05:** Ja, genau. |
| 116 | **I2:** Gut, aber Sie haben schon recht. Das gehört zu so- bisschen tickartig, dass man das nicht so richtig mitbekommt, was man da die ganze Zeit macht. Okay. Ich glaube, das waren jetzt so die wichtigen Fragen zu Gedanken. Wie gesagt, das Gedanken äh Sie manchmal das Gefühl hatten, andere könnten das lesen, hatten Sie ja gesagt, aber das war eben nur in der Psychose? |
| 117 | **RM05:** In der Psychose, ja. |
| 118 | **I2:** Ja. Passiert es. Mithören können, oder mit- oder einfach mitbekommen? Mitkriegen? |
| 119 | **RM05:** Mithören. |
| 120 | **I2:** Mithören, tatsächlich? |
| 121 | **RM05:** Ja. |
| 122 | **I2:** Weil Sie es auch so ein bisschen laut gehört haben, oder Ihre eigenen Gedanken so etwas laut waren, oder nicht deswegen? |
| 123 | **RM05:** Nicht deswegen. Ich hatte einfach nur den Eindruck, dass die das alles mithören können. |
| 124 | **I2:** Hören können, ja. Mh-h. Ähm, kam es mal vor, dass Sie Gedanken äh, ist eine komische Frage, aber so was gibt es, dass man äh das Gefühl hat, die Gedanken, die sind da irgendwo in bestimmten Bereichen, so zu lokalisieren, quasi? Die sind da links, oder rechts (RM05: Hm, nee). Oder wandern da im Kopf herum, oder (RM05: Nee.) so etwas? Nee? Im Körper? Das hatten Sie nicht. Okay. Das war jetzt, glaube ich mal, das Wichtigste zu den Gedanken. Äh so in den alltäglichen Situationen, wenn Sie da mal so zurückdenken, kommt es öfter mal vor, dass Sie sich da schwertun äh ja einfache Dinge zu tun, zu entscheiden, sich für etwas zu einschließen, beim Einkaufen oder sonst wo, oder sowas? |
| 125 | **RM05:** Definitiv. Einkaufen mit mir ist der Hammer (I2: Ist [LACHT]). Ich kann mich vor so ein Regal stellen (I2: So was zum Beispiel, ja). Ich kann mich Stunden damit beschäftigen, zur Not (I2: Okay.). Ich gucke mir alles ganz genau an und vergleiche (I2: Mh-h (zustimmend)). Ich habe eine gewisse Entscheidungsarmut, möchte ich sagen (I2: So was, ja, ja, also-) Also es fällt mir extrem schwer, Entscheidungen zu treffen |
| 126 | **I2:** Ja. Und zwar gerade so, auch so, wo Sie selber merken, äh wissen, dass es eigentlich gar nicht so wichtig ist, oder? |
| 127 | **RM05:** Ja bei- gerade bei banalen Dingen. |
| 128 | **I2:** Gerade bei banalen Dingen? |
| 129 | **RM05:** Ja. |
| 130 | **I2:** Ja. Also was anderes ist ja, wenn es um was wirklich was geht. Aber da beim Einkaufen geht es ja eigentlich um nicht viel. |
| 131 | **RM05:** Da geht es um nicht viel (I2: Ja.), aber (I2: aber es kann trotzdem lange dauern.) um mal ein Beispiel zu nennen (I2: Ja, gern): Kennen Sie das [ANONYMISIERT, NAME EINES RESTAURANTS] hier in [ANONYMISIERT, ORT IN DEUTSCHLAND]? |
| 132 | **I2:** Hm (I: Ja). |
| 133 | **RM05:** Das ist ein Restaurant, die haben 100 verschiedene Schnitzel (I: Mh-h (zustimmend), I2: Oh Gott, ja.). Und es ist im Endeffekt immer dasselbe mit eben einer anderen Sauce oder anderen Gewürzen (I2: Mh-h (zustimmend)). Aber das war die Hölle für mich. Ich saß da erstmal eine halbe Stunde und musste mir die alle ganz genau anschauen, bevor ich eine Entscheidung treffen konnte. |
| 134 | **I2:** Mh, mh, mh. Irgendein Kriterium zu finden versuchen, wonach man es entsch- wonach man es auswählt. |
| 135 | **RM05:** Am Ende habe ich was völlig Banales genommen. Habe einfach ein Zigeunerschnitzel gegessen (I2: Mh-h (zustimmend)). Also, ich glaube das war sogar der vierte, oder, das vierte oder das fünfte Schnitzen auf der Karte (I2: Mh-h (zustimmend)). So was ganz Normales. |
| 136 | **I2:** Vor lauter Verzweiflung dann einfach irgendwas genommen. Aber Sie haben richtig eine halbe Stunde darüber nachgedacht? |
| 137 | **RM05:** Ich habe eine halbe Stunde darüber nachgedacht. |
| 138 | **I2:** Hm. So etwas geht Ihnen auch öfter ahm öfter mal so? Ich weiß nicht, beim Anziehen? Ob morgens man in der Früh anzieht- |
| 139 | **RM05:** Das kann passieren. |
| 140 | **I2:** Kann auch passieren. Mh-h. Ambivalenz nennen wir das, aber das tut jetzt nichts zur Sache. Sie haben es schon beschrieben, so eine Entscheidungsschwäche oder Unfähigkeit, da (RM05: Ja.) Entscheidungen zu treffen, ja? |
| 141 | **RM05:** Ja |
| 142 | **I2:** Ähm. Gibt es manchmal auch Schwierigkeiten, so in Gang zu kommen? Etwas umzusetzen, was Sie sich eigentlich vorgenommen haben? Das ist dann so, ja, manchmal gibt es so Aufschieberei, das kennt jeder von uns, ne? Aber ich meine jetzt mehr so was äh wo man wirklich blockiert ist in dem, was man eigentlich als nächstes machen will. Bin ich denn sch- nicht hineinkommt, in das Handeln. In das, was man eigentlich tun möchte. Dann so eine- |
| 143 | **RM05:** Ich will mir seit Monaten ein neues Bett kaufen. |
| 144 | **I2:** Mh-h, mh-h. Und, gelingt nicht? |
| 145 | **RM05:** Ich guck mir immer wieder Prospekte an (I2: Mh-h) und bin dann unschlüssig, welches ich nehmen soll. Wo wir dann wieder beim Thema Ambivalenz sind (I2: Mh-h, genau), ein Stück weit. Aber ich krieg mich aktuell nicht aufgerafft dazu, einfach mal in ein Möbelhaus zu fahren und zu sagen: Okay, das ist es jetzt und das nehme ich jetzt. |
| 146 | **I2:** Mh-h, mh-h (zustimmend). Also, obwohl Sie es eigentlich wollen (RM05: Obwohl ich eigentlich will) und brauchen. |
| 147 | **RM05:** Ja, genau. |
| 148 | **I2:** Ja. Okay. Das hat auch noch mit viel mit Entscheidungsschwierigkeit zu tun, glaube ich, ja? |
| 149 | **RM05:** Ja. |
| 150 | **I2:** Mh. Und sonst? Im Laufe des Tages? Dinge tun, die Sie tun wollen. Was weiß ich, einkaufen gehen, oder klappt jetzt schon? |
| 151 | **RM05:** Also, ich muss sagen, mit der neuen Medikation ist es besser geworden, (I2: Mh-h (zustimmend)) ein Stück weit (I2: Mh-h (zustimmend)), aber früher habe ich sehr, sehr viel aufgeschoben (I2: Mh-h (zustimmend)). Also ich hatte auch schon so Phasen, wo ich wirklich drei Wochen nur im Bett verbracht habe (I2: Mh-h (zustimmend)) und an die Decke gestarrt habe (I2: Mh-h, mh-h (zustimmend)). Und dann, das Schlimme war, ich habe quasi nichts getan (I2: Mh-h (zustimmend)) und habe mir davon Vorwürfe gemacht, dass ich nichts getan habe. |
| 152 | **I2:** Mh-h, dann fang- find das Grübeln über- fing das Grübeln an? |
| 153 | **RM05:** Genau. |
| 154 | **I2:** Das Gedankenkreisen (RM05: Das Gedankenkreisen, ja). Warum hast du jetzt nichts gemacht? |
| 155 | **RM05:** Ja. |
| 156 | **I2:** Ja. Kann ich mir vorstellen. Ein anderer Punkt noch: Ähm, kommt es manchmal vor, dass Sie bei etwas hängenbleiben? Ja, so kleine Details die Ihnen noch auffallen in Ihrer Umgebung? Was weiß ich, dass Sie von Kleinigkeiten abgelenkt sind, die Sie immer wieder anschauen müssen? Oder so auf einem Detail von irgendeinem Bild hängenbleiben? Und da genau hinschauen müssten, oder? |
| 157 | **RM05:** So was kenne ich nicht. |
| 158 | **I2:** So was kennen Sie nicht, ja. Kommt es Ihnen- äh haben Sie manchmal Schwierigkeiten so, sich zu konzentrieren im Sinne von, ja einer Person zuhören, wenn andere da mit dabei sind, oder? |
| 159 | **RM05:** Ja, das kann vorkommen. |
| 160 | **I2:** Das kann vorkommen. So, Aufmerksamkeit wirklich zu fokussieren, auf etwas zu richten, wenn viele andere ablenkende (RM05: Ich) Reize da oder Dinge da sind. |
| 161 | **RM05:** Ich bin sehr geräuschempfindlich, tatsächlich. |
| 162 | **I2:** Ja. Dass Sie das rasch ablenkt? |
| 163 | **RM05:** Ja. |
| 164 | **I2:** Ja. Das kann vorkommen. Oder wenn irgendwo ein Fernseher läuft, dass Sie dann- oder ein Radio, dass Sie da leicht ablenkbar sind, oder sich nicht konzentrieren können dann auf etwas? |
| 165 | **RM05:** Ja, ich drehe gerade zu Hause komplett durch. Also seit meiner Erkrankung wohne ich bei meinen Eltern (I2: Mh-h (zustimmend)) bis ich wieder auf die- auf meinen eigenen Beinen stehen kann (I2: Mh-h (zustimmend)) und äh mein Vater ist mittlerweile 78 und er bräuchte dringend ein Hörgerät (I2: Mh-h (zustimmend)). Und der Fernseher läuft so laut und mich stört das quasi wirklich daran- also es ist nicht laut in meinem Zimmer. Aber schon dieses ganz leise Fiepsen, was ich da im Hintergrund höre, treibt mich manchmal völlig in den Wahnsinn (I2: Mh-h (zustimmend)) und sorgt dafür, dass ich auch so ein Stück weit handlungsunfähig bin, weil ich mich zu abgelenkt fühle (I2: Mh-h, mh-h (zustimmend)). Verstehen Sie? |
| 166 | **I2:** Ja, ja, ja. Durchaus. Eigentlich durch ein harmloses oder gar nicht mal so lautes Geräusch, das irgendwo im Hintergrund ist (RM05: Ja.), das reicht schon (RM05: Ja.). Hm, ja. Wie ist so denn Gedächtnis? Manchmal Schwierigkeiten gehabt, sich ähm ein Buch zu lesen, sich einen Film anzuschauen? Weil Sie das dann wieder vergessen hatten, was Sie gelesen oder gesehen hatten? |
| 167 | **RM05:** Ja, das hatte ich schon mal. Also ich hatte schon mal so eine Phase, da konnte ich keine drei Sätze am Stück lesen (I2: Mh-h, mh-h (zustimmend)), oder beziehungsweise musste wieder von vorne anfangen (I2: Vorne anfangen, so was, ja). Oder ich habe mal geschafft, eine komplette Seite zu lesen und dachte mir dann auch: Eigentlich habe ich gar nicht verstanden, was jetzt (I2: Mh-h (zustimmend)) da drin stand. |
| 168 | **I2:** Das ist praktisch leer geblieben (RM05: Ja.), wenn man irgendwas leer liest und dann nicht, ja (RM05: Ja), gar nicht den Sinn ver- mit aufnimmt (RM05: Ja.). (I2: Mh-h (zustimmend)), ja. Also das hat auch mit Aufmerksamkeit zu tun, aber Gedächtnis so ähm eng damit verbunden, ja? Dass man sich dann halt merken kann, was gewesen ist die letzten fünf Minuten. Das kann Ihnen auch manchmal schwer fallen? |
| 169 | **RM05:** Ja. |
| 170 | **I2:** Mh. Wie ist es mit Zeitgefühl? Zeiterleben? Hat sich da manchmal etwas verändert? Das Gefühl hatten, weiß nicht: Läuft alles plötzlich ganz schnell oder ganz langsam, oder? |
| 171 | **RM05:** Also, gerade auf Station [ANONYMISIERT, NAME DER ÖRTLICHEN PSYCHIATRISCHEN STATION] habe ich den Eindruck, dass alles extrem langsam läuft (I2: Mh-h (zustimmend)), weil ich mittlerweile wieder gesünder bin und ich mich (I2: Mh-h (zustimmend)) schlicht und ergreifend bisschen langweile. |
| 172 | **I2:** Ach, okay, gut. Langeweile ist nochmal was relativ- (RM05: Das ist nochmal was anderes) ja gut, auch interessant, aber das kennen- kennt man natürlich, wenn man das Gefühl hat: So, jetzt kenne ich alles. Und äh jetzt müsste mal was Neues passieren. |
| 173 | **RM05:** Ja, genau. |
| 174 | **I2:** Aber das ist das generelle Gefühl, dass die Zeit irgendwie jetzt langsamer abläuft? |
| 175 | **RM05:** Nee. |
| 176 | **I2:** Oder schneller, auf der anderen Seite, ja. Dass mal die- es Ihnen so vorkam, dass die Zeit irgendwie sich so verändert, dass es irgendwie plötzlich nicht mehr die Zukunft weiter gibt? Oder das kam auch nicht vor? |
| 177 | **RM05:** Nee, das kam nicht vor. |
| 178 | **I2:** Ja also so richtig merkwürdige Zeitstörungen kennen Sie nicht? |
| 179 | **RM05:** Nee. |
| 180 | **I2:** Langeweile ist noch relativ (RM05: Okay) nachvollziehbar [LACHT]. Mal- dass es mal Lücken gegeben hat in Ihrem Gedächtnis, in Ihrem Bewusstsein? Dass Sie das Gefühl hatten: Da war jetzt so ein Sprung? Da weiß ich gar nicht mehr, was davor war oder was ich gerade gemacht habe? Wie ich überhaupt dahinkomme, wo ich jetzt bin? (I2: Hm (überlegend)). Also so Lücken, Aussetzer sozusagen im Gedächtnis? |
| 181 | **RM05:** Ja, das kann schon mal vorkommen, (I2: Kann auch mal vorkommen) ja. |
| 182 | **I2:** Mh. Oder dass Sie nicht sicher waren: Habe ich das jetzt eigentlich erlebt, was ich erleb- was ich da erlebt habe? War das jetzt tatsächlich? Oder habe ich es mir nur eingebildet oder nur fantasiert? |
| 183 | **RM05:** Nee, so was nicht. |
| 184 | **I2:** So was kennen Sie nicht? |
| 185 | **RM05:** So was in die Richtung nicht (I2: Ja.). Aber dass ich manchmal irgendwo stehe und mich frage: Hoppla, wie bist du denn jetzt dahin gekommen? (I2: Zum Bei- ja genau, so was) Auch weil ich in Gedanken war, zum (I2: Mh-h (zustimmend)) Beispiel (I2: Mh-h, mh-h (zustimmend)). Das kann vorkommen. |
| 186 | **I2:** Ja, ja. Also schon so, dass man wie in Trance irgendwo hingegangen ist und nicht weiß (RM05: Ja), wie das eigentlich gekommen ist (RM05: Ja) und auf einmal bist du da und weißt nicht wie. |
| 187 | **RM05:** Ja, genau (RM05 und I2: [LACHEN]). |
| 188 | **I2:** Unangenehm, oder? So ein bisschen. |
| 189 | **RM05:** Ja, bisschen unangenehm. |
| 190 | **I2:** Mh, mh. Ähm gab es mal Schwierigkeiten, dass Sie das, was Sie so in Gedanken hatten, in Worte fassen? Gut, ja, bis zu einem gewissen Grad kennt das auch jeder. Äh ich meine halt so, dass Sie das Gefühl hatten: Jetzt wollte ich doch eigentlich was ganz anderes sagen? Was habe ich denn da jetzt Komisches gesprochen? |
| 191 | **RM05:** Ja. Ja, das kenne ich. |
| 192 | **I2:** Kommt da was aus dem Mund sozusagen, was ich gar nicht richtig- ja, passt gar nicht richtig zu dem, was ich eigentlich sagen wollte? |
| 193 | **RM05:** Ja, das kenne ich. |
| 194 | **I2:** Das kennen Sie? |
| 195 | **RM05:** Ja |
| 196 | **I2:** Erinnern Sie sich an ein Beispiel? |
| 197 | **RM05:** Uff müsste ich jetzt kurz nachdenken. Fällt mir jetzt- |
| 198 | **I2:** Fällt Ihnen jetzt nichts mehr ein. Aber Sie erinnern sich, dass es das gab (RM05: Ja.). Wissen Sie noch ziemlich genau. Kommt es oft vor, oder? |
| 199 | **RM05:** Nee, eher selten. |
| 200 | **I2:** Eher selten, schon. Genau. Ähm, ja. Also generell haben Sie schon das Gefühl, die Worte finden zu können um etwas zu beantworten? |
| 201 | **RM05:** Generell finde ich die Worte, um irgendwas zu beantworten (I2: Zu Beantworten oder zu sagen, was Sie sagen wollen). Aber ich bin man- ich bin manchmal in so einer Situation, wo ich mir denke: Oh Gott [ANONYMISIERT, NAME DER INTERVIEWTEN PERSON], wieso hast du jetzt das gesagt? (I2: Mh-h (zustimmend)) Das äh- |
| 202 | **I2:** Ist Ihnen so rausgerutscht? |
| 203 | **RM05:** Ist mir so rausgerutscht, genau. |
| 204 | **I2:** Mh-h, mh-h, mh-h, okay. Ja. Äh dass Sie es nicht vorher gut überlegen und dann merken: Es passt eigentlich gar nicht so richtig. |
| 205 | **RM05:** Ja. |
| 206 | **I2:** Mh-h, ja. Jetzt kommen wir zu einem anderen Bereich, der hat so mit dem- Geht es noch, oder? |
| 207 | **RM05:** Ja, geht noch, alles gut. |
| 208 | **I2:** Gut, dem anderen Bereich, der hat so mit dem Gefühl, hm, zu tun, wie-wie spüre ich mich eigentlich? Wie bin ich so da? Bin ich da [LACHT]? Bin ich überhaupt anwesend? (RM05: Hm (zustimmend) Gegenüber den- spüre ich mich so richtig? Äh- |
| 209 | **RM05:** Ganz großes Thema bei mir. |
| 210 | **I2:** Ganz großes Thema, dann (RM05: Ja.) sagen vielleicht einfach selber, wie das sich für Sie anfühlt oder nicht anfühlt. Ich will es Ihnen möglichst wenig vorgeben (RM05 und I2: [LACHEN]). |
| 211 | **RM05:** Also ich habe das- das- |
| 212 | **I2:** Sie wissen, worauf ich hinauswill. So das Gef- gründsätzliche Gefühl um Dasein, ja? Um Hiersein. |
| 213 | **RM05:** Also ich neige extrem dazu, in Gedanken abzudriften (I2: Mh-h, mh-h (zustimmend)). Und zwar auch äh, zum Beispiel, wenn sich gerade eine Person mit mir unterhält und im nächsten Moment bin ich wie weg (I2: Ja.) und äh bin in meinen Gedanken und kann das so schnell auch nicht unterbinden. Bis dann irgendjemand so neben mir steht und sagt: [ANONYMISIERT, NAME DER INTERVIEWTEN PERSON] Bist du noch da? (schnippt dabei mehrfach mit den Fingern). |
| 214 | **I2:** Bist du noch da? Mh-h, mh-h (bestätigend). |
| 215 | **RM05:** Es ist so ähnlich wei bei: Kennen Sie die Folge [ANONYMISIERT: NAME EINER SERIE] zufällig? |
| 216 | **I2:** Nee, leider nicht. |
| 217 | **RM05:** Kennen Sie die? |
| 218 | **I:** Ich glaube schon. (RM05: Der-) Das sind Ärzte? |
| 219 | **RM05:** Ja, (I: Ja.) genau. Der J.D., der Hauptcharakter, der driftet auch immer wieder so in Gedanken (I: Mh-h (zustimmend)) ab (I: Mh-h (zustimmend)). Und da habe ich mich irgendwie stark wiedererkannt. |
| 220 | **I2:** Mh-h (bestätigend). Äh ist einfach nicht so richtig präsent geworden oder (RM05: nicht so richtig präsent), wenn andere da sind (RM05: Ja) und äh es zieht ihn so weg und (RM05: genau) ja, ja, okay. Also das ist ein großes Thema? |
| 221 | **RM05:** Das ist ein sehr großes Thema bei mir. |
| 222 | **I2:** Ist Ihnen das unangenehm auch, oder? |
| 223 | **RM05:** Die meisten Leute lachen irgendwann darüber, wenn sie mich kennen. |
| 224 | **I2:** Aha. Wie so jemand, der immer so ein bisschen geistesabwesend ist, oder? |
| 225 | **RM05:** Ja, genau. |
| 226 | **I2:** Irgendwie nicht richtig bei der Sache ist, wie man so sagt. |
| 227 | **RM05:** Ja. |
| 228 | **I2:** Mh-h, okay. Äh was ich äh das ist eines- ein Teil von dem, was ich- worauf ich auch noch hinauswollte, ein anderes [/eine andere/] Möglichkeit ist, dass man nicht nur in Gedanken so abdriftet, sondern auch grundsätzlich mehr das Gefühl hat: Ich bin gar nicht so richtig da. Ich bin- nicht jetzt bei anderen, sondern auch wenn Sie mit sich alleine sind: Ich spüre mich nicht so richtig, bin gar nicht so richtig (RM05: So nicht.) auf der Welt, wenn Sie so wollen. |
| 229 | **RM05:** So nicht. |
| 230 | **I2:** Das kennen Sie nicht? |
| 231 | **RM05:** Nee, das kenne ich nicht. |
| 232 | **I2:** So, dass Sie sich eher selbst ganz fremd fühlen? Oder hier auf der Welt ganz fremd fühlen? |
| 233 | **RM05:** Nee. |
| 234 | **I2:** Wie komme ich jetzt überhaupt auf diese Welt? Oder wer bin ich überhaupt? |
| 235 | **RM05:** Nee, so extrem ist es nicht. |
| 236 | **I2:** Mh-h (zustimmend), dass Sie sich über sich selber so bohrend fragen müssen: Sitze ich eigentlich hier? We-we-wer bin ich? |
| 237 | **RM05:** Nee. |
| 238 | **I2:** Warum komme ich eigentlich auf die Welt? Warum bin ich überhaupt da? [LACHT] etwas komische Fragen. |
| 239 | **RM05:** Also, dass ich mir nach dem Sinn des Lebens quasi (RM05: Ja.) die Frage stelle? |
| 240 | **I2:** Hm nein, das meinte ich gar nicht so sehr (RM05: Okay.). Wirklich mehr so dieses, wie wenn man auf die Welt gefallen ist und nicht weiß, wie das- wie das gekommen ist. |
| 241 | **RM05:** Nee, so extrem nicht. |
| 242 | **I2:** [LACHT] so- so kennen Sie es nicht, ja. Ähm oder dass man ganz anders ist als alle anderen Menschen und eigentlich gar nicht auf dem richtigen Planeten, also so was wie- |
| 243 | **RM05:** Den Eindruck habe ich manchmal. |
| 244 | **I2:** [LACHT] den Eindruck haben Sie schon. |
| 245 | **RM05:** Den Eindruck habe ich schon manchmal, dass ich (I2: Hm (zustimmend)) mir irgendwie denke: Okay, ich bin irgendwie anders als die anderen (I2: Hm (zustimmend)). Was ja auch tatsächlich so ist (I2: Hm (zustimmend)), aber nicht in dem Ausmaß, das ich mir manchmal vorstelle (I2: Mh-h (zustimmend)). Verstehen Sie, was ich meine? |
| 246 | **I2:** Noch nicht ganz, dass Sie meinen ähm dass Sie- |
| 247 | **RM05:** Hm (zustimmend) also ich fühle mich deutlich anders, als andere Personen (I2: Ja, mh-h). Was, glaube ich auch ein Stück weit mit meiner Erkrankung zusammen hängt (RM05: Kann sein.). Aber ich fühle mich damit oft zu alleine (I2: Mh-h, mh-h (zustimmend)). Also ich fühle mich oft damit zu alleine, im Vergleich- jetzt habe ich wieder den Faden verloren. Wo war ich gerade? |
| 248 | **I2:** Sie sagten, äh, dass äh Sie sich oft ähm ja ganz anders als andere Menschen fühlen und damit alleine fühlen, und nicht ähm- |
| 249 | **RM05:** Ja, genau, dass ich mich damit alleine fühle (I2: dass Sie es nicht erklären können vielleicht, oder) genau, genau. Das kann ich ni- dass kann ich mir nicht so richtig erklären, ja. |
| 250 | **I2:** Mh-h, mh-h (zustimmend). Oder anderen auch nicht so richtig erklären? |
| 251 | **RM05:** Anderen auch nicht so richtig erklären, nee (I2: Mh-h (zustimmend)). Nicht so wirklich. |
| 252 | **I2:** In welcher Weise sind Sie denn anders (RM05: Hm (überlegend))? Oder könnten Sie anders sein? |
| 253 | **RM05:** [MACHT SCHNALZENDES GERÄUSCH] also ich bin halt sehr anders durch meine Tagträumerei (I2: Ah ja, mh-h, mh-h (zustimmend)) und da denke ich mir immer ähm: Was ist eigentlich los mit mir? |
| 254 | **I2:** Mh-h (zustimmend). Dann könnten Sie ja vielleicht diese Tagträumerei ein kleines bisschen beschreiben? Oder ist das unangenehm? |
| 255 | **RM05:** Ähm nee, kann ich (RM05: nee) gerne ein bisschen beschreiben. |
| 256 | **I2:** Wie das abläuft? |
| 257 | **RM05:** Also es läuft quasi so ab, dass ich äh in dem einen Moment in einem Gespräch mit jemandem bin (I2: Mh-h (zustimmend)) und im nächsten Moment drifte ich ab (I2: Mh-h (zustimmend)) und denke zum Beispiel, äh was ich vorhin gesagt habe, mit so einem Musiktitel (I2: Mh-h (zustimmend)). Der si- wo sich der Text immer (I2: wiederholt), immer wieder wiederholt (I2: Ja, mh-h (zustimmend). Dass si- dass so etwas passiert. Oder (I2: Mh-h (zustimmend)) dass ich abdrifte und mir völlig abstruse Dinge vorstelle (I2: Mh-h (zustimmend)). Also keine Ahnung. |
| 258 | **I2:** Macht nichts. Sagen Sie es ruhig. Kann ruhig so abstrus sein, wie- |
| 259 | **RM05:** Nee, also (I2: es Ihnen einfällt [LACHT]). Mir fällt gerade nichts so richtig Abstruses ein, das ist das Problem [LACHT]. |
| 260 | **I2:** Macht nichts. |
| 261 | **RM05:** Ähm. Nee, aber einfach so Dinge, die irgendwie irreal sind. |
| 262 | **I2:** Ja. Ja. Mh-h (zustimmend). Das machen Sie selber oder kommt es mehr so über Sie? Dass Sie so, ja. Tagträume kann man einfach entweder sozusagen so sich dem hingeben oder man fängt selber aktiv so ein bisschen sich in so etwas hineinzufantasieren. |
| 263 | **RM05:** Ich gebe mich dem- also es passiert automatisch. |
| 264 | **I2:** Passiert mehr mit automatisch (I2: Ja), ja. Und ähm sind es dann auch Bilder? Erinnerungen? |
| 265 | **RM05:** Bilder, (I2: Vorstellungen?) Erinnerungen. |
| 266 | **I2:** Bilder auch, ja? Werden die recht konkret, so dass Sie dann fast so ein bisschen wie anschauen können (RM05: Ja) in Ihrem Geist, sozusagen? |
| 267 | **RM05:** In meinem Geiste kann ich mir das sehr gut anschauen. Ich (I2: Mh-h (zustimmend)) rede zum Beispiel wahnsinnig gerne über äh- ich muss dazu vorher erklären: Ich war ein Jahr lang in [ANONYMISIERT: NAME EINES LANDES] mit [ANONYMISIERT: NAME EINER NGO] (I2: Mh-h, mh-h (zustimmend)) und Paviane sind die Hölle. |
| 268 | **I2:** Wieso? [LACHT] |
| 269 | **RM05:** Sie müssen sich vorstellen: Um in mein Haus zu gelangen, hat der eine Pavian sein Babypavian durch mein Fenster geschmissen. Der Pavian ist durch das Haus gelaufen. Hat sich alles zu Essen gekrallt, was er irgendwie finden konnte (I2: und es mit rausgenommen) und ist mit rausgelaufen (RM05, I und I2: [LACHEN]) und so etwas- so etwas stelle ich mir dann zum Beispiel (I2: Okay.) zwischendurch immer mal wieder vor (I2: Mh-h (zustimmend)) und das ist dann in meinem Kopf (I2: Mh-h (zustimmend)) und dann grinse ich. Und dann gucken mich die Leute an und sagen mir: [ANONYMISIERT, NAME DER INTERVIEWTEN PERSON], wieso grinst du? Ich sage: Ja, Gedankenkarussel. |
| 270 | **I2:** Mh-h, mh-h, mh-h (zustimmend. Okay. Äh, das ist in dem Fall ja auch eine Art Erinnerung? |
| 271 | **RM05:** Ja, genau. |
| 272 | **I2:** Wahrscheinlich, ja. Und Sie haben das ja miterlebt, so. |
| 273 | **RM05:** Ja. |
| 274 | **I2:** Mh-h (zustimmend) und dann taucht das so auf und Sie gehen es wieder durch sozusagen, wie das- (RM05: Genau, ja) wie der da in alle Zimmer geht (RM05: Ja) und durch alle Zimmer streicht da. |
| 275 | **RM05:** Genau. |
| 276 | **I2:** Ähm ist es für Sie besonders äh wie soll man es sagen? Ist es ganz lebendig für Sie? Dass das wirklich wie- wie so etwas- wie fast so ein Film, dann in Ihnen abläuft, den Sie anschauen können? |
| 277 | **RM05:** Ja. Würde ich sagen. |
| 278 | **I2:** Oder bloß so ganz kurz, ja? Es kann ja, wie andere sagen- |
| 279 | **RM05:** Nee, ich bin in dem Moment so richtig weg. |
| 280 | **I2:** Sind- sind so richtig weg und dadrinnen? |
| 281 | **RM05:** Ich bin so richtig weg (I2: Ja.) und da drinnen. |
| 282 | **I2:** Ja, ja. Sodass Sie es also quasi mitverfolgen, oder so sich mit anschauen? |
| 283 | **RM05:** Ja. |
| 284 | **I2:** Mh-h (zustimmend) und das richtig- da richtig, ja, im Virtual Reality sagt man so: "Immersion", dass man da richtig eingetaucht ist (RM05: Hm (zustimmend)), ja, in diese Welt dann. |
| 285 | **RM05:** Ich bin richtig weg. |
| 286 | **I2:** Sind richtig drin? |
| 287 | **RM05:** Ja. |
| 288 | **I2:** Und müssen sich dann so wieder rausholen? Oder von jemand anderem wieder? |
| 289 | **RM05:** Ich brauche dann: [ANONYMISIERT, NAME DER INTERVIEWTEN PERSON] [SCHNIPST MEHRFACH MIT DEN FINGERN]. |
| 290 | **I2:** Mh, genau, okay. Mh-h. Ähm. Kam es mal vor, dass Ihnen diese Bilder auch so- ja, dass die sich verselbstständigen? Dass die quasi ablaufen, ohne dass Sie äh ja, dass wirklich, wie das ist so wie einen Film anschauen. Von selber laufen, oder? |
| 291 | **RM05:** Also dass mit dem- (I2: im äußeren Film sind?) das mit dem Pavian könnte ich mir- ich verstehe die Frage gerade nicht. |
| 292 | **I2:**  Ja, also es ist natürlich nicht sehr gut formuliert gewesen. Ich wollte damit sagen, äh dass Sie das Gefühl hatten: So, das ist jetzt wirklich damals. Äh sitze ich da und schaue diesem Film, ja? Der in mir abläuft. (RM05: Mh-h, mh-h (zustimmend)) So ein bisschen. Ja? Dass wir denken, das ist jetzt wie- wie wenn mir mein Geist da etwas vorführt, was- was (RM05: Ja) aufführt. So einen Film zeigt (RM05: Ja, ja, ja.) Ja? Äh dass so wie- so ein bisschen Abstand dazu haben (RM05: Ja.). So-so ähnlich könnte man es beschreiben. |
| 293 | **RM05:** Könnte man so gut beschreiben. |
| 294 | **I2:** Ja, okay. Mh-h. Das ist so richtig, also, oder ein richtig [/richtiger/] Tagtraum, in den man ganz- |
| 295 | **RM05:** versinkt. |
| 296 | **I2:** Versinkt, ja (RM05: Ja.). Genau. Und das meinen Sie ist so ein bisschen, was Sie so anders macht? |
| 297 | **RM05:** Was mich anders macht, ja. |
| 298 | **I2:** Also wo Sie auch aus der Gemeinschaft von anderen oft mal so herausfallen? |
| 299 | **RM05:** Ja, genau (I2: Hm (zustimmend)) das ist jetzt noch das- abgesehen von meiner Erkrankung, (I2: Ja.) die ich habe, was mich anders macht, ja. |
| 300 | **I2:** Ja. Okay. Aha, gut. Dass Sie da mal das Gefühl hatten- ich habe so etwas Ähnliches schon mal gefragt, aber nur zur Sicherheit: Das- das- diese- zum Beispiel diese Tagträume oder die Gedanken oder dass-dass das gar nicht- dass Sie gar nicht richtig äh dass das- dass das nicht Ihre eigenen sind. Dass das Sie- nicht Sie selbst sind, der das denkt oder der das erlebt oder der das erinnert, oder. |
| 301 | **RM05:** So etwas hatte ich mal, ja. |
| 302 | **I2:** Ja? |
| 303 | **RM05:** Also ich hatte konkret- jetzt muss ich dazu sagen: Das war jetzt nicht äh in- |
| 304 | **I2:** Nicht in der Psychose? |
| 305 | **RM05:** Das war in der Psychose. |
| 306 | **I2:** Ah in der Psychose, ja, okay, mh-h (zustimmend). |
| 307 | **RM05:** Das war in der Psychose und äh ich habe mich quasi wie ein anderer Mensch verhalten (I2: Mh-h (zustimmend)). Also ich habe quasi- wie beschreibe ich das? Ich habe mich so verhalten, wie jemand, den ich kenne und (I2: Mh-h (zustimmend)) habe quasi die Entscheidungen getroffen, die er getroffen hätte (I2: Mh-h (zustimmend)). Verstehen Sie, (I2: Ja.) was ich meine? |
| 308 | **I2:** Ja, ja, ja. Mh-h. Das Gefühl äh plötzlich jemand- ja fast ein bisschen wie jemand anders zu sein. |
| 309 | **RM05:** Ja, genau. |
| 310 | **I2:** Mh-h (zustimmend). War aber so eine eher besondere Situation? |
| 311 | **RM05:** War eine besondere Situation. |
| 312 | **I2:** Kam nicht öfter? |
| 313 | **RM05:** Nee, nee, kam nicht öfter vor. |
| 314 | **I2:** Jemand, den Sie gut kannten, oder? |
| 315 | **RM05:** Ja, jemand, den ich gut (I2: Hm (zustimmend)) kannte. |
| 316 | **I2:** Und waren vielleicht auch gar nicht mehr sicher: Wer bin ich? Ich oder der andere? |
| 317 | **RM05:** Ja. |
| 318 | **I2:** So in die Richtung? |
| 319 | **RM05:** Ja. |
| 320 | **I2:** Mh-h (RM05: Aber das-) war so so mehr in der Psychose, ja? |
| 321 | **RM05:** Das war in der Psychose. |
| 322 | **I2:** Das kommt jetzt sonst nicht vor, dass Sie- |
| 323 | **RM05:** Das kommt sonst nicht vor, nee. |
| 324 | **I2:** Irgendwie sonst mal sich so zwei- zweigeteilt erleben, oder so? |
| 325 | **RM05:** Nee, mh-h (verneinend). |
| 326 | **I2:** Oder Gefühl haben, ich bin vielleicht jemand ganz anderes oder so etwas? |
| 327 | **RM05:** Nee, nee, nee. |
| 328 | **I2: Das** kam Ihnen- (RM05: Das kommt-) das bekommen Sie sonst nicht? |
| 329 | **RM05:** Nee, das kommt nur in der Psychose vor. |
| 330 | **I2:** Dieses Gefühl, mal ganz anders zu sein und mit den Tagträumen, das habe ich gut verstanden. Kennen Sie das auch von früher? Manchmal von- hat man so etwas schon als Kind mal erlebt. Ich- irgendwie bin ich ganz anders als die (RM05: Hm (zustimmend)) als die Freunde und Klassenkameraden. Bin irgendwie so ein bisschen wie vom Mars? [LACHT] Irgendwas (RM05: Hm (überlegend)) anderes? Irgendjemand? |
| 331 | **RM05:** Ja, also (I2: Der nicht so richtig dazu gehört? Nicht so) das- (I2: richtig hineinpasst in diese Welt?) Ja, das wurde so ein bisschen äh verstärkt dadurch, dass ich in der Grundschule (I2: Mh-h (zustimmend)) war ich der einzige Junge, der aufs Gymnasium geschickt wurde (I2: Mh-h (zustimmend)). Und entsprechend wurde ich in der Grundschule, weil Kinder sind grausam (I2: Ja.), können Sie sich vorstellen (I2: Genau.). Und ich wurde gehänselt, weil ich war der Einserschüler (I2: Ah) und die anderen waren (I2: Mh-h, mh-h, mh-h (zustimmend)) so durchschnittlich (I2: Mh-h (zustimmend)) und sind (I2: Mh-h (zustimmend)) auf die Realschule (I2: Mh-h (zustimmend)) und die Hauptschule geschickt worden und war halt wirklich der einzige. |
| 332 | **I2:** Ja, gut. Aber in dem Punkt waren Sie ja dann auch wirklich anders. |
| 333 | **RM05:** Ja, genau. |
| 334 | **I2:** War sehr unangenehm, so ein Gefühl, aber es ist jetzt nicht so ein- es ist nachvollziehbar aus der Situation (RM05: Ja.), wollte ich sagen (RM05: Ja.). Sie waren ja auch so ein bisschen äh der Primus oder der mh (überlegend)- |
| 335 | **RM05:** Das ist auch deutlich besser geworden als ich dann aufs Gymnasium gekommen bin. |
| 336 | **I2:** Mh, klar, wenn man dann wieder- |
| 337 | **RM05:** Da war ich einer (I2: Ja.) von vielen. |
| 338 | **I2:** Vielen. Insofern war es jetzt kein Grundgefühl, äh das Sie immer hatten, sondern das war halt durch diese Situation bedingt? |
| 339 | **RM05:** Ja, genau. |
| 340 | **I2:** Von der Volksschule, ja. Von der Grundschule. Okay. Also, das Gefühl ähm, dass alles, was Sie da erlebt haben, was Sie beschrieben haben: Gehört gar nicht zu Ihnen? Das sind Sie gar nicht selbst? Das haben Sie glaube ich nicht gehabt. Sagten Sie vorhin, also dass äh so eine grundsätzliche Selbstentfremdung. Ich spür- ich bin gar nicht der, der das jetzt erlebt. (RM05: Hm (überlegend)) Ich bin gar nicht der, der das jetzt spürt oder sieht oder wahrnimmt? |
| 341 | **RM05:** Nee, das war nur diese kurze Phase (I2: Ja.) in der Psychose (I2: Ja, die Psychose selber, ja), ja. |
| 342 | **I2:** Mh-h. Ja. Kam Ihnen denn die Umgebung manchmal verändert vor? Wenn Sie sich selbst schon äh also wenn es nicht bei Ihnen selbst war, dass Sie sich ganz anders oder fremd in der Welt vorkamen, kann es ja mal umgekehrt sein, dass Sie das Gefühl hatten: Die Umgebung, die Welt hat sich irgendwie verändert, ist ganz komisch, ganz (RM05: Hm (überlegend)), weiß nicht, wie ein Traum? |
| 343 | **RM05:** Hm nee, so etwas hatte ich nicht. |
| 344 | **I2:** Ganz fremd? |
| 345 | **RM05:** Nee. |
| 346 | **I2:** Oder Dinge bedeuten irgendetwas, was Sie manchmal nicht so genau erkennen oder- erklären oder erkennen können? Also alles hat so eine Bedeutsamkeit für Sie? |
| 347 | **RM05:** Ja, das kenne ich. |
| 348 | **I2:** Könnten Sie das vielleicht selber mit eigenen Worten beschreiben? |
| 349 | **RM05:** Hm (überlegend) also Sie müssen wissen, ich habe äh das war allerdings auch in der Psychose. |
| 350 | **I2:** Okay, war wieder eine besondere Situation? |
| 351 | **RM05:** War wieder eine besondere Situation. |
| 352 | **I2:** Vielleicht können Sie es trotzdem kurz schildern? |
| 353 | **RM05:** Äh ich habe in der Psychose äh hatte ich ja wahnsinnige Angst, dass ich verklagt werde (I2: Mh-h (zustimmend)) und habe gedacht, meine (RM05: von dem Arbeitgeber, also was Sie jetzt noch immer wieder?), genau, meine Arbeitskollegen haben mich aufgezeichnet und (I2: Mh-h (zustimmend)) meine ganze Wohnung ist verwanzt (I2: Mh-h (zustimmend), genau) und mein Smartphone ist verwanzt weiß ich, was noch alles sein könnte (I2: Mh-h (zustimmend)). Und ich habe in der Psychose ein Videospiel gespielt. [ANONYMISIERT: NAME DES VIDEOSPIELS] (I2: Mh-h (zustimmend)). Und ähm in diesem Videosp- dieses Videospiel habe ich quasi auf mich bezogen. Also (I2: Mh-h (zustimmend)) ich habe die Handlung von diesem Videospiel auf meine Situation bezogen (I2: Mh-h (zustimmend)) und am Ende des Videospiels gab es ein Kapitel, das nannte sich "Alles ist gut" (I2: Mh-h (zustimmend)) und dann war ich auf einmal unfassbar erleichtert. (I2: Also das war-) Weil alles gut war (RM05: vorhatten, ja-), weil alles gut war. |
| 354 | **I2:** Ja, ja. Okay. Mh-h, verstehe. Und das Spiel haben Sie dann immer wieder gespielt, oder? |
| 355 | **RM05:** Einmal (RM05: Einmal.) Das hat gereicht. |
| 356 | **I2:** Okay [LACHT]. |
| 357 | **RM05:** Aber am Ende war alles gut (Gleichzeitig I: Das dauert, ne?). |
| 358 | **I2:** Das dauert? |
| 359 | **RM05:** Ja, ja, ja, das hat bestimmt 40 Stunden oder so beansprucht. |
| 360 | **I2:** 40 Stunden? (laut) |
| 361 | **RM05:** Ja, garantiert. |
| 362 | **I2:** wow, das ist aber lang. Mh-h. Dann waren Sie da auch wirklich mitten drin. |
| 363 | **RM05:** War ich mitten drin, ja. |
| 364 | **I2:** Alles auf Sie- hat mit Ihnen zu tun gehabt? |
| 365 | **RM05:** Ja, genau. |
| 366 | **I2:** Und war nicht zufällig, oder so, sondern alles war irgendwie auf Sie ausgerichtet? |
| 367 | **RM05:** Alles war auf mich ausgerichtet, ja. |
| 368 | **I2:** Mh-h. Waren Sie sozusagen im Mittelpunkt? |
| 369 | **RM05:** Ich war im Mittelpunkt. |
| 370 | **I2:** Ja, wenn alles sich um Sie bewegt sozusagen? |
| 371 | **RM05:** Ja. |
| 372 | **I2:** Das war jetzt diese Situation bei dem Video in- auch in der Psychose. Gab es denn das jetzt auch sonst mal, dass Sie so dieses Gefühl hatten im Mittelpunkt zu sein? Was weiß ich, wenn man auf der Straße geht und das Gefühl hat, alles passiert so, weil es- weil ich ich gerade jetzt da bin? |
| 373 | **RM05:** Außerhalb der Psychose nicht. |
| 374 | **I2:** Nie? Nein. |
| 375 | **RM05:** Nee. |
| 376 | **I2:** Mh-h. Kennen Sie das sonst nicht. |
| 377 | **RM05:** Nee. |
| 378 | **I2:** Ja. Ja. [MACHT GERÄUSCHE MIT DEM MUND] [PAUSE] Das Gefühl, mal ähm verändert zu sein, in Bezug auf Ihren Körper? Wir kommen auf den Körper nochmal zu sprechen, aber (RM05: Mh-h (zustimmend)) dass Sie das Gefühl hatten, ich gehöre gar nicht irgendwie da in diesen Körper? Oder, oder vielleicht waren Sie nicht sicher, ob Sie das Geschlecht haben, dass Sie jetzt eigentlich haben? Oder dass es eine Fremdheit gab zu Ihrem, ja, einfach zu Ihrem Körper oder zu Ihrem Alter? Oder hatten jetzt ein anderes Lebensalter oder gehören in einen ganz anderen Körper hinein? Oder ich bin gar nicht sicher, ob ich ein Mann oder eine Frau bin? |
| 379 | **RM05:** Hm nee. |
| 380 | **I2:** Das war- war alles nie ein Problem? |
| 381 | **RM05:** Nee. |
| 382 | **I2:** Ja. Identität kann man dann sagen, also so bin ich "Ich" oder- (RM05: Mh-h (zustimmend), nee) das war Ihnen nie eine Frage? |
| 383 | **RM05:** Nee. |
| 384 | **I2:** Dass Sie Sie selbst sind? |
| 385 | **RM05:** Dass ich ich selbst bin, (I2: [LACHT] okay) war mir immer bewusst. |
| 386 | **I2:** So ungefähr, wer Sie sind, ja? |
| 387 | **RM05:** Ich wusste so ungefähr, wer ich bin (I2: [LACHT] okay). Ich habe mich zwischendurch, weil ich auch eine wahnhafte Phase hatte, (I2: Mh-h (zustimmend)) hatte ich mich maßlos überschätzt (I2: Mh-h, mh-h (zustimmend)). Aber so- ich war immer ich selbst (I2: Ja, ja. Okay.). Bis auf diese kurze Phase, wo ich quasi (I2: Ja.) wie jemand anders gehandelt habe. |
| 388 | **I2:** Das hatten Sie erzählt, wo Sie den Bekannten oder Freund da so quasi wie sich selbst erlebt haben, ja. |
| 389 | **RM05:** Ja. |
| 390 | **I2:** Mh-h (zustimmend). Ähm. Ängste? Mein Gott, Ängste sind so häufig, aber vielleicht trotzdem: Könnten Sie nochmal beschreiben, ob es außerhalb der Psychose jetzt wieder (RM05: Hm (zustimmend)) auch Phasen, Zeiten, gibt, in denen Sie stark unter Ängsten leiden? |
| 391 | **RM05:** Ja, definitiv. |
| 392 | **I2:** Welche Art sind die dann? |
| 393 | **RM05:** Ich habe Angst vorm Autofahren (I2: Mh-h (zustimmend)) und- |
| 394 | **I2:** Spezifisch Autofahren? |
| 395 | **RM05:** Genau. Autofahren und ähm ja nee, Autofahren ist es primär (I2: Ja.). Also das ist auch etwas, das außerhalb der Psychose stattfindet (I2: Ja.). Allerdings ist es zum ersten Mal aufgetreten nach meiner ersten psychotischen Episode. |
| 396 | **I2:** Ah, ja. Gab es da irgendein Erlebnis, dass Sie darauf gebracht hat, dass Autofahren irgendwie bedrohlich sein könnte? |
| 397 | **RM05:** Ich hatte einen Unfall. |
| 398 | **I2:** Achso. Ja, dann verstehe ich es aber auch. |
| 399 | **RM05:** Aber der war relativ harmlos. Ich bin (I2: Mh-h (zustimmend)) quasi mit zehn Stundenkilometern bin ich rückwärts gefahren und habe jemand, der hinter mir stand, bisschen gerammt. |
| 400 | **I2:** [LACHT] Ist jetzt nicht dramatisch, ja? |
| 401 | **RM05:** Es ist nicht dramatisch. Aber seitdem habe ich wirklich (I2: Mh-h (zustimmend)) große Angst vorm Autofahren (I2: Mh-h, mh-h (zustimmend)) und äh- |
| 402 | **I2:** Sie setzen sich selber gar nicht mehr in ein Auto, oder- |
| 403 | **RM05:** Ich setze mich- ich setze mich noch in ein Auto, aber ungewohnte Strecken sind das Problem (I2: Mh-h (zustimmend)). Also ich fahr- trau mich aktuell nur auf Strecken, die mir bekannt. Zum Beispiel der Weg zur Arbeit (I2: Mh-h (zustimmend)). Das ist kein Problem, weil den bin ich schon 200 Mal oder so gefahren (I2: Mh-h, mh-h (zustimmend)). Da fühle ich mich relativ sicher- |
| 404 | **I2:** Also können Sie selber auch fahren? |
| 405 | **RM05:** Genau. Da kann ich selber fahren (I2: Ja.). Aber wenn man mir jetzt sagen würde: "Äh, [ANONYMISIERT, NAME DER INTERVIEWTEN PERSON], fahr morgen nach [ANONYMISIERT: NAME EINES ORTES IN DEUTSCHLAND]" (I2: Mh-h (zustimmend)), dann würde ich sagen: "Oh Gott, oh Gott, lieber nicht." (I2: Mh-h (zustimmend)). Oder: "Fahr an irgendeinen Ort, der dir unbekannt ist" (I2: Mh-h (zustimmend)) oder irgendwas in die Richtung und ich mache mir halt große Sorgen, dass mir beim Unfall irgendwas passieren könnte. |
| 406 | **I2:** Aha, aha. Seit dieser kleinen Sachen da? |
| 407 | **RM05:** Seit dieser kleinen (I2: Mh-h (zustimmend)) Sache da (I2: Mh-h (zustimmend)), genau. |
| 408 | **I2:** Mh-h. Also schon auch fast so ein bisschen Tod- also Todesangst klingt vielleicht dramatisch. Oder Angst, dass Ihnen wirklich etwas passieren könnte? Dass Sie- dass Sie da einen schweren Unfall erleiden können? |
| 409 | **RM05:** Ja, ja, ja, hm (zustimmend). |
| 410 | **I2:** Mh-h. Sodass Sie das lieber nicht mehr machen wollen? |
| 411 | **RM05:** Ich würde da- also ich würde tatsächlich, wäre mir wahnsinnig geholfen, wenn ich zur Arbeit mit den öffentlichen Verkehrsmitteln kommen (I2: Mh-h (zustimmend)) könnte (I2: Mh-h, mh-h, mh-h (zustimmend)). Das wäre mir deutlich lieber (I2: Ja.) und ich würde mich wesentlich wohler fühlen. |
| 412 | **I2:** Wöhler fühlen, ja. Das geht nicht, weil das zu weit ist, oder? |
| 413 | **RM05:** Das geht nicht. Weil das würde zwei Stunden mit den ganzen Dorfbussen dauern, bis ich von [ANONYMISIERT, ORT IN DEUTSCHLAND] bis nach [ANONYMISIERT, ORT IN DEUTSCHLAND] gekommen bin. |
| 414 | **I2:** Und Sie gewöhnen sich da nicht wieder daran? An das Autofahren? Dass Sie sagen (RM05: Ich wollte jetzt): Jetzt geht es doch eigentlich ganz gut wieder? |
| 415 | **RM05:** Ich wollte jetzt als Maßnahme dagegen nochmal zwei, drei Fahrstunden nehmen (I2: Mh-h, ja.). Um mich da wieder besser daran zu gewöhnen und (I2: wär ja blöd, wenn Sie es-) irgendwann auch mit jemand nebendran (I2: Ja.) zu fahren. |
| 416 | **I2:** Ja. Ja. Ja. Das wär ja blöd, wenn Sie das- |
| 417 | **RM05:** Wenn ich es gar nicht mehr- |
| 418 | **I2:** vor lauter Ängsten das gar nicht mehr machen würden. |
| 419 | **RM05:** Ja, das wär richtig schlecht. |
| 420 | **I2:** Ja. Ja. Ja. Müssen Sie aufpassen, dass Sie das nicht sich abgewöhnen aus lauter unbegründeter Angst, ne? |
| 421 | **RM05:** Ja, genau, wobei ich sagen muss, das ist in letzter Zeit jetzt wieder besser geworden (I2: Hm (zustimmend)) und mein Verhaltenstherapeut sagt immer, dass das äh an einem Stimmungstief liegt. |
| 422 | **I2:** Klar, ja, das kann sehr damit zusammenhängen. |
| 423 | **RM05:** Dass die Ängste an dem- an dem Stimmungstief liegen. |
| 424 | **I2:** Und dann wieder zunehmen. Ja. Ja. |
| 425 | **RM05:** Also aktuell würde ich sagen- ich darf aktuell ja noch kein Auto fahren (I2: Mh-h (zustimmend)), weil ich noch hier bin (I2: auf Station sind, ja, mh-h), aber aktuell würde ich mir, glaube ich, wieder mehr zutrauen. Aber so mit Ängsten (I2: Mh-h (zustimmend)), da kenne ich mich schon ganz gut aus. |
| 426 | **I2:** Mh-h, okay. Ängste auch noch in anderen Sinnen? So- manchmal gibt es auch ganz pff unspezif- also so-so-so grundlose Ängste, die man hat. Die einen irgendwie überkommen. Man weiß gar nicht, wovor man sich ängstigt. Aber auch, da die Welt so, weiß ich, bedrohlich ist. Oder, wenn man- |
| 427 | **RM05:** Ja, also was ich noch habe ist das was ich auch schon [crosstalk] was ich auch schon erwähnt hatte, mit dem E-Mail-Postfach? |
| 428 | **I2:** Ja, das hatten Sie schon, genau. |
| 429 | **RM05:** Dass ich verklagt werden (I2: Mh-h (zustimmend)) könnte (I2: Mh-h, mh-h, mh-h (zustimmend), ja) und Autofahren und ein Stück weit Zugfahren auf unbekannten (I2: Mh-h (zustimmend)) Strecken auch (I2: Mh-h (zustimmend), das, ja.). Wobei Zugfahren deutlich entspannter ist als Autofahren. |
| 430 | **I2:** Aber das sind alles so jedenfalls so relativ benennbare Angst- äh Situationen- (RM05: Ja.) angstauslösende Situationen (RM05: Ja.). So etwas Unbenennbares, freie Angst, die einen so überkommt, ohne dass (RM05: nee) man direkt weiß, warum? |
| 431 | **RM05:** Nee. |
| 432 | **I2:** Vor- vor irgendwas (RM05: Mh (überlegend)) Bedrohlichem, Unbestimmten? |
| 433 | **RM05:** Nee. |
| 434 | **I2:** Das kennen Sie nicht, ja. Haben Sie mal das Gefühl gehabt, irgendwie Sie- Sie- so die Welt, äh so wie durch einen Sch- durch einen Schleier zu sehen? Durch äh durch Watte hindurch die Welt zu erleben oder so dass zwischen Ihnen und der- der Welt irgendwie so ein- ein äh Matte- Watte liegt, oder Schleier oder ein- Sie wie in einem Glashaus sind? |
| 435 | **RM05:** Nee, so etwas kenne ich nicht. |
| 436 | **I2:** Gefühl von durchgreifender äh Vitalitalitsstörungen? Erschöpfungen? Ein Ausgebranntsein? Nicht- keine Energie mehr haben? |
| 437 | **RM05:** Ja, definitiv. |
| 438 | **I2:** Das kennen Sie schon? |
| 439 | **RM05:** Ja. |
| 440 | **I2:** Auch- damit meine ich jetzt auch weniger die Zeit jetzt in oder nach der Psychose. Da hat man so etwas häufiger (RM05: Mh (zustimmend), dass man so äh von der Erkrankung erschöpft ist, ne? |
| 441 | **RM05:** Ja. |
| 442 | **I2:** Aber jetzt so, weiß nicht, oder, davor, vor der letzten Erkrankung, dass Sie in einem normalen Zustand immer so das Gefühl haben: Da fehlt so die Kraft? Ich habe nicht mehr so richtig die Initiative, mich zu allem aufzuraffen, so wie früher? |
| 443 | **RM05:** Das kenne ich definitiv. |
| 444 | **I2:** Kennen Sie schon auch? |
| 445 | **RM05:** Ja. |
| 446 | **I2:** Schlimm, ausgeprägt, oder mittel, oder? |
| 447 | **RM05:** Mittel |
| 448 | **I2:** Mittel. |
| 449 | **RM05:** Mittel, also ich kriege es irgendwie (I2: kriegen schon noch etwas hin) hin, die Initiative zu ergreifen (I2: Mh-h, mh-h (zustimmend)). Allerdings ähm habe ich immer- ich war so in der Schulzeit, konnte ich mich nie zu etwas aufraffen (I2: Mh-h, mh-h (zustimmend)). Das war ähm also meine Mutter hat zu den anderen [RÄUSPERT SICH] gesagt: "[ANONYMISIERT, NAME DER INTERVIEWTEN PERSON] macht gerade irgendwie Abitur" (I2: (lacht schnaubend), mh-h). Und ich habe wirklich irgendwie Abitur gemacht, also (I2: Mh-h (zustimmend)) ich habe einen Abschluss von 2,2 gehabt, glaube ich. Also relativ gut, so. |
| 450 | **I2:** Ja, Sie waren ja auch sehr gut, auf (RM05: Ich habe) der (RM05: Ich habe allerdings nichts) Grundschule zumindest, ja. |
| 451 | **RM05:** Ich habe nichts dafür gelernt (I2: Mh-h, mh-h, mh-h (zustimmend)). Also ich habe wirklich- ich habe mich immer einen Tag vorher oder [crosstalk] eine Nacht vorher hingesetzt (I2: Ja.). Habe mir kurz was angeschaut. Und dann habe ich eine zwei oder eine drei geschrieben [crosstalk]. |
| 452 | **I2:** Dann müssen Sie aber auch wirklich gut gewesen sein, dass es dann trotzdem so gut geworden ist, gel? |
| 453 | **RM05:** Ja, dann war ich damit zufrieden. |
| 454 | **I2:** Mh-h, mh-h (zustimmend). Also so lange durchhalten, lange kontinuerlich an etwas dranbleiben, fällt Ihnen schwer? |
| 455 | **RM05:** Fällt mir schwer. |
| 456 | **I2: So** Fleiß, sagt man früher. |
| 457 | **RM05:** Naja nicht fleiß. Ich bin schon fleißig, was manche Dinge angeht (I2: Mh-h (zustimmend)). Also fleißig, wenn mich zum Beispiel etwas extrem interessiert (I2: Okay, ja). Ich- mein aktueller Beruf ex- interessiert mich zum Beispiel extrem und- |
| 458 | **I2:** Ja, was ist das? |
| 459 | **RM05:** Ich mache [ANONYMISIERT: ART DES BERUFS]. |
| 460 | **I2:** Ah. Ah. |
| 461 | **RM05:** Also ich [ANONYMISIERT: ART DES BERUFS] (I2: Mh-h, mh-h (zustimmend)) eines [ANONYMISIERT: ART DES UNTERNEHMENS] darauf hin, dass der besonders gut bei [ANONYMISIERT: NAME EINES UNTERNEHMENS] gefunden wird. |
| 462 | **I2:** Das ist eine wichtige- heute eine wichtige Aufgabe, ja. |
| 463 | **RM05:** Das ist eine wichtige Aufgabe. |
| 464 | **I2:** Okay. Mh-h. Und das macht Ihnen auch Spaß? |
| 465 | **RM05:** Das macht mir auch Spaß. |
| 466 | **I2:**  Oder so, das reizt Sie? |
| 467 | **RM05:** Das reizt mich, (I2: Ja) genau (I2: Ja, ja). Das macht mir großen Spaß (I2: Mh-h (zustimmend)) und in der Schulzeit war das so, dass- also Politik habe ich zum Beispiel extrem gerne gemacht (I2: Mh-h (zustimmend)) und Englisch, da hatte ich dann auch gute Noten. Und dann habe ich auch bisschen mehr die Initiative ergriffen. Aber in den meisten Fächern musste ich schlicht und ergreifend nichts lernen. Oder habe mich dann (I2: beneidenswert) kurz mit was- (I2: beneidenswert, eigentlich ne?) oder habe mich nur kurz mit etwas beschäftigt. Im Studium an der [ANONYMISIERT: ART DER HOCHSCHULE] (I2: Mh-h (zustimmend)) war das schon bisschen schwieriger. Da habe ich mich dann schon paar Tage (I2: Mh-h (zustimmend)) vorher dransetzen müssen (I2: Mh-h, mh-h (zustimmend)). Aber so den richtig großen Aufwand habe ich auch da nicht betrieben (I2: Mh-h (zustimmend)). Und deswegen glaube ich auch, dass meine Erkrankung erst so richtig ähm ausgebrochen ist, als ich arbeiten gehen musste (I2: Mh (zustimmend)), wegen diesem Stress-Vulne-Vulnerabilitäts-Modell. |
| 468 | **I2:** Ja, ja, ich weiß, was Sie meinen, ja. |
| 469 | **RM05:** Und ich habe halt in dem Moment, wo ich arbeiten gehen musste, das erste Mal so richtigen Stress gespürt (I2: Mh-h, mh-h (zustimmend)). Also den andere jetzt vielleicht schon vorm Abitur gehabt hätten (I2: Mh-h (zustimmend), ja) oder zu einem anderen Zeitpunkt (I2: Ja.) und äh bei mir war das halt der Einstieg in die Arbeitswelt. |
| 470 | **I2:** Arbeitswelt. Aha. War das auch von den Betriebsabläufen oder Firmenabläufen her sehr stressig, oder sehr anspruchsvoll? Fordernd? Dass man sehr rasch arbeiten musste, oder? |
| 471 | **RM05:** Es war, ja, ja, ja. (I2: Mh-h (zustimmend)). Es war- also ich habe in einem- in einer [ANONYMISIERT: ART DES UNTERNEHMENS] gearbeitet (I2: Mh-h, mh-h (zustimmend)) und äh mit vielen, vielen Kunden (I2: Mh-h (zustimmend)) und äh Deadline hier, Deadline (I2: Mh-h (zustimmend), okay) da und- |
| 472 | **I2:** Also es war schon objektiv richtig- |
| 473 | **RM05:** Das war (I2: fordernd, stressig) richtig stressig (I2: Ja, ja.). Es war richtig, richtig stressig (I2: Ja.). Und äh ab dem Zeitpunkt habe ich zum ersten Mal auch so richtig gemerkt: [ANONYMISIERT, NAME DER INTERVIEWTEN PERSON], mit dir stimmt irgendetwas nicht. (I2: Mh-h (zustimmend)). Und dann habe ich es noch irgendwie ab dem Zeitpunkt "[ANONYMISIERT, NAME DER INTERVIEWTEN PERSON], mit dir stimmt irgendetwas nicht" (I2: Mh (zustimmend)), habe ich es noch zwei, drei Jahre ausgehalten bis ich dann wirklich- bis die Psychose ausgebrochen ist. (I2: Was) Und bis gar nichts mehr ging. |
| 474 | **I2:** Wo hatten Sie das Gefühl oder in welcher Hinsicht hatten Sie denn das Gefühl: Stimmt etwas nicht mit Ihnen? (RM05: Mh (überlegend)). Mal Stress kennt jeder. |
| 475 | **RM05:** Ja, ja, ja. Nee, ich hatte das Problem mit ähm Beziehungswahn? |
| 476 | **I2:** Beziehungs? |
| 477 | **RM05:** Beziehungswahn? |
| 478 | **I2:** Ah schon damals, ja? |
| 479 | **RM05:** Ja. Also dass ich ständig dachte, andere Leute sprechen über mich (I2: über mich, ja, ha mh-h). Und das ging so weit, dass ich dachte: Wenn ich diesen Job jetzt hier vermassel, wird mein Chef überall schlecht über mich reden und ich werde nie wieder irgendwie einen Job kriegen (I2: Okay, mh-h, mh-h). Also das war- |
| 480 | **I2:** Das ging über längere Zeit? (RM05: Das ging über) Also über zwei, drei Jahre? |
| 481 | **RM05:** Ja. |
| 482 | **I2:** Die ganze Zeit so ein- |
| 483 | **RM05:** Ja, ja, ja. |
| 484 | **I2:** Mh-h. |
| 485 | **RM05:** Und ich habe mir leider Gottes viel zu spät Hilfe gesucht. |
| 486 | **I2:** Ja, ja, ja. Bis es dann richtig zum Ausbruch kam. |
| 487 | **RM05:** Es musste halt so richtig Knall machen (I2: ja, ja, ja.), bis ich eingesehen habe, dass ich jetzt nicht noch mehr leisten muss. |
| 488 | **I2:** Aber Sie waren schon vorher zwei, drei Jahre, würden Sie sagen, unter dem Stress äh (RM05: ja) die anderen beobachten, wie ich da (RM05: ja) jetzt arbeite, wie ich da zurechtkomme (RM05: ja) und wenn ich da jetzt versage, bin ich erledigt, weil mein Chef mich da irgendwie hinhängen wird (RM05: ja) oder so etwas (RM05: Genau.). Ja, ein richtiger Stress. |
| 489 | **RM05:** Ja, richtiger Stress. |
| 490 | **I2:** Mh-h. Das ist jetzt aber nicht mehr so? |
| 491 | **RM05:** Ist (I2: oder) jetzt nicht mehr so, nee. |
| 492 | **I2:** Sie haben ja jetzt noch gearbeitet auch vor der- vor der Aufnahme hier? |
| 493 | **RM05:** Ja, genau. Nee, ist nicht mehr. |
| 494 | **I2:** Das haben Sie nicht mehr? |
| 495 | **RM05:** Habe ich abgelegt. |
| 496 | **I2:** Ja, ja. Aber da vor der erste- ersten Erkrankung war das längere Zeit? |
| 497 | **RM05:** Ja. |
| 498 | **I2:** Ja. Bisschen bräuchten wir noch. Geht es noch? |
| 499 | **RM05:** Ja, klar. |
| 500 | **I2:** Gut. Ähm noch zum Körpererleben, haben wir noch (RM05: mh-h (zustimmend)) gesagt- hatte ich vorher gesagt, wollte ich Sie noch etwas fragen ähm ob Sie da mal Veränderungen bemerkt haben. Damit meint man jetzt, mehr so, was man so spürt im Körper. Ob der sich so halbwegs normal anfühlt. Oder ob man Empfindungen bekommt: Da stimmt etwas nicht. Der fühlt sich ganz komisch an. Da sind Veränderungen. Da sind komische- Empfi- komische Empfindungen. Regungen. Schmerzen, oder was auch immer, was man nicht so recht einordnen kann. Kennen Sie so etwas? Ich meine also nicht die normalen Kopfschmerzen oder (RM05: Mh-h (zustimmend)) Bauchschmerzen oder so (RM05: Ja) etwas, sondern irgendso ein mehr Komisches, Grundlegendes. Veränderungen im Körper. |
| 501 | **RM05:** Eher nicht, nee. |
| 502 | **I2:** Kennen Sie nicht? |
| 503 | **RM05:** Eher nicht, nee. |
| 504 | **I2:** Ja. Sie mal geschaut haben: Auch außerlich hat sich etwas an mir verändert? Sie mal in einen Spiegel öfter geguckt haben oder so etwas? |
| 505 | **RM05:** Das kommt durchaus vor. Weil ich mich regelmäßig begutachte. Weil ich durch die Medikamente so viel zugenommen [LACHT] habe. |
| 506 | **I2:** Okay. Das ist ein nachvollziehbarer Grund, ja (RM05: Ja, ja). Okay gut, äh, Sie schauen so ein bisschen: Wie ist mein Körper (RM05: Wie mein) Umfang (RM05: Körper sich entwickelt). Wie der sich entwickelt, okay (RM05: Ja), gut. Aber dass Sie deswegen in den Spiegel geschaut haben, weil Sie dachten, irgendwas ist mit mir komisch? Oder hat Sie das äh Verändern in meinen (RM05: nee) Gesichtszügen (RM05: nee), ich weiß gar nicht mehr, drückt sich so komisch aus im Spiegel? |
| 507 | **RM05:** Mh-h (verneinend). |
| 508 | **I2:** Bin gar nicht mehr ich? |
| 509 | **RM05:** Nee. |
| 510 | **I2:** Kennen Sie nicht. Dass Ihnen der Körper zu groß, zu klein vorkam. Ich meine jetzt nicht vom (RM05: [LACHT], nee, nee, nicht vom Umfang) Gewicht her, oder vom Umfang, sondern dass Sie so das Gefühl hatten: Da stimmt etwas nicht. Hände sind ganz anders, oder so etwas? |
| 511 | **RM05:** Nee. |
| 512 | **I2:** Kennen Sie gar nicht. Körperfremdheit hatten Sie, glaube ich, schon gesagt. Kennen Sie auch nicht, dass der Körper nicht zu Ihnen gehörte, oder passte, oder nicht richtig stimmte, oder- kennen Sie nicht. Rätselhafte Körperempfindungen: Dass etwas vibriert oder sich wie Strom anfühlt im Körper? Oder dass Sie das Gefühl haben, irgendwas bewegt sich da herum? |
| 513 | **RM05:** Mh-h (verneinend). |
| 514 | **I2:** Kennen Sie alles nicht? |
| 515 | **RM05:** Nee. Also so vom Körperempfinden war bei mir (I2: war bei Ihnen alles normal) alles relativ normal. |
| 516 | **I2:** Nee, ich gehe es auch nur kurz durch, dass Sie (RM05: ja) ähm so das Gefühl hatten, der Körper bewegt sich nicht richtig? Oder- oder zu viel? Plötzlich, wie wenn man sich hochgehoben fühlt? Oder plötzlich runtersackt? |
| 517 | **RM05:** Mh-h (verneinend). |
| 518 | **I2:** Bewegungen mal blockiert sind? Dass man irgendwas wie gelenkt sich erlebt? |
| 519 | **RM05:** Mh-h (verneinend). |
| 520 | **I2:** Kennen Sie auch nicht. Gab es mal Ver- irgendwie das Gefühl, dass andere äh irgendwie mit Ihrem Körper zu tun haben, in dem Sinne, dass sie dasgleiche machen, wie Sie? Also- also, wenn ich jetzt auch genau das machen würde, also dass das so- |
| 521 | **RM05:** Das kenne ich, ja. |
| 522 | **I2:** So, das Gefühl gibt, die- so komisch, der macht jetzt genau dasgleiche. |
| 523 | **RM05:** Ja, (I2: wie ich) das kenne ich. |
| 524 | **I2:** Mh. Vielleicht beschreiben Sie selber ein Beispiel, wie Sie es (RM05: Mh, also ich hatte einmal) erleben? |
| 525 | **RM05:** So eine- Ich war einmal in einem Schuhladen (I2: Mh-h (zustimmend)) und da hat sich jemand Stiefel angezogen (I2: Mh-h (zustimmend)). Und ich habe mir im selben Moment auch Stiefel angezogen, und ich habe mir gedacht, der zieht die Stiefel genauso an wie ich. |
| 526 | **I2:** Mh-h, mh-h (zustimmend). Und das war komisch? |
| 527 | **RM05:** Das war komisch. Das war unangenehm (I2: Mh-h (zustimmend)). Und dann sind auch so ein paar Verfolgungsgedanken dazu gekommen (I2: Mh-h, mh-h (zustimmend)). Und das war unangenehm, auf jeden Fall. |
| 528 | **I2:** Mh-h, also dass sich das so angefühlt hat, wie wenn Ihr Kö- Ihr- Sie jetzt dasgleiche machen mit Ihrem Körper wie der und das irgendwie sich so spiegelt, sozusagen? |
| 529 | **RM05:** Ja, genau. |
| 530 | **I2:** Ja. Oder dass der Sie dadurch irgendwie beeinflussen könnte? Weil Sie sagten, Verfolgung kam dann dazu. |
| 531 | **RM05:** Ja, genau. |
| 532 | **I2:** Mh-h, mh-h. Kam das auch mal vor, dass Sie das Gefühl hatten, da löst sich so ein bisschen etwas auf wegen- so eine Abgrenzung zwischen mir und dem anderen? Dass das ineinander übergeht? |
| 533 | **RM05:** Nee, so etwas nicht. |
| 534 | **I2:** Das nicht. So weit nicht, dass Sie da das Gefühl hatten, irgendwas geht- Gegenstände oder andere Körper, die werden sozusagen so- dehnen sich so aus und fließen in Sie hinein oder so etwas Ähnliches? Hört sich ein bisschen Science Fiction an, aber (RM05: (lacht schnaufend)) man- das gibt solche Erlebnisse, ja. |
| 535 | **RM05:** So etwas kenne ich nicht. |
| 536 | **I2:** Ja. Also die Grenze sich auflöst, so dass man so verschmilzt mit anderen. Das nicht. Aber das, was Sie beschreiben, dass man so auffällig äh bemerkt, das ist irgendwie so ähnlich, was (RM05: ja, ja, ja) der jetzt macht, und was ich mache (RM05: das kenne ich, ja). Dass- dass einem das auffällt und (RM05: ja) und ein bisschen beunruhigt. |
| 537 | **RM05:** Ja, das kenne ich. |
| 538 | **I2:** Ähm. Das Gefühl, dass- dass so etwas- dass Ihnen da irgendwas selbst- von sich selbst verloren geht? Dass Sie da von einem anderen über- überwältigt werden? Über- dass der- dass der in Sie hineinkommt? Oder sich selbst verlieren dabei? So dramatisch jetzt auch nicht? |
| 539 | **RM05:** Nee. |
| 540 | **I2:** Letzter Bereich wäre noch so ein bisschen, äh ob Sie besondere ähm ja, Erlebnisse, Erfahrungen oder Gedanken haben zu ähm dem, was man so philosophische Themen nennen könnte, oder religiöse Themen, die manchmal so einen aufwühlen können. Also ähm äh wie ein Gefühl, dass es irgendwas ganz besonders Bedeutsames mit Ihnen passiert. Dass Sie irgendwie zu einer besonderen äh Wahrnehmung befähigt sind? Die andere so nicht haben? Dass Sie das Gefühl haben, da ist irgendwas grundsätzlich äh mit der Welt- dass mir zugänglich ist, aber einem anderen nicht. So etwas Ähnliches (RM05: Mh-h (zustimmend)). Und dann da so ein bisschen ins Nachdenken geraten? Oder mit philosophischen Fragen beschäftigen, die in die Richtung gehen. |
| 541 | **RM05:** Mh (überlegend). So extrem würde ich es nicht sagen (I2: Mh-h (zustimmend)). Aber ich hatte, als ich noch ähm in [ANONYMISIERT: ORT IN DEUTSCHLAND] gearbeitet habe, in den [ANONYMISIERT: ART EINES UNTERNEHMENS], also ich habe in zwei [ANONYMISIERT: ART EINES UNTERNEHMENS], insgesamt gearbeitet (I2: Mh-h (zustimmend)), hatte ich so den Gedanken, dass ich der Auserwählte bin (I2: Mh-h (zustimmend)), der diese [ANONYMISIERT: ART EINES UNTERNEHMENS], retten muss und auf den richtigen Weg führen muss (I2: Mh-h (zustimmend)). Und habe dann quasi ähm habe- wollte Abläufe ändern, mit denen ich überhaupt nichts zu tun hatte. Oder habe dem reingequasselt, und habe dem reingequasselt (I2: Mh-h (zustimmend)) und habe mich um Dinge gekümmert, die eigentlich überhaupt nicht meine Aufgabe waren (I2: Mh-h, mh-h (zustimmend)), die aber trotzdem- |
| 542 | **I2:** Haben sich nicht immer beliebt gemacht, dabei? [LACHT] |
| 543 | **RM05:** Habe mich nicht immer beliebt gemacht dabei, nee. |
| 544 | **I2:** Mh-h. Aber trotzdem, was, ich habe [crosstalk]. |
| 545 | **RM05:** Aber ja, genau, ich habe mich halt wirklich als der Auserkorene (I2: Mh-h (zustimmend)) gefühlt in dem Moment. |
| 546 | **I2:** Mh-h (zustimmend). Von- kann man sagen, von wem oder von was auserkoren? Irgendwie vom (RM05: Mh (überlegend) Schicksal? Von der- |
| 547 | **RM05:** Vom Schicksal, definitiv. |
| 548 | **I2:** Nicht von Gott jetzt, oder? |
| 549 | **RM05:** Nicht von Gott, (I2: nein) nee (I2: Mh (zustimmend)), so gläubig bin ich nicht. |
| 550 | **I2:** Sind Sie nicht. Aber vom Schicksal, irgendwie so etwas: Das- das ist jetzt meine Berufung. |
| 551 | **RM05:** Genau (I2: Mh-h (zustimmend)), das war meine Berufung, diese [ANONYMISIERT: ART EINES UNTERNEHMENS], zu retten. |
| 552 | **I2:** Mh-h, mh-h, und die waren so am Abdriften, oder? |
| 553 | **RM05:** Äh die zweite war ziemlich am Abdriften (I2: Mh-h (zustimmend)). Also haben- reihenweise sind die Kunden abgesprungen (I2: Mh-h (zustimmend)) ähm und es gab jemanden in leitender Funktion, der keinen guten Job gemacht hat (I2: Mh-h, mh-h (zustimmend)). Und ich habe da mal zwei Monate darauf hingewiesen, dass da einiges schiefläuft (I2: Mh-h, mh-h (zustimmend)). Das hat leider sehr lange gedauert, bis man mir geglaubt, aber ähm- |
| 554 | **I2:** Aber Sie hatten recht? |
| 555 | **RM05:** Ich hatte recht. |
| 556 | **I2:** Mh-h, mh-h. Gut, dann hatten Sie ja nicht nur ein besonderes Auserwähltheitsgefühl, sondern eben auch einen unwahrscheinlich guten Blick für das, was da schiefläuft. |
| 557 | **RM05:** Ja (I2: Mh-h (zustimmend)), genau. |
| 558 | **I2:** Mh-h. Aber es kam so ein bisschen hinzu, so ein leichtes Größengefühl, so? |
| 559 | **RM05:** Es war def- (I2: Ich bin der) das war definitiv größenwahnsinnig (I2: Ich bin der Retter. Größenwahnsinnig ein bisschen). Das war größenwahnsinnig (I2: Mh-h (zustimmend), ja), was ich da alles gemacht habe (I2: Mh-h (zustimmend)), das ist unglaublich. Ich frage [crosstalk]. |
| 560 | **I2:** Das war auch jetzt nicht in der Psychose, ne? Also nicht in diesen- |
| 561 | **RM05:** Das war vor der Psychose. |
| 562 | **I2:** War vorher, ah ja. Da kamen Sie in so in so einen Höhen- (RM05: Ja.) Flug? |
| 563 | **RM05:** Ja, da war definitiv so ein Höhenflug (I2: Mh-h (zustimmend)) vorhanden (I2: Mh-h, mh-h (zustimmend)). Und ich habe auch immer wieder gefragt: Wieso habt ihr mich nicht gekündigt? Wieso habt ich mich nicht gekündigt (I2: Mh-h, mh-h (zustimmend))? Und habe bis heute noch keine vernünftige Antwort darauf bekommen, aber es ist mir ein Rätsel. |
| 564 | **I2:** Vielleicht waren Sie ja trotzdem gut? |
| 565 | **RM05:** Vielleicht war ich trotzdem gut, das glaube ich mittlerweile auch. |
| 566 | **I2:** [LACHT] Dass man Sie nicht gerne äh gehen hätte lassen, ja. Aber da war etwas verschoben? |
| 567 | **RM05:** Ja, definitv. |
| 568 | **I2:** Ja. Äh aber das war jetzt wieder mehr eine Phase jetzt, aber sonst mh so kommt so etwas nicht vor? |
| 569 | **RM05:** In letzter Zeit [crosstalk, I2 gleichzeitig: in letzter Zeit zumindest] ist so etwas nicht vorgekommen, nee. |
| 570 | **I2:** Mh-h. Und dieses Gefühl, eine besondere Einsicht, besondere äh Zugang zu besonderen Realitäten zu haben? Zu verborgenen Geheimnissen der Welt oder so etwas Ähnliches? Also kennen Sie sonst nicht? |
| 571 | **RM05:** Nee. |
| 572 | **I2:** Ähm kam es mal vor, dass Sie die Welt überhaupt äh ja ganz anders gesehen haben als andere? Zum Beispiel, dass alles nur noch für eine große Illusion gehalten haben? Oder- |
| 573 | **RM05:** Nee, so extrem nicht. |
| 574 | **I2:** Sie sich mit besonderen mystischen Gedanken oder geheimen Lehren beschäftigt haben? |
| 575 | **RM05:** Mh-h (verneinend). |
| 576 | **I2:** Auch das nicht. Dass Sie die Welt besser verstehen? Naja, das sagten Sie schon. In dieser einen Situation, ja. |
| 577 | **RM05:** Ja, da habe ich die Welt besser verstanden (I2: besser verstanden) als jeder andere. |
| 578 | **I2:** Gut, okay [LACHT]. |
| 579 | **RM05:** Ich habe mich auch mit meinen Mitbewohnern gestritten um wirklich die banalsten Themen (I2: Mh-h (zustimmend)) und habe eine Riesendiskussion wegen allem angefangen (I2: Mh-h (zustimmend)). Also ich war wirklich nicht angenehm in der Zeit (I2: Mh-h (zustimmend), ja). Muss ich rückblickend sagen. |
| 580 | **I2:** Ja. Sie so manchmal das Gefühl- auch das hört sich jetzt komisch an, dass äh die- nur das da ist, was Sie gerade sehen? Dass die Welt nur für Sie da ist, sozusagen? Also: alle- alless hängt von mir ab. Äh nur d- nur das exisitiert, was ich gerade wahnehme? Kennen Sie nicht? |
| 581 | **RM05:** Nee, kenne ich nicht. |
| 582 | **I2:** Ja. Okay. Beziehungswahn hatten Sie selber schon beschrieben. So ein bisschen äh in dieser Arbeitssituation. Aber dass sonst Sie äh in der Stadt herumlaufen und Sie das Gefühl haben: Alles das, was gerade jetzt passiert, passiert nur für mich? Oder hat nur mit mir zu tun? |
| 583 | **RM05:** Mh so Gedanken habe ich hin und wieder noch (I2: Mh-h, mh-h (zustimmend)). Ich versuche die gerade mit Verhaltenstherapie ein Stück weit abzustellen. Aber (I2: Mh-h (zustimmend)) Sie müssen sich vorstellen: Ich war ja ähm also ich war erst kurz in der Psychiatrie in [ANONYMISIERT, ORT IN DEUTSCHLAND] (I2: Mh-h (zustimmend)) und anschließend war ich in [ANONYMISIERT, ORT IN DEUTSCHLAND] auf der Geschlossenen (I2: Mh-h (zustimmend)) und da gab es diesen Sozialarbeiter (I2: Mh-h (zustimmend)) und dieser Sozialarbeiter ist jetzt gerade von der- von [ANONYMISIERT, ORT IN DEUTSCHLAND] hierher gewechselt (I2: Mh-h, mh-h (zustimmend)). Und ich dachte, das ist alles nur wegen mir passiert (I2: mir, das kommt Ihnen) für einen Moment (I2: falsch vor, ja, ja. mh). Und ich dachte mir noch: Oh Gott, ich werde verfolgt. |
| 584 | **I2:** Mh-h, mh-h (zustimmend). Also das ist schnell da, so ein (RM05: So ein Gedanke) Gefühl, so ein Gedanke, das ist doch nicht zufällig? |
| 585 | **RM05:** Ja, genau. |
| 586 | **I2:** Hat doch was auf sich? |
| 587 | **RM05:** Genau. |
| 588 | **I2:** Mh-h, mh-h (zustimmend). Also da müssen Sie immer noch so ein bisschen aktiv an Gedanken- |
| 589 | **RM05:** Muss ich aktiv gegensteuern, (I2: Mh-h (zustimmend)) ja. Das ist wohl auch durch die Medikamente nicht ganz wegzukriegen. |
| 590 | **I2:** Ja, ja. Aber man kann da selber auch ein Stück- |
| 591 | **RM05:** Ja, man kann das selbst selbst viel machen, ja. |
| 592 | **I2:** Mh. Aber die Medikamte und so schützen das in der Regel so? |
| 593 | **RM05:** Ja, ja. |
| 594 | **I2:** Dass es nicht mehr ganz so ?(verpannt)? einem vorkommt, ja? |
| 595 | **RM05:** Ja. |
| 596 | **I2:** Mh-h. Ja. Okay. Ja. Wann würden Sie denn sagen, hat denn so eine- so Veränderungen bei Ihnen begonnen? Die jetzt- also nicht die Psychose, entweder die akute, sondern Sie sagten schon, drei Jahre vorher, zwei, drei Jahre vorher waren (RM05: ja) so Bedrohungsgefühle da. Das Gefühl, so ein bisschen anders zu sein? In Tagträumen zu leben? Geht das schon länger zurück (RM05: Mh (überlegend))? Ab wann, würden Sie sagen, hat so ein bisschen so etwas sich verändert in Ihrem Leben? Schon mit zehn, oder ab 15, oder 20? |
| 597 | **RM05:** Ist schwer zu sagen (I2: Schwer zu sagen, ja). Ich habe mir- ich habe mir schon wirklich lange Gedanken darüber gemacht (I2: Mh-h (zustimmend)). Und komme immer nicht auf den grünen Zweig mit (I2: Mh-h (zustimmend)) einer Antwort die mich irgendwie zufriedenstellt (I2: Mh-h, mh-h (zustimmend)). Ähm aber ich habe auch mit meiner Mutter sehr viel darüber gesprochen (I2: Mh-h, mh-h (zustimmend)) und ähm die meinte eigentlich, dass das schon äh in der Grundschule angefangen hat (I2: Mh-h (zustimmend)). Und zwar hat eine Lehrerin über mich geschrieben: [ANONYMISIERT, NAME DER INTERVIEWTEN PERSON] kann sehr vieles, traut sich aber sehr wenig zu (I2: Mh-h, mh-h (zustimmend)). Und das beschreibt mich äh (I2: Mh-h (zustimmend)) sehr gut bis- (I2: Mh-h (zustimmend)) bis heute also (I2: Mh-h (zustimmend)) ich denke immer, dass das, was ich mache, nicht gut genug ist (I2: Mh-h (zustimmend)) aus irgendeinem Grund. |
| 598 | **I2:** Mh-h, mh-h. So Selbstzweifel? |
| 599 | **RM05:** Selbstzweifel. |
| 600 | **I2:** Liegen nahe bei Ihnen? |
| 601 | **RM05:** Ja, liegen nah bei mir (I2: Mh-h (zustimmend)). Und das ist glaube ich schon so ein Stück weit- ja, das ist- das war so der Anfang von allem. |
| 602 | **I2:** Mh-h, mh-h, mh-h. Dass Sie sich nicht so Ihrer selbst ganz sicher sind? |
| 603 | **RM05:** Ja, genau. |
| 604 | **I2:** Mh-h. Mh-h. Aber das sind vielleicht auch mehr äh Zweifel, ja wie gut bin ich? Wie gut schaffe ich etwas? Wie komme ich an? Aber es ging nicht so in diesen, das habe ich ja schon gefragt, so in diese Ebene: Ja, bin ich überhaupt da? Wer bin ich überhaupt? |
| 605 | **RM05:** Nee. |
| 606 | **I2:** So etwas nicht. |
| 607 | **RM05:** Nee. |
| 608 | **I2:** Grundsätzlichere, ja, Selbstabgründigkeit, so eine Ungefestigtkeit, oder so etwas. Wo Sie gar nicht wussten (RM05: Nee): Bin ich da überhaupt? Ja? So weit ging es nicht? Mh-h (zustimmend). Ich glaube, wir sind so langsam am Schluss. Haben Sie denn selber noch irgendeinen- einen Punkt, da könnte man noch etwas ergänzen zu solchen besonderen Erfahrungen oder Erlebnissen, die so ich so ein bisschen durchgegangen bin? Wie gesagt, wir müssen jetzt nicht über die Psychose im Detail sprechen (RM05: Ja, ja, ja), aber sonst so das Wichtigste? |
| 609 | **RM05:** Wir haben, glaube ich, das Wichtigste (I2: behandelt, ja?) behandelt, ja. |
| 610 | **I2:** Was Sie noch ergänzen wollen? Okay. War es unangenehm? |
| 611 | **RM05:** Mh, nö. War okay. |
| 612 | **I2:** War okay, gut. Vielen Dank, dass Sie mitgemacht haben. |
| 613 | **RM05:** Ja, gerne |
| 614 | **I2:** Ähm. Es bezieht sich ja auch auf ein bisschen auf die Studien insgesamt, oder, haben Sie da mit- |
| 615 | **I:** Wir haben schon einen anderen Termin (I2 gleichzeit: Schon darüber gesprochen, ja.) Genau. Ich mache jetzt mal hier das wieder aus. |
| 616 | **I2:** Ja, wir sind ja am Ende. |
| 617 | **I:** Genau. |

RM05_EASE_anonymized_2

| 1 | RM05 Fortsetzung |
| --- | --- |
| 2 | **I:** Okay. Also. Wollen Sie erstmal sagen, was Sie- was Ihnen zu Gedankenlautwerden noch eingefallen ist? |
| 3 | **RM05:** Dass ich das schon mal hatte. Dass das (I: Okay.) beschrieben wurde, und dass ich das auf jeden Fall schon mal hatte. (I: Und-) Dass meine Gedanken so laut geworden sind, dass ich das Gefühl, dass ich Sie selbst hören kann. |
| 4 | **I:** Okay. Und andere sie andere gegebenfalls auch hören könnten? |
| 5 | **RM05:** Genau, allerdings (I: Ja.) in der Psychose. |
| 6 | **I:** Ja, alles klar. Davor, danach? |
| 7 | **RM05:** Davor, danach nicht, (I: nie?) nee. |
| 8 | **I:** Und ähm wollen Sie sagen, was für Gedanken das waren? Oder? |
| 9 | **RM05:** Möchte ich lieber nicht sagen. |
| 10 | **I:** Nicht sagen, alles klar. Also Gedanken, für die man sich auch schämen könnte? |
| 11 | **RM05:** Ja. |
| 12 | **I:** Sozuagen. Aber waren das Stimmen, die Sie auch- sozusagen, die über Sie selbst gesprochen haben? |
| 13 | **RM05:** Ja, genau (I: Okay.). Kommentierend. |
| 14 | **I:** Okay, alles klar. Gut. Aber gut, dass Ihnen das noch eingefallen ist. Da hatten Sie "Nein" gesagt, ne? |
| 15 | **RM05:** Da hatte ich nein gesagt (I: Ja.) im Interview, genau (I: Ja.). Und da dachte ich mir, ich muss das nochmal- (I: berichtigen) ich weiß nicht, wieso. Ich habe irgendwie im Affekt- |
| 16 | **I:** Ist ja auch okay. |
| 17 | **RM05:** Ja. Okay. |
| 18 | **I:** Und es geht ja- es geht ja auch nicht unbedingt nur um die Psychose. Vielleicht ging es auch- hatten Sie vorher über die Zeit davor oder danach nachgedacht (RM05: Hm (zustimmend) und deswegen (RM05: Ja) "nein" gesagt. Ist ja auch in Ordnung. |
| 19 | **RM05:** Genau, ja. |
| 20 | **I:** Ähm. Und dieses Schnellerwerden, das hatte Herr Fuchs auch gefragt, dieses Gedankenrasen, dass sie schneller werden und ähm nicht mehr zu fassen sind und so. Hatten Sie das? Hatten Sie dazu- hatten Sie auch nein gesagt? |
| 21 | **RM05:** Dazu hatte ich, glaube ich, nein gesagt. |
| 22 | **I:** Ja (RM05: Ja). Das ist ähm auch nicht- das ist nach wie vor nicht so? |
| 23 | **RM05:** Nee. |
| 24 | **I:** Okay. Alles klar. |
| 25 | **RM05:** Genau. Und jetzt wollten wir noch über Bewegung sprechen. |
| 26 | **I:** Genau. Über Bewegung, richtig. Ähm Sie sagten in dem Interview, dass beim Körper alles in Ordnung wäre und Ihnen da nie irgendwas aufgefallen ist, außer (RM05: Mh-h (zustimmend)) diese ähm dieses Erlebnis, dass Sie- dass jemand anders dasgleiche gemacht hat, wie Sie, beim Schuhebinden? |
| 27 | **RM05:** Ja, genau. |
| 28 | **I:** Genau (RM05: Genau). Und ähm ich wollte jetzt nochmal spezifisch zu Bewegungen fragen, weil (RM05: Mh-h (zustimmend)) wir das auch nochmal erfassen, ne? |
| 29 | **RM05:** Ja. |
| 30 | **I:** Ähm und dann gucken können, wie die Bewegung- die eigene Wahrnehmung der Bewegung mit der Bewegung, die wir messen (RM05: Mh-h (zustimmend)) zusammenhängen könnte (RM05: Mh-h (zustimmend)). Ähm grundsätzlich, Ihre- so wenn Sie jetzt über Ihre ganze gesamte Lebensspanne und auch vor und nach der Psychose nachdenken, wie nehmen Sie Ihre eigenen Bewegungen wahr? Gab es da Veränderungen? Gibt es da Veränderungen? Oder Störungen? Oder Dinge, die Sie stören? |
| 31 | **RM05:** Mh also ich habe noch das Problem, das ist allerdings körperlich bedingt, dass ich eine bisschen schiefe Körperhaltung habe, weil ich mir mal die Schulter gebrochen habe (I: Okay.). Und ich immer wieder in diese Schutzhaltung zurückgehe (I: Ja.), obwohl ich die Schutzhaltung eigentlich nicht mehr benötige. |
| 32 | **I:** Okay. Das war ein Unfall? Oder? |
| 33 | **RM05:** Das war ein Unfall, genau. Ich bin ausgerutscht. Habe mir im Tuberculum majus, also im Kugelgelenk von der Schulter (I: Ja.) ist mir etwas abgesplittert (I: Ah) und ich nehme diese Schu- diese Schutzhaltung nehme ich immer ein (I: Ja.), automatisiert, obwohl ich sie überhaupt nicht mehr benötige (I: Okay.). Also das ist etwas, das mir- |
| 34 | **I:** Wann war dieser Unfall? Wann ist das passiert? |
| 35 | **RM05:** Oh Gott, vor zehn Jahren ungefähr. |
| 36 | **I:** Okay. Das hat gar nichts mit der Erkrankung zu tun? |
| 37 | **RM05:** Nee, hat gar nichts (I: völlig unabhängig) mit der Erkrankung zu tun. |
| 38 | **I:** Okay. Ähm und jetzt ist es wieder alles völlig geheilt? |
| 39 | **RM05:** Es ist alles geheilt (I gleichzeitig: Und sie können Sport machen und alles?), genau, ja. |
| 40 | **I:** Aber das ist so eine- |
| 41 | **RM05:** Die Schutzhaltung ist nach wie vor (I: Ja.) da, dass ich die Schulter so nach oben ziehe. |
| 42 | **I:** Das passiert völlig unbewusst? |
| 43 | **RM05:** Das passiert völlig unbewusst (I: Mh-h (zustimmend)), ja. |
| 44 | **I:** Okay. Also Sie denken sich jetzt nicht ähm irgendwas äh: Ich muss mit meiner Schulter vorsichtig (RM05: nee) umgehen? Oder so? |
| 45 | **RM05:** Nee. |
| 46 | **I:** Okay. Ähm. Ansonsten nichts anderes? |
| 47 | **RM05:** Nichts anderes. Also was mir explizit aufgefallen war: Meine Bewegungen sind schon immer, habe ich den Eindruck, etwas langsamer als die von anderen Menschen (I: Okay.). Also ich bewege mich sehr gemächlich. |
| 48 | **I:** Ja? Aber das ist schon immer so gewesen? |
| 49 | **RM05:** Das ist schon immer so, meiner Meinung nach, ja. |
| 50 | **I:** Also seit Sie über Ihre eigenen Bewegungen nachdenken? |
| 51 | **RM05:** Ja, genau. |
| 52 | **I:** Auch schon als Kind? |
| 53 | **RM05:** Auch schon als Kind, ja. |
| 54 | **I:** Ja. Und wo fällt Ihnen das auf, zum Beispiel? |
| 55 | **RM05:** Ich wurde schon ein paar Mal darauf angesprochen (I: Ach ja, von). Deswegen ist mir das- |
| 56 | **I:** Freunden? |
| 57 | **RM05:** Von Arbeitskollegen, so: Ah, der schleicht halt wieder durch den Gang und (I: Ah (lacht)) so. |
| 58 | **I:** Alles klar. Okay. Aber Ihnen selber ist das dann- also Sie wurden von außen hingewiesen, aber Sie selber haben das nicht bemerkt? |
| 59 | **RM05:** Ich habe es nicht bemerkt. Mir ist es erst aufgefallen, als ich darauf hingewiesen wurde. |
| 60 | **I:** Okay. Okay. Und seitdem achten Sie eher darauf und (RM05: genau) und dann fällt es Ihnen manchmal auf? |
| 61 | **RM05:** Dann fällt es mir manchmal auf, ja. |
| 62 | **I:** Wenn Sie vielleicht zum Beispiel auf der Straße oder so? |
| 63 | **RM05:** Ja, genau. |
| 64 | **I:** Okay. Okay. Okay. Und gab es schon mal das Gefühl, die Erfahrung, dass sich ähm Körperteile oder der Körper an sich bewegt hat, ohne dass Sie das eigentlich wollten? |
| 65 | **RM05:** Nee. |
| 66 | **I:** Nee? |
| 67 | **RM05:** Nee. |
| 68 | **I:** Und ähm oder- oder dass andere Personen- dass Sie das Gefühl hatten, der Körper bewegt sich, ohne dass andere Personen das sehen können? |
| 69 | **RM05:** Nee. |
| 70 | **I:** Nicht. Alles klar. Und ich gehe jetzt die Sachen (RM05: Mh-h (zustimmend)) nochmal durch, so, die wir vergessen haben das letzte (RM05: ja) Mal. Ähm. Oder ist es schon mal vorgekommen, dass Sie Bewegungen ausgeführt haben, ausführen mussten, obwohl Sie das gar nicht wollten? Dass irgendetwas Sie dazu gebracht hat, diese Bewegungen auszuführen? |
| 71 | **RM05:** Mh nicht, dass ich wüsste, nein. |
| 72 | **I:** Okay. Oder dass Sie eine Bewegung ausführen wollten und es hat nicht- es ging nicht, es war wie so blockiert? |
| 73 | **RM05:** Mh wenn ich mal einen Muskelkater hatte (RM05 und I: (lachen)) vielleicht. |
| 74 | **I:** Alles klar (RM05: Aber-). Aber nicht jetzt, dass es öfters vorgekommen ist, oder- oder ähm zum Beispiel vor der Psychose intensiver geworden ist? |
| 75 | **RM05:** Mh (überlegend), wäre mir nichts aufgefallen (I: alles klar), nee. |
| 76 | **I:** Oder dieses Gefühl, manchmal wie gelähmt zu sein? Und dass man sich dann tatsächlich auch nicht mehr bewegen kann? |
| 77 | **RM05:** Mh-h (verneinend). |
| 78 | **I:** Nee. Und als allerletzte Frage: Ähm mit alltäglichen Bewegungen, so etwas- alltägliche Sachen. Gehen, Zähneputzen, Fahrradfahren, Mantelöffnen oder Zumachen (RM05: Mh-h (zustimmend)) und so weiter. Ähm so Sachen, die eigentlich automatisch und nebenbei ablaufen. Gab es da mal Veränderungen, dass das vielleicht zum Beispiel plötzlich nicht mehr einfach so automatisch ging? Sondern, dass Sie irgendwie besonders darauf achten mussten, das zu tun? |
| 79 | **RM05:** Das hatte ich mal eine Zeit lang nach der Psychose beim Schuhebinden (I: Ah ja. Wollen Sie-). Dass ich dann quasi nachdenken- das ist ja so etwas, das macht man völlig automatisch (I: genau). Und da habe ich manchmal dabei ertappt, dass ich nachdenken musste, wie der Knoten jetzt zu machen ist. |
| 80 | **I:** Alles klar. Nur beim Schuhebinden oder auch bei anderen? |
| 81 | **RM05:** Beim Schuhebinden speziell wäre es mir jetzt aufgefallen. |
| 82 | **I:** Und ähm da- da eine längere Zeit? Oder nur ein, zwei Mal? |
| 83 | **RM05:** Pff, weiß ich gerade nicht, ehrlich gesagt. Ich würde sagen, schon öfter, als ein, zwei Mal. |
| 84 | **I:** Okay. Also so, dass es Ihnen auch wirklich mal öfter aufgefallen ist? |
| 85 | **RM05:** Ja, genau. |
| 86 | **I:** Okay. |
| 87 | **RM05:** Dass ich mir gedacht habe: Okay, jetzt musst du irgendwie nachdenken, obwohl du gar nicht müsstest eigentlich. |
| 88 | **I:** Ja. Ja. So eine automatische Handlung war plötzlich nicht mehr automatisch? |
| 89 | **RM05:** Ja, genau. |
| 90 | **I:** Okay. Genau. Gut, danke. Das war es schon zu den Bewegungen. Jetzt habe ich noch eine letzte Frage. Weil wir uns auch gedacht haben, dass wir gerne ähm alle Leute als Experten sozusagen einmal die Forschungsfrage sagen würden (RM05: Mh-h (zustimmend)). Dass Sie sozusagen sagen können aus Ihrer Erfahrung als Experte für die Erkrankung, wie würde ich die Forschungsfrage beantworten. Ja? |
| 91 | **RM05:** Okay. |
| 92 | **I:** Also ich sage Ihnen jetzt einfach mal, was unsere Forschungsfrage ist (RM05: Mh-h (zustimmend)). Und zwar geht es, habe ich schon grob erzählt, geht es darum: Wirkt sich die Schizophrenie auf den Körper aus (RM05: Mh-h (zustimmend)) und auf die Bewegung des Körpers? Und wenn ja, wie? |
| 93 | **RM05:** Ja. |
| 94 | **I:** Also vielleicht wollen Sie erstmal kurz dazu? |
| 95 | **RM05:** Ähm also ich denke, dass sich die Schizophrenie definitiv auf die Bewegungen des Körpers auswirkt, weil man durch die Negativsymptomatik, diese Antriebslosigkeit, automatisch in den Bewegungen auch etwas gehemmter ist (I: Mh-h (zustimmend)) als vielleicht ein gesunder Mensch. |
| 96 | **I:** Ja. Ja. Okay. Und aus Ihrer ganz persönlichen Erfahrung ähm, haben Sie da noch etwas hinzufügen? |
| 97 | **RM05:** Aus meiner persönlichen Erfahrung muss ich halt sagen, dass äh, wie Sie schon mal erwähnt hatten, das mit den Automatismen ist mir (I: Ja.) aufgefallen (I: Ja.), besonders. Und mir ist aufgefallen, dass ich teilweise dachte, dass andere Menschen Dinge genauso tun wie ich (I: Ja.). Was ein Stück weit, glaube ich, auch ein Beziehungswahn (I: Ja.) ist (I: Ja.). Aber auch mit Bewegung zu tun hat. |
| 98 | **I:** Ja, genau. Da haben wir darüber gesprochen, super, danke. (RM05: ja). Genau. Ähm weil die Idee, die Hypothese, die dem Ganzen dahinter steht, ist, dass Schizophrenie von einer fehlenden Verkörperung herrührt oder damit etwas zu tun hat (RM05: Mh-h (zustimmend)). Dass die Selbstwahrnehmung plötzlich nicht mehr so verkörpert ist wie vorher (RM05: Okay.). Und ähm das sozusagen äh versuchen wir zu sehen oder herauszufinden und zu gucken, ob das stimmt (RM05: Mh-h (zustimmend)). Und ähm, was halten Sie davon? Ist meine letzte Frage. |
| 99 | **RM05:** Äh ich (I: Macht das Sinn?) finde den- finde den Ansatz sehr interessant (I: Mh-h (zustimmend)). Und Schizophrenie ist einfach etwas, das sehr wenig erforscht wurde (I: Ja.) bisher. Und ich denke, dass man wirklich alle Aspekte, die irgendwie möglich sind, beleuchten sollte, und dass es deswegen sehr sinnvoller Ansatz ist. |
| 100 | **I:** Okay. Schön. Danke. Das war es schon. Cool. Dann, wie viel Uhr haben wir jetzt? 49? |
| 101 | **RM05:** Na, bitte. |
| 102 | **I:** Die Zeit gut überbrückt. |
| 103 | **RM05:** Ja. |
| 104 | **I:** Mache ich hier mal aus. |

AB04_EASE_anonymized

| 1 | Interview AB04 |
| --- | --- |
| 2 | **I:** Start. Und es ist in Ordnung, dass wir Sie aufzeichnen, oder? |
| 3 | **AB04:** Ja. |
| 4 | **I:** Gut. Dankeschön. Okay. Dann würde ich gerne mit der allerersten Frage anfangen. Und zwar ist die, wenn ich Sie frage: Wer sind Sie. Was würden Sie dann sagen? |
| 5 | **AB04:** Ich bin ähm ein Mensch. (I: Ja.) [LACHT] Ja. |
| 6 | **I:** Ein Mensch. Und wie würden Sie diesen Menschen beschreiben? |
| 7 | **AB04:** Hm. Das ist ja wirklich eine schwere Frage. |
| 8 | **I:** Ist eine schwere Frage, ja. (AB04: Ja.) Aber Sie können alles sagen, was Ihnen einfällt. |
| 9 | **AB04:** Hm. Wie würde ich den Menschen- mich, mich als Menschen sehen? (I: Genau, genau.) Naja. Ich bin manchmal ein bisschen faul, aber auch manchmal auch sehr ehrgeizig. (I: Hm (bestätigend)) Hm. Ja. |
| 10 | **I:** Okay. Und wie sind Sie aufgewachsen? |
| 11 | **AB04:** Äh in normalen Verhältnissen. |
| 12 | **I:** Was sind denn, was sind normale Verh- |
| 13 | **AB04:** Also meine Familie, alle haben Jobs, alle haben- führen ein normales äh Durchschnittsleben. Ja, ich bin ein bisschen abgedriftet (I: Okay.) Ja. Hm, aber ansonsten- ich bin gut aufgewachsen, eigentlich. (I: Wo sind-) Ja. Ich bin ähm bis zu, bis zu meinem 14. Lebensjahr bei meiner Mom gelebt (I: Hm (zustimmend)). Und dann ähm und als- als ich sechs war, haben meine Eltern sich getrennt (I: Okay.) Und mit meinem 14. Lebensjahr bin ich von meiner Mom zu meinem Vater gezogen dann (I: Okay). Bis ich 17 war (I: Okay). Und dann bin ich ähm raus von zu Hause. |
| 14 | **I:** Okay. Und wo war das jeweils? Wo ist Ihre Mutter? Wo ist- |
| 15 | **AB04:** Ähm also meine ganze Familie lebt in [ANONYMISIERT, ORT IN DEUTSCHLAND] (I: Hm (zustimmend)). Und ähm als ne- als ich sechs war, als die Trennung war, ist mein Vater mit meiner neuen Frau in die [ANONYMISIERT, ORT IN DEUTSCHLAND] gezogen. Und, ja. Ist immer noch so. Also mein Vater ist mit seiner Frau und mit ähm deren Kinder von der Frau in der [ANONYMISIERT, ORT IN DEUTSCHLAND]. (I: Hm (zustimmend)) Und der ganze Rest in [ANONYMISIERT, ORT IN DEUTSCHLAND]. |
| 16 | **I:** Und haben Sie Geschwister? |
| 17 | **AB04:** Hm ja angeheiratete Ge- Stiefgeschwister. Eine Stiefschwester und einen Stiefbruder. Beide älter als ich. |
| 18 | **I:** Okay. (AB04: Ja.) Und die ähm die gab es aber schon bevor sich Ihre Eltern getrennt haben? Das heißt, die waren von Ihrem Vater, oder von Ihrer Mutter? |
| 19 | **AB04:** Die waren äh das sind die Kinder von der Frau von der neuen Frau von meinem Vater (I: Ah ja okay! Verstehe.) Ja. Und dadurch, dass mein Vater und die Frau geheiratet haben, (I: Hm (zustimmend)) sind es meine Stiefgeschwister. |
| 20 | **I:** Alles klar, (AB04: Ja.) alles klar. Also jetzt von Ihrem Vater und Ihrer Mutter gibt es keine Geschwister für Sie? |
| 21 | **AB04:** Nein, ich bin Einzelkind. |
| 22 | **I:** Okay, okay. Und ähm weshalb sind Sie dann zu Ihrem Vater gezogen? Was war da der Grund? |
| 23 | **AB04:** Ähm mehr wegen Drogen (I: Wegen Drogen?), ja. Ich bin suchtkrank geworden und meine Mom wollte mich aus dem Kreis noch raushauen (I: Hm (zustimmend)). Und hat mich dann praktisch von [ANONYMISIERT, ORT IN DEUTSCHLAND] nach [ANONYMISIERT, ORT IN DEUTSCHLAND] zum- hm. Ja. Aber das ging da auch weiter. |
| 24 | **I:** Da gings ja auch weiter (AB04: Ja.) Okay. Und wie ist es jetzt mit den Drogen? |
| 25 | **AB04:** Ja seit, seit Mitte Januar bin ich clean. |
| 26 | **I:** Okay. (AB04: Ja.) Und wie haben Sie das- |
| 27 | **AB04:** Mit meiner Freundin zusammen, (I: Hm (zustimmend)) haben wir es gemacht (I: Hm (zustimmend)) ähm an dem Tag, wo wir ähm mitgekriegt haben, dass wir Eltern werden. |
| 28 | **I:** Aah! (AB04: Ja.) Das war der Auslöser sozusagen (AB: Ja, genau, das war der Erlöser.) und dann haben Sie einfach selber entschieden: So, (AB04: Ja genau.) jetzt reicht es (AB04: Ja.). Keinen Entzug gemacht? |
| 29 | **AB04:** Nee gar nix. (I: Nee? Okay) Doch also immer mal wieder im Leben, klar, in den Kliniken, und a- a- auch mal alleine. Also im- immer mal wieder, ja. Hm. |
| 30 | **I:** Okay. Und jetzt das letzte Mal haben Sie es ganz alleine geschafft? |
| 31 | **AB04:** Ja (I: Schön). Ja. Aber die Psychose ist trotzdem wieder gekommen (I: Okay.). Also ich habe ja im Januar aufgehört und ist dann trotzdem immer stärker geworden und dann bin ich Mitte April ja hier in auf Station [ANONYMISIERT, NAME EINER ÖRTLICHEN PSYCHIATRISCHEN STATION] in die Klinik gekommen. |
| 32 | **I:** Ja, ja (zustimmend). Und ähm wann war die erste Psychose? |
| 33 | **AB04:** 2013 glaube ich. 2013, 14 so (I: Hm (zustimmend)). Ich bin mir nicht mehr ganz sicher (I: Hm (zustimmend)). Ist schon, ist schon eine Weile her. |
| 34 | **I:** Wie alt waren Sie da? Darf ich das einmal aufschreiben? |
| 35 | **AB04:** Ja [KUGELSCHREIBER KLICKT], da war ich 19, 20. |
| 36 | **I:** 19. [KURZE PAUSE] Und wie haben Sie das gemerkt? |
| 37 | **AB04:** Am Anfang, hm. Also am Anfang war ich mir gar nicht so sicher. Muss ich ganz ehrlich sagen. Ähm. Ich ähm ich habe mich verfolgt gefühlt, dann mal wieder nicht. Dann äh gab es hm komische- komische Erlebnisse, wie dass man hm aus dem Fernseher oder aus hm oder aus anderen Sachen ähm Dinge auf sich bezogen hat (I. Hm (zustimmend)). Wo man wieder- wiederum gleichzeitig gedacht hat so hm, hm als normales Denken, so, hä, das kann ja gar nicht sein (I: Ja) oder was (I: Ja). Ja genau, ja. Und das ist dann eigentlich immer stärker geworden. Ja. |
| 38 | **I:** Über was für einen Zeitraum ungefähr? |
| 39 | **AB04:** Hm so ein halbes, halbes dreiviertel Jahr. |
| 40 | **I:** Und dann, wie- was haben Sie dann gemacht? |
| 41 | **AB04:** Äh mein Vater hat mich in die Psychiatrie gebracht (I: Okay.). Ja. In [ANONYMISIERT, ORT IN DEUTSCHLAND], damals noch (I: Hm (zustimmend)). Ja. |
| 42 | **I:** Weil Sie mit ihm das geteilt haben, sozusagen, was da passiert ist? |
| 43 | **AB04:** Äh ja, ich hatte ja- ich hatte sehr große Angst gehabt und bin alleine nicht mehr raus und alles (I: Hm (zustimmend)) und habe gedacht, ich muss sterben und mein Vater hat das natürlich gemerkt. Ich war fix und fertig (I: Ja, ja.). Ja. Und die- die Frau von meinem Vater hat dann gesagt, dass es eine Krankheit ist und hat ähm- mich in die Psychiatrie gebracht, ja. Da war ich aber da der Überzeugung, dass es keine Krankheit ist, sondern dass es Wirklichkeit ist (I: Okay.). Ja. |
| 44 | **I:** Und das ist dann öfter passiert, so, oder wie? |
| 45 | **AB04:** Ja, immer mal wieder in den- (I: Ja) in den Jahren jetzt seit 2013 (I: Hm (zustimmend). 12, 13. Ja. |
| 46 | **I:** Waren Sie dann immer mal wieder in der Klinik. |
| 47 | **AB04:** Fast nur, muss ich sagen. (I: Ja?) Also ich war, ich war 2013 äh in [ANONYMISIERT, ORT IN DEUTSCHLAND], ungefähr, ein Vierteljahr, drei Monate, vier Monate, dann bin ich von da aus in die Drogentherapie nach [ANONYMISIERT, ORT IN DEUTSCHLAND] (I: Hm (zustimmend)). Von da aus in die Nachsorge nach [ANONYMISIERT, ORT IN DEUTSCHLAND] (I: Hm (zustimmend)), dann war ein halbes Jahr Pause. In dem halben Jahr war ich obdachlos, da habe ich- hm, ha- (nuschelt) weil ich es mit dem System nicht so auf die Reihe gekriegt hab. Hm. Und dann bin ich wieder in die- dann bin ich in die Psychiatrie das erste Mal hier (I: Hm (zustimmend)) in der [ANONYMISIERT, ORT IN DEUTSCHLAND], das war dann 2016. Dann bin ich von da aus noch nach [ANONYMISIERT, ORT IN DEUTSCHLAND], dann bin ich von da aus ähm- da weiß ich jetzt gar nicht mehr, was danach kam. Hm. |
| 48 | **I:** Also so ein- Sie sind so (AB04: Ja!) von Klinik zu Klinik. |
| 49 | **AB04:** Boa, es es nim- kei- es nimmt kein Ende. |
| 50 | **I:** Okay, und wo sind (AB04: Ja.) Sie jetzt? |
| 51 | **AB04:** Wieder in der Klinik (kleinlaut). |
| 52 | **I:** Aber sind Sie ja zu Hause auch, zum Teil. (AB04: Ja stimmt, ja) Wo ist zu Hause denn? |
| 53 | **AB04:** Ja, das ist das erste Mal, dass ich habe die Psychose so schnell in den Griff gekriegt (I: Ja.). Weil ich bin im April hin, und musste nicht mal auf eine fortführende Klinik oder so (I: Ja, ja.). Also ich habe- ich gehe ja jetzt schon wieder alleine nach Hause meistens. Das ist das erste Mal, dass ich habe das so schnell in den Griff gekriegt und da bin ich eigentlich stolz darüber. Also, dass ich mich habe getraut rauszugehen, (I: Ja, ja.) dass nichts passiert ist und ja, da bin ich sehr froh drüber. |
| 54 | **I:** Und wo gehen Sie dann hin, wenn Sie nach Hause gehen jetzt? |
| 55 | **AB04:** Äh in [ANONYMISIERT, ORT IN DEUTSCHLAND]. |
| 56 | **I:** Okay (AB04: Ja.) Und da wohnen Sie alleine, oder? |
| 57 | **AB04:** Äh nee das ist eine WG. Ein Zimmer steht leer, ein Zimmer wohnt da- da ist [ANONYMISIERT, NAME EINES MITBEWOHNERS] drin. Wir verstehen uns gut. Ist in meinem Alter. Und hm eins gehört mir. |
| 58 | **I:** Und wer organisiert diese WG? Also wie- (AB04: Hm.) Sie haben sie sich selber gesucht oder ist das ein Träger? |
| 59 | **AB04:** Äh das ist vom Amt für Soziales und Senioren in der [ANONYMISIERT, ORT IN DEUTSCHLAND] (I: Ah ja, ah ja, okay). Ja. Aber organisieren tut da keiner was. Also das ist ne Wohnung, wir werden in Ruhe gelassen wie jeder. Wir haben einen Bezug zu dem Amt, so wie man halt einen Bezug zum Vermieter: gar nicht [LACHT]. Ja. |
| 60 | **I:** Also Sie machen alles selbst, (AB04: Ja, genau ja) kaufen selber ein (AB04: Ja, alles selber). Alles klar. Ja super (AB04: Hm (zustimmend)). Ist doch- also dann sind Sie ja eigentlich gar nicht in der Klinik. Sie kommen ja nur wie zum Job, kommen Sie tagsüber hierher, oder? |
| 61 | **AB04:** Ja, stimmt, (I: jetzt) ja, jetzt, jetzt. Ich war 12 Wochen auf [ANONYMISIERT, NAME EINER ÖRTLICHEN PSYCHIATRISCHEN STATION] (I: Hm (zustimmend)) und da war ich hm nicht hm wie im Job. |
| 62 | **I:** Nee, das stimmt (AB04: Ja.), da waren Sie- da sind Sie stationar, aber jetzt (AB04: Ja.) ist ja eine Tagesklinik, also (AB04: genau) im Prinzip wie eine Arbeit (AB04: Ja), hm. Okay, schön. |
| 63 | **AB04:** Ich bin sehr foh darüber, dass ich alles so schnell in den Griff gekriegt habe, weil [ANONYMISIERT, NAME DER PARTNERIN DER INTERVIEWTEN PERSON] ist ja schwanger. Und wenn ich jetzt noch in drei fortführenden Kliniken hätte gemusst, zum Beispiel, (I: ja) dann wäre die Zeit alles weggewesen, deswegen. |
| 64 | **I:** Ja, aber sie ist noch schwanger oder sie hatte- |
| 65 | **AB04:** Sie- sie ist noch schwanger, am 12.8. kommt der Kleine auf die Welt. Aufgrund, äh der- auf d- aufgrund, weil sie hat Epilepsie, wird es ein geplanter Kaiserschnitt. |
| 66 | **I:** Ah ich dachte, das wär schon da, das Kind? |
| 67 | **AB04:** Nee, nee, ist noch nicht da (I: Achso (erstaunt)). Am 12.8. ist das. Ja, ich habe schon hier gefragt, und da habe ich frei bekommen für den Tag. |
| 68 | **I:** Super (AB04: Ja). Super, ja dann sind Sie dabei, natürlich. |
| 69 | **AB04:** Ja klar (I: schön). Ja [ANONYMISIERT, NAME DER PARTNERIN DER INTERVIEWTEN PERSON] wollte, dass ich die Nabelschnur durchschneide, weil ich habe ihr ganz stolz erzählt, wie ich das habe damals bei meiner Tochter gemacht. Aber ich habe zu ihr gesagt, dass ich das nicht machen kann. |
| 70 | **I:** Warum? |
| 71 | **AB04:** Weil, weil sie hat ja einen Kaiserschnitt und- und wenn ich das sehe alles, die Bilder werde ich nie wieder los (I: Ja) das ist, ja. Das ist ähm keine, keine gute Idee für mich. |
| 72 | **I:** Gute Entscheidung. (AB04: Ja) Also sie müssen ja auch entscheiden, wo sind meine Grenzen (AB04: genau, ja) was kann ich, was kann (AB04: ja) ich nicht. |
| 73 | **AB04:** Das hat sie auch gut auch aufgenommen (I: ja), dass ich nein gesagt habe. (I: super), ja. |
| 74 | **I:** Super. Ja ein Kaiserschnitt zu sehen, ist nicht ohne, (AB04: nee, ist nicht ohne) das kann ich mir vorstellen, ja, okay. Aber toll, also das klingt als gibt es Pläne, die jetzt in die Zukunft gehen. (AB04: ja, genau) Jetzt läuft (AB04: ja) die ganze Geschichte. Schön. (AB04: ja) Schön. |
| 75 | **AB04:** [RÄUSPERT SICH] |
| 76 | **I:** Okay, dann lassen Sie uns so ein bisschen über Ihre Kognitionen, über Ihre Gedanken, alles was so innerlich an Gedanken passiert und in Ihrem Kopf abgeht, (AB04: Hm) sprechen. Gibt es da irgendwas, was Ihnen sofort einfällt? Gedanken, Konzentration? Können Sie Ihre Gedanken immer gut- |
| 77 | **AB04:** Kon-Konzentration so gut wie Null. (I: Aha) Also ich habe, ich habe ein gutes Beispiel. Ich habe ähm früher gern Schach gespielt. Ich war auch sehr gut, ein sehr guter Schachspieler (I: ja, ja (zustimmend)) und äh habe das ähm also habe- ich habe einen Freund, der heißt [ANONYMISIERT, NAME EINES FREUNDES DER INTERVIEWTEN PERSON] (I: Hm (zustimmend)) und der war mh und der kann kein Schach (I: Hm (zustimmend)) und dem- und der hat dann ein Schachbrett gekauft und dann wollten wir Schach spielen und alles so und dann wollte ich es ihm halt beibringen und so. (I: Ja) Und er hat mich besiegt, besiegt, besiegt, ich konnte mich überhaupt nicht konzentrieren (I: Okay) Ja. |
| 78 | **I:** Und wenn Sie sagen "früher", wann war denn früher? |
| 79 | **AB04:** Früher war. Hm, ja. So bis- bis zum 17., 18. Lebensjahr. |
| 80 | **I:** Okay (AB04: Ja), und da haben Sie viel Schach gespielt? |
| 81 | **AB04:** Nee, nee, nee (energisch). Das- das war in der Kindheit (I: Okay). In- in der Schule hatte ich einen Schachkurs, (I: okay) in der Grundschule. (I: okay) Ja. Ich meine, mit der Konzentration ist es erst bergab gegangen seit dem 17, 18. Lebensjahr. |
| 82 | **I:** Alles klar. (AB04: Ja) Alles Klar. So. Mit 17 und dann mit 19 war die erste Psychose? |
| 83 | **AB04:** Ja, so 17, 18. Genau. |
| 84 | **I:** Da hat das schon angefangen. (AB04: Ja) Aber hatte das was mit Drogen zu tun? Oder- oder (AB04: Hm) kam das auch so? |
| 85 | **AB04:** Das weiß ich gar nicht so genau. Man hat in den ersten Kliniken gesagt, das ist eine drogeninitiierte Psychose ist, was ich habe (I: Hm (zustimmend)). Der Doktor [ANONYMISIERT, NAME EINES ARZTES] hat jetzt vor ein paar Wochen gesagt, dass die Schizophrenie ist nicht äh von Drogen ausgelöst also. Ich weiß es ehrlich gesagt selber gar nicht mehr genau. |
| 86 | **I:** Wie war das denn mit den Drogen? Wann haben Sie damit angefangen? |
| 87 | **AB04:** Hm an meinem 13. Lebensjahr, (I: Okay) angefangen Cannabis zu rauchen (I: Okay) Ja. Und später, als ich älter wurde, wurden denn auch härte Drogen. (I: Hm (zustimmend)) Hm. |
| 88 | **I:** Ja gut, dann kann man nicht so richtig sagen, ne? Woran lag es jetzt (AB04: Ja, ja, ka- kann man nicht!) dass die Konzentration sich verändert hat? (AB04: Ja) Wobei, wenn Sie sagen, Sie haben mit 13 schon angefangen Drogen zu nehmen und mit 17 hat sich erst die Konzentration verändert- (AB04: Hm) weiß man nicht so genau. |
| 89 | **AB04:** Ja gut, man-man fängt nicht an Drogen zu nehmen und von einem Tag auf den anderen geht das ganze den Bach runter. (I: das stimmt) Also, das- das geht ja langsam und schleichend. |
| 90 | **I:** Ja. (AB04: Ja) Ja. Wobei fünf Jahre ja schon- das ist schon viel, ne? (AB04: Hm (zögerlich). Also woran haben Sie denn gemerkt, dass- also Sie haben gemerkt, ich kann mich beim Schach nicht mehr konzentrieren? |
| 91 | **AB04:** Ja, das war jetzt ein Beispiel, wo aktuell ist. (I: Aktuell) Das ist- das ist jetzt erst einen Monat her, wo ich (I: okay) grad hier erzählt habe, (I: okay) ja. |
| 92 | **I:** Und, wenn Sie jetzt- wenn Sie jetzt sagen, mit 17, 18 ging es mit der Konzentration- mit der Konzentration den Bach runter, was war da anders? |
| 93 | **AB04:** In der Schule habe ich es gemerkt. (I: Hm (zustimmend)) Ja. Oder was heißt in der Schule? Das war nun schon- das war dann schon äh das ist [ANONYMISIERT, NAME EINES JUGENDHEIMS] (I: Ja.) war das, ja. (I: Was haben Sie-) Schule hatte ich da schon abgeschlossen, und da habe ich meinen Hauptschulabschluss nachgeholt. (I: Okay) Ja und da habe ich dann schon- |
| 94 | **I:** Was haben Sie da gemerkt? |
| 95 | **AB04:** Hm (zögerlich). Na dass ich mich nicht mehr konzentrieren kann. |
| 96 | **I:** Dass Sie nicht zuhören können? Oder nicht mehr- |
| 97 | **AB04:** Ja, genau, oder- oder so etwas hier wie den Text zu lesen. Ich lese einen Satz, den zweiten Satz, dann habe ich den ersten schon wieder vergessen, (I: Okay) dann überspringe ich einen, weil es mir so anstrengend ist und ähm so gehe ich dann den ganzen Text durch, also- |
| 98 | **I:** Verstehe. (AB04: Ja) Okay, okay. Und das ist heute so, und das war damals auch schon so? |
| 99 | **AB04:** Ja, schon. (I: Ja?) Ja. (I: Hm) Ich habe auch heut- als Kind wurde diagnostiziert ähm LHS [/LRS/] und ADHS, bei mir. |
| 100 | **I:** Also Konzentrationsschwierigkeiten. |
| 101 | **AB04:** Konzationsschwierigkeiten [/Konzentrationsschwierigkeiten/] und ähm Legio- Legasteniker also, ja. |
| 102 | **I:** Okay, (AB04: Hm) das ist fest diagnostiziert? |
| 103 | **AB04:** Hm ja, als Kind halt, das- das war- |
| 104 | **I:** Hat sich das verändert, ist das jetzt- Wie ist mit Legasthenie heute? |
| 105 | **AB04:** Hm. Also ich kann, ich kann schreiben, ich kann lesen, äh aber nicht so schnell und so gut wie andere. |
| 106 | **I:** Okay, (AB04: Ja) okay. Okay, aber Sie sagen, wenn Sie jetzt einen Text lesen, wie gerade das, was wir hier- was wir liegen haben, (AB04: ja) dann äh fällt es Ihnen schwer, sich an den, an den ersten Satz davor (AB04: Ja genau) zu erinnern? |
| 107 | **AB04:** Oder überhaupt manchmal den Satz zu verstehen, was ich gelesen habe. |
| 108 | **I:** Also schon die Worte, davor? |
| 109 | **AB04:** Ja. (unverständlich). Ah nee! Einzelne Worte gehen. Also wenn ich jetzt hier zum Beispiel "Zeit" oder "stellen", verstehe ich direkt, dauert nicht mal eine Sekunde. Aber den ganzen Satz, den Zusammenhang dann, und dann gleich den nächsten dahinter, und das ist (I: Hm (zustimmend)) dann dies- diese Masse (I: ja) oder so etwas, das ist dann zu viel, (I: okay) ja. |
| 110 | **I:** Okay. Alles klar. Und wie sieht es mit- mit Gedanken aus? Können Sie, wenn Sie irgendwie über irgendwas nachdenken, (AB04: Hm) können Sie dem gut folgen oder wird das auch manchmal unterbrochen von- |
| 111 | **AB04:** Ja, es wird unterbrochen. Also ich kann dem gut folgeleisten. Hm. Ja. |
| 112 | **I:** Und, ähm, kann es sein, dass mal so ein Gedanke zwischen reinschießt und Sie dann vergessen haben, worüber Sie vorher nachgedacht haben? |
| 113 | **AB04:** Ja, ja. (I: Ja?) Gut, oder dass ich den Faden verliere so, ja. (I: Ja?) Ja, passiert. (I: das passiert?) Ich spreche mit meiner Mom, und mittem im Satz ist es dann auf einmal weg und dann weiß ich nicht mehr, was ich sagen wollte. |
| 114 | **I:** Okay, also das ist wie plötzlich gelöscht. (AB04: ja, genau) Nicht dass es so langsam verfliegt, (AB04: mh-mh (verneinend)) und Sie es nicht greifen können, sondern (AB04: nee) es ist wie plötzlich gelöscht? |
| 115 | **AB04:** Nee, manchmal kommt es auch wieder. Ich überleg dann "hä, was wollte ich denn jetzt sagen?". Manchmal kommt es nicht wieder, manchmal kommt es wieder. |
| 116 | **I:** Und passiert das oft? |
| 117 | **AB04:** Ja. |
| 118 | **I:** Ja? |
| 119 | **AB04:** Drei, vier Mal am Tag. |
| 120 | **I:** Ah, ja, okay. Und das ist jetzt gerade, auch was, was Ihnen auffällt? Also- |
| 121 | **AB04:** Das habe ich jetzt gerade auch, ja. |
| 122 | **I:** Ja? Okay. Hm. Und war das dann früher auch schon vorher so? Wenn wir jetzt an die Zeit vor der Psychose denken, zum Beispiel, war das da auch so? |
| 123 | **AB04:** [KURZE PAUSE]. Äh, weniger, (I: weniger) das- das war eher weniger. |
| 124 | **I:** Das hat sich jetzt erst entwickelt, okay. Ja. Und ähm kann es auch passieren, dass Gedanken ganz viel werden, wie viele Gedanken auf einmal, oder dass sie so schnell werden dann auch? |
| 125 | **AB04:** [KURZE PAUSE] Dass die Gedanken schnell werden? |
| 126 | **I:** Hm, dass man das Gefühl hat, die rasen so im Kopf. |
| 127 | **AB04:** So, ja. Ja, schon (I: Und es sind so viele-) also was heißt rasen, das ist eher so ein, dass man hört nicht auf. "Das nachdenken", "das nachdenken", und "das nachdenken", aah, "das nachdenken" "und das" "und das". (I: Okay) Und dann ähm das geht nicht unbedingt schnell, aber es nimmt kein Ende |
| 128 | **I:** Okay. (AB04: Ja) Also, es geht so ein bisschen weg, von der eigenen Kontrolle? Man kann es nicht mehr kontrollieren? |
| 129 | **AB04:** Ja, genau, ja. |
| 130 | **I:** Ja? Okay. Und ähm fühlt sich das dann so an als drängen die Gedanken einem so auf, als kann man sie nicht stoppen? |
| 131 | **AB04:** Ja, ja (I: Wie ist es für Sie?) Ja genau, ja. Das trifft es |
| 132 | **I:** Das trifft es ganz gut? Und ähm ist es auch tagsüber- jetzt, jetzt gerade so? |
| 133 | **AB04:** Ja, ist immer, immer so. |
| 134 | **I:** Immer so? |
| 135 | **AB04:** Ja. |
| 136 | **I:** Und belastet Sie- |
| 137 | **AB04:** Am- am besten am- kann ich mich konzentrieren morgens. Und alles, und das- am besten. Und wenn ich den ganzen Tag erstmal erlebt habe so, dann ist immer abends am schlimmsten. |
| 138 | **I:** Okay, abends liegen Sie dann im Bett und machen sich über den Tag Gedanken, oder wie sieht das aus? |
| 139 | **AB04:** Hm ja, genau. |
| 140 | **I:** Ja? |
| 141 | **AB04:** Hm. |
| 142 | **I:** Könnten Sie mir das beschreiben, woran denken Sie dann? |
| 143 | **AB04:** [PAUSE] Das kann ich nicht wiedergeben. |
| 144 | **I:** Nee? |
| 145 | **AB04:** Nee. Weiß ich nicht. |
| 146 | **I:** Also Inhalte fallen Ihnen nicht- |
| 147 | **AB04:** Es ist, w- ist qu- quer gemischt eigentlich. |
| 148 | **I:** Quer Beet, sozusagen. |
| 149 | **AB04:** Ja, quer Beet, ja. (I: Alles) Normale Sachen, so wie jetzt mit [ANONYMISIERT, NAME DER PARTNERIN DER INTERVIEWTEN PERSON] (I: Aha) oder- oder unser Gespräch hier. (I: Ja, ja) Hm, ja und dann Sachen, wo nicht normal sind wie, wo ich zum Beispiel hm letztens in der Bahn gestanden habe an der Tür und hinter mir einer gelacht und ich habe gedacht, der lacht über mich. (I: aha (verständnisvoll)) und so etwas- so etwas- ist alles gemischt. |
| 150 | **I:** Okay. (AB04: Ja) Okay. Also es geht so um was Sie erlebt haben, das denken Sie dann abends noch einmal durch. |
| 151 | **AB04:** Nö, nicht immer. Das kommt automatisch. |
| 152 | **I:** Ah, das ah- okay, das kommt einfach so? |
| 153 | **AB04:** Ja, ja. |
| 154 | **I:** Die Gedanken? |
| 155 | **AB04:** Ich- ich leg- ich leg mich jetzt nicht ins Bett und denke so, so heute, jetzt denkst du noch mal über den heutigen Tag nach. (I: nee) So nicht, also das kommt automatisch. |
| 156 | **I:** Das fliegt so rein. |
| 157 | **AB04:** Ja genau. |
| 158 | **I:** Aha, okay. Und machen Sie sich dann auch viel Gedanken über Ihr eigenes Verhalten? Also habe ich mich jetzt (AB04: ja) richtig verhalten (AB04: ja, sehr, sehr) oder falsch verhalten? (AB04: sehr viel, ja). Und können Sie mir da ein Beispiel sagen? |
| 159 | **AB04:** Na, ich weiß, dass es blöd ist, dass ich heute verschlafen habe (kleinlaut). |
| 160 | **I:** Okay, okay (verständnisvoll). (AB04: Ja) Das- das ist- ja. Aber ich meine, das heißt, Sie gehen dann heute Abend nach Hause und denken da quer- quer darüber nach, "wie hätte ich das anders machen können", oder? |
| 161 | **AB04:** Hm, nee (I: nee). Nicht unbedingt, also ich weiß, ich bin ja jetzt schon 14 Wochen hier und jetzt- ich bin zwei Mal zu spät gekommen jetzt oder zwei Mal einen Ausrutscher hatte ich. Ich denke, das ist noch zu verkraften. (I: Ja) Äh wenn es jetzt nicht o-öfters passiert, (I: ja) ja, da mache ich mir jetzt nicht so einen Kopf. |
| 162 | **I:** Okay. Hm. Und wenn Sie sich über Ihr eigenes Verhalten Gedanken machen: Ist es dann so, dass Sie sich fragen "habe ich das richtig gemacht?" oder dass Sie nicht genau wissen, ob Sie Sachen richtig gemacht haben, oder falsch oder so? Oder wie- wie ist, wie denken Sie über sich selbst? |
| 163 | **AB04:** Ja genau, ich stehe an der Ampel und dann stelle ich mich hin und dann weiß ich nicht wie ich mich hinstellen soll. (I: Hm (zustimmend)) Und dann- und dann läuft jemand los und dann laufe ich auch los und dann hm also wenn es grün ist natürlich, ja (I: okay) und dann denke ich "mh, der ist schon längst über alle Berge" und ich denke nach noch über die Ampel und halt, aber (undeutlich). |
| 164 | **I:** Alles klar, alles klar. Und kann es auch sein, dass Sie dieses- dieses Grübeln, sagen wir auch, dieses Nachdenken über sich selber, dass Sie das daran hindert, spontan zu reagieren oder in einem Gespräch spontan zu antworten? |
| 165 | **AB04:** Hm, nö eigentlich nicht. |
| 166 | **I:** Nicht, okay, so stark ist es nicht, (AB04: nein) dass Sie jetzt zum Beispiel während wir reden- |
| 167 | **AB04:** Kann sein, dass ich es schon mal erlebt habe, aber (I: Ja?) habe es wieder vergessen. |
| 168 | **I:** Okay. (AB04: Ja) Okay. (AB04: Hm (leise)). Also gab es Phasen, wo Sie- wo Sie ähm viel darüber nachgedacht haben: wie verhalte ich mich jetzt? Verhalte (AB04: ja auf jeden Fall, ja) ich mich richtig? Und dass das auch Sie gehindert hat daran- |
| 169 | **AB04:** Ähm ich habe ja jahrelang geglaubt, ich muss sterben (I: mh-mh?) und dann haben- und dann ist man halt- wie- wie soll- wie soll man das beschreiben? Man hat halt selber so das Gefühl, dass man nichts wert ist, so wenn man umgebracht werden soll, (I: ja) so, (I: ja) ja, und ja. Und da habe ich immer gedacht, so "mache ich das" und hm und "jetzt hast du einen Ausdruck gesagt" und "darf ich das überhaupt?", ja. |
| 170 | **I:** Okay. (AB04: Hm (leise)) Also das ging dann auch einher mit einem Gefühl "Ich bin weniger wert, weil ich habe mich falsch verhalten"? |
| 171 | **AB04:** Ja, genau, (I: ja?) ja hm. |
| 172 | **I:** Okay, verstehe. Ja das ist belastend, ne? |
| 173 | **AB04:** Ja. |
| 174 | **I:** Ja. |
| 175 | **AB04:** Hm, es ist wirklich belastend. |
| 176 | **I:** Ja, das glaube ich. |
| 177 | **AB04:** Mal-mal mehr, mal weniger (kleinlaut). |
| 178 | **I:** Okay. Wie ist es jetzt gerade? |
| 179 | **AB04:** Hm. [PAUSE] Ja, eigentlich okay. |
| 180 | **I:** Also hat sich gebessert? |
| 181 | **AB04:** Hm (zögerlich). |
| 182 | **I:** Ja? |
| 183 | **AB04:** Ja, hat sich gebessert, ja. Ich kann draußen alles, und ja. Habe mir jetzt zwar morgen einen Einlauf von meiner Freundin abgeholt, weil ich zu spät gekommen bin, aber ansonsten ist hm alles okay. |
| 184 | **I:** Okay. (AB04: hm) Und diese Gedanken, die Sie haben, kommen die Ihnen manchmal auch fremd vor, oder sind das immer Ihre Gedanken? |
| 185 | **AB04:** Ja, kommt mir fremd vor. |
| 186 | **I:** Wie ist es, können Sie mir beschreiben so einen Gedankengang der fremd vorging oder Situation, wo das so war vielleicht? |
| 187 | **AB04:** Hm ja, wie das mit der Ampel, wo ich grad erzählt habe. (I: Ja?) Denn ähm oft habe ich ähm stelle ich mich selber so die Frage: "wie war es früher so, da hast du dir ja auch nicht so- so'ne Fragen gestellt" (I: Hm (zustimmend)) und denn denke ich, da- dadurch, dass ich mir diese Frage gestellt habe, kommt mir dann eben die Ampel, mit der Situation, die ich gerade erklärt habe, fremd vor. |
| 188 | **I:** Und, fremd heißt, dann haben Sie das Gefühl, dass da jemand anderes Ihren Gedanken beeinflusst? Oder dass die nicht von Ihnen gemacht (AB04: ja, auch, ja) wurden? |
| 189 | **AB04:** Alles gemischt (I: Okay). Alles ist ein- ein Q- Querbeet. Ja. Manche Gedanken sind meine, wie wenn ich jetzt zum Beispiel von hm von [ANONYMISIERT, NAME DER PARTNERIN DER INTERVIEWTEN PERSON] oder so (I: ja) oder wo jetzt mit dem Zuspätkommen, das ist ja auch meine Schuld. Ja, manche Gedanken halt sind nicht meine. |
| 190 | **I:** Die kommen von woanders? Oder- |
| 191 | **AB04:** Ja, k- mh. Das ist schwer zu beschreiben. |
| 192 | **I:** Ja, das klingt komisch, (AB04: ja) ich weiß. |
| 193 | **AB04:** Ja, das klingt komisch, (I: Ja) Sie haben recht. Jetzt wo ich selber mal so hör, so habe ich das noch nie gesehen. (I: ja) Also das klingt echt komisch. Also es sind ja klar meine Gedanken weil es äh ich denke es ja. (I: ja) Aber ich habe nicht das Gefühl, dass ich es- dass es halt meins ist. (I: ja) Also, fremd, komisch irgendwie. |
| 194 | **I:** Ja, aber das ist nicht- also das beschreiben viele Leute, das ist nicht selten, das ist- (AB04: ja) deswegen frage ich danach. (AB04: ja) Aber- aber Sie haben recht, das klingt komisch und dann fragt man sich: "Okay, wenn es nicht, wenn die Gedanken nicht meine sind, woher kommen sie dann?" |
| 195 | **AB04:** Ja. |
| 196 | **I:** Ja, okay. |
| 197 | **AB04:** Hm, das weiß ich aber nicht. |
| 198 | **I:** Das weiß man nicht, ist okay, muss man nicht wissen. (AB04: hm) Aber also, Sie haben das klare Gefühl bei manchen, die sind nicht von mir, die Gedanken? |
| 199 | **AB04:** Ja |
| 200 | **I:** Okay. (AB04: Hm) Und das war auch heute noch so? Öfter? |
| 201 | **AB04:** Ja, wie zum Beispiel jetzt, wo das praktisch- wo ich hm, wo ich genannt hab, wenn ich an der Tür gest- hm, an der Tür gestanden habe, an der Bahn, und hinter mir lacht einer, der lacht über mich, (I: Hm (zustimmend)) denn ist das ein Gedanke, wo zur Krankheit gehört, wo halt nicht meiner ist. |
| 202 | **I:** Okay, (AB04: so) okay. (AB04: Ja) Also die Krankheit ist sozusagen zusätzlich, die kommt darein? |
| 203 | **AB04:** Ja, das ist halt, genau- auf das normale G- Gedenkensgerüst wird halt noch etwas draufgesetzt. |
| 204 | **I:** Alles klar, alles klar, okay [RÄUSPERT SICH]. |
| 205 | **AB04:** Muss ich auch dazu sagen, bin ich stolz, dass ich heute so reden kann. (I: Ja (bekräftigend)) Weil noch vor zwei Jahren wa- hätte man gesagt: "bist du krank?", habe ich gesagt: "ne?" (I: Ja) Also, da war es- ja. |
| 206 | **I:** Dass Sie das so reflektieren, und von viel- vielen Seiten beschreiben können, ist super. Super gut und- |
| 207 | **AB04:** Wie meinen Sie das? |
| 208 | **I:** Naja, dass Sie jetzt zu mir sagen können, da gibt es ein Gerüst, (AB04: Hm) das kommt dadrauf, (AB04: Ja) das ist super gut, weil das hilft Ihnen, ne? |
| 209 | **AB04:** Ja, genau. |
| 210 | **I:** In Situationen, (AB04: ja stimmt) wo es schwieriger wird. |
| 211 | **AB04:** Richtig, dann denke ich wieder an das, was- was ich jetzt gerade gesagt habe (I: genau) und dann geht es mir wieder besser, (I: genau) ja, (I: ja super) ja stimmt. |
| 212 | **I:** Ähm wie ist es ähm haben Sie manchmal das Gefühl, Gedanken sind wie nachgesprochen, wie so ein Echo im Kopf? Haben Sie so etwas schon mal erlebt? |
| 213 | **AB04:** Hm (zustimmend). |
| 214 | **I:** Ja? |
| 215 | **AB04:** Ja (kleinlaut). |
| 216 | **I:** Haben Sie da ein Beispiel? |
| 217 | **AB04:** Ein hm ich habe jetzt kein direktes Beispiel, aber einzelne Sätze, was Leute ähm gesagt haben mal zu mir oder so äh bleibt Jahre. |
| 218 | **I:** Ja? |
| 219 | **AB04:** Ja. |
| 220 | **I:** Okay. Und das heißt? |
| 221 | **AB04:** Ich kann jetzt kein Beispiel nennen, weil ich vergesse immer wieder, (I: Hm (zustimmend)) aber es kommt auch immer wieder. (I: Okay) Ja und ähm vo- vor fünf oder sechs Jahren hat mal ein Kollege was zu mir gesagt, das weiß ich heute noch. |
| 222 | **I:** Okay. (AB04: Ja) Und das haben Sie dann richtig im Kopf, (I: Ja das habe ich dann), das- das hallt dann wie? |
| 223 | **AB04:** Ja, genau ja. |
| 224 | **I:** Alles klar. Alles klar. Und hatten Sie auch schon mal ähm das Gefühl, Ihre eigenen Gedanken sind laut im Kopf, die können Sie hören? |
| 225 | **AB04:** Ich kann die hören, ja. |
| 226 | **I:** Also wie gesprochen im Kopf? |
| 227 | **AB04:** Genau. (I: ja?) Hm. |
| 228 | **I:** Okay? Und ähm, das ist also dann wie eine innere Stimme, die diese Gedanken spricht? |
| 229 | **AB04:** [ATMET AUS] Ja. Ja, gut wenn man halt was hm äh denkt, dann ist es ja so, wie als wenn man redet, aber es redet keiner. |
| 230 | **I:** Hm (zustimmend). (AB04: So) So hören Sie das dann. (AB04: Mh, ja) So deutlich, auch? |
| 231 | **AB04:** Ja. |
| 232 | **I:** Ja? Okay. Und ähm, hatten Sie da auch schon mal Angst, dass die Gedanken so laut sind, dass andere Leute die hören können? |
| 233 | **AB04:** Hm habe ich auch schon mal gedacht, ja. |
| 234 | **I:** Haben Sie auch schon erlebt? |
| 235 | **AB04:** Ja, hm. |
| 236 | **I:** Gibt es da ein Beispiel dafür? |
| 237 | **AB04:** Ja, wo ich ähm extrem psychotisch war, wo ich durch die Stadt gelaufen bin, da habe ich gedacht, man will mich zum besseren Menschen machen, und ich war davon überzeugt, dass jeder in ganz [ANONYMISIERT, ORT IN DEUTSCHLAND], alle wissen, was ich denke, was ich tue, was ich mache, wer ich bin. (I: Hm (zustimmend)) Alles, äh ich habe mal in der Bahn gesessen, und da hat irgendeine Frau gehustet, dann hat es für mich "Ja" geheißen. (I: ah, ja) Also das hat eine Bedeutung (I: Ja.). Dann mor- musste ich anfangen zu lachen, weil ich mich wieder gefreut habe, weil dann habe ich gedacht, ich habe irgendeine Aufgabe bestanden. (I: Ja.) Und al- all so'ne Sachen, das ging drei vier Tage. Und denn hat sich das ein kleines bisschen gelegt, aber so weit gelegt, dass ich selber sagen konnte, ich gehe in die Klinik, also. (I: Okay) [GERÄUSCH VOM HANDY] Ja. Oh, Entschuldigung. |
| 238 | **I:** Gar kein Problem, das heißt Sie haben jetzt ähm das war jetzt, bevor Sie hierher gekommen sind, diesmal? |
| 239 | **AB04:** Nee, das war 2016. |
| 240 | **I:** Das war 2016, (AB04: Ja) da haben Sie selber entschieden, ich gehe in die Klinik? |
| 241 | **AB04:** Ja, (I: Okay) aber auch nur, ähm ich glaube auch da- dass ich hm das Glück hatte, weil es hat dann so ein Ausmaß genommen, (I: Hm (zustimmend)) wo ich selber wusste, das kann nicht real sein. (I: Okay) Also das wurde dann immer verrückter [AUTO HUPT] und immer komischer alles, (I: okay) ja. Und dann habe ich nachts auf einer Bank gesessen, dann hatte ich Halluzinationen und dann habe ich irgendwas von Atomkraftwerke erzählt, mir selber. (I: Hm (zustimmend)) Von Atomkraftwerke, ich weiß gar nicht wie ich darauf komm auf Atomkraftwerke, also, (I: Hm (zustimmend)) aa, das- das war aber der Punkt, wo ich dann wusste, wo- hm das ist alles nicht echt, da erkennt man also- ich war mir nicht sicher, ob die Leute mich ähm kennen, oder nicht, da war ich mir nicht sicher. Aber ich wusste das äh nicht, das auch irgendwie nicht sein kann, weil es kann ja nicht sein, [FAHRRAD KLINGELT] dass man jahrelang [FAHRRAD KLINGELT] äh lebt man normal und dann kennen einen plötzlich alle. (I: Ja.) Dann müssten das ja alle vorher verschwiegen haben, (I: Ja, ja.) oder so. Und so'ne Gedanken kamen dann, mh-mh recht normale Gedanken eigentlich, wo man sich- wo man hat halt das, was man erlebt hat, infrage gestellt hat. (I: Ahja.) Und deshalb- und deshalb- und das war dann ein Glück, äh grad so stark, dass ich gesagt habe, ich gehe in die Klinik, also. Ja. Hm. |
| 242 | **I:** Super. Gut. Ähm, okay. Und ähm. Die- Nochmal zurück zu den Gedanken, ne? Die- die Sie laut gehört haben, hatten Sie auch mal das Gefühl, dass Sie die, Ihre Gedanken, sehen können, wie in so einem Film oder (AB04: Mh-mh (verneinend) wie aufgeschrieben vor Ihrem inneren Auge? |
| 243 | **AB04:** Mh-mh (verneinend). (I: Nee, okay) Ich habe auch nie Stimmen gehört. (I: Nee?) Noch nie. (I: Noch nie.) Gott sei- Gott sei Dank, also das fehlt mir gerad noch. (I: Ja!) Ja, also das brauche ich nicht unbedingt. |
| 244 | **I:** Okay (AB04: Ja.) Das heißt, Sie haben nur Ihre eigenen Gedanken gehört (AB04: Mh-h (zögerlich zustimmend)). Und die waren- die waren sozusagen wie laut, oder so ähm zugänglich zu anderen Leuten- für andere Leute? |
| 245 | **AB04:** Hm, ja. |
| 246 | **I:** Ja. (AB04: Hm (zögerlich zustimmend). Das waren nicht fremde Stimmen, die Sie gehört haben? |
| 247 | **AB04:** Hm. [PAUSE] Äh ich ähm das ist- das äh das geht alles schon Jahre, ich habe schon alles erlebt. Da könnte ich hier auf die Frage Ja sagen, könnte ich Nein sagen, auf die nächste Frage Ja und Nein, kommt immer drauf an, mh wann das war. |
| 248 | **I:** I: Hm (zustimmend). (AB04: Ja.) Also, weil Sie- ich frage deshalb, weil Sie gesagt haben: "Ich habe nie Stimmen gehört." (AB04: Nee, habe ich nicht, nee) Jetzt ähm hatten wir vorher gesagt , Sie haben (AB04: Hm) aber Ihre eigenen Gedanken schon mal laut gehört, da- da habe ich einen Unterschied gemacht zwischen "eigene Gedanken laut gehört im Kopf" (AB04: Hm) und "Stimmen von fremden Leuten, fremde Stimmen gehört im Kopf". |
| 249 | **AB04:** Ja. (I: Und wenn Sie sagen-) Ja, gut, das- das kann ich ja be- bewusst aufrufen, dass können Sie aber auch. Wenn Sie jetzt zum Beispiel ein Familienmitglied oder so, wo man- oder eine Person, wo man gut kennt, wo man (I: Hm (zustimmend)) auch die Stimme kennt, (I: Hm (zustimmend)) kann man sich das ja vorstellen im Kopf. (I: Ja, das stimmt) Äh un- und denn ähm hat man das ja, was- was Sie halt sagen. |
| 250 | **I:** Ja. (AB04: Ja) Naja, dann hat man- Also, ich stell es mir so vor, ne? Dass man- dass das schon noch mal ein Unterschied ist, dass man, (AB04: Ja) wenn man sich selber entscheidet, dass das sich vorzustellen, die Stimme von meinem Vater jetzt zum Beispiel (AB04: Mh-mh) oder wenn ich die plötzlich einfach höre, ohne dass (AB04: Achso!) ich das wollte- |
| 251 | **AB04:** Nee, das habe ich nicht. |
| 252 | **I:** Das hatten Sie noch nicht? |
| 253 | **AB04:** Nee. |
| 254 | **I:** Okay. |
| 255 | **AB04:** Noch nicht, vielen Dank! [LACHT LAUT] Nee. |
| 256 | **I:** [LACHT] Grundsätzlich nicht. |
| 257 | **AB04:** Ja. |
| 258 | **I:** Ich hoffe, dass es auch nicht passiert. |
| 259 | **AB04:** Ja genau, ja. |
| 260 | **I:** Ähm, nur das haben einfach- das beschreiben einfach superviele Leute, ne? (AB04: Hm.) Ich meine, umso besser- |
| 261 | **AB04:** Ich habe- ich habe ein bisschen Angst davor, vor dem allem. Weil, wenn ich überlege, wo das alles begonnen hat, ähm das ähm-m mit den Jahren wurde das langganz- langsam schleichend, (I: Hm (zustimmend)) immer- immer ähm schlimmer. Und ich habe Angst, dass ich mit 40, 50 oder so total durchgedreht bin, oder so (I: Ja.) Oder in der Bahn laut, komische Sachen erzähle. (I: Okay) Völlig neben mir stehe, (I: Ja) davor habe ich Angst. |
| 262 | **I:** Das- das verstehe ich, dass Sie da (AB04: Ja) Angst haben (AB04: Ja), aber ich kann Ihnen sagen, so wie Sie- wie ich Sie jetzt erlebe, (AB04: Hm) wie Sie jetzt reden können darüber, (AB04: Ja) das ist ein toller Fortschritt, also (AB04: Ja) da habe ich viel positive Gefühle, was die Zukunft betrifft. |
| 263 | **AB04:** Danke. (I: Ja) Hm (zögerlich). |
| 264 | **I:** Okay. Dann kommen wir mal zurück, ähm zu meinen Fragen. Wie ist es, wenn Sie, da hatten wir kurz drüber gesprochen, wenn Sie abends im Bett liegen und all die Gedanken kommen? (AB04: Hm), "Grübeln" nennen wir das auch. (I: Grübeln) Kennen Sie den Begriff, ja? (AB04: Hm, ja [RÄUSPERT SICH]). Gibt es da dann auch Dinge, die Sie tun müssen, wenn Sie so grübeln? Irgendwelche Handlungen, die man dann machen muss? |
| 265 | **AB04:** Nö. |
| 266 | **I:** Nee. (AB04: mh-mh (verneinend)) Also Rituale, manche Leute machen so Rituale, oder irgendwas, was dann die Gedanken- was die Gedanken brauchen. So etwas gibt es nicht? |
| 267 | **AB04:** Hm nein. |
| 268 | **I:** Okay. Und ähm sind das Gedanken, wo Sie selber sagen würden, die sind unsinnig, oder irgendwie unnötig, die brauche ich eigentlich gar nicht? Und will ich gar nicht haben? |
| 269 | **AB04:** Ja, alles. (I: Ja?) Alles ist dabei. Also es sind- ich habe Gedanken, wo wichtig sind, wo unwichtig sind, w- wo lustig sind, wo traurig sind, wo- (I: Okay) ja also al- alles ist dabei. |
| 270 | **I:** Alles mögliche. (AB04: Ja) Und ähm sind da dann auch angstvolle Gedanken dabei? |
| 271 | **AB04:** Dass ich Angst habe? (I: Ja) Ja. |
| 272 | **I:** In dem Moment, ja? |
| 273 | **AB04:** Hm (zustimmend, kleinlaut). |
| 274 | **I:** Ähm, okay. Also es ist so ein Mischmasch an allen möglichen Sachen. (AB04: Ja) Hm. Oder sind da Gedanken dabei, die Sie- wo Sie das Gefühl haben, die wollen Sie eigentlich gar nicht? So etwas darf man gar nicht denken? |
| 275 | **AB04:** Ja (zögerlich). |
| 276 | **I:** Ja? |
| 277 | **AB04:** Hm (kleinlaut). |
| 278 | **I:** Haben Sie ein Beispiel dafür? Wäre das okay, wenn Sie da ein Beispiel- |
| 279 | **AB04:** Ähm. |
| 280 | **I:** Ich gucke hier einmal. [GERÄUSCHE IM RAUM] |
| 281 | **AB04:** Was- ähm- Ich muss mal kurz überlegen. Ich habe im- im- im Herbst letzten Jahr hatte ich irgendwie immer wieder den Gedanken, meine Mom unter Drogen zu setzen, um ihr mal zu zeigen, (I: Hm (zustimmend)) wie toll das ist. (I: Okay) Und eigentlich ist ja total schrecklich, die Idee. |
| 282 | **I:** Also Sie wollten- (AB04: Ja) Sie wollten das sozusagen, ohne dass sie davon weiß? |
| 283 | **AB04:** Nee-nee! (I: was war Ihr Gedanke?) Das mit ihr besprechen vorher und dann (I: Aha) mal etwas abgeben. (I: Okay) Ja. Aber nicht aus dem Grund, weil ich ihr irgendwie damit ähm schaden will, sondern (I: Hm (zustimmend)) einfach so mh. So. Eigentlich- eigentlich total schrecklich, der Gedanke! (laut) Ja. |
| 284 | **I:** Naja, wenn sie es nicht will, dann- dann schrecklich. |
| 285 | **AB04:** Naja, ach nee sie will es auf gar keinen Fall (I: Okay.). Aber, also das- da brauch ich gar nicht fragen. (I: Hm (zustimmend)) Habe ich auch nie gefragt. (I: Hm (zustimmend)) Aber so Gedanken, wie so- da denkt man dann so, "das sollte man nicht denken". |
| 286 | **I:** Okay. (AB04: Ja). Der kam immer wieder, ohne dass Sie ihn eigentlich denken wollten, diesen Gedanken? |
| 287 | **AB04:** Hm (zustimmend). |
| 288 | **I:** Hm, okay. [ATMET EIN] Hm. Und zur- zu den ähm Gedanken, Gefühlen: Hatten Sie schon mal das Gefühl, die sind irgendwie lokalisierbar, im Körper? Manche Leute sagen, meine Gedanken sind im Kopf, oder sind ich weiß nicht vielleicht in der rechten Schulter oder- |
| 289 | **AB04:** Nee, im Kopf. |
| 290 | **I:** Sie sind im Kopf? |
| 291 | **AB04:** Ja. |
| 292 | **I:** Und spüren Sie da Ihre Gedanken auch, oder sind die einfach da? Das ist klar, dass sie da sind? |
| 293 | **AB04:** [PAUSE] Hm. Ja also ich kann gar nicht sagen, ob ich das normal spüre, oder ob das schon äh unnormal ist. Weil das schon so lange her ist, dass ich nicht erkrankt war (I: Hm (zustimmend)), dass ich es vergessen hab, wie es eigentlich sein müsste. Und jetzt weiß ich gar nicht genau, ob ähm ist das so richtig, oder ist das nicht richtig? |
| 294 | **I:** Okay, aber wenn wir jetzt mal die Bewertung wegnehmen, wenn wir einfach fragen: "wie ist es denn jetzt gerade?" Also ob es jetzt richtig oder falsch ist, ist ja im Prinzip- jeder von uns ist anders, ne? (AB04: Hm (zustimmend)) Man spürt das anders. (AB04: Ja) Es geht ja mehr darum, ob das für Sie unangenehm ist. Oder ob es für Sie irgendwie schwierig ist, oder es Ihnen auffällt, oder so. |
| 295 | **AB04:** Ja, auch. Also kann man auch sagen alles. (I: Ja?) Also ma- manche Sachen sind schwierig, manche sind ängstlich, manche sind glücklich. (I: Mh-mh?) Ja. |
| 296 | **I:** Okay, und wenn wir jetzt nochmal zurück gehen zur Frage, ob Sie Ihre Gedanken irgendwie im Körper verorten, oder spüren, oder so können, (AB04: mh-mh (verneinend)) dann würden Sie Nein sagen? |
| 297 | **AB04:** Nein. |
| 298 | **I:** Nein. Oder im Raum auch? |
| 299 | **AB04:** Ich habe halt- ich habe manchmal so ähm so zum Beispiel- zum Beispiel ich sitze so und gucke Fernsehen, dann denk ich mir irgendwas, weiß ich nicht, "die Tür ist grau", und dann macht mein Finger so (I: okay.) und denn äh ist dieser Satz mh in Verbindung mit dem Finger. Und wenn nächstes Mal der Finger geht, denn "denk an den Satz". |
| 300 | **I:** Okay, also (AB04: Ja.) Gedanken sind verbunden mit körperlichen Bewegungen? |
| 301 | **AB04:** Hm, ja. |
| 302 | **I:** Mh-hm. Und- also- das ist immer so, ja? Da gibt es dann immer wieder Verbindungen? |
| 303 | **AB04:** Immer wieder neue. |
| 304 | **I:** Immer wieder neue? |
| 305 | **AB04:** Ja. So- immer wo die Psychose sehr stark war 2016, da war es häuptsächlich das Husten als "Nein", und das [ATMET DURCH DIE NASE SCHARF EIN] Nase Hochziehen als "Ja". |
| 306 | **I:** Okay. (AB04: Mhh.) Bei Ihnen selber auch? Also wenn Sie selber die Nase- |
| 307 | **AB04:** Nee bei mir selber nich, nur wenn andere das machen. |
| 308 | **I:** Wenn andere das gemacht haben. |
| 309 | **AB04:** Mh, ja. |
| 310 | **I:** Okay. [PAUSE] Ja. [GERÄUSCHE IM RAUM, GESPRÄCH WIRD UNTERBROCHEN] Hallo, sorry, wir sind noch im Gespräch, hat später angefangen. Ähm wir haben erst um- vor einer halben Stunde, ne? Haben wir angefangen. In einer Stunde vielleicht oder nochmal halbe Stunde. Am Montag, ja? Okay. Ciao. [GESPRÄCH WIRD FORTGESETZT] Mein Kollege! Der arbeitet eigentlich hier an dem Platz, aber wenn wir Interviews machen dann hat immer einer- |
| 311 | **AB04:** Ah, der arbeitet hier! Hätte ich jetzt nicht gedacht. |
| 312 | **I:** Doch, arbeitet hier. Genau. Ähm und sonst machen wir ähm den Raum für einen von uns- |
| 313 | **AB04:** Hm, ja okay. |
| 314 | **I:** Okay, wo waren wir? Okay. Ähm wie ist es denn, wenn Sie Entscheidungen treffen müssen? Ganz einfache Entscheidungen, wie ich gehe in den Supermarkt und ähm wähle aus, was ich einkaufen will. (AB04: Hm) Ist das schwierig, oder? |
| 315 | **AB04:** Nein. |
| 316 | **I:** Nein, das ist ganz leicht |
| 317 | **AB04:** Ja. |
| 318 | **I:** Auch was ich morgens anziehe? |
| 319 | **AB04:** Ich äh kann ich ähm schneller als früher. |
| 320 | **I:** Ja? (AB04: Ja.) Super. Gut. Äh auch was so zum Anziehen- egal da was Sie- was Sie da aussuchen- |
| 321 | **AB04:** Nö, ist mir nicht egal, also suche ich auch aus. |
| 322 | **I:** Ja? (AB04: Ja.) Und dann da fällt es Ihnen aber auch leicht? |
| 323 | **AB04:** Ja. |
| 324 | **I:** Okay. Und ähm gab es Situationen, wo Sie nicht so richtig wussten, ob Sie Sachen erinnern oder ob die wirklich passiert sind, oder was ein Traum war oder wirklich passiert ist? |
| 325 | **AB04:** Ja, stimmt. |
| 326 | **I:** Ja? Können Sie mir da ein Beispiel nennen? |
| 327 | **AB04:** Nein, leider nicht. (I: Fällt Ih-) Ich weiß, dass ich schon mal erlebt habe, aber ich weiß nicht mehr ähm wie das war, genau. |
| 328 | **I:** Okay. Und das ist jetzt auch nichts, was häufiger auftritt? Also ist nur so ein paar Mal? |
| 329 | **AB04:** Nö! Selten. |
| 330 | **I:** Selten, okay. Und ähm haben Sie manchmal Schwierigkeiten, Ihr Denken oder Handeln in Gang zu bringen? Also dass Sie dann irgendwie denken, ich will was tun oder ich will jetzt was machen, und das klappt nicht so richtig? |
| 331 | **AB04:** Also die Erfahrung habe ich immer wieder im Leben gemacht. |
| 332 | **I:** Ja? |
| 333 | **AB04:** Hm. |
| 334 | **I:** Können Sie mir das beschreiben? |
| 335 | **AB04:** Ja also eigentlich will ich nur das ganz normale Ding, aber ich scheiter immer (kleinlaut). |
| 336 | **I:** Okay. Was- worauf beziehen Sie das? |
| 337 | **AB04:** Mh. Familie, Arbeit, mh. Halbwegs Gesundheit. (I: Ja.) Keine Drogen, alles so das ganz normale Ding halt. (I: Ja.) Ja, aber ich- aber halt bei dem Versuch, das äh umzusetzen, scheitere ich seit Jahren immer wieder. |
| 338 | **I:** Okay. (AB04: Ja.) Und ähm liegt das dann daran, dass Sie sich vornehmen, was zu tun, also heute morgen stehe ich auf zum Beispiel, ganz früh (AB04: Mh) und dann machen Sie es nicht, und dann klappt es nicht? Oder- |
| 339 | **AB04:** Ja, genau, ja auch, ja. Hm. |
| 340 | **I:** Oder wenn Sie sagen, ich scheitere, was meinen Sie damit? |
| 341 | **AB04:** Ja meistens ist es wegen Drogen gewesen, immer (I: Ja.) wegen der Sucht. (I: Ja.) Dann hat man irgendwann wieder- da kam der Punkt, da hat man wieder angefangen, und dann ist wieder alles den Bach runtergegangen. |
| 342 | **I:** Ja, okay. (AB04: Ja.) Gut, das ist eine Sache jetzt. (AB04: Ja) Dass- Drogen (AB04: [RÄUSPERT SICH]) beeinflussen einen sehr, ne? Das- das (AB04: Ja) nehmen wir jetzt mal weg (AB04: Okay) und ähm einfach in Zeiten, wo Sie keine Drogen genommen haben, jetzt (AB04: Mh) zum Beispiel, die letzten sechs Monate, (AB04: Ja) ähm, mh, gibt es da Situationen, ganz einfache Situationen, wo Sie sagen: "Ich will jetzt etwas tun" und dann klappt das nicht? |
| 343 | **AB04:** Hm ja ich habe mich gestern ziemlich gefreut, dass- also ich war ziemlich überrascht, ich muss ganz ehrlich sagen, das ist überhaupt nicht böse gemeint, (I: alles gut) aber drüben in der Klinik bei [ANONYMISIERT, NAME EINER ÖRTLICHEN PSYCHIATRISCHEN STATION], da ist alles so- das Gebäude an sich, ist alles so neu und so chic und alles automatisch und so (I: Hm (zustimmend)) und- und hier ist ja überhaupt nicht so (I: Nee) und da habe ich erst gedacht so: "Oh, [ANONYMISIERT, NAME EINER ÖRTLICHEN PSYCHIATRISCHEN STATION]?" Und dann weiß ich nicht. Das- das hat mir irgendwie mein Denken und dann über die Tagesklinik ähm verschlechtert aber ich war relativ ähm überrascht, also habe mich gefreut über das Angebot, wa- was es hier gibt. Un- und habe mich eigentlich gefreut, heute wieder herzukommen. (I: Ja.) Und dann habe ich erstmal verschlafen (entrüstet)! Zwei Stunden! (I: Ja.) Ich musste sogar geweckt werden! (I: Ja.) Und das ist dann wieder so ein Ding, so, ich scheitere immer irgendwie. Äh ist irgendwas. Ich habe denn- bin gestern Abend eingeschlafen, ich habe nicht mal geschafft, meine Nachtmedis zu nehmen. Mh-mh. |
| 344 | **I:** Okay. Und warum waren Sie so müde? Was- was ist da- |
| 345 | **AB04:** Wegen [ANONYMISIERT, NAME EINES MEDIKAMENTS]. |
| 346 | **I:** Und wegen dem Clozapin? |
| 347 | **AB04:** Ja. |
| 348 | **I:** Hm. Okay. |
| 349 | **AB04:** Wo ich es nicht genommen habe, war ich nicht müde. Normalerweis bin ich Frühaufsteher. (I: Okay) Ja. |
| 350 | **I:** Ja. Aber hilft Ihnen das Clozapin sonst? |
| 351 | **AB04:** Ja, schon. (I: Ja?) Ja. (I: Ja.) Ja. Wenn es nicht helfen würde, würde ich es nicht nehmen, (I: Hm (zustimmend)) weil die Nebenwirkungen sind so stark, da nimmt man echt etwas in Kauf. Beim letzten Mal habe ich 60kg zugenommen. Und war eine Schlaftablette vom Feinsten! |
| 352 | **I:** [LACHT] Okay. Schlaftablette. Klingt- klingt lustig. (AB04: Hm) Aber Sie- Sie ähm. Letztes Mal haben Sie auch schon Clozapin genommen? |
| 353 | **AB04:** Ja, genau, ja. (I: Hm (zustimmend)) Von 2016 bis ähm 18, so. |
| 354 | **I:** Okay. Okay. Und es scheint zu- was zu bringen, das Clozapin? |
| 355 | **AB04:** Ja, (I: Ja?) ja, bringt schon etwas, (I: Ja?) hm. |
| 356 | **I:** Vor allem gegen die Angst wahrscheinlich? |
| 357 | **AB04:** Ja, ja. Das Lyrica hat überhaupt nicht geholfen. Ich habe noch Lyrica gekriegt. Da hat der Oberarzt gesagt, das ist gegen Angstzustände. Gegen Angst. Ähm und hat auch nichts- hat gar nichts gebracht- (I: Okay.) Ja. Aber ich kriege es komischerweise trotzdem weiter, weil ich wollt nix sagen- |
| 358 | **I:** Okay. Ja, das müssten Sie mit den Ärzten besprechen. (AB04: Ja) Weil es ist auch gut, wenn Sie was sagen, weil nicht dass Sie- dass Sie die Sachen einfach weglassen. (AB04: Hm) weil das ist das Schlimmste. |
| 359 | **AB04:** Nee, ich hab- ich hab es nicht ähm bewusst weggelassen, ich bin eigentlich nur eingeschlafen. (I: Ja.) Wäre ich wach gewesen, hätte ich ja auch meine Medis genommen. Also ich verweigere das jetzt nicht, die Einnahme. |
| 360 | **I:** Ja, ja. (AB04: Ja) Das glaube ich Ihnen sofort. Nur- nur wenn Sie sagen, das bringt mir eigentlich nichts. Und dann in ein paar Monaten, lassen Sie es einfach weg, das wär blöd. Deswegen reden Sie dann lieber mit Ihren Ärzten darüber. |
| 361 | **AB04:** Ja, auf jeden Fall, ja. Ich habe äh wo ich auf [ANONYMISIERT, NAME EINER ÖRTLICHEN PSYCHIATRISCHEN STATION] gekommen bin, gleich am Anfang an, geäu-geäußert, weil- weil ich- mein Körper äh reagiert nochmal sehr, sehr stark auf die Nebenwirkungen von der Leponex im Gegensatz zu anderem (I: Hm (zustimmend)). Weil ich ähm, wie ich schon sagte, ich habe es ja- ich hatte in eineinhalb Jahren 60 Kilo zugenommen (I: Ja.) und habe den ganzen Tag nur geschlafen. Und dann hat man das so gelöst, dass man mir hat nu- nur knapp unter 100 Milligramm Clozapin pro Tag äh jetzt aktuell gegeben und äh hat das mit einem anderen Medikament, mh das steigt den Leponex-Spiegel in die Höhe (I: Ja.) un- und dass Nebenwirkungen dann nicht ganz so stark sind. Hat ein bisschen funktioniert, aber ich merke es trotzdem noch heftig. |
| 362 | **I:** Okay. Ja. wird sich vielleicht auch noch einpendeln, ne? Ist jetzt (AB04: Jo) noch nicht so lange her. |
| 363 | **AB04:** Jo ist (I: Ja.) gerade mal zwei Wochen her, wo ich fest ähm feste Medikation. |
| 364 | **I:** Genau. Okay. Über Aufmerksamkeit haben wir schon gerade kurz gesprochen, ne? Sie haben gesagt, ähm nee über Gedächtnis haben wir gesprchen, da haben Sie gesagt, Sie können sich an den Anfang des Satzes nicht mehr erinnern, genau. [GLAS WIRD AUF TISCH GESTELLT] Und wie ist es mit der Aufmerksamkeit? Das gibt so Situationen, wo- wenn zu viele Sachen passieren, dass Sie sich dann nicht mehr auf eine Sache konzentrieren können, also, wir führen ein Gespräch, daneben würde gleichzeitig noch Radio laufen. Würde das stören, oder würde das gehen? |
| 365 | **AB04:** Äh nee. Ich kann alles gleichzeitig wahrnehmen. |
| 366 | **I:** Ja? (AB04: Ja) Das ist kein Problem mit der Aufmerksamkeit? Ist auch nicht überfordernd, dann? |
| 367 | **AB04:** Doch, es ist überfordernd. (kleinlaut) (I: Ja?) Ja. |
| 368 | **I:** Fühl- fühl- wie fühlt sich das an? |
| 369 | **AB04:** Ja, wenn ich durch die Stadt laufe, dann- dann hupt es da und da und dann äh läuft hier und da jemand an mir an mir vorbei, der redet gerad zum Beispiel ähm über seine Gesundheit und dann verstehe ich nur drei, vier Worte, und dann überlege ich was äh w- w- wie- was [SIRENE IM HINTERGRUND] ist der Rest vom Satz, und dann hupt es wieder da. Und s- ich nehm- ich nehm alles wahr. Äh- |
| 370 | **I:** Okay. (AB04: Ja) Und das ist- wie fühlt sich das an, ist das überfordernd dann? |
| 371 | **AB04:** Ja, (I: Ja?) hm. |
| 372 | **I:** Unangenehm? |
| 373 | **AB04:** Ja. |
| 374 | **I:** Okay. Und das ist heute auch noch so? |
| 375 | **AB04:** Ja. |
| 376 | **I:** Okay. Hm. |
| 377 | **AB04:** Das habe ich auch jetzt gerade, ich hör schon die ganze Zeit, was draußen los ist. (I: Ja?) Ja. Ich habe mich nu, schon dran gewöhnt. (I: Okay.) Und ich sa- und ich sag auch nix. Also das äh ich schluck das alles und deswegen merkt man mir es auch nicht so an. |
| 378 | **I:** Okay. (AB04: Ja) Hm. Das heißt, die- die Geräusch- ähm am besten wäre es eigentlich, es wär ganz ruhig hier drin? |
| 379 | **AB04:** Mh ja. (I: Das wär am einfachsten für Sie?). Am besten ist immer, wenn ich Musik höre, weil- (I: Ahja?) ja. |
| 380 | **I:** Beschreiben Sie mal, was passiert dann? |
| 381 | **AB04:** Dann höre ich nichts mehr, außer die Musik (I: Dann machen Sie Kopfhörer rein, und dann nur die Musik?) hm nur die Musik. |
| 382 | **I:** Und das ist angenehm? |
| 383 | **AB04:** Ja. (I: Schön) Wenn man keine Ängste hat! (I: Okay.) Wenn ich Ängste habe, dann kann ich keine Kopfhörer hören, dann höre ich ja nicht- wenn mich von hinten jemand angreift oder so. |
| 384 | **I:** Verstehe (AB04: Ja) Okay, okay. Und wie ist es gerade? Jetzt gerade würde- also jetzt in den letzten Wochen, (AB04: Hm) würde Ihnen Musik- ist es angenehm, dass Sie Kopfhörer reinmachen und nur die Musik hören? |
| 385 | **AB04:** Hm joa es hat sich eigentlich normalisiert. (I: Ahja) Also ich höre eigentlich nur Musik au- auf der Straße, wenn ich Lust habe auf Musik und nicht wegen den Geräuschen (I: Okay) ähm zu unterdrücken. |
| 386 | **I:** Okay. Das haben Sie aber schon auch gemacht mal, dass Sie- |
| 387 | **AB04:** Ja, ja! Jetzt aktuell ja auch! Am Anfang, wo ich rausgegangen bin von [ANONYMISIERT, NAME EINER ÖRTLICHEN PSYCHIATRISCHEN STATION] immer nur mit Musik. |
| 388 | **I:** Alles klar. (AB04: Ja) Okay, verstehe. Hm. Und kann es auch passieren, dass äh Dinge in der Umgebung Ihre Aufmerksamkeit besonders auf sich ziehen? Irgendwelche Lichter? Oder- |
| 389 | **AB04:** Autokennzeichen. |
| 390 | **I:** Autokennzeichen? (AB04: Hm) Warum Autokennzeichen? |
| 391 | **AB04:** Das weiß ich ehrlich gesagt selber noch nicht so genau (kleinlaut). [PAUSE] Hm. |
| 392 | **I:** Da achten Sie- |
| 393 | **AB04:** Kann- kann ich- Kann ich Ihnen nicht sagen, aber ich achte da total drauf. Ja, aber ich weiß nicht, wieso. Und ich weiß auch nicht wie das äh woher das kommt. |
| 394 | **I:** Hm (zustimmend). Was passiert dann in Ihnen, wenn- wenn so ein Autokennzeichen vorbei- Ihnen auffällt, oder vorbeifährt? |
| 395 | **AB04:** Hm ja einzelne Kennzeichen haben eine Bedeutung. (I: Ahja?) Ja. |
| 396 | **I:** Was für Bedeutungen? |
| 397 | **AB04:** Hm. [PAUSE]. Hm. Naa. Zum Beispiel "PS", das ähm ist von [ANONYMISIERT, ORT IN DEUTSCHLAND] (I: Hm (zustimmend)), [ANONYMISIERT, LANDKREIS IN DEUTSCHLAND], da wohnt mein [ANONYMISIERT, ANGEHÖRIGER DER INTERVIEWTEN PERSON]. Das ist immer ähm erleichternd. Und ähm dann äh frö- äh ein fröhliches Kennzeichen. |
| 398 | **I:** Okay, okay. (AB04: Ja) Also das macht richtig etwas mit Ihnen (AB04: Ja) emotional? (AB04: Ja) Alles klar. Hm. Spüren Sie das richtig? |
| 399 | **AB04:** Hm, (I: So leicht) ja. Man- man denkt halt noch darüber nach, (I: Ja.) so, ja. |
| 400 | **I:** Okay. Okay. [PAUSE] Und wie ist es mit Zeit? Wie erleben Sie Zeit? Gibt es da irgendwie besondere Erlebnisse, dass die Zeit- |
| 401 | **AB04:** Eigentlich normal. |
| 402 | **I:** Normal? (AB04: Ja) Die passt so, die wird nicht mal schneller, oder plötzlich schneller, oder langsamer? |
| 403 | **AB04:** Nur unter Drogen. |
| 404 | **I:** Nur unter Drogen. |
| 405 | **AB04:** Ja. |
| 406 | **I:** Okay. Oder, dass Sie manchmal das Gefühl haben, es gibt nur das Hier und Jetzt, und nichts früher und nichts morgen? |
| 407 | **AB04:** Nein, habe ich nicht. |
| 408 | **I:** Nein. [PAUSE] Gab es schon mal ähm kleine Lücken oder Brüche im Bewusstsein, dass Sie nicht mehr so richtig wussten, wie Sie irgendwo hingekommen sind? Oder- (AB04: Mh ja (zögerlich)) ohne dass Sie Drogen genommen haben? |
| 409 | **AB04:** Achso! Ja, bei Alkohol passiert mir das oft. |
| 410 | **I:** Hm ja, da ist das, ja. Filmriss- den kennen wir. |
| 411 | **AB04:** Hm, ja genau (unverständlich). |
| 412 | **I:** Aber ohne dass Sie Drogen genommen haben? |
| 413 | **AB04:** Mh-mh (verneinend) [SIRENE IM HINTERGRUND] (I: Nein?) Habe ich eigentlich nich, ne. |
| 414 | **I:** Okay. Und wie ist es, wenn wir uns jetzt so unterhalten? Haben Sie da das Gefühl, Sie können das ausdrücken, was Sie (AB04: Äh) sagen wollen? |
| 415 | **AB04:** Ja ich- ich denke, ich kriege das ganz gut hin (I: Ja?), ja. Ich- ich fühl mich sehr wohl gerade in dem Gespräch, (I: Schön, das ist schön) ja. Weil es irgendwie ähm ma- man redet mal ein bisschen genauer und alles hm und das ist äh das nimmt mir sehr leicht die Arbeit ab, dass Sie immer fragen. (I: Hm (zustimmend)) Und da- dass ich nicht von mir aus erzählen muss und das ist- ist ähm sehr angenehm. |
| 416 | **I:** Okay. (AB04: Hm) Und haben Sie dann das Gefühl, das, was Sie sagen, passt zu dem, was- was ähm Sie sagen wollen? Also was (AB04: Ja, genau) Sie innerlich empfinden? (AB04: Ja, auch) Ja? (AB04: Hm (zustimmend)) Gut, also die Worte sind irgendwie- passen richtig und- |
| 417 | **AB04:** Ja, ich kann mich gut ausdrücken. (I: Okay, gut) Gut artikulieren (SPRICHT ÜBERTRIEBEN GENAU UND LANGSAM). |
| 418 | **I:** Artikulieren [SPRICHT EBENSO ÜBERTRIEBEN GENAU] [LACHT] Gut. Okay. Dann ähm kommen wir jetzt zu einem Bereich, der geht so ein bisschen um Ihr- das Gefühl, man selber zu sein, oder da zu sein, anwesend zu sein (AB04: Hm) in der Welt. Fällt Ihnen da was zu ein? |
| 419 | **AB04:** Hm. [PAUSE]. (I: Gibt es da irgendein besonderes Gefühl?) [PAUSE] Also ich denke schon, dass ich anwesend bin (I: Hm (zustimmend)) in der Welt, (I: Hm (zustimmend)) ja. Ich- ich denke schon halt nur, dass ich ein bisschen abgegrenzt bin. Äh mein Vater wohnt [ANONYMISIERT, KILOMETERANGABE] Kilometer weit weg. (I: Okay.) Und da ist noch meine Stiefmutter und die beiden Stie- äh Stiefgeschwister und der g- mh- und der ganze Rest ist [ANONYMISIERT, KILOMETERANGABE] Kilometer weit weg. (I: Ja.) Und [ANONYMISIERT, NAME DER PARTNERIN DER INTERVIEWTEN PERSON] ist jetzt noch in [ANONYMISIERT, ORT IN DEUTSCHLAND] ist ja auch nochmal [ANONYMISIERT, KILOMETERANGABE] Kilometer weg und ähm ist niemand mehr übrig. Denke ich halt, dass ich da ein bisschen, na, am falschen Ort bin, vielleicht. (I: Hm (zustimmend)) Hm. |
| 420 | **I:** Okay, also Sie fühlen sich einsam, so ein bisschen? |
| 421 | **AB04:** Manchmal, ja. (I: Ja?) Ja, stimmt, manchmal habe ich extrem das Gefühl. Hatte ich auch letztens erst. Da habe ich so bei- da habe ich so bei- mei- meinem Zimmer gesessen. Und da- und da bin- da bin ich auch von einer Tagesklinik, von [ANONYMISIERT, NAME EINER ÖRTLICHEN PSYCHIATRISCHEN STATION] gekommen. Und da habe ich nicht mehr den Fernseher angemacht. Sondern ich habe in meinem Zimmer gesessen, habe gedacht, so eigentlich bin ich- m- bin ich ja voll einsam, so. (I: Hm (zustimmend)) Ja. Also ich bin normal nicht der Mensch, wo jetzt- wo- wo jetzt auch auf die Tränendrüse drücken muss, (I: Ja.) aber das ist ähm ja eigentlich, mh, muss man das auch schon mal sagen, so. (I: Okay.) Ja. |
| 422 | **I:** Und ähm. Gibt es da Pläne, vielleicht? Mit [ANONYMISIERT, NAME DER PARTNERIN DER INTERVIEWTEN PERSON]? |
| 423 | **AB04:** Ja klar (I: Ja?) gibt es Pläne. (I: Ja.) Ja. Es gibt immer wieder Pläne. Schon seit Jahren. Das normale Leben. Aber ich scheitere aber immer. (I: Hm (zustimmend)) Ja. Hoffentlich dieses Mal nicht wieder. |
| 424 | **I:** Ja. (AB04: Ja.) Und dann wäre der Plan, dass [ANONYMISIERT, NAME DER PARTNERIN DER INTERVIEWTEN PERSON] zu Ihnen kommt? Oder was ist der Plan? |
| 425 | **AB04:** Äh nee sie ist in einem Mutter-Kind-Heim bei [ANONYMISIERT, ORT IN DEUTSCHLAND] für neun Monate. (I: Okay.) Und nach dem neunten muss sie sich eine Wohnung suchen. Und mit mir zusammen, logischerweise. |
| 426 | **I:** Mit Ihnen zusammen? |
| 427 | **AB04:** Ja, ja, ich helfe auch mit Wohnungssuche. |
| 428 | **I:** Ja. Also Sie wollen dann zusammen wohnen, auch? |
| 429 | **AB04:** Ja. (I: Ja?) Hm. |
| 430 | **I:** Okay. Weil das ist ja ein Plan, der so ein bisschen die Einsamkeit dann verändert. |
| 431 | **AB04:** Ja, genau. (I: Ja.) Es gibt für alles ne- für alles ähm hm für alles Negatives äh gibt es ja auch einen positiven Plan um das ähm zu verändern so. (I: Ja.) Mein Problem ist aber das umzusetzen. (I: Ja.) Ja. Ich habe eigentlich gute Ideen immer. (I: Okay.) Ja. Und bin ich auch sehr von überzeugt, dass es auch so klappt. Wenn man halt immer morgens aufsteht, und das macht, und das macht, (I: Hm (zustimmend)) hm. Habe ich eigentlich ein sehr normales Denken. Und zum Beispiel mei- mein Mitbewohner, hier der [ANONYMISIERT, NAME EINES MITBEWOHNERS], wo bei mir wohnt, der hat zu mir gesagt: "Mein Leben"- der ist 28, (I: Hm (zustimmend)) er hat gesagt äh: "Arbeiten, gehen, nö. Mein Leben ist gegessen so, für mich ist das alles nichts mehr" und da habe ich zu ihm gesagt: "Arbeiten gehen ist das mh- mh erste halbe Jahre anstrengend, wenn man es nicht gewohnt ist, und dann ist man drin im Trott (I: Ja.) und dann hat man aber auch etwas davon, (I: Ja.) nicht nur Geld", habe ich zu ihm gesagt. Und das sind ja alles Sachen, wo- wo- wo wirklich so sind. (I: Ja.) Da bin ich der Überzeugung, dass es eigentlich die richtige Einstellung. Aber das dann auch so umzusetzen, das ist- da scheitere ich auch immer dran, ja. |
| 432 | **I:** Das ist eine- eigentlich eine perfekte Aufgabe für eine Psychotherapie, ne? (AB04: Ja.) So- so dieses an dem Umsetzen zu arbeiten. |
| 433 | **AB04:** Ja, genau, ja, hm. (I: Und das machen die-) Find es total blöd, dass ich heute zu spät gekommen bin, wei- äh (I: Das ist-) vielleicht ist es- vielleicht ist es jetzt hier für die Therapeuten oder so, äh vielleicht drücken da alle jetzt nochmal ein Auge zu. Aber, hm an dem Abend darf ich es gar nicht erzählen, die wäre wieder total enttäuscht. (I: Hm (zustimmend)) Die würde schon wieder denken, die Welt geht unter. (I: Hm (zustimmend)) Ja. Also, das kann- gerade mal der zweite Tag heute. |
| 434 | **I:** Wer ist denn Ihr Psychotherapeut? Ist es Herr- |
| 435 | **AB04:** Ich wei- ich weiß nicht, wie sie heißen. Ich kann mir so schlecht die Namen merken. Ich war 13 Wochen auf [ANONYMISIERT, NAME EINER ÖRTLICHEN PSYCHIATRISCHEN STATION] und habe mir nicht einen Name vom Pfleger merken können. |
| 436 | **I:** Okay. (AB04: Ja.) Aber vielleicht entschuldigen Sie sich da einfach da nochmal, für das Zuspätkommen, so wie bei mir und dann- |
| 437 | **AB04:** Ja, habe ich schon gemacht bei der Blutentnahme. |
| 438 | **I:** Genau, dann wird es dem schon leicht fallen, (AB04: Ja) Ihnen zu verzeihen (AB04: Ja) und dann mit einer Psychotherapie anzufangen (AB04: Genau, ja, hm) , denke ich, ja. Und ähm Sie haben gesagt, Sie fühlen sich in der Welt anwesend (AB04: Ja [RÄUSPERT SICH]) und ähm haben Sie manchmal das Gefühl gehabt, irgendwie anders zu sein, als andere Menschen? |
| 439 | **AB04:** Ja. |
| 440 | **I:** Ja? |
| 441 | **AB04:** Hm (zustimmend). |
| 442 | **I:** Wann hatten Sie das Gefühl, zum Beispiel? |
| 443 | **AB04:** Na, ich bin Suchtkrank (kleinlaut). Ist wie- das sind die meisten Leute nicht (I: Okay.) Und das ist ein großer Unterschied. (I: ist ein großer Unterschied) M- mein Denken und mein Handeln äh in- ist sehr- ist anders als bei Leuten, wo nicht so krank sind. (I: Okay.) Ja, wenn zum Beispiel, Sie- ich mach jetzt mal ein Beispiel ähm mh wir beide sind jetzt Hartz-4 Empfänger (I: Hm (zustimmend)) un- und es ist der 20. im Monat und nur noch 10 äh Tage noch und man hat- man hat 30 Euro. Sie würden es für Essen oder so ausgeben (I: Hm (zustimmend)) und nicht für Drogen. (I: Okay.) Ja und das ist dann halt immer die falsche Entscheidung, so etwas. (I: Okay.) Da- da grenze ich mich halt ab, von der- von dem normalen Leben, (I: Ja.) wo ich eigentlich hin will, ja. |
| 444 | **I:** Und, ähm. Wenn Sie das nochmal- wenn Sie die Suchtproblematik wegnehmen, (AB04: Hm (zustimmend)) haben Sie dann immer noch das Gefühl- sagen wir mal, die- die nehmen (AB04: Ah) wir jetzt in die Hand (AB04: Hm (zustimmend)) und stellen sie nach hinten. (AB04: Okay) Ähm haben Sie dann immer noch das Gefühl- haben Sie da mal das Gefühl gehabt, sich anders zu fühlen? |
| 445 | **AB04:** Hm na auf jeden Fall. (I: Ja?) Ich bin schizophren. |
| 446 | **I:** Okay (AB04: Hm (zustimmend)) und was- was- was macht Sie da anders? [PAUSE] Abgesehen von der- dass Sie manchmal ähm- |
| 447 | **AB04:** Ich habe ähm (I: Angst) mit meine Gedanken keine Ruhe. (I: Hm (zustimmend)) Ja. (I: Okay.) Viele Menschen, denke ich, können auch mal entspannen, einfach mal kurz an gar nichts denken oder an nur leichte Sachen, so. (I: Hm (zustimmend)) Wo mal ein bisschen vorbeifliegen, sage ich jetzt mal. (I: Ja.) Ähm ja und bei mir ist immer, [MACHT LAUTE ELEKTRISCHE GERÄUSCHE] ja. |
| 448 | **I:** Im Kopf? |
| 449 | **AB04:** Ja, genau. |
| 450 | **I:** Alles klar, alles klar. Und ähm das ist schon immer so, oder also auch schon als Kind? oder- |
| 451 | **AB04:** Mh-Mh (verneinend). (I: Nee?) Also ne das hm das ging los mit der Erkrankung. |
| 452 | **I:** Also mit 17, 18 haben Sie gesagt, hat sich da was verändert? |
| 453 | **AB04:** Hm ja, genau, ja. |
| 454 | **I:** Okay. [PAUSE] Hm. Oder hatten Sie mal das Gefühl, irgendwie, wie so eine Rolle zu spielen? Auf der Bühne? Als wären das gar nicht Sie selbst? Als würden- als wüssten Sie gar nicht so sehr, was Ihre Identität ist? |
| 455 | **AB04:** Das weiß ich. |
| 456 | **I:** Das wissen Sie? |
| 457 | **AB04:** Ja (I: Ja.). Ich weiß, wer ich bin, wo ich herkomme, Familie, so, oder mh. |
| 458 | **I:** Wo Sie hinwollen? |
| 459 | **AB04:** Mh, wo ich hin will. |
| 460 | **I:** Ja? Okay. Also Sie haben eine feste Identität. (AB04: Ja) Die steht nicht infrage? |
| 461 | **AB04:** Mh-mh (verneinend). |
| 462 | **I:** Okay. Und ich habe Sie so etwas Ähnliches schon mal gefragt, ähm vorhin. Hatten- haben Sie mal das Gefühl, Ihre Gedanken, Handlungen, Gefühle gehören zu Ihnen? Oder kann es auch mal sein, dass sie Ihnen fremd vorkommen? |
| 463 | **AB04:** Ja, beides, hm. (I: Ja?) Beides schon erlebt. |
| 464 | **I:** Beides schon erlebt (AB04: Ja). Und wie ist es jetzt zum Beispiel, also nach der letzten Phase? |
| 465 | **AB04:** Nach welcher letzten Phase? |
| 466 | **I:** Der letzten akuten Phase in der Klinik. |
| 467 | **AB04:** Achso. |
| 468 | **I:** Jetzt, wo es Ihnen eigentlich besser geht, wie ist es da? |
| 469 | **AB04:** Hm ja es ist n- es ist nicht ganz weg. (I: Also Sie haben immer noch-) Es ge- es geht niemals weg. |
| 470 | **I:** Okay. (AB04: Ja) Dieses Gefühl, dass manche Handlungen, Gefühle, Gedanken, nicht zu Ihnen gehören, das geht nicht weg? |
| 471 | **AB04:** Ja. Mh im Allgemeinen die Schizophrenie- (I: Hm (zustimmend)) das ge- das geht niemals weg. (I: Hm (zustimmend)) Man kann es nur eindämmen, irgendwie und mh- mh und halbwegs geordnet haben im Kopf, sag ich jetzt mal, (I: Ja.) mh. Und ansonsten, ich glaub nicht, dass das jemals ein Ende nimmt (kleinlaut). Hm. |
| 472 | **I:** Also Sie lernen damit umzugehen (AB04: Ja, genau). Das ist immer da, dieses Gefühl, (AB04: Ja.) dass manche Sachen nicht zu einem selber gehören, ja? |
| 473 | **AB04:** Ja. Ich versuch mein normales Verhalten aufrecht zu erhalten, sodass die Leute nicht merken, dass ich krank bin. |
| 474 | **I:** Okay? Und wie- wie. Okay. Also jemand, der Sie nicht kennt, nach Ihrer Meinung, darf das nicht merken? |
| 475 | **AB04:** Hm ja auch Leute, wo mich kennen, merken das nicht. (I: Okay.) Ja. (I: Also so-) Meine Mom war- war im April da, die hat gesagt: "Warum musst denn du in die Klinik, du bist doch ganz normal." Also, (I: Eigentlich ein Kompliment, ne?) ja. Das hat mich schon ein bisschen gefreut, ja. |
| 476 | **I:** Ja, (AB04: Hm) Okay. Und ähm das heißt, manchmal haben Sie so ein Gefühl, wie von sich selber getrennt sein, distanziert sein von sich selbst, kann man das so sagen? |
| 477 | **AB04:** Hm. Ich versteh die Frage nicht. |
| 478 | **I:** Hm, weil ich habe Sie- als erstes habe ich gesagt, haben Sie manchmal Gefühle, Gedanken, die nicht zu Ihnen gehören? Die fremd sind? Da haben Sie gesagt: "Ja." (AB04: Hm (zustimmend)) Und jetzt ähm frage ich danach- nach dem Gefühl. Gibt es manchmal das Gefühl, von sich selber getrennt zu sein, wie- wie außerhalb von sich selbst zu stehen? Vielleicht nicht ganz so extrem. |
| 479 | **AB04:** Ja, schon. Würde ich schon sagen, ja. |
| 480 | **I:** Ja? (AB04: Ja) Können Sie mir das in- jetzt habe ich so viele Sachen gesagt. Können Sie mir das in Ihren eigenen Worten beschreiben? |
| 481 | **AB04:** Nein. |
| 482 | **I:** Nein? |
| 483 | **AB04:** Mh-mh (verneinend). |
| 484 | **I:** Okay. Ähm. Oder ein Beispiel, wo Sie das Gefühl stark hatten? |
| 485 | **AB04:** [ATMET STARK AUS] und jetzt merk ich das gerade mit der Konzentration. (I: Okay?) Ja. Ich so- ich höre Ihnen zwar zu, aber die letzten drei Sachen, wo sie gesagt haben, hm habe ich jetzt nicht mehr verstanden, also (I: Hm (zustimmend)) lässt gerad total nach. Ich- Ich trink mal kurz einen Schluck. |
| 486 | **I:** Sollen wir eine Pause machen? [MINERALWASSER ZISCHT] |
| 487 | **AB04:** Hm, ja vielleicht kurz. Vielleicht ein, zwei Minuten. [MINERALWASSER GIEẞT EIN] |
| 488 | **I:** Vielleicht nehmen Sie einen Keks? |
| 489 | **AB04:** Nee, danke. (I: Nö?) Nee. Ich bin nicht so der Süße, deshalb. (I: Ah) Ja. Das einzige wa- was wirklich gut war, wo- wo ich normal eigentlich auch nicht esse, ist äh Griesbrei immer morgens beim Frühstück. Da ist ja auch Zucker drin (I: Ja.). Und normal ich esse keins- kei- kein Milchreis, oder Waffeln, oder so was, alles gar nicht. Aber der Griesbrei, der war echt gut, da habe ich immer ordentlich zugehauen. (AB04 und I: [LACHEN]) |
| 490 | **I:** Vielleicht können Sie nach einem Rezept fragen? Den selber machen? |
| 491 | **AB04:** Ja. Ja, ist eigentlich nicht schwer, so ein Griesbrei. (I: Eigentlich nicht) Das kriege ich- kriege ich bestimmt hin. |
| 492 | **I:** Gibt es auch in- in Packungen, glaube ich. |
| 493 | **AB04:** Ja, ja. |
| 494 | **I:** Ich weiß auch nicht, ob die den hier selber machen in der Klinik. |
| 495 | **AB04:** Das weiß ich auch nicht, ne. Klingt auch ein bisschen eklig, aber: Sie müssen mal in den Grießbrei den Joghurt reinmachen, (I: [LACHT]), das ist der Hammer (AB04 und I: [LACHEN]). Noch zwei Tütchen Zucker rein. [LACHT] |
| 496 | **I:** Das ist richtig Powerfrühstück. |
| 497 | **AB04:** Ja. Okay |
| 498 | **I:** Okay? (AB04: Ja) Gut, dann machen wir weiter. Ähm, okay. Ich hatte Sie gefragt, ob Sie manchmal das Gefühl haben, distanziert zu sich selber zu sein. |
| 499 | **AB04:** Ja, schon, teilweise. |
| 500 | **I:** Ja? Aber dafür können Sie mir aber kein Beispiel nennen? |
| 501 | **AB04:** Mh. Ich würde sagen distanziert zu mir selber. Ähm. [PAUSE]. Naja, das würde ich- würde ich ähm so beschreiben, dass ich halt meinen eigenen Wünsche n- nicht nachkommen kann. |
| 502 | **I:** Hm, okay (AB04: Ja). Aber das ist aber nicht so eine Situation, wo Sie dann richtig neben sich stehen und sich von Außen sehen können? |
| 503 | **AB04:** Nein, nein |
| 504 | **I:** Und so was, da irgendwie so richtig eine räuml- also das fühlen auch, dass Sie- oder sehen, dass Sie (AB04: Mh-mh (verneinend)) distanziert von sich selbst sind? (AB04: Mh-mh (verneinend)) Okay. Also das ist mehr so ein: Ich bin von meinen eigenen Wünschen und Zielen getrennt? |
| 505 | **AB04:** Ja. |
| 506 | **I:** So etwas? |
| 507 | **AB04:** Ja. |
| 508 | **I:** Hm (zustimmend). Okay. Mh. [PAUSE] Und hatten Sie schon mal das Gefühl, nicht so richtig in der Welt anwesend zu sein? Da haben Sie vorhin gesagt: "Nee." |
| 509 | **AB04:** Nee, eigentlich nicht, nee. |
| 510 | **I:** Okay. Also Sie sind immer da? |
| 511 | **AB04:** Hm ja, seit meiner Geburt. (AB04 und I: [LACHEN]) |
| 512 | **I:** Okay. (AB04: Hm (zustimmend)) Ähm. Naja manche Leute beschreiben so etwas wie, dass "ich bewege mich in der Welt, und ähm und interagiere mit den Leuten, aber bin gar nicht so richtig da. Also immer, als wäre ich so ein bisschen wie in einer anderen Welt." |
| 513 | **AB04:** Ja, das ist die Erkrankung. |
| 514 | **I:** Ja, das ist- genau. Das ist die Erkrankung, die das macht, ja? (AB04: Ja) Das glaube ich auch. Nur manche Leute erleben das, und andere erleben das nicht. |
| 515 | **AB04:** Ja, ich weiß. Das ähm. Die Schizophrenie ist bei jedem ganz anders, (I: ganz anders, genau) ja und das macht das sehr kompliziert. |
| 516 | **I:** Ja. Total. (AB04: Hm (kleinlaut)) Also Sie erleben so etwas nicht? |
| 517 | **AB04:** Mh-mh (verneinend). |
| 518 | **I:** Nee, gut. Okay. Das ist ja umso besser. |
| 519 | **AB04:** Hm ja, stimmt, ja. |
| 520 | **I:** Ähm, gibt es denn Situationen, wo Ihnen die Umgebung irgendwie verändert vorkommt? Wo die sich irgendwie- wo die anders aussieht, oder sich komisch verändert? Nee? |
| 521 | **AB04:** Mh-mh (verneinend). |
| 522 | **I:** Und dann hatten wir- vorhin hatten wir das so angerissen ähm, dass es Situationen gibt, wo Sie viel über Ihre eigenes Verhalten nachdenken? Und sich fragen: "Wieso habe ich das so gemacht? Habe ich das richtig gemacht?" (AB04: Hm, ja, ja, ja) Ähm. Gibt es da denn auch so Momente, wo Sie sich fragen, ähm, warum sind bestimmte Dinge so und nicht anders? Also es gab zum Beispiel mal jemand, der mir gesagt hat: "Ja dann frage ich mich, warum ist denn eigentlich bei der Ampel unten grün und oben rot?" (AB04: Hm) So ganz grundsätzliche Sachen, (AB04: Ja) die- die so irgendwie einfach sind, (AB04: Ja) dass Sie die hinterfragen? |
| 523 | **AB04:** Ja. (I: Ja?) Sehr viel. |
| 524 | **I:** Sehr viel? |
| 525 | **AB04:** Ja . |
| 526 | **I:** Können Sie mir das beschreiben? |
| 527 | **AB04:** Naja. Warum- Warum hat man ähm- warum- warum hat man ähm Geld und nicht tauschen und äh (I: Hm (zustimmend)) naja alles, so. Ja, ich weiß, was Sie meinen. (I: Ja?) Ja. Das habe ich. |
| 528 | **I:** Ja. (AB04: Hm) Und dass Sie sich dann fragen: "Wieso funktioniert die Welt so, und warum funktioniert sie nicht eigentlich ganz anders?" |
| 529 | **AB04:** Mh ja, genau. (I: Ja?) Ja warum ist bei der Kohlensäure- warum blubbert das dabei? (I: Ah.) So. Ja, alles, so (I: Ja.) Sachen, ja. Hm. |
| 530 | **I:** Stimmt, ja. So. Habe ich mich auch noch nicht gefragt: "Wieso blubbert das eigentlich?" |
| 531 | **AB04:** Ja, genau. (I: Ja) Für- das ist eigentlich wichtig, so Sachen: "Warum blubbert das eigentlich?" (I: Ja.) Gibt es natürlich ne ähm (I: genau) Antwort. |
| 532 | **I:** Irgendeine chemische. Aber- aber die Fragen kommen Ihnen dann? |
| 533 | **AB04:** Ja. (I: Ja?) Hm. |
| 534 | **I:** Und bezieht sich das dann auch auf Ihr eigenes Verhalten? "Warum verhalte ich mich so? Warum verhalten wir uns eigentlich in sozialen Situationen so?" |
| 535 | **AB04:** Ja, ich frage mich schon seit Jahren, warum wir Menschen sehen, dass wir ich sage jetzt mal- ich mache jetzt mal ein gutes Beispiel. Und zwar, wenn [GLAS WIRD AUF TISCH GESTELLT] man jetzt hier, das sind so, [GLAS WIRD AUF TISCH GESTELLT] das ist jetzt äh die äh die Erde, (I: Hm (zustimmend)) ja unser Planet (I: Ja.) und die Kekse sind äh Ressourcen für unsere Leben. (I: Okay.) So und wir stehen hier (I: Ja.) und hier sind die Ressourcen (I: Ja.) und wir nehmen uns davon (I: Ja.) und irgendwann fallen wir dann runter [TELLER MACHT GERÄUSCH AUF TISCH]. (I: Ah) Weil- und warum sehen wir das, k- erkennen das, dass wir unsere Welt kaputt machen, (I: Ja.) beispielsweise (I: Ja.) un- und stehen dann trotzdem jeden Morgen auf [TELLER MACHT GERÄUSCH AUF TISCH] und machen weiter so? (I: Genau.) Ja. (I: Okay.) Hm. |
| 536 | **I:** Also so eine Umwelt- Umwelt- |
| 537 | **AB04:** Äh, so- (I: bezogene) so komische Sachen, oder so etwas, mache ich mir viel Gedanken, so, ja. |
| 538 | **I:** Und das ja, (AB04: Ja) gar nicht- ist ja gar nicht so komisch, ne? Das fragen sich gerade viele Umweltschützer auch. (AB04: Hm, ja, stimmt.) Stimmt, (AB04: Hm, ja) ja. |
| 539 | **AB04:** Stimmt, es ist ja eigentlich gar nicht komisch, das zu denken, oder? |
| 540 | **I:** Nee. |
| 541 | **AB04:** Ja. |
| 542 | **I:** Also, eigentlich ist es nicht komisch. |
| 543 | **AB04:** Ja. [PAUSE] (I: Und dann-) Das finde ich gerade faszinierend, irgendwie. (I: Ja?) Dass ich das vergessen habe, ob das jetzt ein normales Denken ist, oder nicht. (I: Okay.) Darüber denke ich gerade nach. (I: Ja.) Ja. |
| 544 | **I:** Ja. Und hat das dann- bezieht sich äh also denken Sie dadrüber nach, aber bezieht sich das dann auch auf Ihr Handeln? |
| 545 | **AB04:** Mh-mh (verneinend). |
| 546 | **I:** Also das sind Ge- das sind einfach Gedanken, die Ihnen kommen? |
| 547 | **AB04:** Hm ja. |
| 548 | **I:** Hm (zustimmend). [GLAS WIRD AUF TISCH GESTELLT] |
| 549 | **AB04:** Na das bespreche ich ja auch mit einem Kumpel mit so Themen. |
| 550 | **I:** Ja? |
| 551 | **AB04:** Ja. |
| 552 | **I:** Okay. (AB04: Hm) [PAUSE] [WASSER GIEẞT EIN UND FLASCHE SCHLIEẞT] (AB04: Mh-mh (unsicher)). Also das ist ja gerade ein Riesenthema, die ganze Ressourcenfrage und wie (AB04: Ja, ja.) [Crosstalk] geht es auch um Klimaerwärmung und jetzt ist es so heiß. (AB04: Hm(zustimmend)) Ja. Ähm. Hatten Sie denn schon mal das Gefühl, irgendwie, dass Sie nicht mehr eine Person sind? Dass Sie sich wie so spalten in zwei Personen? [PAUSE] Oder in mehrere? |
| 553 | **AB04:** Hm nee, das ist die Schizophrenie. Einmal die Krankheit und einmal ich. |
| 554 | **I:** Okay. Das sind (AB04: Ja) zwei Personen sozusagen? |
| 555 | **AB04:** Nö, das ist eine Person. |
| 556 | **I:** Das ist eine Person? |
| 557 | **AB04:** Ja, aber- |
| 558 | **I:** Da gibt es Tei- mehrere Teile (AB04: Ja, genau, ja) in Ihnen? |
| 559 | **AB04:** Ja. |
| 560 | **I:** Okay. Und ähm. Also eine- einen kranken Teil, und einen gesunden Teil, sagen wir so? |
| 561 | **AB04:** Hm (zustimmend). |
| 562 | **I:** Ja? |
| 563 | **AB04:** Hm (zustimmend). |
| 564 | **I:** Okay. Und ähm. Können Sie diese Teile verorten? Sind die irgendwo? Oder sind- |
| 565 | **AB04:** Kann ich nicht verorten. |
| 566 | **I:** Die sind beide überall? |
| 567 | **AB04:** Hm (fragend). Wie meinen Sie das? |
| 568 | **I:** Naja. Ähm. Manche Leute sagen auf der linken Seite ist der eine Teil, oder auf der rechten Seite ist der andere Teil. |
| 569 | **AB04:** Mh-mh (verneinend). |
| 570 | **I:** So etwas? Geht nicht? |
| 571 | **AB04:** Mh-mh (verneinend). |
| 572 | **I:** Okay. Die sind sozusagen beide überall in Ihnen, diese- diese Anteile? |
| 573 | **AB04:** Mh gehören zu- zu mir. |
| 574 | **I:** Gehören zu Ihnen? |
| 575 | **AB04:** Ja. |
| 576 | **I:** Ja. (AB04: [RÄUSPERT SICH]) Hm. [PAUSE] Und hatten Sie schon mal das Gefühl ähm, jemand anders zu sein? |
| 577 | **AB04:** [PAUSE] Hm (kleinlaut). |
| 578 | **I:** Als würden S- also vielleicht jemand, den Sie kennen, oder vielleicht auch ganz fremd? |
| 579 | **AB04:** Hm ja also, wie kann- wie kann man es sagen. Eher so, wenn man anderen was nachmacht, meinen Sie? |
| 580 | **I:** Vielleicht, (AB04: Hm (kleinlaut)) ja. Aber (AB04: Hm (kleinlaut)) auch nicht unbedingt. Manchmal kommt so ein Gefühl einfach so. (AB04: Hm(zögerlich)) Also. Wenn Sie- |
| 581 | **AB04:** Hm ja, habe ich auch schon gehabt, ja. |
| 582 | **I:** Haben Sie schon gehabt? |
| 583 | **AB04:** Hm (zustimmend). |
| 584 | **I:** Und können Sie sich erinnern an, wer Sie dann waren, oder wie Sie- wie es sich angefühlt hat? |
| 585 | **AB04:** Hm (zögerlich). Nee, kann ich irgendwie nicht wiedergeben, nee. |
| 586 | **I:** Nee. Gibt es eigentlich eine- wenn das plötzlich auftritt, dass man das Gefühl hat: "Jetzt bin ich jemand anders", das ist ja was, was man- was einem dann- [ATMET VERSCHRECKT EIN] das ist so ein Schreck, oder? |
| 587 | **AB04:** Hm nee. |
| 588 | **I:** Nee? |
| 589 | **AB04:** Mh-mh (verneinend). |
| 590 | **I:** Wie war das- |
| 591 | **AB04:** Ich bin ja nicht jemand anders, ich bin mh ich bin ich. |
| 592 | **I:** Sie sind sie selbst? |
| 593 | **AB04:** Hm ja! (starke Zustimmung) |
| 594 | **I:** Ja? |
| 595 | **AB04:** Ja. |
| 596 | **I:** Okay, das heißt- |
| 597 | **AB04:** Und das ganze Denken, was Sie jetzt beschreiben, (I: Hm (zustimmend)) was man hat, das ist ja alles Denken, wo nicht normal ist. Das ist äh- und so- und so das denk ich dann (I: Hm (zustimmend)) und dann ist wieder ein bisschen besser so. |
| 598 | **I:** Okay. (AB04: Ja) Also das ist einfach so ein Anteil, der ist da, und den haben Sie unter (AB04: [RÄUSPERT SICH]) Kontrolle? Jetzt gerade? |
| 599 | **AB04:** Hm na ich habe es- ich habe es nicht unter Kontrolle, nee. |
| 600 | **I:** Ein bisschen unter Kontrolle? |
| 601 | **AB04:** Hm ja (zustimmend, zögerlich). |
| 602 | **I:** Ja? |
| 603 | **AB04:** Hm. Naja, was- was heißt "unter Kontrolle"? Es ist halt- ist halt da und so wam- wie man es verarbeitet oder so, (I: Hm (zustimmend)) das ist ja sozusagen eine eigene Entscheidung. |
| 604 | **I:** Okay. (AB04: Ja) Okay. Und ähm wie erleben Sie Ihr eigenes Alter? Ist es (AB04: Hm (zögerlich)) richtig? |
| 605 | **AB04:** Na also ist schon- also ich muss ganz ehrlich sagen, ich bin [ANONYMISIERT, ALTERSANGABE] (I: Hm (zustimmend)) und es kommt mir nicht vor, wie [ANONYMISIERT, ALTERSANGABE] Jahre. |
| 606 | **I:** Sondern? |
| 607 | **AB04:** Wie [ANONYMISIERT, ALTERSANGABE] irgendwie. |
| 608 | **I:** Ja? |
| 609 | **AB04:** Ja. |
| 610 | **I:** Also erleben sich viel jünger, als Sie- als Sie sind? |
| 611 | **AB04:** Nee, nicht jünger! Ich mh- es kommt mir nicht so vor, wie, dass ich schon [ANONYMISIERT, ALTERSANGABE] Jahre auf der Welt bin. (I: Ah, okay.) Also kommt mir eher wie [ANONYMISIERT, ALTERSANGABE] Jahre, oder (I: Ahja?) [ANONYMISIERT, ALTERSANGABE], ja. |
| 612 | **I:** Okay. Okay. Und wie bis- wie ist es mit dem Geschlecht? (AB04: Hm (leise)) Also passt das Geschlecht? |
| 613 | **AB04:** Ja ich bin- ich bin ein Mann. |
| 614 | **I:** Sie sind ein Mann? |
| 615 | **AB04:** Ja. |
| 616 | **I:** Pass- passt das zu Ihrer Wahrnehmung? |
| 617 | **AB04:** Ja. |
| 618 | **I:** Ja? |
| 619 | **AB04:** Hm (leise). |
| 620 | **I:** Gab es da mal Zeiten, wo Sie das Gefühl hatten: "Das passt nicht"? |
| 621 | **AB04:** Mh-mh (verneinend). |
| 622 | **I:** Nee? |
| 623 | **AB04:** Nee. |
| 624 | **I:** Es gibt auch Leute, die dann sagen ähm, plötzlich hat mein Geschlecht nicht mehr zu mir gepasst. |
| 625 | **AB04:** Ah, so Transen! |
| 626 | **I:** Zum Beispiel, aber ähm das muss- kann- muss auch nicht über so eine lange Zeit sein, dass dieses Gefühl (AB04: Achso) besteht, sondern (I: AB05 (zustimmend)) bei manchen Leuten ist es gut so, und dann verändert sich das. Und auch was die sexuelle Orientierung betrifft. (AB04: Hm (zustimmend)) Also da sind Sie auch immer- die war immer klar bei Ihnen? |
| 627 | **AB04:** Hm, ja. |
| 628 | **I:** Dass Sie auf- auf Frauen stehen? |
| 629 | **AB04:** Ja, genau. (I: Ja?) Hm. |
| 630 | **I:** Okay. Okay. Mh. Dann lassen Sie uns noch über Ängste sprechen. (AB04: Okay) Ähm, jetzt haben Sie schon gesagt, Sie hatten Angst, Sie müssen sterben (AB04: Hm (zustimmend)) oder Angst ähm, ähm rauszugehen, dass Sie- dass Sie jemand attackiert oder so. (AB04: Hm (zustimmend)) Können- das- das ist immer in akuten psychotischen Phasen gewesen, oder? Oder war- ist es auch jetzt noch da? |
| 631 | **AB04:** Ist auch jetzt da. |
| 632 | **I:** Sie haben jetzt auch noch Ängste? |
| 633 | **AB04:** Ja. |
| 634 | **I:** Ja? |
| 635 | **AB04:** Ich gehe nicht im Dunkeln raus. |
| 636 | **I:** Im Dunkeln, okay. |
| 637 | **AB04:** Nee, ich geh nicht im Dunkeln raus! |
| 638 | **I:** Ja, ja, im Dunkeln gehen (AB04: Ja) Sie nicht raus. (AB04: Ja) Okay. Und ähm. Können Sie mir Ihre Ängste beschreiben, oder ist das zu unangenehm? |
| 639 | **AB04:** Mhh. Na ich habe halt Angst, entführt zu werden, umgebracht zu werden. (I: Hm (zustimmend)) Ja. |
| 640 | **I:** Okay. Und ähm- |
| 641 | **AB04:** Aber auf der einen Seite- auf der einen Seite glaube ich, dass es irgendwann passiert, auf der anderen Seite glaube ich, dass es nicht passiert. |
| 642 | **I:** Ja (verständnisvoll). (AB04: Ja) Ja. Und die Medikamente, machen die die Ängste weniger? |
| 643 | **AB04:** Ja. |
| 644 | **I:** Ja, also- die sind stärker in- in- in psychotischen Phasen sind diese- sind diese Ängste stärker? Die werden dann unkontrollierbar? |
| 645 | **AB04:** Ja, genau, (I: Ja.) ja. |
| 646 | **I:** Hm (verständnisvoll). Und wenn SIe so Angst haben, spüren Sie das dann auch körperlich? |
| 647 | **AB04:** Mh (zustimmend). |
| 648 | **I:** Also was- was spüren Sie da? |
| 649 | **AB04:** Na ich äh ich zittere und so etwas. |
| 650 | **I:** Sie zittern (AB04: Ja) und Sie haben (AB04: Hm), Sie kriegen schwitzige Hände, (AB04: Hm, ja) vielleicht? |
| 651 | **AB04:** Zum Beispiel, ja. |
| 652 | **I:** Müssen schnell atmen? |
| 653 | **AB04:** Ja. |
| 654 | **I:** Okay. Mh. Und was machen Sie dann? |
| 655 | **AB04:** Gar nichts, alles schlucken. [PAUSE] |
| 656 | **I:** Sie sitzen dann- w- können Sie- |
| 657 | **AB04:** Durchstehen! |
| 658 | **I:** Durchstehen, (AB04: Ja) okay. Können Sie- |
| 659 | **AB04:** Ich versuch mich in so'n Situationen immer daran zu erinnern, was- was würde jetzt sein, wenn- die Krankheit nicht da wäre (I: Hm (zustimmend)) und dem versuche ich Folge zu leisten. |
| 660 | **I:** Okay. Können Sie mir ein Beispiel beschreiben, eine Situation beschreiben, wo das als letztes so passiert ist? Kommt das dann einfach (AB04: Mh) plötzlich auf oder gibt es einen Auslöser dafür? |
| 661 | **AB04:** Hm. Beides, also es kommt manchmal plötzlich und manchmal macht es ein Auslöser. |
| 662 | **I:** Okay. (AB04: Ja) Können Sie mir eine Situation beschreiben, die gut passt? |
| 663 | **AB04:** Naja, ich ah- ich empfinde Hupen als sehr aggressiv. (AB04: Ahja) Ja. |
| 664 | **I:** Und dann kriegen Sie Angst? |
| 665 | **AB04:** Mh ja, schon. |
| 666 | **I:** Hm (zustimmend). Okay. |
| 667 | **AB04:** Bei so- bei so'n äh bei so'n äh Themen rede ich eigentlich immer gar nicht drüber, (I: Hm (zustimmend)) weil ich irgendwie der Überzeugung bin, dass dann alles noch schlimmer wird. (I: Okay.) Weil wenn denn- wenn ich anderen Leuten äh mitteile, wie es mir geht und- und hm was ich gerade denke und wie es funktioniert, sozusagen, dass es dann n- noch schlimmer wird, weil die anderen Leute wissen es ja dann. |
| 668 | **I:** Hm (nachdenklich). (AB04: Ja) [GLAS WIRD AUF TISCH ABGESTELLT] Und- und haben Sie die Erfahrung gemacht, dass es so ist? Dass, wenn Sie jetzt mit mir darüber reden, dass es dann schlimmer wird? |
| 669 | **AB04:** Hm- hm nicht unbedingt, aber ich fühle mich schon seit Jahren über mein Handy ausspioniert zum Beispiel, ja. (I: Okay.) Und deswegen ähm denn- über so etwas rede ich dann eigentlich gar nicht. |
| 670 | **I:** Ja, ich kann verstehen, dass das schwierig ist, über so etwas zu reden. (AB04: Ja) Klar. Wenn man die Dinge- |
| 671 | **AB04:** Man hat halt Angst, wenn man zu viel preisgibt, das alles noch schlimmer wird dann. (I: Ja, ja) Ja. |
| 672 | **I:** Klar, klar. Und ähm haben Sie die Erfahrung gemacht, dass Psychotherapie, wo man ja viel redet, (AB04: Hm (zustimmend)) ne? Das haben Sie ja jetzt auch erlebt, oder? |
| 673 | **AB04:** Ja. |
| 674 | **I:** Dass Ihnen das hilft, oder dass das eher schwieriger wird? |
| 675 | **AB04:** Hm. Also das- das ist ja jetzt vielleicht jetzt kein therapeutisches Gespräch, aber ich find das hier jetzt am besten. (I und AB04: [LACHEN]) |
| 676 | **I:** Das- das- das ist schön, (AB04: [RÄUSPERT SICH]) da- das ist schön, dass Sie das als positiv erleben. (AB04: Hm) Aber ich meinte jetzt generell, haben Sie- wenn Sie reden darüber, haben Sie dann die Erfahrung, dass- gemacht, dass Ihnen das hilft? |
| 677 | **AB04:** Ich rede nicht drüber. |
| 678 | **I:** Das machen Sie gar nicht? |
| 679 | **AB04:** Hm (zustimmend, kleinlaut). |
| 680 | **I:** Okay. Also Sie haben jetzt auf Station [ANONYMISIERT, NAME EINER ÖRTLICHEN PSYCHIATRISCHEN STATION], wo Sie drei Monate waren, haben Sie da individuelle Psychotherapie gehabt? |
| 681 | **AB04:** Ja. |
| 682 | **I:** Ja? Da- was haben Sie da geredet? |
| 683 | **AB04:** Hm normale Sachen. |
| 684 | **I:** Okay. (AB04: Hm (zustimmend)) Normale Sachen heißt? |
| 685 | **AB04:** Hm ja, wenn- wie es einem geht, und so, wo- wo hm wegen [ANONYMISIERT, NAME DER PARTNERIN DER INTERVIEWTEN PERSON] und alles hm (I: Okay.), ja. |
| 686 | **I:** Darüber, also über- (AB04: Hm (zustimmend)) über den Alltag, auch? |
| 687 | **AB04:** Ja, über den Alltag. |
| 688 | **I:** Nicht über Ihre- Ihre Ängste? |
| 689 | **AB04:** Hm. |
| 690 | **I:** Hm, okay. Naja- |
| 691 | **AB04:** Also nicht, weil die Psychologin schlecht war, oder so, sondern weil ich habe das immer so- eben- (I: Genau) ähm ja. |
| 692 | **I:** Genau, also das glaube ich Ihnen sofort, dass Sie- dass das schwierig ist einfach, da-darüber zu reden, ne? |
| 693 | **AB04:** Hm (zustimmend, kleinlaut). |
| 694 | **I:** Hm. Und, Sie müssten selber wissen, was Ihnen was hilft, ne? Das können nur Sie entscheiden, was Ihnen was bringt. Nur (AB04: Hm (leise)) dafür ist Psychotherapie eigentlich da, um über solche Dinge zu reden. Da können Sie mal gucken (AB04: Hm(zustimmend)), vielleicht mal ausprobieren, wie das- wie das sich verändert, wenn ich dadrüber spreche. Aber Sie müssen selber entscheiden- |
| 695 | **AB04:** Ich habe sehr viele Sachen, wo man eigentlich erzählen müsste, wenn man in der Klinik aufgenommen wird. Der Doktor kommt, stellt fragen, müsste man sagen und erzählen. Aber ich mach das schon seit Kind an nicht mehr. (I: Okay.) Also erzähle ich gar nicht alles. (I: Okay.) Die Ganze Geschichte so. Mh. |
| 696 | **I:** Und was erzählen Sie dann da, wenn Sie da kommen? |
| 697 | **AB04:** Mh. Na ich ähm das was- was- was ähm in Wirklichkeit ist, was mit mir los ist (I: Hm (zustimmend)) und das würde ich nicht erzählen, und das, was ich nicht erzählen will, lass ich einfach weg (I: Okay.) und dadurch, dass die Leute das ja nicht wissen, können Sie das auch nicht erfragen. (I: Ja, ja, klar) Hm. (I: Klar.) Das war top secret, so das ich Ihnen das gesagt habe. [LACHT LAUT] |
| 698 | **I:** Okay, das äh das nehm ich- das nehm ich- Ihre Daten werden auch anonym gespeichert, also wir machen gleich noch einen Code für Sie, damit werden die abgespeichert und dann kann (AB04: Ja) niemand wissen, dass Sie das erzählt haben. Und ähm das ist nur für wissenschaftliche Zwecke, (AB04: Hm (zustimmend) also es wird nicht weitergegeben. (AB04: Hm (zustimmend)) [QUITSCHENDES GERÄUSCH IM HINTERGRUND]. Aber ich würde trotzdem noch mal eine Lanze brechen für die Psychotherapie. (AB04: Ja.) Für die Zukunft, wenn Sie da- wenn Sie da reingehen und jetzt auch auf [ANONYMISIERT, NAME EINER ÖRTLICHEN PSYCHIATRISCHEN STATION], ne? (AB04: Hm, hm (zustimmend)) Da haben Sie ja intensiv auch Psychotherapie. (AB04: Ja) Dass- dass Sie da versuchen können, mal zu gucken, was bringt mir was, was ich erzähle. |
| 699 | **AB04:** Nee, mache ich nicht, weil ich habe ja nur eine Chance. |
| 700 | **I:** Was meinen Sie, Sie haben nur eine Chance? |
| 701 | **AB04:** Na wenn ich tot bin, bin ich tot. Wenn, dann lässt sich daran nichts mehr ändern. |
| 702 | **I:** Okay. Und (AB04: Hm) Sie meinen, wenn Sie das erzählen- |
| 703 | **AB04:** Nee! Das hm mache ich nicht (unverständlich), das würde es- [PAUSE] [MOTORGERÄUSCHE IM HINTERGRUND] (I: Das könnte-) Die Frage kann ich nicht beantworten, weil sonst gebe ich dann schon meiner Meinung nach zu viel preis. |
| 704 | **I:** Okay, okay. (AB04: Ja.) Kein Problem, kein Problem. Mh. Dann frage ich einfach weiter. Ähm. Diese Ängste, die Sie haben, sind das Ängste, die immer mal wieder kommen und dann wieder weggehen (AB04: Ja.) oder sind es- ist es die ganze Zeit so etwas, was da ist, immer? |
| 705 | **AB04:** Ja es ist die ganze Zeit leicht da. (I: leicht) Aber es gibt auch als wenn man Hügel ich so bekomme, (I: Hm (zustimmend)) wo stärker wird. |
| 706 | **I:** Hm. Also kann man sagen, dass es so eine tiefgreifende Unsicherheit ist, so eine ähm, so ein ständiges Gefühl, man muss sich irgendwie verteidigen, oder man muss auf sich aufpassen? |
| 707 | **AB04:** Mh. Verteidigen nicht unbedingt, also. Ich habe mich schon oft gefragt, wenn ich wirklich entführt werde, was mach ich denn dann? Wehre ich mich, kämpfe ich? (I: Hm (zustimmend)) Lass ich es einfach über mich ergehen? (I: Hm (zustimmend)) Hm (ratlos). Weiß ich ehrlich gesagt gar nicht so genau. (I: Okay.) Ja. |
| 708 | **I:** Und jetzt mal [FAHRRADKLINGEL ERTÖNT] ähm bezogen- |
| 709 | **AB04:** Eine Zeit lang- eine Zeit lang war ich zu Hause alleine, und da habe ich eine Schreckschusspistole gehabt. Da ha- da habe ich mir gedacht, wenn jetzt einer kommt, schieße ich dem Pfefferspray ins Gesicht. Und da- da war ich voll so auf- auf dem Aggressivtrip, (I: Hm (zustimmend)) sag ich jetzt mal. (I: Hm (zustimmend)) Mh, dann gab es wieder aber auch Zeiten, wo-wo ich gesagt hab, so hm wenn es passiert, passiert es, dann bin ich halt weg. So. Ja. (I: Okay.) Ganz unterschiedlich. |
| 710 | **I:** Also die Einstellung dazu verändert sich? |
| 711 | **AB04:** Ja, ja, genau. (I: Ja.) Es verändert sich (I: Hm (zustimmend)). |
| 712 | **I:** Aber die Angst an sich. Ist die- also ist es so ein- immer wie so ein Gefühl, jetzt könnte gleich etwas Schreckliches passieren? |
| 713 | **AB04:** Hm (zustimmend). (I: Ja?) Draußen, ja. |
| 714 | **I:** Draußen? |
| 715 | **AB04:** Ja. |
| 716 | **I:** Drinnen auch? |
| 717 | **AB04:** Hm, eher se- eher selten. |
| 718 | **I:** Also weniger stark, oder? |
| 719 | **AB04:** Ja, weniger stark, ja. |
| 720 | **I:** Oder ist es dann ganz weg? |
| 721 | **AB04:** Nö, nicht ganz weg. |
| 722 | **I:** Ist immer so ein bisschen da, ne? (AB04: Hm (zustimmend)) Okay. |
| 723 | **AB04:** So komische, keine Ahnung, Gedanken wie es könnte ja eine Granate irgendwann durch das Fenster reinkommen. |
| 724 | **I:** So etwas auch? |
| 725 | **AB04:** Ja, (I: Ja?) also ganz verrückte Sachen, so, (I: Okay.) ja. Löst nicht richtig Angst aus, weil es irgendwie ein bisschen äh für mich ein bisschen verrückt ist so, ein bisschen, (I: Hm (zustimmend)) "woher eine Granate?", so. Aber (I: Hm (zustimmend)) ich denke dann nur: "Ist da". |
| 726 | **I:** Okay. (AB04: Hm (zustimmend)) Okay. Und es kommt auch so aus dem Nichts, ne? Das ist ja nicht so- |
| 727 | **AB04:** Ja. (I: Hm (zustimmend)) Genau, guck ich halt Fernsehen, und auf einmal denk ich: "Da kommt gleich eine Granate rein." (I: Okay.) Und dann denke ich wieder: "Was für ein Quatsch." Und dann gucke ich weiter Fernsehen. (I: Okay) Hm. |
| 728 | **I:** Hm (zustimmend). Und hatten Sie schon mal das Gefühl, nicht so richtig bei vollem Bewusstsein zu sein, ohne dass Sie Alkohol getrunken hatten oder Drogen genommen haben? Ne? |
| 729 | **AB04:** Mh-mh (verneinend). |
| 730 | **I:** Da haben Sie ja vorhin auch gesagt: "Ich fühl mich da. Ich bin da (AB04: Ja.) in der Welt." |
| 731 | **AB04:** Ich bin da, genau. |
| 732 | **I:** Ja. Ahm. Gab es Zeiten, wo Sie unfähig waren, Lust oder Spaß zu empfinden? Wo so Sachen, die Ihnen eigentlich Spaß gemacht haben, (AB04: Ja (leise)) Sport, bestimmtes Essen, die plötzlich nicht mehr spüren? |
| 733 | **AB04:** Ja, grad am Anfang, wo es alles angefangen hat, war es ähm sehr stark. (I: Ja?) Und mit den Jahren, hat sich das, was Sie grad beschrieben haben, ahm abgeschwächt. |
| 734 | **I:** Okay. Was war da zum Beispiel nicht mehr schön? |
| 735 | **AB04:** Na alles. Alles (kleinlaut). |
| 736 | **I:** Die ganze Welt. |
| 737 | **AB04:** Ja, ja. Alles. |
| 738 | **I:** Also Essen hat nicht mehr geschmeckt? |
| 739 | **AB04:** Ja. Äh doch es hat geschmeckt, aber hm es- hm ja, wie- wie hm kann man es am besten beschreiben? Äh ja man hat halt die ganze Zeit nur so den Gedanken gehabt so, mh "Ich muss sterben, ich muss sterben, ich muss sterben" und hm da ist halt alles irgendwie an einem vorbei gegangen, so der ganze Rest. (I: Hm (zustimmend)) Ja. |
| 740 | **I:** Also da war die Situation- gab es keine schönen Situationen mehr? |
| 741 | **AB04:** Ja, genau. (I: Ja?) Hm. Man konnte sich an nichts erfreuen, mehr, und so. |
| 742 | **I:** Hm (zustimmend). Weil- weil die- weil die Angst so stark war, oder weil die Dinge auch einfach nicht mehr mh, nicht mehr schön waren, also nicht mehr mh, wie so- wie kann man das sagen? Manche Leute sagen dann, die Sache- die Welt ist dann wie tot, also wie grau. |
| 743 | **AB04:** Ja, genau, grau! Das-das trifft es sehr gut. |
| 744 | **I:** Ja? |
| 745 | **AB04:** Ja. |
| 746 | **I:** Ja? |
| 747 | **AB04:** Ja. (AB04: Also dass die Farben irgendwie, also-) Ich weiß also- ich weiß, was Sie meinen, genau. Wie das- das alles so- es- es- läuft zwar alles, aber es läuft ab wie so ein Film so, wo (I: Aha.) es total ohnmächtig ist, irgendwie total, genau, ja, wo, hm- zweite Reihe ist, so. |
| 748 | **I:** Man sitzt in der Reihe. Das zieht so an einem vorbei? |
| 749 | **AB04:** Nee, nee! Das Leben. Und mh alles- alles andere ist- ist äh zweite Reihe. |
| 750 | **I:** Achso, okay. (AB04: ja.) Okay. Also weiter weg, irgendwie so ein bisschen von einem? |
| 751 | **AB04:** Ja, genau, ja. |
| 752 | **I:** Mh (zustimmend). (AB04: Hm (zustimmend)) Und grau, und passt- |
| 753 | **AB04:** Ja, grau. (I: Ja?) Hm (leise). |
| 754 | **I:** Okay. Also kann man sagen, dass Sie da auch keine Energie hatten, und keinen Elan, und so? |
| 755 | **AB04:** Ja, auf jeden Fall. (I: Ja?) Ja. Wo ich ähm, nach meiner ersten Therapie, wo ich das halbe Jahr obdachlos war, da habe ich im [ANONYMISIERT, ANSCHRIFT EINER OBDACHLOSENUNTERKUNFT] und da- und da äh habe ich gar nichts mehr gemacht. Da ging es mir auch nicht gut. Ich war wochenlang nicht duschen, ich bin- ich bin äh ich habe mich nicht raus, und so. Gar nichts. (I: Gar nichts?) Hm (zustimmend). |
| 756 | **I:** Nichts hat mehr Sie gezogen, sozusagen? |
| 757 | **AB04:** Ja, doch, Alkohol und Drogen. (I: Aha.) Ja, aber das war auch alles. |
| 758 | **I:** Das (AB04: Ja.) war es, hm. Und ähm dass diese- diese Momente, wo diese ganze Energie, der ganze Elan weggeht, (AB04: Hm (zustimmend)) ähm oder nicht da ist, (AB04: Hm (zustimmend)) die gehen aber auch- die sind auch wieder weggegangen, ne? |
| 759 | **AB04:** Ja, ist auch wieder weggegangen. |
| 760 | **I:** Also (AB04: Ja) das ist nicht- das war nicht (AB04: Ja) die ganze Zeit so? |
| 761 | **AB04:** Nein. (I: Okay.) Ich habe danach auch Phasen gehabt, wo ich äh arbeiten gegangen bin sogar ein (I: Ja.) paar Monate und- und- und- und geplant hab, einen Urlaub und (I: Ja.) weiß was ich alles, (I: Ja.) voller Elan. |
| 762 | **I:** Okay. Also es sind nur Phasen? |
| 763 | **AB04:** Ja sind nur Phasen, ja. (I: Hm (zustimmend)) ja. |
| 764 | **I:** [WASSERFLASCHE ÖFFNET] Gut, dann lassen Sie noch kurz über- uns noch kurz [GEGENSTAND WIRD AUF TISCH GESTELLT] über den Körper sprechen [WASSER GIEẞT EIN]. Körpererleben. Hm. Fühlt sich Ihr Körper wie eine Einheit an, oder so etwas? Oder haben Sie das Gefühl, Sie können ihn gut kontrollieren? Wie fühlen sich Bewegungen an? Das ist so ein großer- großer Themenkomplex. Fällt (AB04: Hm (zustimmend)) Ihnen da irgendwie direkt irgendwas ein? |
| 765 | **AB04:** Ja es ist- es ist ziemlich ähm normal geblieben. (I: Ja?) Außer ich ha- halsab so- so Sachen, wo es auf einmal so zuck, oder so. Das hatte ich früher nicht. Hm, ja. Und das halt den Gedankengang, hatte ich ja vorhin erklärt (I: Genau.). Ja, genau. |
| 766 | **I:** Dass die mit dem Zucken zusammenhängen? |
| 767 | **AB04:** Hm, ja. (I: Hm (zustimmend)) [RÄUSPERT SICH] |
| 768 | **I:** Ähm. Also das heißt, so Veränderungen im äh Erlebnis, im Erleben des- Ihres Körpers, wie zum Beispiel, dass Sie mal eine Hand zu groß oder zu klein gesehen haben, oder so? |
| 769 | **AB04:** Mh-mh, nein, (I: Das ist nicht passiert?) nee, nee. |
| 770 | **I:** Und wie ist es, wenn Sie in den Spiegel schauen? (AB04: Hm (abwartend)) Gab es da Phasen, wo Sie viel in den Spiegel geschaut haben? |
| 771 | **AB04:** Nee, gar nicht. (I: Gar nicht.) Normal. |
| 772 | **I:** Ganz normal? |
| 773 | **AB04:** Hm (zustimmend) |
| 774 | **I:** Okay. Oder haben Sie mal die Erfahrung gemacht, dass Ihr Körper, oder Teile des Körpers nicht mehr so richtig zu Ihnen gehört- gehören? |
| 775 | **AB04:** Mh-mh (verneinend). |
| 776 | **I:** Nee. Dann wie sich so taub oder fremd anfühlen? |
| 777 | **AB04:** Mh nur im Normalen, als wenn ja, oder- hm man schläft mal ein Bein ein, (I: Ah, ja.) oder man hat mal einen Krampf oder so (I: Ja.). Ja, alles nur- alles nur normale Sachen. |
| 778 | **I:** Okay. Oder dass Sie das Gefühl hatten, der Körper passt nicht mehr so richtig zu Ihnen? Eigentlich gehört ein anderer Körper zu Ihnen? |
| 779 | **AB04:** Nein. |
| 780 | **I:** Nein. Hm (überlegend). Und irgendwelche anderen rätselhaften Schmerzen? Irgendwelche Körpergefühle, die komisch für Sie waren? Wie zum Beispiel: Ähm, manche Leute sagen: "Ich hatte das Gefühl, ich schwebe", oder so etwas? Oder irgendwelche Schmerzen, die Sie nicht einordnen können? Kribbelgefühle? |
| 781 | **AB04:** Ja, ich habe halt hm das Zucken am Tag (I: Ja.) oder- oder zum Beispiel jetzt, äh- ähm. Also da- da ist ja der Knochen in der Mitte (I: Ja.) und dann sind da außen Muskeln (I: Am Arm?). Und dass die sich jetzt zum Beispiel so kurz mal zusammen zucken und (I: Aha.) dann wieder locker lässt oder so. Und so- und so etwas am ganzen Körper. (I: Okay.) Also, es war nicht immer so, ab und zu mal da, dann wieder halbe Stunde gar nichts. Zuckt nochmal kurz was, und dann wieder gar nichts. So- so habe ich es. |
| 782 | **I:** So etwas haben Sie? |
| 783 | **AB04:** Ja, (I: Okay.) so etwas habe ich, ja. |
| 784 | **I:** So kurze Muskelbewegungen? |
| 785 | **AB04:** Hm (zustimmend). |
| 786 | **I:** Hm. (AB04: Ja.) Wo Sie nicht sagen können, wo die herkommen? |
| 787 | **AB04:** Kann ich nicht sagen. (I: Nee.) Die habe ich auf [ANONYMISIERT, NAME EINER ÖRTLICHEN PSYCHIATRISCHEN STATION] Bescheid gesagt, dort hat man gesagt, das kann viele Ursachen haben. |
| 788 | **I:** Ja. (AB04: Ja.) Ja. Klar. Das kann natürlich auch mit den Medikamenten zusammen hängen, das weiß ich nicht, aber, aber ähm. (AB04: Hm (zögerlich)) Oder mit Stress. |
| 789 | **AB04:** Wei- weiß ich auch (I: ne?) nicht, (I: Ja.) ja. Vielleicht das gibt mir ja manchmal Nasenbluten. |
| 790 | **I:** Oder- Zuckungen (AB04: Ja.) kann man schon auch kriegen, (AB04: Ja?) ja, ja. (AB04: Hm). Sie kriegen Nasenbluten, wenn Sie Stress haben? |
| 791 | **AB04:** Mh nee, eigentlich nicht, (I: Andere-) aber ich kenne das so. |
| 792 | **I:** Okay (AB04: Ja.) Sie kennen das von anderen Leuten. |
| 793 | **AB04:** Weiß gar nicht mehr, wann ich das letzte Mal Nasenbluten hatte, ewig her. |
| 794 | **I:** Lange her? [LACHT] |
| 795 | **AB04:** Ja. |
| 796 | **I:** Okay. Oder hatten Sie mal das Gefühl, Ihr Körper, oder Körperteile sind wie Dinge oder Gegenstände im Raum? |
| 797 | **AB04:** Nein. |
| 798 | **I:** Nein. Und wie ist es mit den eigenen Bewegungen? Wie nehmen Sie die wahr? |
| 799 | **AB04:** Mh, na norma- normal eigentlich. |
| 800 | **I:** Ja? (AB04: Ja.) Also ist Ihnen nichts aufgefallen? Zum Beispiel, so Sachen wie, ähm dass Sie- dass sich Körperteile bewegen, ohne dass Sie es eigentlich wollen? |
| 801 | **AB04:** Ja, doch, (I: Doch?) habe ich ja. (I: Dass mit den Zuckungen) Zuckungen. |
| 802 | **I:** Genau, das haben Sie gesagt. (AB04: Ja.) Aber das ist jetzt nicht so, dass Ihr Arm sich plötzlich bewegt, ohne dass Sie das wollen? |
| 803 | **AB04:** Ja, doch. So etwas passiert auch ähm oder meine Zehen, oder so. |
| 804 | **I:** Okay, also das heißt, es sind nicht nur Ihre Muskeln? |
| 805 | **AB04:** Wenn das jetzt meine Füße sind [MACHT TIPPENDES GERÄUSCH] (I: Hm (zustimmend)) und ich mh- mh gucke zum Beispiel Fernsehen, hm geht es auf einmal beides, so. Immer wieder. Und dann denke ich wieder: "Hä, warum machst'n das?" und dann mache ich wieder so. |
| 806 | **I:** Ah, verstehe. (AB04: Und- Ja.) Also es sind schon richtige Bewegungen, (AB04: Ja.) nicht nur so kleine- es gibt kleine Zuckungen, (AB04: Ja.) aber auch richtige Bewegungen, wo Sie-? |
| 807 | **AB04:** Ja, ge- ja genau. |
| 808 | **I:** Okay. (AB04: Ja.) Mh-h (zustimmend). Und ähm hatten Sie mal das Gefühl, Ihr Körper bewegt sich, ohne dass er sich ge- bewegt hat? |
| 809 | **AB04:** Mh-mh (verneinend). |
| 810 | **I:** Und, also, oder äh da- dass es andere Leute s- sehen konnten? |
| 811 | **AB04:** Mh-mh (verneinend). |
| 812 | **I:** Nee. |
| 813 | **AB04:** Mh-mh (verneinend). |
| 814 | **I:** Oder so etwas wie, dass Sie Bewegungen machen wollten, und das ging nicht, also es war wie blockiert? |
| 815 | **AB04:** Ne, wenn es eingeschlafen und taub ist. (I: Nur dann.) Also auch im Normalen. |
| 816 | **I:** Okay. Oder so wie gelähmt, obwohl Sie es eigentlich bewegen wollen? Das haben Sie? |
| 817 | **AB04:** Nee, habe ich nicht, nee. |
| 818 | **I:** Und wie ist es mit automatischen Handlungen, wie zum Beispiel Schuhebinden (AB04: Hm (zustimmend)) oder sich anziehen? (AB04: Hm (zustimmend) Treppen steigen, solche Sachen? Ähm laufen die ganz normal? |
| 819 | **AB04:** Äh die Treppen- Treppen kommen mir unterschiedlich hoch vor. (I: Ja?) Ja. (I: Okay.) Wie als wenn die Stufen unterschiedlich sind, wie als wenn ich mal müsste ich äh höher [MACHT GERÄUSCH AUF DEM TISCH] steigen, (I: Ja.) um die nächste Stufe zu überwinden, (I: Ja.) als bei der anderen. (I: Ah, ja.) Ja. (I: Okay.) Komisch, ja. (I: Ja.) Ja. Dabei ist es ja überall gleich. |
| 820 | **I:** Genau, hier sind sie, also zumindest soweit ich weiß, sind sie hier gleich. (AB04: Hm) Ich denke, in so ganz alten Gebäuden kann das schon sein. |
| 821 | **AB04:** Auf der ganzen Welt ist es gleich, wenn man überlegt, wie- wie knapp wir nur immer rübergehen. (I: Stimmt.) Wie äh ist es- auf der ganzen ist es gleich, Treppen sind immer gleich, (I: Ja.) oder hm sonst würde man ja stolpern. |
| 822 | **I:** Stimmt. (AB04: Ja) Ja. [HUSTET] Entschuldigung. (AB04: Kein Problem.) Aber Sie haben das Gefühl, die sind unterschiedlich hoch? |
| 823 | **AB04:** Nicht immer, aber, (I: manchmal?) habe ich schon erlebt, so etwas. |
| 824 | **I:** [HUSTET] Und ähm, dann müssen Sie darüber nachdenken, wie- dann fällt- kommt das in Ihr Bewusstsein und Sie denken darüber nach, wie steige ich jetzt Treppen? |
| 825 | **AB04:** Hm (zustimmend). |
| 826 | **I:** Sie achten dadrauf, ja? |
| 827 | **AB04:** Ja, genau, ja. |
| 828 | **I:** Okay, also es läuft (AB04: Hm (leise)) nicht mehr nur noch im Hintergrund ab? |
| 829 | **AB04:** Nee, läuft nicht mehr im Hintergrund ab. |
| 830 | **I:** Und ist es auch bei anderen Sachen, wie zum Beispiel Schuhebinden oder anderen alltäglichen Handlungen so, dass Sie manchmal das Gefühl haben, die laufen nicht mehr so automatisch ab, sondern ich muss jetzt mal kurz drauf achten, wie ging das nochmal? |
| 831 | **AB04:** Nee, eigentlich eher weniger. |
| 832 | **I:** Nur beim Treppensteigen? |
| 833 | **AB04:** Ja, das war jetzt ein gutes- nur beim Treppensteigen zum Beispiel. Ich sage ja, das war jetzt ein Beispiel, wo mir eingefallen ist (I: Ja.) und es sind auch andere Sachen schon gewesen, (I: Ja.) wo mir jetzt nur nicht einfallen. (I: Ah, okay.) Aber es ist nichts, wo jetzt immer wieder gekommen ist. (I: Hm (zustimmend)) Also sozusagen einmal erlebt, irgendwann, (I: Hm (zustimmend)) so, ja. |
| 834 | **I:** Also es fällt Ihnen nicht oft auf, (AB04: Mh-mh (verneinend)) sondern das ist so eine Das-Ist-Mal -Passiert-Sache, (AB04: Hm (zustimmend)) ne? |
| 835 | **AB04:** Hm (zustimmend). |
| 836 | **I:** Okay. [RÄUSPERT SICH] Entschuldigung. (AB04: Macht nichts) Und hatten Sie schon mal das ähm Gefühl, dass jemand das gleiche macht, wie Sie, auf der Straße zum Beispiel? Wie Sie- wie Sie nachahmt mit Bewegungen, oder so? |
| 837 | **AB04:** Nur im Normalen. |
| 838 | **I:** Mh-mh (zustimmend). (AB04: Ja.) Also so, dass es Zufall war? |
| 839 | **AB04:** Ja, genau, Zufall. |
| 840 | **I:** Okay. Okay, ähm. Lassen Sie uns noch kurz über Grenzen, eigene Grenzen zwischen Ihnen und anderen Leuten, aber auch zwischen Ihnen und der Umwelt, (AB04: Hm (zustimmend)) sprechen. (AB04: Hm (zustimmend)). Haben Sie manchmal Schwierigkeiten, sich abzugrenzen? Oder haben Sie eine klare Grenze zwischen sich und der Welt? Oder zwischen sich und der- anderen Leuten? Oder nicht so klar? |
| 841 | **AB04:** Hm (überlegend). Na eigentlich alles normal. |
| 842 | **I:** Ja? |
| 843 | **AB04:** Hm (zustimmend). |
| 844 | **I:** Ist Ihnen nichts aufgefallen? |
| 845 | **AB04:** Mh-mh (verneinend). |
| 846 | **I:** Also dass Sie manchmal das Gefühl hatten, irgendwie mit anderen Menschen zu verschmelzen, oder die Grenze ist nicht mehr so richtig da? |
| 847 | **AB04:** Nee. (I: Nee.) Ich bin mit keinem verschmolzen. |
| 848 | **I:** Nee. (AB04: Hm (zustimmend)) Und äh oder dass- also- irgendwie, wenn Sie in den Spiegel geguckt haben, Sie das Gefühl hatten, Sie wissen nicht mehr so richtig, bin ich jetzt im Spiegel oder draußen? Ich verschmelze mit meinem eigenen Spiegelbild, so etwas? |
| 849 | **AB04:** Nein, auch nicht. |
| 850 | **I:** Nein. Und wie ist es mit Körperkontakt? Gab es Momente, wo Körperkontakt unangenehm war? |
| 851 | **AB04:** Mh-mh (verneinend). |
| 852 | **I:** Nee? Also dass- manche Leute beschreiben dieses Gefühl, dass, wenn Sie jemand berührt, dass er wie in sie hineinfasst. Dass das nicht- dass die Grenze einfach nicht mehr da ist. |
| 853 | **AB04:** So etwas habe ich alles nicht. |
| 854 | **I:** Haben Sie nicht erlebt? |
| 855 | **AB04:** Mh-mh (verneinend). |
| 856 | **I:** Und ähm. Hatten Sie schon mal das Gefühl, irgendwie ähm der Welt ausgeliefert zu sein? Gleich könnte etwas Schreckliches passieren? |
| 857 | **AB04:** Hm (zustimmend). |
| 858 | **I:** Ja? Also, dass Sie irgendwie beeinflusst werden, von der Welt? |
| 859 | **AB04:** [PAUSE] Nee, aber dass es- aber das vielleicht etwas Schreckliches passiert, so, das. Da habe ich schon Angst. |
| 860 | **I:** Das sind- das sind diese, diese- |
| 861 | **AB04:** Ängste. |
| 862 | **I:** Ängste. (AB04: Ja.) Ähm, ver- verschleppt zu werden? |
| 863 | **AB04:** Ja. |
| 864 | **I:** So etwas, ne? (AB04: Hm (zustimmend)) Okay. Hm. Und- aber grundsätzlich haben Sie das Gefühl, da gibt es eine Grenze zwischen Ihnen und der Welt, und die können Sie auch irgendwie gut wahren? Und- und, die ist da, und schützt Sie auch? (AB04: Hm (schwerfällig)) Also die- Sie verschmelzen jetzt nicht mit Leuten auf der Straße, die Sie angucken? |
| 865 | **AB04:** Nein. (I: oder so) Nein. |
| 866 | **I:** Nein. Okay. Okay, letzter Bereich, (AB04: Hm (zustimmend)) ja? Dann sind wir fertig. Ähm da geht es um Ihre Weltanschauung, irgendwie (AB04: Hm (zustimmend)) Religion, ähm philosophische Themen, weltanschauliche Fragen. (AB04: Ja.) Gibt es da irgendwelche Themen, mit denen Sie sich besonders beschäftigen? |
| 867 | **AB04:** Hm, also ich bin nicht gläubig. (I: Hm (zustimmend)) Aber es gab schon Situationen im Leben, wo ich halt im- im- mit mir selber so, in meinen Gedanken, mit Gott geredet habe. (I: Hm (zustimmend)) Aber eigentlich bin ich nicht so gläubig. Hm. |
| 868 | **I:** Okay, also es gab Momente, wo Sie sich mit- oder Zeiten, wo Sie sich mehr mit Gott oder mit Religion beschäftigt haben? |
| 869 | **AB04:** Hm, beschäftigt kann man jetzt nicht- (I: nee?) nicht- nee. |
| 870 | **I:** Sondern eher so, dass Sie mal schon mal mit ihm geredet haben? |
| 871 | **AB04:** Ja, genau. |
| 872 | **I:** Und das war jetzt nicht, dass Sie sich da dann ganz viele Texte durchgelesen haben? |
| 873 | **AB04:** Nee, (I: oder) nee, (I: Filme geguckt) alles nur für mich selber. |
| 874 | **I:** Aha. (AB04: Hm (zustimmend)) Das waren dann so- wann- wann haben Sie mit Gott geredet? |
| 875 | **AB04:** Hm also am Anfang habe ich mich gefragt: Warum muss mein Leben so sein, wie es ist. Warum muss mir so etwas (I: Hm (zustimmend)) alles so passieren. (I: Hm (zustimmend)) Weil es gibt neun Milliarden auf der Welt. (I: Ja.) Warum muss ausgerechnet ich so ein Scheißleben haben? (I: Ja.) So- so etwas habe ich mich (I: Okay.) dann gefragt, ja. |
| 876 | **I:** Das haben Sie dann mit Gott besprochen, sozusagen, (AB04: Ja, genau) ja? Okay. Hm. Und gab es Situationen, wo Sie das Gefühl hatten, äußere- äußere Ereignisse stehen mit Ihnen irgendwie in Beziehung? Also das Auto fährt vorbei, weil ich hier bin, oder (AB04: Hm (zustimmend)) solche Sachen? |
| 877 | **AB04:** Hm ja so etwas habe ich sehr viel. |
| 878 | **I:** Ja? |
| 879 | **AB04:** Ja. |
| 880 | **I:** Und haben bestimmte Bedeutungen für Sie? |
| 881 | **AB04:** Hm nicht alle Sachen. |
| 882 | **I:** Nicht alle Sachen? |
| 883 | **AB04:** Hm (zustimmend). |
| 884 | **I:** Können Sie mir da ein Beispiel nennen? |
| 885 | **AB04:** Hm, nee. Das will ich nicht, weil dann habe ich Angst, dass es noch schlimmer wird. |
| 886 | **I:** Okay. (AB04: Hm (zustimmend)) Okay. Also, das ist etwas, was Ihnen jetzt auch- was Ihnen jetzt auch noch Angst macht? |
| 887 | **AB04:** Ja. |
| 888 | **I:** Dass bestimmte Dinge mit Ihnen zusammenhängen, irgendwie? |
| 889 | **AB04:** Hm (kleinlaut). |
| 890 | **I:** Ja. Okay. Dann reden wir nicht weiter drüber, ne? |
| 891 | **AB04:** Hm (kleinlaut). |
| 892 | **I:** Hm (überlegend). Haben Sie manchmal das Gefühl, das hat- das hatte ich so ähnlich schon mal gefragt, dass nur das existiert, was jetzt gerade ist? Zwischen uns beiden? Nur das ist Realität? Und Vergangenheit und Zukunft gibt es nicht mehr und so? |
| 893 | **AB04:** Nee, das eine gibt es nicht mehr, und das andere gibt es noch nicht. |
| 894 | **I:** Noch- okay. Hm. Und ist es dann wirklich so, das Gefühl, es existiert nur, was hier zwischen uns ist, oder existieren andere Sachen auch? |
| 895 | **AB04:** mh-mh (verneinend). |
| 896 | **I:** Was- was heißt "mh"? |
| 897 | **AB04:** Achso! Ähm. Nee, ist normal, äh, ja. |
| 898 | **I:** Also wir reden jetzt hier gerade, aber draußen existiert die Welt trotzdem? |
| 899 | **AB04:** Hm existiert alles trotzdem, genau. |
| 900 | **I:** Okay, okay. (AB04: Hm (zustimmend)) Oder hatten Sie mal das Gefühl, Sie haben außergewöhnliche Kräfte? Oder besondere- |
| 901 | **AB04:** Ja. (I: Fähigkeiten) In- in mei- in meiner Psychose. |
| 902 | **I:** Und was waren da so- was waren da die Fähigkeiten, oder die Kräfte, die Sie hatten? |
| 903 | **AB04:** [PAUSE] Hm. Ja, ich habe mir eingebildet, dass man mich zum besseren Menschen machen- machen will. Dass das, wo ich äh glaube, über zum Beispiel, wie ich vorhin erzählt habe, mit einer- mit einer Idee, so wie man arbeiten gehen könnte, (I: Hm (zustimmend)) dass man halbes Jahr durchhalten muss, zum Beispiel oder (I: Hm (zustimmend)) so, dass die- dass- dass- dass so etwas halt meine Kraft ist, so, anderen Leuten das mitzuteilen, wie es funktioniert, (AB04: Ahja) obwohl ich ja eigentlich selber das alles gar nicht auf die Reihe kriege. (I: Okay.) Das ähm. Genauso komisch wieder. (I: Okay.) Ja. Ganz komisch. Ich komm mir manchmal so vor, so als wenn ähm als wenn ich anderen Leuten das ähm irgendwie äh so erklären kann, wie es funktioniert, (I: Hm (zustimmend)) aber ich selber krieg es nicht hin. |
| 904 | **I:** Also Sie wissen, wie die Welt funktioniert (AB04: Ja, genau) und Sie können das anderen Leuten sagen? (AB04: Ja, genau) [HUSTET]. Wissen Sie es denn auch besser, als andere Leute? |
| 905 | **AB04:** Hm, ja, ja, genau, (I: Ja?) das meine ich ja damit (I: Ah, ja.). Ja, ich weiß es besser, als andere Menschen. (I: Okay.) Ja, obwohl es ja eigentlich gar nicht so ist, weil sonst würde ich es ja nicht- so würde ich ja nicht immer scheitern. |
| 906 | **I:** Hm, okay. Verstehe. (AB04: Hm (kleinlaut)) Also Sie hatten das Gefühl, dass Sie besser verstehen, wie es eigentlich in der Welt läuft? |
| 907 | **AB04:** Hm, ja, genau. (I: Ja?) Kleinigkeiten, wie ähm ob es jetzt zum Beispiel mal ein Fremdwort ist, oder- oder ob es große Sachen sind, wie- wie arbeiten, Urlaubsplanung (I: Hm (zustimmend)) oder so (I: Hm (zustimmend)) oder ob es riesengroße Sachen sind, wie dass zum Beispiel ein Elternteil ein Haus baut, dass man das Kind vererbt, die das wieder vermieten (I: Hm (zustimmend)) nä- nächste Haus bauen, um so die Familie zu bereichern (I: Hm (zustimmend)) und all so'ne Ideen, so etwas, dass ich so etwas kann- besser kann, als andere Menschen. |
| 908 | **I:** Okay. Das- (AB04: Ja.) das den- dass ist auch jetzt noch so? |
| 909 | **AB04:** Das ist auch jetzt noch so, ja. |
| 910 | **I:** Das- das- also Sie durchblicken das einfach besser? |
| 911 | **AB04:** Schon mein ganzes Leben habe ich (I: Okay.) das Gefühl, ja. (I: Okay.) Hm. |
| 912 | **I:** Und äh trotzdem ist es so ein, so ein Unterschied für Sie? Sie wissen das alles, aber das dann zu machen, ist dann nochmal schwierig, (AB04: [RÄUSPERT SICH]) ne? |
| 913 | **AB04:** Das schaffe ich nicht. (I: Ja?) Hm (zustimmend). |
| 914 | **I:** Okay. Ähm. Und wie ist es mit- mit magischen Ideen? Also so, dass Sie irgendwelche Dinge beeinflussen können, ähm, die passieren in der Welt? |
| 915 | **AB04:** Mh-mh (verneinend). Ja, doch! (I: Doch?) Äh-äh zum- kann ich jetzt ein Beispiel? Na wenn ich jetzt zum Beispiel ähm ein Riesenfeuer mache und ich mache das und es kommt in den Nachrichten, dann habe ich das beeinflusst. |
| 916 | **I:** Ja, okay. (AB04: Ja) Ähm. Das ist ja eine tatsächliche Handlung, (AB04: Ja, genau) die Sie dann- da- da legen Sie dann ein Feuer, und dann brennt es. (AB04: Hm, ja, genau) Hm, es gibt äh aber Leute, die beschreiben, ähm so etwas wie: "Ich denke an etwas, und dann passiert es auch." Oder ich äh kann mit meinem Gedanken irgendwie beeinflussen, dass- dass ähm in einem anderen Land irgendwas passiert, solche Sachen. |
| 917 | **AB04:** Nee, habe ich gar nicht. |
| 918 | **I:** So etwas, okay. Okay. Und ähm hatten Sie schon mal das Gefühl, irgendwie im Mittelpunkt, oder im Zentrum des Universums zu stehen? Der Welt? Dass alles sich irgendwie- |
| 919 | **AB04:** Dass alles sich um mich dreht? (I: Hm (zustimmend)) Das war in der Psychose, wo ich so extrem psychotisch war, war das alles sehr stark, (I: Ja?) ja. Jetzt ist es eher normal. (I: jetzt ist es-) Ich bin halt einer unter Millionen, (I: Okay.) ja. |
| 920 | **I:** Jetzt ist es weniger geworden (AB04: Ja, genau) und auch davor war es weniger, (AB04: Ja.) ne? Hm. Und in der Psychose ähm wie hat sich das geäußert, dass sich alles um Sie dreht? |
| 921 | **AB04:** Hm das ist eigentlich, hm. Irgendwie verstehe ich die Frage nicht. |
| 922 | **I:** Also woran haben Sie gem- m- woran haben Sie- wie haben Sie das erklärt, dass sich alles um Sie dreht in der Psychose? Woran hat- |
| 923 | **AB04:** Achso, weil ich (I: haben Sie das gemerkt-) das Gefühl gehabt habe, dass mich alle kennen, und so- (I: [HUSTET]) und so- und so (I: Ah, ja.) bin ich automatisch im Mittelpunkt. |
| 924 | **I:** Ah ja, stimmt, (AB04: Ja.) das hatten Sie gesagt. (AB04: Ja.) Genau. Hm. Okay! Ich denke, damit ähm sind wir am Ende und stelle Ihnen meine letzte Frage. (AB04: Hm (zustimmend)) Ja? |
| 925 | **AB04:** Hm (zustimmend). |
| 926 | **I:** Das ist so, dass ich am Ende immer einmal die Forschungsfrage erkläre (AB04: Hm (zustimmend)) und Sie dann gerne als Experte für die Erkrankung fragen würde, was Sie davon halten, von der Forschungsfrage. (AB04: Hm (zustimmend)) Und zwar ist die Forschungsfrage, unsere Studie, ähm ob sich die Schizophrenie auf Bewegung auswirkt und wenn ja, wie sie sich auswirkt? Und jetzt hätte ich gerne als- als Sie als Experte für Ihre eigene Erkrankung, wie würden (AB04: Hm (zustimmend)) Sie diese Frage beantworten? |
| 927 | **AB04:** Ich würd sagen: Ja, es be- äh es be- löst teilweise äh Bewegungen aus, dadurch, dass im Gehirn äh irgendwas ähm ich sag jetzt mal einen Schalter umgelegt wird ohne dass man das eigentlich denkt, so, und der Schalter aber dafür zuständig ist, den- das zu bewegen. |
| 928 | **I:** Den Finger zu bewegen? |
| 929 | **AB04:** Ja, genau, ja. |
| 930 | **I:** Okay. (AB04: Hm (zustimmend)). Also es kommen Be- es kommt zu Bewegungen, die man eigentlich nicht unter Kontrolle hat. Die man eigentlich (AB04: Hm (zustimmend)) nicht- (AB04: Ja, genau) nicht machen will. |
| 931 | **AB04:** Wie bei mir, mit dem- (I: Mit dem Zucken) ja. |
| 932 | **I:** Ja, okay. Noch irgendwelche anderen Einflüsse, von der Erkrankung auf den Körper, oder die Bewegung die Sie in ihrer Erfahrung oder Ihrer Kenntnis? |
| 933 | **AB04:** Hm ja, ich habe sie- die ganze Zeit Gef- das Gefühl schon seit Jahren, dass ich so einen Druck auf dem Kopf habe, (I: Okay.) auf dem Kopf, (I: Hm (zustimmend)) ja. So wie jetzt wenn- als wenn man irgendwie so in so einem Wahn ist die ganze Zeit, wo es die ganze Zeit so [MACHT VIBRIERENDE GERÄUSCHE] (I: Hm (zustimmend)) und- und es drückt so ein bisschen so, (I: Hm (zustimmend)) ja, und hm Leute, wo nicht erkrankt sind, haben das nicht. |
| 934 | **I:** Okay, also (AB04: Ja.) Sie spüren diesen Druck richtig? |
| 935 | **AB04:** Ja, genau, ja. |
| 936 | **I:** Ja? (AB04: Hm (zustimmend)) Den- den fühlt man im Kopf? |
| 937 | **AB04:** Ja. (I: Okay.) Ich habe schon äh gerade (I: [HUSTET]) am Anfang der- der äh gab es mal eine Situation, da habe ich- da war auch jemand, wo schizophren war, und da habe ich gefragt, warum es so ist, und der wusste gar nicht, von was ich rede. |
| 938 | **I:** Und dann hat er das- (AB04: Ja.) das Gefühl- |
| 939 | **AB04:** Weil ich wusste noch gar nicht, dass es so unterschiedlich ist bei den Menschen. (I: Ja.) Ich habe gedacht alle äh, wo schizophren sind, haben genau das gleiche wie ich. (I: Ja.) Und dass es unterschiedlich ist, wusste ich am Anfang noch gar nicht und da hatte ich mal irgendjemanden gefragt, da habe ich gesagt: "Ich habe so einen komischen Druck auf dem Kopf und hm das ist irgendwie ganz komisch. Das tut nicht weh, oder so!" (I: Hm (zustimmend)) oder so etwas. Gar nichts, oder so. Man kann mit leben, aber- aber es ist so wie als wenn man die ganze Zeit ähm im Wahn ist halt, ja. |
| 940 | **I:** Okay und äh damit ich das richtig verstehe: Also, ähm, im- im Wahn heißt, da sind viele Gedanken, und so, und drücken die Gedanken im Kopf, oder was drückt auf den Kopf? |
| 941 | **AB04:** Nee, nee! So wie jetzt hm wie jetzt wenn man jetzt mh man nimmt zwei Plastikschüsseln so mh macht und so. [MACHT VIBRIERENDE GERÄUSCHE] |
| 942 | **I:** Macht das an den Kopf? |
| 943 | **AB04:** Ja, genau. |
| 944 | **I:** Okay. Okay. Also auch so ein bisschen dumpf dann? |
| 945 | **AB04:** Ja, genau! (I: Ja?) So-so dumpf! (I: So ein) Das- das trifft (I: dumpf) es sehr gut, (I: Ja?) ja, genau, (I: Okay, okay) ja. Hm. Und ansonsten habe ich viel gesagt, das mit den Muskeln und so, das- ich weiß jetzt nicht, ob das jetzt von der Erkrankung kommt, von den Medikamten. (I: Hm (zustimmend)) Das kann ich Ihnen jetzt nicht so genau sagen. (I: Hm (zustimmend)) Aber ich würde im groben und ganzen würde ich sagen, dass das dadurch, dass die Krankheit auf das Gehirn einwirkt, und das Gehirn lenkt den ganzen Körper, (I: Ja.) würde ich sagen, ja. |
| 946 | **AB04:** Ja, okay. Alles klar. Vielen Dank, dann mache ich jetzt hier einmal aus. |

IH12_EASE_anonymized

| 1 | Interview IH12 |
| --- | --- |
| 2 | **I:** Nicht so, damit man den Tisch, wenn man den berührt, nicht (IH12: ja) hört, ne? Okay. Gut. EASE Interview, nennt sich das Interview. Examination of Anomalous Self-Experience. Also es geht um persönliche, eigene Erfahrungen von Ihnen. Ähm und ganz persönliche Erfahrungen, die bei bestimmten psychiatrischen Erkrankungen, wie zum Beispiel der Schizophrenie auftreten können. Ähm das sind oft Erfahrungen, die im klinischen Kontext nicht zur Sprache kommen oder wenig, weil es einfach nicht Zeit- nicht genügend Zeit dafür gibt. Und die im Hintergrund viel passieren und die nicht so viel besprochen werden. Deswegen kann es sein, dass ähm sich die Fragen komisch anhören oder dass Sie nicht so wirklich wissen, wie Sie es ausdrücken sollen, was Sie (IH12: Mh-h (zustimmend)) da erfahren haben. Und das können Sie- also ich will Sie bitten, Ihre ganz eigenen Worte und Ihre ganz eigene Erfahrung zu beschreiben, auch wenn es sich komisch anhört (IH12: Mh-h (zustimmend)). Oder wenn sich die Frage komisch anhört (IH12: Mh-h (zustimmend)), dann ähm wenn Sie es nicht verstehen, fragen Sie mich (IH12: Mh-h (zustimmend)), ansonsten (IH12: Mh-h (zustimmend)) können Sie auch einfach sagen: Okay, habe ich noch nie erlebt, dann gehen wir zum nächsten Punkt. (IH12: Mh (zustimmend)), (GEGENSTAND WIRD AUF TISCH ABGESTELLT). Ja? Und wenn es aber etwas gibt, was so ein bisschen so eine Glocke klingelt bei Ihnen oder das kennen Sie oder da haben Sie ähnliche Erfahrungen gemacht, dann können wir ein bisschen genauer darüber reden. Dann frage ich Sie nach Beispielen, oder wie oft das passiert (IH12: Mh-h (zustimmend)) ist und solche Sachen. Genau, es betrifft so Bereiche, wie ähm Denken, Gefühle, Körperempfindungen (IH12: Mh (zustimmend)), Selbstwahrnehmung (IH12: Mh-h (zustimmend)), so etwas (IH12: Mh-h (zustimmend)) und genau, ich nehme das auf Band auf (IH12: Mh-h (zustimmend)). Das ist okay für Sie? |
| 3 | **IH12:** Mh-h (zustimmend). |
| 4 | **I:** Ja. Ähm. Das ähm dient der reinen wissenschaftlichen Verwendung. Das habe ich schon gesagt. Und es hängt total davon ab, wie viel Sie erzählen wollen, wie lange das ganze dauert (IH12: Mh-h (zustimmend)). Wenn Sie viel zu sagen haben, viel erzählen wollen, dann dauert es länger. Wenn Sie nicht so viel reden wollen, dann nicht. Also es ist äh ganz Ihre eigene Entscheidung (IH12: Mh-h (zustimmend)) und wenn Sie Pausen wollen, haben wir schon gesagt (IH12: Mh-h (zustimmend)), können wir Pause machen (IH12: Mh-h (zustimmend)) oder auch zum Beispiel morgen Nachmittag oder so (IH12: Mh-h (zustimmend)) weiter machen. Et cetera. Genau, jederzeit unterbrechen und fragen (IH12: Mh-h (zustimmend)), wenn Sie Fragen haben. |
| 5 | **IH12:** Ja. |
| 6 | **I:** Okay dann wüsste ich einfach erstmal gerne bisschen etwas über Ihren Hintergrund. Also wir haben schon gesagt, Sie sind in [ANONYMISIERT, ORT IN DEUTSCHLAND] aufgewachsen. Wie sind Sie aufgewachsen? Haben Sie Geschwister? (IH12: Mh-h (zustimmend)) Ähm, wo sind Sie zur Schule gegangen? (IH12: Mh-h (zustimmend)). Wie war das? (IH12: Mh-h (zustimmend)), so. |
| 7 | **IH12:** Also ich bin in [ANONYMISIERT, ORT IN DEUTSCHLAND] aufgewachsen. |
| 8 | **I:** Mh-h. Ähm mit wem? Haben Sie Geschwister? |
| 9 | **IH12:** Achso. Ich habe drei Geschwister, ja. |
| 10 | **I:** Ja? (IH12: [LACHT]). Und ähm Sie sind auch in [ANONYMISIERT, ORT IN DEUTSCHLAND] zur Schule gegangen? |
| 11 | **IH12:** Ja. (I: Mh-h (zustimmend)). Ich habe einen älteren Bruder, zwei Jahre älter, und eine jüngere Schwester (I: Mh-h (zustimmend)), anderthalb Jahre jüngere. Und dann noch eine jüngste Schwester, die ist sechs Jahre jünger. |
| 12 | **I:** Okay. Und gemeinsam mit Vater und Mutter zusammen (IH12: Ja) aufgewachsen? Okay. Und in der Familie war- war guter Familienzusammenhalt oder wie- wie war das für Sie? |
| 13 | **IH12:** Ja, eigentlich schon. |
| 14 | **I:** Ja? Haben Sie noch Kontakt zu Ihren Geschwistern? |
| 15 | **IH12:** Ja (I: Ja?), ja, ja, ja. Meine Schwester, die ist jetzt am Montag Mutter geworden. |
| 16 | **I:** Ah, schön. |
| 17 | **IH12:** [LACHT]. Samstag besuche ich den [ANONYMISIERT, NAME EINES VERWANDTEN DER INTERVIEWTEN PERSON]. |
| 18 | **I:** Ah ist ein Junge? |
| 19 | **IH12:** Ja. |
| 20 | **I:** Toll, toll. Und die wohnen alle hier in der Umgebung? |
| 21 | **IH12:** Nee, leider nicht. Die wohnen in [ANONYMISIERT, ORT IN DEUTSCHLAND] (I: Mh-h (zustimmend)), die zwei Söhne. Ihr Mann. [ANONYMISIERT, ORT IN DEUTSCHLAND], wissen Sie, das ist dort bei [ANONYMISIERT, ORT IN DEUTSCHLAND]. |
| 22 | **I:** Ah, ja. Okay. Ja. Ja. Okay. |
| 23 | **IH12:** Also schon ein bisschen näher, aber man muss schon weit fahren. |
| 24 | **I:** Okay. Und Sie selbst haben auch Kinder? |
| 25 | **IH12:** Nee. |
| 26 | **I:** Nee. |
| 27 | **IH12:** Keine Kinder. |
| 28 | **I:** Okay. Und leben allein oder? |
| 29 | **IH12:** Ja, eher nicht. Das ist, wir haben mit meinen Vater damals ein Haus gebaut mit mehreren Wohnungen (I: Mh-h (zustimmend)) und da habe ich eine Wohnung (I: Okay.). Und meine Eltern wohnen unten dran. |
| 30 | **I:** Alles klar. Das heißt, Sie kümmern sich auch um Ihre Eltern? Oder Sie- |
| 31 | **IH12:** Ja, schon, aber im Moment- im Moment können sie es noch. |
| 32 | **I:** Cool. Gut. (I: [LACHT]), ja. |
| 33 | **IH12:** Gehen ja auch schon auf die 80 zu. |
| 34 | **I:** Ja. Wow. Ja, das ändert sich dann vielleicht auch irgendwann (IH12: Ja), ne? (IH12: ja) Und dann dreht sich das Rollenverhältnis. |
| 35 | **IH12:** Dreht es sich um. |
| 36 | **I:** Ja. Und jetzt hatte ich Sie vorhin gefragt, ähm wann Sie zum ersten Mal in die Klinik gekommen sind (I: Mh-h (zustimmend)), da haben Sie gesagt 92, ne? |
| 37 | **IH12:** 92. |
| 38 | **I:** Wie- wie ist das passiert? Was ist da passiert? |
| 39 | **IH12:** [ATMET SCHNAUFEND] Hm. Also ich habe erst eine Ausbildung zur Erzieherin gemacht (I: Mh-h (zustimmend)). Und da war ich im Anerkennungsjahr und da bin ich praktisch krank geworden. |
| 40 | **I:** Okay. Und wie hat sich das geäußert? Wie ist das- |
| 41 | **IH12:** [LACHT] ähm das hat sich so geäußert, dass ich gedacht habe, dass ich der Messias bin. |
| 42 | **I:** Ah, alles klar. Und dann (IH12: [LACHT]), dann haben Sie das den anderen Leuten erzählt, (IH12: [LACHT]) irgendwann hieß es- |
| 43 | **IH12:** Nee, das ging eigentlich- bei mir ging es eigentlich ziemlich plötzlich. |
| 44 | **I:** Ja? |
| 45 | **IH12:** Ja. Also, das war halt- das Anerkennungsjahr das war halt sehr stressig, da war ich im Kindergarten- mit der Erzieherin- Ich fand- ich hab die total toll gefunden, wie die das mit den Kindern macht und so. Aber die hat halt auch von mir total viel, ich weiß auch nicht, das war einfach- und dann ähm ja wie war denn das? Ja die hat dann auch immer gesagt: Meine Eltern hätten irgendwie Schuld an irgendetwas. Was weiß ich, was die damit- weiß auch nicht mehr. Und dann- |
| 46 | **I:** Die hat Sie nicht gut behandelt, die Frau? Oder? |
| 47 | **IH12:** Nee, gar nicht. |
| 48 | **I:** Was- was ist da passiert? Was hat Sie? Druck ausgeübt, oder? |
| 49 | **IH12:** Ja, das war schon ein Druck (I: Mh (zustimmend)), das war schon ein sehr großer Druck. Und dann, wo ich da war- ich wäre auch ohne den- ohne dass ich jetzt dort gewesen wäre, krank geworden (I: Mh-h (zustimmend)). Das war dann so, dass ich dann die ganze Nacht ähm gepredigt habe. |
| 50 | **I:** Okay. Wo- wo gepredigt? |
| 51 | **IH12:** In meinem Bett [LACHT]. |
| 52 | **I:** Ah, okay. |
| 53 | **IH12:** Mein Vater ist rein: "Ach Gott, [ANONYMISIERT, NAME DER INTERVIEWTEN PERSON], was ist denn jetzt mit dir los?". Meine Schwester stand hinten dran. Haben Sie mir noch Joghurt gegeben und alles. Und ich habe halt geprädigt, dass die Leute weniger Auto fahren müssen- dürfen, weil die Umwelt kaputt geht und so Zeug. |
| 54 | **I:** Was heißt gepredigt? Was- Sie haben das erzählt, oder? |
| 55 | **IH12:** Ja. |
| 56 | **I:** Ja? Okay. |
| 57 | **IH12:** Ich hab- ja, irgendwie habe ich das, irgendwie ja, gedacht, dass das alle Menschen hören, was ich jetzt sage. |
| 58 | **I:** Ah, okay. Okay. Also dass Sie sozusagen einen Lautsprecher- |
| 59 | **IH12:** Ich weiß es nicht, ich habe gedacht, ja- ja genau. Und dann, ja. Und dann, wie ich dann morgens aufgewacht bin, weil natürlich das- kam natürlich daher, dass ich mich halt bewegt habe im Bett. Aber da war das Leinentuch zerrissen (I: aha). Und dann habe ich das sofort da- darauf zurückgeführt, da ist ja auch- da ist- wie es dass Jesus gestorben ist, ist ja auch irgendwas zerrissen. |
| 60 | **I:** Ah, okay. Ich weiß gar nicht genau. Also ich kenne die Bibel (IH12: [LACHT]), aber ich kenne- ich weiß nicht ganz genau ähm was zerrissen ist, oder was nicht zerrissen ist. |
| 61 | **IH12:** Im Tempel ist irgendein Tuch zerrissen (I: Okay.) und dann habe dann auch ähm- |
| 62 | **I:** Das zusammengebracht (IH12: ja), sozusagen. |
| 63 | **IH12:** Ja, genau. Und dann bin ich dan run- ich hatte vorher auch ganz viel aufgeschrieben, was mich halt so in der Kindheit belastet hat und so. Und dann habe ich dann gedacht: Ah, ich habe das jetzt alles überwunden und so. Und dann habe ich den ganzen Zettel zerrissen und in die Mülltonne. Und dann war es alles ganz hell (I: Mh-h (zustimmend)). Also ich habe alles ganz hell gesehen. |
| 64 | **I:** Die Welt generell? |
| 65 | **IH12:** Ja, ja. |
| 66 | **I:** Okay. Okay. |
| 67 | **IH12:** Von der Farbe her. |
| 68 | **I:** Ja? Intensiver? |
| 69 | **IH12:** Ja. |
| 70 | **I:** Ja. Ja. Und ähm das war jetzt nur direkt davor oder das hat sich langsam angekündigt? |
| 71 | **IH12:** Nee, das ging ganz schnell (I: ganz schnell). Am Wochenende vorher war ich noch gesund, da war ich- habe ich noch eine Freund besucht in [ANONYMISIERT, ORT IN DEUTSCHLAND]. Und, und, und Mittwoch war es glaube ich, dass ich in die Klinik kam (I: Okay.). Und- und, wie war das denn? Ja. Ja, meine Mutter, die hat sich natürlich totale Sorgen gemacht um mich. Die hat mich dann zu [ANONYMISIERT, NAME EINES ARZTES] Psychiater- (I: Mh-h (zustimmend)) [LACHT], hat die mich dann dorthin gebracht. Ist damals noch Auto gefahren (I: Mh-h (zustimmend)). Hat mich in meinem Auto dahingebracht. Und- und dann saß ich da im Wartezimmer, und dann saß halt so eine da, die saß so. Und dann habe ich zum Psychiater gesagt: Ja, ich gehe jetzt mal mit der in den Raum. Viertelstunde, und dann werde ich die heilen. |
| 72 | **I:** Ah. Alles klar (IH12: [LACHT]). Also Sie hatten besondere Kräfte? |
| 73 | **IH12:** Genau |
| 74 | **I:** Ja (IH12: ja, ja, ja). Alles klar, okay. |
| 75 | **IH12:** Und dann, hat der gesagt, [ANONYMISIERT, NAME EINES ARZTES], hat er gesagt: Jetzt kommen Sie mal zu mir rein. Und ich habe natürlich gemerkt, dass meine Mutter total aufgeregt war (I: Ja.). Habe gesagt: Bin ja gar nicht wegen mir da, ich bin ja wegen meiner Mutter da! Gucken Sie mal, wie aufgeregt die ist! [LACHT] (I: Ja, alles klar). Und dann hat der [ANONYMISIERT, NAME EINES ARZTES] dann zu meiner Mutter gesagt: Fahren Sie- fahren Sie nach [ANONYMISIERT, ORT IN DEUTSCHLAND] mit der, da kann ich nichts mehr machen [LACHT]. |
| 76 | **I:** Okay. Und da waren Sie dann zum ersten Mal (IH12: ja) auch direkt hier auf- |
| 77 | **IH12:** [ANONYMISIERT, NAME EINER ÖRTLICHEN PSYCHIATRISCHEN STATION], war ich da |
| 78 | **I:** [ANONYMISIERT, NAME EINER ÖRTLICHEN PSYCHIATRISCHEN STATION], okay, verstehe. |
| 79 | **IH12:** Und dann hat ähm meine Mutter die war dann so ganz aufgeregt im Auto, weil wir nicht genug Benzin hatten (I: Oh). Also sie hatte die ganze Zeit Angst, dass sie stehenbleibt (I: [LACHT]). Und ich habe die ganze Zeit mit Gott geredet, und der hat mir gesagt: Nee, nee, wir bleiben nicht stehen, keine Angst. Ich hab halt mich mit Gott unterhalten. |
| 80 | **I:** Alles klar, Sie haben Gott als Stimme in Ihrem- in Ihrem Kopf- |
| 81 | **IH12:** Ich habe, ja, aber nicht gehört, sondern einfach in Gedanken. |
| 82 | **I:** Okay. Also Sie haben jetzt nicht- |
| 83 | **IH12:** [crosstalk] nicht akustisch (I: nicht akustisch, okay). Aber halt so, wie man denkt (I: Mh-h (zustimmend)), habe ich den gehört. |
| 84 | **I:** Mh-h. Okay, das heißt, Sie hatten das Gefühl, Ihre Gedanken sind nicht Ihre eigenen Gedanken, sozusagen? |
| 85 | **IH12:** Nee, das waren schon meine Gedanken. Das war ein Gegenüber von mir. |
| 86 | **I:** Okay. Also Sie haben mit einem Teil von sich gesprochen (IH12: Ja), sozusagen. |
| 87 | **IH12:** Genau (I: Okay). Nur dass ich halt diesen Teil als Gott dann gesehen habe. Und ich habe es dann auch gar nicht kapiert, als ich in die Psychiatrie gekommen bin. In [ANONYMISIERT, NAME EINER ÖRTLICHEN PSYCHIATRISCHEN STATION], das hieß glaube ich damals noch [ANONYMISIERT, NAME EINER ÖRTLICHEN PSYCHIATRISCHEN STATION]. |
| 88 | **I:** Mh-h. 92. [ANONYMISIERT, NAME EINER ÖRTLICHEN PSYCHIATRISCHEN STATION]. Also, ähm wir sind jetzt schon direkt reingesprungen in den Bereich von Kognition, Gedanken (IH12: Mh-h (zustimmend)). Alles was so innerlich in einem abläuft. Ähm und Sie haben gesagt, Sie haben innere Gedanken, die nicht Teil von sich waren, oder die- oder die ähm- |
| 89 | **IH12:** Nee, die waren nicht- die haben ja mit mir gesprochen. |
| 90 | **I:** Genau, also die, sozusagen, dass Sie selbst verschiedene Teile hatten, kann man das sagen? |
| 91 | **IH12:** Ja |
| 92 | **I:** Ja? |
| 93 | **IH12:** Ja. Kann man eigentlich sich glaube ich schwer- schwer vorstellen, ne? |
| 94 | **I:** Ja, sehr schwer von außen bestimmt (IH12: [LACHT]), aber das beschreiben- beschreiben einige, tatsächlich, die- die- das- die eine Psychose oder eine Schizophrenie erlebt haben. Deswegen ist es auch hier mit aufgeführt in unserem Interview. Ich gucke gerade. |
| 95 | **IH12:** Also ich habe sie nicht- ich habe sie nicht laut gehört in meinem- zum Beispiel, ich habe eine- meine Patentante, mit der ich aber keineswegs verwandt war, die hatte auch Psychose (I: Mh-h (zustimmend)), und die hat dann, wenn sie so saß, hat sie im Nachbarhaus Stimmen gehört. Also so etwas habe ich nicht gehabt. |
| 96 | **I:** Nee. Okay. Also es waren Teile von Ihnen (IH12: Mh-h (zustimmend)) selbst, die sozusagen miteinander (IH12: gesprochen) sich gesprochen, sich unterhalten haben. Und das haben Sie (IH12: nee, gehört nicht) nicht gehört, sondern einfach gewusst, dass das so- |
| 97 | **IH12:** Also bei mir ist das so, wenn ich denke, dann höre ich in mir drin eine Stimme, also nicht akustisch laut (I: Mh-h (zustimmend)), sondern- auch wenn ich lese, dann ist so als wenn mir jemand etwas vorliest. |
| 98 | **I:** Okay. Okay. Verstehe. Ja. |
| 99 | **IH12:** Meine Mutter hat mir halt früher immer viel vorgelesen (I: Okay.) und wenn ich etwas lese, das ist dann immer die Stimme meiner Mutter, wenn ich lese. |
| 100 | **I:** Ah, alles klar. Okay. Und ähm könnten Sie diese- diese Teile von sich selbst, können Sie die verorten irgendwo? Von damals, also konnten Sie die verorten irgendwo im Kopf, oder im Körper? Wenn nicht, kein Problem, dann sagen Sie einfach nein. |
| 101 | **IH12:** Also ich denke das mir, so- so über mir halt. |
| 102 | **I:** Mh-h. Also ein Teil ist auf der einen Seite und einer auf der anderen Seite, oder egal? |
| 103 | **IH12:** Nee, ich bin ja ich (I: Mh-h (zustimmend)) und so oben da würde ich mal sagen. |
| 104 | **I:** Achso. Das heißt, diese zusätzlichen Teile sind oben drüber? |
| 105 | **IH12:** Ist ja auch schon so lange her, ich muss mal [LACHT] überlegen. |
| 106 | **I:** Nicht schlimm. War das auch nur einmalig, und auch nur in der Psychose? Oder gibt es manchmal Momente, wo das immer noch- wo dieses Gefühl noch aufkommen könnte? |
| 107 | **IH12:** Das war nur in der Psychose. |
| 108 | **I:** Nur in der Psychose. Okay. |
| 109 | **IH12:** War nur in der Psychose. |
| 110 | **I:** Ja. |
| 111 | **IH12:** Ja, ja, ich, damals, da- ich habe als Kind immer darauf gewartet, dass irgendwann der Jesus wieder auf die Welt kommt (I: Mh-h (zustimmend)). Weil es schon so- also ich bin ja in den achtziger Jahren groß geworden mit Tschernobyl und wo das mit der Umwelt so schlimm war, und alles (I: Ja.). Und wie ich dann- da habe ich mir- da habe ich mir so gesagt: [ATMET SCHARF EIN] ich habe nicht gedacht, dass ich das machen muss [LACHT]. (I: Ja.) Der Mess- also- |
| 112 | **I:** Dass Sie- dass Sie sozusagen diese Umwelt verbessern müssen? |
| 113 | **IH12:** Ja, dass ich- dass ich als Messias- dass ich es bin. |
| 114 | **I:** Achso. Und das war natürlich eine große Belastung? (IH12: Ja) wenn man so etwas- wenn man so etwas- so eine Aufgabe plötzlich bekommt. |
| 115 | **IH12:** Ja, ja [LACHT]. |
| 116 | **I:** Ja, Wahnsinn. Okay, kommen wir zurück zu den Gedanken (IH12: Ja). Mh. Haben Sie manchmal das Gefühl, Ihre Gedanken nicht so gut nachvollziehen zu können? Oder dass die unterbrochen werden? Oder dass andere Gedanken dazwischen kommen und Gedanken, die Sie gerade denken, unterbrechen? |
| 117 | **IH12:** Mh-h (verneinend). |
| 118 | **I:** Nee? Also dass es so ein Durcheinander im Kopf gibt? |
| 119 | **IH12:** Achso. Ja, aber das ist denke ich ganz normal. Wenn ich jetzt bei der Arbeit bin, und das eine mache, dann denke ich: Das und das muss ich noch machen. Und manchmal hilft es mir dann, dass ich einfach aufschreibe (I: Ja.). Und wenn ich dann mit dem einen fertig bin, streiche ich es durch und dann gucke ich mir die Liste an, und überlege, was ist jetzt als nächstes wichtig, und dann mache ich dann das. |
| 120 | **I:** Genau, genau, klar. Wie- wie- wie jeder sich das macht. |
| 121 | **IH12:** [crosstalk] wird jeder machen (I: ja, ja?) genau. Also das ist (I: aber das ist jetzt nicht so, dass-) das ist jetzt würde ich jetzt nicht sagen krankhaft. |
| 122 | **I:** Nee, das also- nee. Nein, nein. Ich- es geht hier auch um einfach grundlegende (IH12: ah, ja) Erlebnisweisen. Muss nicht alles äh irgendwie gewertet werden (IH12: Mh-h (zustimmend)). Oder krankhaft ist jetzt (IH12: Mh-h (zustimmend)) so wertend, ne? |
| 123 | **IH12:** Ja, ja. |
| 124 | **I:** Ähm. Nee, aber ähm auch nicht, wenn Sie jetzt einen Gedanken- oder gerade arbeiten, oder so, dass dann so Gedanken dazwischen schießen, und Sie stören richtig? Das passiert nicht? |
| 125 | **IH12:** Nee, das habe ich (I: Okay.) zum Glück nicht. Zum Glück nicht, nee (I: Ja.). Aber was bei mir in der Psychose war, da war alles hundertprozentig logisch. |
| 126 | **I:** Ja? Meistens ist das so, ne? Dass- dass die Dinge dann für einen selber logisch wirken, aber von außen [crosstalkt] ganz schwierig nachvollziehen. |
| 127 | **IH12:** Ich musste da ins [ANONYMISIERT, ORT IN DEUTSCHLAND], da haben sie meinen Kopf geröntgt (I: Ja.), weil man wusste ja nicht, ob es vielleicht auch ein Tumor (I: Ja.) sein könnte (I: Ja, ja). Und dann habe ich der Schwester, die mich da hochgebracht hat, so alles erklärt: Und so ist das, und so und so und so. Und die hat dann gesagt: Frau [ANONYMISIERT, NAME DER INTERVIEWTEN PERSON], Sie haben bestimmt recht, aber ich kann Ihnen leider nicht folgen [LACHT]. |
| 128 | **I:** [LACHT] Das wissen Sie noch, ja? Das- das ist, ja- das ist natürlich auch total schwierig, wenn einen dann niemand versteht, ne? Wenn man versucht, etwas zu erklären? Und die Logik des Ganzen zu erklären? |
| 129 | **IH12:** Und meine- und meine Mutter, die- der habe ich das ja auch alles so erklärt. Die hat dann schon selbst gedacht, ob sie jetzt falsch denkt, oder ob ich [LACHT]. |
| 130 | **I:** Ja? Natürlich, das kommt bei einem oft dann in dem Moment, klar. Vor allem, wenn man das nicht kennt. Wenn man die- und es ist ja auch wichtig, die- diese Erlebnisse nachzuvollziehen, auf eine gewisse Art und Weise. Das ist auch Teil von so einem phänomenologischen Interview, dass wir versuchen zu verstehen, was ist da eigentlich- passiert auch auf der eigenen Erfahrungsebene? (IH12: Hm (zustimmend)). Ähm zu den Gedanken (IH12: Mh-h (zustimmend)): Sie hatten erzählt, dass Sie auch Gedanken hatten, von denen Sie das Gefühl haben, die gehören nicht unbedingt zu Ihnen. Also die waren fremd oder nicht nur sie selbst. Kann man das so sagen? |
| 131 | **IH12:** Da, wo ich mit Gott gesprochen habe? |
| 132 | **I:** Mh, zum Beispiel. Oder vielleicht auch zu anderen Zeitpunkten. Also meine Fragen beziehen sich im Prinzip auf die Zeit vor, während, und nach der Psychose (IH12: Mh-h (zustimmend)) und auch jetzt vielleicht, je nachdem. |
| 133 | **IH12:** Mh-h. Was war jetzt nochmal gefragt? |
| 134 | **I:** Ob es Gedanken, manchmal, oder früher Gedanken gab, die ähm, sich nicht unbedingt- die nicht unbedingt zu Ihnen gehören? Die Ihnen fremd vorkommen? Nicht unbedingt Ihre eigenen Gedanken sind? Oder waren? |
| 135 | **IH12:** Hm (überlegend). Ja, also, aber eigentlich- ja, in der Psychose schon (I: Mh-h (zustimmend)). Ich- ich konnte ja nicht beeinflussen, was er jetzt zu mir sagt. |
| 136 | **I:** Ja, ja. Aber das war ausschließlich während der Psychose (IH12: ja), so? [crosstalk] |
| 137 | **IH12:** Sonst hatte ich es nicht. Nur in der Psychose gehabt . |
| 138 | **I:** Okay. Und bei den anderen Episoden, ist es dann auch wieder so gewesen? |
| 139 | **IH12:** [LACHT]. Mal überlegen. Ich glaube, das war nur das erste Mal- |
| 140 | **I:** Nur das erste Mal, dass Sie Gedanken hatten, die nicht zu Ihnen gehören? Okay. Wie haben sich die anderen Psychosen- |
| 141 | **IH12:** Da habe ich das ja eigentlich da schon gewusst. Ja. Wobei ich da auch schon wieder gedacht habe, dass ich der Messias bin, also komisch (I: Mh-h (zustimmend)). Also, ich hatte dann ja auch die feste Überzeugung, dass jetzt zum Beispiel auch Weihnachten gar nicht mehr am 24. Dezember ist, sondern am [ANONYMISIERT, DATUM]. Da habe ich nämlich Geburtstag. |
| 142 | **I:** Ah (IH12: [LACHT]). Ja. Sie haben es auf sich selber bezogen, sozusagen, auf Ihre- auf Ihren eigenen Geburtstag? |
| 143 | **IH12:** Ja. |
| 144 | **I:** Ja. Okay. Das war dann in den späteren Psychosen? |
| 145 | **IH12:** [RÄUSPERT SICH] Da- da habe ich dann ja schon dazu gelernt gehabt. Da wusste ich dann, dass Weihnachten am- am 24. ist, aber ich habe trotzdem noch- [LACHT] |
| 146 | **I:** Okay. Ja, klar. Ähm. Das ist ja häufig so, ne? Leu- Menschen, die- die dann mehrere Erfahrungen machen, erleben das trotzdem, obwohl sie rational wissen, dass es nicht so sein kann, vielleicht. (IH12: Mh (zustimmend)). Ja? War das bei Ihnen auch so? |
| 147 | **IH12:** Ja, wahrscheinlich. |
| 148 | **I:** Ja? Okay. |
| 149 | **IH12:** Ja, beim zweiten Mal hatte ich- da hatte ich dann plötzlich eher so ein- das war auch Zufall. Da hatte ich dann plötzlich da so eine- so einen Punkt, so einen Blutpunkt. |
| 150 | **I:** Am Fuß? |
| 151 | **IH12:** Am Fuß. Beim Duschen habe ich das gesehen. |
| 152 | **I:** Okay. Der war auch wirklich da. Den haben andere auch gesehen? |
| 153 | **IH12:** Ich habe das niemandem gezeigt. |
| 154 | **I:** Okay. Und was- |
| 155 | **IH12:** Und das war natürlich dann wieder Jesus am Kreuz [LACHT]. |
| 156 | **I:** Achso, verstehe, okay. Ja. Aber an den Händen hatten Sie keins? |
| 157 | **IH12:** Nein, da hatte ich nichts. Ich hatte das nur an den- an den- aber ich habe es, ich habe es wirklich (I: Okay.). So groß und- und- und es ist dann auch von alleine weggegangen. Ich habe es auch niemandem gezeigt, weil ich dachte, wenn es irgendwas Schlimmes gewesen wäre (I: Ja.), wäre es ja auch bisschen blöd gewesen. |
| 158 | **I:** Ja. Also selbst den Ärzten haben Sie es nicht (IH12: nee) gezeigt? Okay. Okay. Ähm ist es manchmal so, dass Sie Gedanken haben, die schneller werden oder so rasen? Oder ähm äh drängen? Sagt man auch. |
| 159 | **IH12:** Das kenne ich, wenn ich nachts im Bett liege und mir dann die Gedanken durch den Kopf rattern und rattern (I: Okay.). Und dann sage ich mir so: Das hast du jetzt gedacht, das ist jetzt mal weg. Und dann kommt der nächste Gedanke, und denke ich so: Das hast du jetzt auch gedacht, weg mit [LACHT]. |
| 160 | **I:** Und das funktioniert? |
| 161 | **IH12:** Manchmal geht es, ja. |
| 162 | **I:** Ja? Was ähm sind das Gedanken, die Sie- also was passiert dann? Sie denken den Tag durch? Oder ähm was denken- was durchdenken Sie abends, wenn Sie im Bett liegen? |
| 163 | **IH12:** [SCHNAUFT] ja was ich alles noch so zu tun habe (I: Hm (zustimmend)) am nächsten Tag, ja. Oder auch, dass ich denke, ich habe bei der Arbeit etwas falsch gemacht. Und dann denke ich immer wieder darüber nach (I: Mh-h (zustimmend)) ob das jetzt auch wirklich falsch war. |
| 164 | **I:** Und schaffen Sie es sich davon zu lösen, dann? Oder ist dann schwer- |
| 165 | **IH12:** Nee, irgendwann, irgendwann schlafe ich dann ein. |
| 166 | **I:** Okay. Das geht? |
| 167 | **IH12:** Mh-h (zustimmend) (I: Ja.). Ich nehme auch meine Tabletten. Wenn ich meine Tabletten genommen habe, eine halbe Stunde ist es so, dass ich gerade noch so ins Bett laufen kann. |
| 168 | **I:** Okay. Und das ist gut so? Oder? |
| 169 | **IH12:** Naja, also unter Schlaflosigkeit leide ich nicht. |
| 170 | **I:** Das ist- das ist gut, oder? |
| 171 | **IH12:** Eigentlich schon (I: Ja.). Wenn andere stundenlang im Bett liegen und nicht einschlafen können (I: Mh-h (zustimmend)), ist auch blöd (I: Mh-h (zustimmend), voll, ja) und dann noch denken müssen: "Ich muss morgen um fünf aufstehen." |
| 172 | **I:** Okay. Sie stehen immer um fünf auf? |
| 173 | **IH12:** Nein. Wenn andere Leute- ich habe eine Kollegin gehabt, die hat morgens um fünf aufstehen müssen und ist eins nicht eingeschlafen (I: ja, furchtbar, ja) und hat sich die ganze Zeit gedacht: Oh Gott, ich muss gleich aufstehen und (I: Ja.) ich kann nicht schlafen (I: Ja.), habe einen ganzen Arbeitstag vor mir. |
| 174 | **I:** Ja, das ist schlimm. Und dann wird es immer schlimmer, weil man denkt- weil man sich noch mehr Stress macht, dass man nicht einschlafen kann. |
| 175 | **IH12:** Und dann macht man sich noch mehr Stress (I: genau) und da habe ich richtig Glück, dass ich morgens erst um halb neun aufstehe (I: ja, okay), weil ich ja erst um zwölf anfange zu arbeiten (I: ja, das ist gut). Also in der Richtung habe ich schon mal Glück gehabt. |
| 176 | **I:** Ja. Und wenn Sie dann so nachts- wir nennen das ja auch grübeln, ne? (IH12: Grübeln, ja, ja.) Sich viele Gedanken machen, den Tag durchdenken, was habe ich bei der Arbeit richtig, was habe ich falsch gemacht. Ähm sind das Gedanken, die Sie dann nicht loslassen manchmal? Oder wo Sie sich auch denken: Das müsste ich eigentlich gar nicht denken? |
| 177 | **IH12:** Ja. [PAUSE]. Ja eigentlich- eigentlich habe ich das jetzt auch schon länger nicht mehr gehabt, so richtig Stimmen. Ich bin jetzt auch schon 19 Jahre beim [ANONYMISIERT, NAME EINES ARZTES]. |
| 178 | **I:** Wie lang? |
| 179 | **IH12:** 19. |
| 180 | **I:** 19. Ah ja. |
| 181 | **IH12:** Und irgendwann ist es ja alles so, dass ich dann weiß, es ist richtig. |
| 182 | **I:** Ja. Aber- |
| 183 | **IH12:** Aber es ist trotzdem jeden Tag wieder irgendetwas, was ich falsch mache. |
| 184 | **I:** Okay, Sie haben jeden Tag das Gefühl, Sie haben etwas falsch gemacht? |
| 185 | **IH12:** Eine Sache mindestens (I: Okay.). Also jetzt nicht von der Arbeit, die ich mache, sondern auch von dem Zwischenmenschlichen. Man sagt ja dann doch etwas Blödes, und dann ist- ist der sauer und- und dann, mein Problem ist das, dass ich zu viel frage. |
| 186 | **I:** Okay. Das sagen Ihnen die Leute oder das ist Ihr eigenes Gefühl oder? |
| 187 | **IH12:** Das ist tatsächlich so (I: Mh-h (zustimmend)). Was weiß ich, die eine Kollegin, die frage ich dann irgendwas, und dann- und dann sage ich gleich: Oder soll ich es lieber doch so machen? Und dann ist schon immer ganz ungeduldig und sagt: Frau [ANONYMISIERT, NAME DER INTERVIEWTEN PERSON], warum fragen Sie mich, wenn Sie es sowieso anders machen? [LACHT] |
| 188 | **I:** [LACHT]. Und dann denken Sie abends darüber nachen, ob das falsch war? |
| 189 | **IH12:** Ja. |
| 190 | **I:** Ja? Okay. |
| 191 | **IH12:** Also das ist echt so. Deshalb bin ich ja auch wirklich, bei mir- oh ich bin mit so einer netten Kollegin zusammen. Und die frage ich dann natürlich auch. Und dann- und dann denke ich, und- und irgendwie, ach das ist dann blöd, dann kommt dann die andere Kollegin: Ach Gott, die Frau [ANONYMISIERT, NAME DER INTERVIEWTEN PERSON], hat mich schon wieder etwas gefragt. Ach ja, dich hat sie auch etwas gefragt. [LACHT] |
| 192 | **I:** Achso, das heißt, Sie haben das Gefühl, die Leute reden über Sie, oder? |
| 193 | **IH12:** Ja. Das habe ich schon oft, das Gefühl. (I: Und ähm-) Und einmal, da war ich dann, ähm bin ich da- war ich dort in der Nähe gestanden. Da habe ich auch gedacht, die reden über mich. Und da habe ich gefragt: Aber, haben Sie das gerade von mir, und so? Haben die gesagt: Ach Frau [ANONYMISIERT, NAME DER INTERVIEWTEN PERSON], wollen wir über Sie reden, dann gucken wir einfach, dass Sie nicht nebendran stehen [LACHT]. |
| 194 | **I:** Und das hat Sie dann beruhigt, oder? |
| 195 | **IH12:** Ja. |
| 196 | **I:** Mussten Sie dann trotzdem darüber nachdenken, was Sie falsch gemacht haben? |
| 197 | **IH12:** Ja, also, deswegen ist es auch ein Grund, deswegen, dass ich sonntags immer in die Kirche gehe, einfach um- ich mache ständig irgendwelche Fehler, und einfach um das loszulassen und zu wissen, jetzt geht es wieder von vorne los. |
| 198 | **I:** Okay. Sie sind katholisch? |
| 199 | **IH12:** Evangelisch. |
| 200 | **I:** Evangelisch. Also, ich hatte jetzt gedacht, weil man da, in der katholischen Kirche kann man ja die Beichte (IH12: Beichte, ja) abgeben, so etwas. |
| 201 | **IH12:** Nee, aber, es ist ja in jedem Gottesdienst so, dass- dass das damit anfängt. |
| 202 | **I:** Dass man sozusagen betet und (IH12: ja) und seine eigenen Sachen (IH12: Segen kriegt und so) mh, genau, ja. Das heißt, wenn ich das noch mal zusammenfassen darf, Sie machen sich viele Gedanken über Ihr eigenes Verhalten? |
| 203 | **IH12:** Ja. |
| 204 | **I:** Habe ich die richtig gemacht? |
| 205 | **IH12:** Ja. |
| 206 | **I:** Habe ich die Sachen falsch gemacht? |
| 207 | **IH12:** Ja, ja. |
| 208 | **I:** Okay. Okay. Und haben Sie manchmal auch Gedanken, die Ihnen unsinnig oder fremd vorkommen? Also solche Gedanken, die sich immer wieder wiederholen? Die Sie nicht gehenlassen können? Haben Sie das Gefühl, das ist unsinnig oder das ist wichtig, was Sie- |
| 209 | **IH12:** Was ich denke? (I: Mh-h (zustimmend)) [crosstalk]. Eigentlich ist es schon (I: schon wichtig, sich-), ja. |
| 210 | **I:** Ja, genau. Und müssten Sie dann auch- also gibt es Momente, wo Sie anhand dieser Gedanken handeln müssen? Irgendwas tun müssen? (IH12: Achso) Zum Beispiel fragen müssen äh ähm: Reden Sie gerade über mich? Oder (IH12: Mh-h (zustimmend)) oder ähm ist das also- gibt es Handlungen, die sich daran anschließen? |
| 211 | **IH12:** Ja, also manchmal, wenn meine Kollegin dann da so ist, ach Gott, da- da habe ich ja fast geheult. Die eine Kollegin, die ist ähm manchmal so ein bisschen ?(mürrisch)? (I: Mh-h (zustimmend)), und dann, also dass sie frei hatte einen Tag und dann haben wir festgestellt, dass kein Münzgeld mehr da ist. Und wenn die [ANONYMISIERT, NAME EINER KOLLEGIN] im Urlaub ist, dann bin ich dafür zuständig, Münzgeld zu bestellen (I: Münzgeld, okay). Und dann habe ich mit der Frau [ANONYMISIERT, NAME EINER KOLLEGIN] gesprochen. Und dann hat die gesagt: Eigentlich- Frau [ANONYMISIERT, NAME EINER KOLLEGIN] hat das eigentlich im Griff. Und dann hatten wir- das ist so, wie ich bin, gehe ich zu anderen Kolleginnen und frage die auch noch, und dann hat die gesagt: Man könnte ja mal ähm unverbindlich bei der Bank anrufen, ob Geld bestellt ist. Wenn da nichts bestellt ist, dass ich dann bestelle. Achso Entschuldigung, ich arbeite ja bei so einer Firma, wo unten Verkauf ist (I: Mh-h (zustimmend)) und die brauchen dann immer Münzgeld (I: Okay.). Und ich bin halt dafür zuständig, wenn die Kollegin im Urlaub ist (I: Ja, ja). Und dann brauchen die immer viele Zwei-Eurostücke und viele Ein-Eurostücke. Und da war definitiv nichts in der Schublade (I: Ja.) und es war auch kein Zettel gelegen, dass irgendetwas bestellt ist (I: Okay.). Und dann hatte er dann bei der Bank gesagt: Nee ist nichts bestellt. Deshalb: Okay gut, dann bestelle ich jetzt mal bei Ihnen Geld. Und dann war die [ANONYMISIERT, NAME EINER KOLLEGIN] am nächsten Tag stinkesauger [/stinkesauer/] auf mich: Frau [ANONYMISIERT, NAME DER INTERVIEWTEN PERSON], wenn ich da bin, brauchen Sie kein Geld zu bestellen. Ich habe noch in der Schublade total viel Münzen, die wollte ich jetzt erstmal einlösen und so. Und dann habe ich zu ihr dann gesagt: Ja, gut, dann gebe ich Ihnen halt das Geld, und dann geben Sie mir halt das Münzgeld (I: Mh-h (zustimmend)). Ich habe ja immer genug Geld dabei, weil ich ja immer- und bei mir geht das alles so plötzlich. Es kann passieren, dass ich jetzt noch zu Hause bin und morgen in [ANONYMISIERT, ORT IN DEUTSCHLAND]. Deswegen habe ich auch immer etwas Geld dabei. |
| 212 | **I:** Wofür brauchen Sie das Geld? |
| 213 | **IH12:** Ja, wenn ich in [ANONYMISIERT, ORT IN DEUTSCHLAND] bin, brauche ich doch Geld. |
| 214 | **I:** Da braucht man Geld in [ANONYMISIERT, ORT IN DEUTSCHLAND]? (IH12: [LACHT]) Wofür braucht man da Geld? [LACHT] Was macht man mit dem Geld in [ANONYMISIERT, ORT IN DEUTSCHLAND]? |
| 215 | **IH12:** Äh. Ja. Eigentlich haben Sie mir alles abgenommen. Mein Vater hat mir da Münzen gebracht, dass ich dann daheim anrufen kann (I: Mh-h (zustimmend)) und er mich dann zurückruft. Aber ich habe halt irgendwie ein ruhigeres Gefühl. Es kann ja auch passieren- passiert, ich bin gestürzt und komme plötzlich in die Klinik, da brauche ich aber auch ein bisschen Geld. |
| 216 | **I:** Okay. Okay. Also es gibt Ihnen ein gutes Gefühl, Geld dabei zu haben? |
| 217 | **IH12:** Ja. |
| 218 | **I:** Ja. Völlig in Ordnung (IH12 und I: [LACHEN])). Jeder hat so seine Sachen, die ihm ein gutes Gefühl geben. |
| 219 | **IH12:** Also es ist jetzt nicht so viel. Also wenn es geklaut wird, könnte ich es auch verschmerzen. Und dann habe ich das der [ANONYMISIERT, NAME EINER KOLLEGIN] gesagt, und dann hat Sie gesagt: Ach was, Quatsch, ist schon okay. Dann wechsel ich das halt nächste Woche. So hat es dann geklappt. Aber ich habe (I: Aber) echt, als ich oben saß, ich habe echt geheult. Und dann hat die [ANONYMISIERT, NAME EINER KOLLEGIN] zu mir gesagt: Ach Frau [ANONYMISIERT, NAME DER INTERVIEWTEN PERSON], jetzt nehmen Sie es sich doch nicht so Herzen. Sie haben es doch nur gut gemeint. Ich habe es doch auch wirklich nur gut gemeint. |
| 220 | **I:** Ja. Das heißt, Sie haben sich schuldig gefühlt (IH12: ja) für irgendwas, weil Sie- weil Sie etwas falsch gemacht haben? |
| 221 | **IH12:** Ja (I: sozusagen, ja). Und die ist halt oft sauer. Aber das- ich darf es nicht immer auf mich beziehen. Ich habe Sie dann einfach gefragt: Ach Gott, Frau, haben Sie irgendwas, habe ich irgend- haben Sie irgendwas? Sind sie irgendwie sauer auf mich? Und so. Und dann hat sie gesagt: Nee, nee, nee, das hat gar nichts mit Ihnen zu tun. Die hat halt viel Arbeit (I: Mh (zustimmend)) und ist dann gestresst (I: Mh (zustimmend)). Sie ist Chefsekrätarin (I: Mh (zustimmend)) und- und sie sitzt da am Empfang und hat halt wirklich viel zu tun (I: Okay.). Ich darf das dann echt nicht auf mich beziehen (I: Ja.). Aber ich beziehe das halt immer auf mich. Auch beim Tennis beziehe ich das auf mich, wenn die lachen. Dann denke ich auch, die lachen über mich. Deswegen habe ich jetzt mit der Trainer- mit der Trainingsgruppe habe ich jetzt auch aufgehört. Deswegen war ich echt immer- und das hatte auch keinen Sinn gehabt. Weil ich dann auch immer gemerkt habe, die wollen nicht mit mir spielen, weil ähm die inzwischen viel besser geworden sind als ich (I: Okay.). Die haben halt den ganzen Winter trainiert. |
| 222 | **I:** Okay und Sie waren im Winter nicht da? |
| 223 | **IH12:** Nee, ich war da nicht dabei im Training (I: Mh-h (zustimmend)). Ich komme da nicht hin nach hin nach [ANONYMISIERT, ORT IN DEUTSCHLAND] (I: Okay.). Ich habe im Auto viel zu viel Angst, um bei Schnee dann immer diesen Buckel zu fahren. |
| 224 | **I:** Okay. Ich schreibe mir das eben mal kurz auf (IH12: Ja) und dann kommen wir da nochmal gleich dazu (IH12: achso, ja) und ähm damit wir das nicht vergessen, was Sie gerade- Tennis und Auto habe ich aufgeschrieben. Kommt es manchmal vor, dass Sie den gedanklichen Faden verlieren, wenn Sie etwas erzählen oder wenn Sie etwas denken im Sinne von, dass Gedanken plötzlich aufhören, zum Beispiel? Wie als wenn Sie plötzlich weg- |
| 225 | **IH12:** Ja, das habe ich schon gehabt [STOẞT VERÄCHTLICH LUFT AUS]. Da habe ich noch im Kindergarten gearbeitet. |
| 226 | **I:** Okay. Das ist schon- schon lange her? Wie alt waren Sie da? |
| 227 | **IH12:** Äh ich habe nach der Realschule [ANONYMISIERT, BERUF] gelernt (I: Ja.). Mit 19 war ich im Anerkennungsjahr und bin krank geworden. Und dann habe ich das Jahr darauf das Anerkennungsjahr in einem anderen [ANONYMISIERT, AUSÜBUNGSSTÄTTE DES BERUFS] gemacht. |
| 228 | **I:** Mh-h (IH12: was auch gut war-) [crosstalk]. Nach der- nach der- |
| 229 | **IH12:** Erkrankung. |
| 230 | **I:** Erkrankung. Okay. |
| 231 | **IH12:** Und da habe ich das Glück gehabt, dass ich das tatsächlich in zwei Jahren gemacht habe [LACHT] (I: Ja.), halbtags [LACHT] (I: Ja.). Da habe ich dann im ersten Jahr die Kinder kennengelernt und äh Liedersammlungen für die Eltern gemacht (I: Ja.). Und die haben immer gesagt: So schade, die Kinder wollten daheim singen und sie wüssten nicht, wie das geht. Damals gab es noch kein Internet (I: Okay.). Das war ja 95 (I: Ja.), 93 (I: Ja.) [LACHT] (I: Okay.). Und dann habe ich für die Liedersammlungen gemacht. Und da habe ich den Kindern Geschichten erzählt (I: Mh-h (zustimmend)). Und die fanden das dann so toll. Und manchmal plötzlich mitten in der Geschichte habe ich plötzlich so total pff [MACHT KNALLENDES GERÄUSCH MIT DEN LIPPEN]. |
| 232 | **I:** War die Geschichte weg? |
| 233 | **IH12:** Ja. Aber es ging dann nur so drei Sekunden, da habe ich dann halt, was weiß ich, Kinder angeguckt, und dann war es wieder da. |
| 234 | **I:** Ach, die kamen dann immer wieder zurück? |
| 235 | **IH12:** Ja. |
| 236 | **I:** Also es waren immer so wie so kurze Blackouts? |
| 237 | **IH12:** Ja, so ganz kurz (I: Okay. Und dann waren-). Und dann war es wieder da (I: alles klar). Die Kinder haben es geliebt, wenn ich denen Geschichte erzählte [LACHT]. |
| 238 | **I:** Das kann ich mir vorstellen. Das ist auch superschön für Kinder. |
| 239 | **IH12:** Ich habe dann halt immer, was die- was die ähm erlebt haben, was weiß ich, im Winter, wenn Schnee war- ich hatte dann zwei Kinder, die da alles erlebt haben (I: Mh-h (zustimmend)). Und dann haben die da plötzlich Spuren im Schnee gesehen. Halt das, was die Kinder gerade erlebt haben. |
| 240 | **I:** Ja. Das wurde eingeflochten, sozusagen? |
| 241 | **IH12:** Ja [LACHT]. |
| 242 | **I:** Ja schön, schön. Ähm und die Gedanken waren dann wie plötzlich blockiert und dann wieder da? Oder sind sie auch verblasst und so- |
| 243 | **IH12:** Einfach weg. |
| 244 | **I:** Einfach sofort weg und dann kamen Sie- |
| 245 | **IH12:** Und nach drei Minuten- drei Sekunden ist es wieder da. |
| 246 | **I:** Okay. Okay. Und ähm das war nur da so? [crosstalk] oder kommt es- war das auch später nochmal? |
| 247 | **IH12:** Ja, ich gehe ja immer auf so Freizeiten. Und da müssen wir dann auch manchmal Geschichten erzählen und dann war es dann auch tatsächlich mal so, dass es plötzlich weg war. |
| 248 | **I:** Aber auch wieder kam? |
| 249 | **IH12:** Auch wieder kam (I: Mh (zustimmend)) und da auch [PAUSE]. Ja. |
| 250 | **I:** Das sind die beiden Beispiele, die Ihnen einfallen? |
| 251 | **IH12:** Mh. |
| 252 | **I:** Okay. Ja. Und ähm das hatten Sie vorher kurz erwähnt, wenn Sie lesen, dass Sie dann das Gefühl haben, Ihre Mutter liest vor (IH12: Mh-h (zustimmend)) für Sie? Ähm die Frage ist jetzt, ob es manchmal Ihnen so vorkommt, dass Gedanken wie nachgesprochen oder nachhallend im Kopf ähm- |
| 253 | **IH12:** Also es ist nicht laut. Ich weiß es auch nicht, wie ich es beschreiben soll. Es gibt ja Leute, wenn die lesen, dann sehen sie die Buchstaben. Und bei mir ist es so, dass ich tatsächlich das ähm so höre. Und nicht laut, sondern so wie ich denke halt. |
| 254 | **I:** Mh-h, okay. Und das ist aber- [crosstalk] |
| 255 | **IH12:** Wenn ich lese- wenn ich lese, dann geht es bei mir auch ganz langsam, weil ich das ja alles praktisch so in Hörbücher, wenn mir jemand alles vorliest. |
| 256 | **I:** Das ist zeitversetzt? Oder das ist- |
| 257 | **IH12:** Nee, es ist gleichzeitig. |
| 258 | **I:** Gleichzeitig. Okay. Und das ist immer assoziiert mit der Stimme Ihrer Mutter (IH12: ja), haben Sie gesagt? Okay. |
| 259 | **IH12:** Also das ist halt eine Stimme, und- und so hat es sich halt angehört, wenn meine Mutter mir früher vorgelesen hat. |
| 260 | **I:** Okay. Okay. Gut. Mh. Genau. Aber das ist nicht so, dass die Stimme irgendwo im Raum ist, sondern (IH12: Nee) die ist in Ihnen- |
| 261 | **IH12:** Die ist bei mir drin. |
| 262 | **I:** Und die ist auch nicht so laut, dass sie andere Leute hören können? |
| 263 | **IH12:** Nee. |
| 264 | **I:** Oder dass Sie zu laut für Sie selber ist? Also dass Sie es stört, im Sinne- |
| 265 | **IH12:** Nee. |
| 266 | **I:** Nee. Okay. Wollten Sie dazu gerade noch? |
| 267 | **IH12:** Wenn ich träume, dann ist es tatsächlich ziemlich laut, dass ich träume (I: Okay.) und auch ganz bunt. |
| 268 | **I:** Extrem, also mehr als die Realität sozusagen? |
| 269 | **IH12:** Sehr, sehr, sehr bunt und sehr, sehr, sehr laut. |
| 270 | **I:** Unangenehm laut? |
| 271 | **IH12:** Es geht. Also, es geht. |
| 272 | **I:** Mh-h. Und das heißt, wenn Sie aufwachen, können Sie sich an die Träume auch immer erinnern? |
| 273 | **IH12:** Meistens, ja. |
| 274 | **I:** Okay. Gab es aber schon mal den Moment, dass Sie nicht genau wussten, ob die jetzt ein Traum waren oder- |
| 275 | **IH12:** Oder ob sie Wirklichkeit waren, ja (I: Ja?). Das manchmal [LACHT] überlege ich tatsächlich, weil ich so lebhaft träume (I: Ja.). Ob das jetzt, dass Sie- aber ich kann es dann schon zuordnen, ob es jetzt Traum war, oder echt. |
| 276 | **I:** Aber Sie denken darüber nach, ob- (IH12: ja, ja, ja) ob es Realität war oder nicht? |
| 277 | **IH12:** Weil das jetzt so- heute Nacht habe ich zum Beispiel plötzlich geträumt, dass ich Blut abgenommen kriege. Und dann ist mir eingefallen: Ach Gott, ich muss ja mal wieder blutabnehmen gehen (I: Ja.). Und das hat irgendwie so super geklappt im Traum [LACHT]. Und da habe ich mich schon so gefreut, dass ich es hinter mir habe (I: Ja.) und dann: Oh, nee, das war ja nur ein Traum (I: Okay.). Egal, was ich erlebe, ich- ich träume sofort davon. |
| 278 | **I:** Okay, also es vermischt so ein bisschen manchmal? |
| 279 | **IH12:** Da bin ich gestürzt, der Zahn war draußen, in der Nacht habe ich sofort geträumt, dass der Zahn draußen ist (I: Alles klar, okay). Und da habe ich auch Glück gehabt, ich hab- dass ich in meinem Rucksack so ein Döschen habe. Da habe ich mal in der Zeitung gelesen, wenn man irgendwie hinfliegt, einen Zahn verliert, muss man das gleich in das Döschen tun und so damit man es wieder einstecken kann. |
| 280 | **I:** Und das war dann so? |
| 281 | **IH12:** Ja. |
| 282 | **I:** Sie konnten Ihren eigenen Zahn retten sozusagen? |
| 283 | **IH12:** Ja. Das erste, was ich gedacht habe: Wo ist mein Zahn? So groß war der. |
| 284 | **I:** Wahnsinn, ich habe kein Döschen in meinem Rucksack dabei [LACHT]. |
| 285 | **IH12:** Das war so ein viertel, halbes Jahr bevor ich geflogen bin, habe ich das in der Zeitung gelesen. Dann bin ich in die Apotheke und habe mir so ein Döschen- und dann hat meine Schwester gesagt: Also das ist ja selbsterfüllende Prophezeiung. |
| 286 | **I:** Wahnsinn, ja. |
| 287 | **IH12:** [LACHT] und die Zahnärztin hat mich total gelobt. Ich war echt eine Viertelstunde später bei mir auf dem- und die hat alles- alle abgesagt, dass sie mich sofort behandelt hat. Geröngt, ob da irgendetwas drin ist, ein Fremdkörper (I: Ja.) und hat es fotografiert, weil das war ein Megaunfall (I: Ja.) für die Berufsgenossenschaft (I: genau, für die Versicherung, ja). Und dann hat sie es mir wieder reingesteckt, ähm an den Nachbarzähnen festgemacht und die Lippe genäht. Und ach Gott, ach Gott. |
| 288 | **I:** Wahnsinn. Wann war das? |
| 289 | **IH12:** Das war im Oktober vor zwei Jahren. |
| 290 | **I:** Okay. Noch gar nicht so lange her. Und jetzt ist er wieder festgewachsen sozusagen? |
| 291 | **IH12:** Ja, die musste eine Wurzelbehandlung machen. Deswegen habe ich kein Gefühl (I: Hm (zustimmend)). Und sie hat gesagt, es kann sein, dass es sich irgendwann mal verfärbt. Bis jetzt noch nicht. |
| 292 | **I:** Nee, man sieht nichts. |
| 293 | **IH12:** Und ich hatte natürlich das große Glück, dass ich noch meine Zahnspange trage, so eine lose, und die hatte ich drin. Und ich glaube, die hat mir den Zahn gerettet. |
| 294 | **I:** Ah. Ah. Okay. Super. (IH12 und I: [LACHEN])). Glück gehabt. Wegen dem Döschen, super. Vielleicht sollte ich mir auch ein Döschen anschaffen. |
| 295 | **IH12:** Eine hat gesagt, man kann auch im Mund lassen. Aber dann hätte ich Angst gehabt, dass ich ihn verschlucke. Weil ich war total entstellt. |
| 296 | **I:** Ja. Ja. Oh man. |
| 297 | **IH12:** Und das war ein Schreck. War ein Schreck (I: Ja.). Habe noch Glück gehabt, mit meinem Knie bin ich drauf geflogen. |
| 298 | **I:** Wie sind Sie hingefallen? |
| 299 | **IH12:** Ich bin zum Bus gerannt (I: Okay.). Berg runter und plötzlich sehe ich mich stürzen. Da war es auch so, genau so, dass ich alles ganz hell gesehen habe, wie ich da hingeflogen bin im Zeittempo- im Zeitraffer. |
| 300 | **I:** In dem Moment? |
| 301 | **IH12:** Ja. |
| 302 | **I:** Okay. Okay. |
| 303 | **IH12:** Und da habe ich auch alles ganz hell gesehen. |
| 304 | **I:** Passiert das öfter, dass Sie die Sachen hell- hell, extrem hell oder extrem farbig sehen? Nicht nur- |
| 305 | **IH12:** Nicht nur in der Psychose, oder wenn ich unter Stress bin? |
| 306 | **I:** Ja, genau. |
| 307 | **IH12:** Eigentlich nicht. |
| 308 | **I:** Nee? Okay. |
| 309 | **IH12:** Ich habe auch zum Beispiel, da diese ganzen Krankenschwestern, die hatten ja diese weißen Kittel, die habe ich ganz hell gesehen (I: Hm (zustimmend)), wie Engel. Ich dachte so, die ganzen Krankenpfleger dort wären Engel. |
| 310 | **I:** In dem Stressmoment sozusagen? |
| 311 | **IH12:** Nee, wo ich halt in der Psychose war. |
| 312 | **I:** In der Psychose, okay. Jetzt nicht, als Sie, als Sie den Zahn- |
| 313 | **IH12:** Nee, da war ja keine Krankenschwester. |
| 314 | **I:** Achso. Nicht? Im- bei der- bei der Ärztin? |
| 315 | **IH12:** Achso, bei der Ärztin. Die haben alle so grüne Kittel. |
| 316 | **I:** Achso (IH12 und I: [LACHEN])), die sind nicht so schön. Okay. |
| 317 | **IH12:** Wobei auch eine tatsächlich einen weißen Kittel hatte, als ich das letzte Mal dort war. Und dann habe ich das so gesehen, und mir gedacht: Stimmt, damals habe ich gedacht Engel. |
| 318 | **I:** Mh. Aber da hat sich das dann nicht nochmal so angefühlt. |
| 319 | **IH12:** Nee, da habe ich nur gedacht: Hoffentlich kriege ich nicht wieder eine Psychose. |
| 320 | **I:** Mh. Mh. |
| 321 | **IH12:** Will ich eigentlich jetzt nicht mehr. |
| 322 | **I:** Ja. Verstehe ich. Klar. Ähm hatten Sie schon mal das Gefühl- über den Körper sprechen wir später nochmal detaillierter, aber dass Ihre Gedanken oder Ihre Wahrnehmungen irgendwo im Körper lokalisierbar sind? Oder im Kopf? Vorhin haben Sie gesagt: Gedanken sind über mir gewesen? Oder haben Sie schon mal die Gedanken durch den Körper- |
| 323 | **IH12:** Nee, das habe ich nicht gehabt. Also meine Gedanken waren immer im Kopf. |
| 324 | **I:** Immer im Kopf. Okay. Und da auch nicht an einer Seite oder irgendwo spezifisch lokalisierbar? |
| 325 | **IH12:** Mh (verneinend). |
| 326 | **I:** Nee. Okay. Alles klar. Gibt es manchmal die Situationen, dass Sie sich schwer entscheiden können? (IH12: [LACHT]) Ja? Sie lachen? |
| 327 | **IH12:** [LACHT]. Oh ich kann mich überhaupt nicht entscheiden. |
| 328 | **I:** In was für Situationen? |
| 329 | **IH12:** Immer. |
| 330 | **I:** Immer, egal. Sagen Sie mir ein Beispiel? |
| 331 | **IH12:** Zum Beispiel werde ich jetzt diese Woche und nächste Woche total den Stress, weil ich nächsten Freitag- Freitag in einer Woche- ich bin im Kirchenchor (I: Mh-h (zustimmend)) und Freitag in einer Woche haben wir ein Konzert. Und das ist so viel, und ich kann es überhaupt nicht. Das ist viel zu schwer für mich. Ich kann ja kein Latein. Und das ist- das ist mit dem katholischen Kirchenchor zusammen, so eine Messe. Ich war solange dabei, um es zu üben, aber ich hätte jetzt heute Abend da wieder um acht eine Probe. Gestern hatte ich eine Probe. Dann Wochenende Probe. Montag, Dienstag, Probe, Mittwoch von sieben bis zehn Probe und am Freitag nochmal eine Probe, und dann die Aufführung. Und ich wollte das alles mitmachen. Und meine Mutter hat- die hat zu mir gesagt: [ANONYMISIERT, NAME DER INTERVIEWTEN PERSON], willst du wieder krank werden? (I: Hm (zustimmend)) Und ich reg mich ja so auf, wenn ich da vorsinge (I: Ja.). Da denke ich ja die ganze Zeit, ich falle jetzt gleich in Ohnmacht. |
| 332 | **I:** Vor Aufregung? |
| 333 | **IH12:** Ja (I: ja okay). Und [ANONYMISIERT, NAME EINES ARZTES] hat mir gesagt, dass man eigentlich nicht in Ohnmacht fällt, wenn man sein Leben noch nie in Ohnmacht gefallen ist [LACHT]. |
| 334 | **I:** [LACHT] Ich glaube, das weiß er besser als ich. Ja. |
| 335 | **IH12:** Und ich bin gestern zur Probe gerannt, und ich wäre heute Abend auch zur Probe gerannt. Und jetzt habe ich mich einfach entschieden, da nicht mitzumachen. |
| 336 | **I:** Und diese Entscheidung ist Ihnen sehr schwer gefallen? Mh-h. |
| 337 | **IH12:** Oder auch die Entscheidung, dass- ob ich jetzt einen Tag früher vom Urlaub zurückkomme oder noch ein Jahr- Tag länger bleibe. Oder- oder ähm die Entscheidung, was ich morgens jetzt eigentlich anziehen soll oder (I: Mh-h (zustimmend)) bei der Arbeit. Oder eigentlich ständig. Aber mein- meine Mutter führt das auch etwas darauf, dass ich Jungfrau bin. |
| 338 | **I:** Dass Sie was? |
| 339 | **IH12:** Ich bin Ascendent Jungfrau. |
| 340 | **I:** Achso, vom Steinzei- Sternzeichen her. |
| 341 | **IH12:** Und mein Onkel, also mein Lieblingsonkel. Der war auch Jungfrau, und der hatte s- konnte sich auch nie entscheiden. |
| 342 | **I:** Okay. Okay. Äh okay, das ist die Erklärung sozusagen (IH12: [LACHT]). Aber, das heißt äh für- für mich ist wichtig in- in einfachen, also auch morgens, wenn Sie- wenn Sie nicht genau wissen, was Sie anziehen sollen, dass- in solchen Situationen kommt das auch vor? |
| 343 | **IH12:** So ganz einfache Situationen? |
| 344 | **I:** Ja. Weil so eine Entscheidung mit dem Chor, das ist ja schon schwierig, ne? Also, da- das finde ich- |
| 345 | **IH12:** Ob man da mitsingt oder nicht? |
| 346 | **I:** Genau, ob man mitsingt oder nicht, das ist äh- |
| 347 | **IH12:** Und immer wieder soll ich, oder soll ich nicht? |
| 348 | **I:** Genau. Während so- so was ziehe ich morgens an? Das hat nicht so viele Konsequenzen, aber das fällt Ihnen auch schwer? |
| 349 | **IH12:** Ja. |
| 350 | **I:** Und auch im Supermarkt? Ähm- |
| 351 | **IH12:** Was ich kaufen soll? Allerdings auch [LACHT]. |
| 352 | **I:** Solche Sachen. Und da- da sind es die Optionen? Die- |
| 353 | **IH12:** Es ist einfach zu viel. Wenn da jetzt eine Sache wäre, dann würde ich die kaufen (I: Mh-h (zustimmend), okay, verstehe.). Ich gucke halt dann immer nach dem Billigsten (I: Okay, ja) [LACHT]. Manchmal gönne ich mir aber auch etwas. |
| 354 | **I:** Na klar (IH12: [LACHT]). Ja, na klar. Und ähm wie ist es so? Ist es dann ähm Sie wissen dann selber nicht mehr genau, wonach ich- wonach Sie entscheiden sollen, oder? |
| 355 | **IH12:** Ja, oder ob die Entscheidung- jetzt habe ich mir eine Fahrkarte gekauft: Soll ich jetzt einen Fensterplatz oder ein einen Gangplatz am Gang nehmen? (I: Mh-h (zustimmend)) Was ja eigentlich jetzt keine große Entscheidung ist (I: Ja.), und ich mach das schon, dass ich so [LACHT] sehr lange darüber nachdenken muss: Nehme ich jetzt einen Fensterplatz oder einen Gangplatz? |
| 356 | **I:** Und was machen Sie dann? |
| 357 | **IH12:** Ich habe jetzt einfach einen Fensterplatz genommen. |
| 358 | **I:** Okay, Sie entscheiden sich dann? |
| 359 | **IH12:** Irgendwann habe ich mich dann- ich- ich war ja da bei der- bei- beim Bahnhof gestanden, und da hat er da so gesagt: Ja, was sollen wir jetzt machen? Und dann habe ich gesagt: Gangplatz. Und da hat er gesagt: Okay, Gangplatz? Und dann sage ich: Nee, doch lieber Fensterplatz! Und da sagt er: Okay, soll ich Fensterplatz einloggen? Und da sage ich: Ja, machen Sie das! [LACHT] |
| 360 | **I:** Ach Sie haben die Fahrkarte bei- beim Schalter gekauft? |
| 361 | **IH12:** Ich- ich hab- ich war vorher- daheim habe ich im Internet geguckt und habe mir die Verbindungen herausgeschrieben (I: Mh-h (zustimmend)), wo ich schon On- Angebot gesehen habe und dann bin ich damit zur Bank. Äh zu- zum Bahnhof. |
| 362 | **I:** Zum Schalter. Und, also "Er" ist die Person, die Ihnen das Ticket verkauft hat. Okay, gut. |
| 363 | **IH12:** Und dann hat er mich gefragt: Welchen Zug wollen Sie jetzt nehmen? Morgens, den um halb neun, oder um zwölf? Um zwölf hätte ich dann weniger umsteigen müssen. Und ich: Was mache ich denn jetzt? 9? 12? Was soll ich so lange noch in [ANONYMISIERT, ORT IN DEUTSCHLAND] machen? Weil ich muss ja aus der Pension raus (I: Mh-h (zustimmend)). Andererseits bin ich da schon um fünf daheim, beim anderen erst um acht daheim. Was mache ich denn jetzt? Lieber einmal weniger umsteigen oder doch das andere machen? Weil, ist auch so, das Umsteigen ist für mich totaler Stress (I: Mh-h, mh-h (zustimmend)), weil ich zehn Mal gucke, ob ich alles habe [LACHT] (I: Ja.). Ich tue dann schon alles zum Ausgang, und dann gehe ich nochmal zurück, dann gehe ich nochmal zurück, und dann ist da eine Frau, die da sitzt, und dann frage ich sie: Da liegt ja jetzt nichts von mir? Und die guckt da: Nee, es ist nichts, Sie können gehen! (I: Okay.) Also es ist für mich echt Stress (I: Okay.), so ein Umstieg. |
| 364 | **I:** Also das ist- da machen Sie sich auch über Ihr eigenes Verhalten und über die eigenen- die eigene Fähigkeit, sozusagen, Gedanken? |
| 365 | **IH12:** Ja, ich habe halt Angst, dass ich etwas vergesse. |
| 366 | **I:** Ja. Und das ist dann auch immer wieder? Also es ist häufig so, dass Sie Angst haben, etwas vergessen zu haben? |
| 367 | **IH12:** Oh ja, also ich brauche bei der Arbeit schon lange, bis ich gehe. (I: Okay, und dann-) Ich habe dann tatsächlich mir mal eine Liste gemacht und habe dann immer abgehakt. Aber das mache ich jetzt [/nicht mehr/] - also da war noch die [ANONYMISIERT, NAME EINER ÄRZTIN] da, meine Ärztin, die kennen Sie vielleicht gar nicht mehr. |
| 368 | **I:** Die kenne ich nicht, nee. |
| 369 | **IH12:** Die ist auch schon lange weg. Und dann habe ich das der erzählt, dass ich mir eine Liste gemacht habe, wo ich das abhake. Und dann hat sie gesagt, okay gut, aber über kurz oder lange muss diese Liste weg (I: Mh-h (zustimmend)). Die hat gesagt: Brauchen Sie nicht von heute auf morgen, aber reduzieren Sie es mal ein bisschen. (I: Ja.) Und dann habe ich es dann auch nur gemacht, wenn ich wirklich total im Stress war (I: Ja.). Oder hatte ich auch wirklich noch mehr Sachen, dann musste ich irgendwann natürlich den Computer ausschalten, dann das [/den/] Strom wegmachen, das Fenster, irgendwas. Da hatte ich so sechs, sieben Sachen, die ich machen musste, und jetzt sind es nur noch zwei (I: Okay.). Also ich muss nur noch den Strom ausmachen und Schrank zusperren (I: Okay.). Und gucken, dass Fenster zu ist, ob das Licht aus ist, und- und der Kühlschrank zu ist. Und die Klimaanlage aus ist [LACHT]. |
| 370 | **I:** Also doch noch einige Sachen, ne? Und das machen Sie- das machen Sie immer genau die gleiche Reihenfolge jeden Abend sozusagen, nach der- nach der Arbeit? |
| 371 | **IH12:** Ja ich- [RÄUSPERT SICH] ich gucke halt dann immer noch mal (I: Mh-h (zustimmend)). Und dann bin ich schon unten, dann gehe ich halt doch noch einmal nach oben und gucke (I: Mh-h (zustimmend)), ob auch die Zimmertür zu ist. Und dann gehe ich wieder runter [RÄUSPERT SICH] und dann habe ich ja auch meinen Geldbeutel und mein Handy und so. Dass alles drin ist, und so (I: Mh-h (zustimmend)) und dann laufe ich, und wenn ich dann fast beim Bus bin, muss ich nochmal gucken, ob auch alles drin ist [LACHT] (I: Mh-h (zustimmend), okay, verstehe). Also ich habe da schon etwas Kontrollzwänge. |
| 372 | **I:** Ja, ja. Ich würde das jetzt, also, ich gebe dem jetzt einfach keine Bezeichnung, erstmal. Aber es geht ja darum, ob es Sie belastet, ne? |
| 373 | **IH12:** Ja, ja, also wenn ich im Stress bin, dann belastet es mich schon, aber wenn ich in aller Ruhe das mache, dann geht es (I: Okay.). Da habe ich mir jetzt auch überlegt, ob bei mir irgendwie die Tabletten? Aber ich habe dann gedacht: Nee, jetzt bin ich schon so lange stabil. |
| 374 | **I:** Mh. Und der wollte Sie vielleicht reduzieren? Oder, oder? |
| 375 | **IH12:** Er hat überlegt, wenn- er hat auch gemeint, das wäre vielleicht auch eine Nebenwirkung von dem [ANONYMISIERT, NAME EINES MEDIKAMENTES] (I: Okay.) [RÄUSPERT SICH]. Weiß es ja nicht. |
| 376 | **I:** Ja, das kann- kann man nicht so genau wissen. |
| 377 | **IH12:** Wobei ich das eigentlich schon immer hatte. Ich habe das auch von meinem Vater ein bisschen übernommen. |
| 378 | **I:** Also Sie hatten das auch schon, bevor Sie [ANONYMISIERT, NAME EINES MEDIKAMENTES] genommen haben? |
| 379 | **IH12:** Ich weiß es nicht, weil ich ja [ANONYMISIERT, NAME EINES MEDIKAMENTES] schon seitdem ich 19 bin- (I: Mh(zustimmend)) ich weiß ja nicht, wie ich in Wirklichkeit bin. 19. |
| 380 | **I:** Was- Sie meinen, Sie sind- Sie sind nicht in- es ist nicht die Wirklichkeit, sozusagen? Mit dem [ANONYMISIERT, NAME EINES MEDIKAMENTES]? |
| 381 | **IH12:** Ich weiß es ja nicht. Also ob ich das mit 16 auch hatte, diese Kontrollzwänge (I: Okay.). Also bei meinem Vater war es zum Beispiel so, wir sind öfter ins Schwimmbad gefahren und jedes Mal, wenn wir fast beim Schwimmbad waren, hat er plötzlich gesagt: Habe ich jetzt das Auto abgesperrt? Und er ist jedes Mal wieder zurückgelaufen und hat geguckt [LACHT] (I: Ja.) und es war immer abgesperrt (I: Okay.). Vielleicht habe ich das auch von ihm ein bisschen übernommen. (I: Hm (zustimmend), ja, das kann sein). Wobei mein Vater ja nun auch keine Tabletten nimmt. |
| 382 | **I:** Der lebt noch, der Vater, ne? |
| 383 | **IH12:** Ja (I: Ja.), der nimmt ja keine- keine Psychopharmaka. |
| 384 | **I:** Ja. Und da ist es- ist es dennoch so. Mh-h. Okay. Verstehe. Das habe ich Sie schon gefragt ähm im Ansatz, gab oder gibt es Situationen, in denen Sie nicht so recht wissen, ob Erinnerung, Gedanken oder Wirklichkeit oder Traum oder Wirklichkeit (IH12: Mh-h), das hatten wir schon besprochen (IH12: Mh-h), ne? Aber Sie haben gesagt, dass Sie immer dann am Ende doch zuordnen können- |
| 385 | **IH12:** Ich kann dann schon zuordnen, was ein Traum war oder ob es kein Traum war (I: genau). Das kann ich dann schon zuordnen. |
| 386 | **I:** Genau. Ähm wie sieht es aus? Sind wir noch fit, oder? Ja? |
| 387 | **IH12:** Ich würde aber jetzt tatsächlich mal auf die Toilette gehen? |
| 388 | **I:** Okay, dann machen wir das kurz. |
| 389 | **IH12:** Okay. Es ist ja gerade bei- |
| 390 | **I:** Ja, hier direkt gegenüber [GERÄUSCHE IM RAUM]. Audio einmal Pause. [AUDIO WIRD UNTERBROCHEN UND WIEDER FORTGESETZT] So. Ja, sind alle für Sie, bitte gerne (IH12: [LACHT]). Ähm haben Sie manchmal Schwierigkeiten, Ihr Denken oder Handeln in Gang zu bringen? Also das Gefühl, ich will etwas tun, aber ich komme nicht so richtig in die Gänge? (IH12: Ja) Ich kann nicht so richtig [TELLER KLAPPERT] es umsetzen, was ich denke? |
| 391 | **IH12:** Mh. Mh. Also wenn ich morgens aufwache, habe ich eigentlich überhaupt keine Lust aufzustehen. Wenn das so- oder, weiß es nicht. Da habe ich schon ziemliche Probleme aufzuwachen. |
| 392 | **I:** Okay. Was ist schwierig morgens? Könnte sein- morgens könnte sein, wegen den Medikamenten auch. Oder- oder- also Sie nehmen sich dann vor aufzustehen, aber es geht nicht so richtig? Ja? Das ist jeden Morgen so? Und äh bezieht sich das- das Ingangbringen nur aufs Aufstehen oder auch auf andere Aspekte? |
| 393 | **IH12:** Dann freue ich mich zum Beispiel jedes Mal, dass Wochenende ist, und dann komme ich nicht aus dem Bett raus! (I: Mh-h (zustimmend)) Stell dann meinen Wecker wieder aus. Wenn ich es dann mal geschafft habe, dann fällt mir dann ab und zu auch schon mal etwas ein, was ich machen könnte. |
| 394 | **I:** Ja. Okay. Und dann stellen Sie sich etwas vor, und das machen Sie dann auch? |
| 395 | **IH12:** Ja. |
| 396 | **I:** Ja. Okay. Und wie ist es mit der Aufmerksamkeit? Gibt es Situationen, in denen Sie Details in der Umgebung besonders fesseln? Und Sie Ihre Aufmerksamkeit gar nicht mehr davon wegreißen können? Nicht, nee? Oder haben Sie manchmal Schwierigkeiten, sich auf etwas zu konzentrieren, wenn gleichzeitig andere Reize da sind? |
| 397 | **IH12:** Ja (I: zum Bei-), kann ich gar nicht. |
| 398 | **I:** Ja? Also zum Beispiel, wenn viele Leute gleichzeitig reden? Oder ein Radio- |
| 399 | **IH12:** Kann ich nicht. |
| 400 | **I:** Nee? Können Sie mir da ein Beipiel nennen? |
| 401 | **IH12:** Mh. (Glas wird auf Tisch abgestellt). Das fängt schon an, wenn ich im Bus bin und da sitzen zwei hinter mir, die sich unterhalten (I: Mh-h (zustimmend)). Im Bus mache ich ja meistens irgendwas. Und dann setze ich mich dann meistens weg [LACHT]. |
| 402 | **I:** Ja? Okay. Okay. So sehr stört Sie das? Ähm und- |
| 403 | **IH12:** Manchmal kann ich es auch ausblenden. (I: Mh-h (zustimmend)) Aber zum Beispiel vor einem Jahr ist mein Juniorchef gekommen. Ich hatte mal mit meiner Seniorchefin das Büro zusammen (I: Mh-h (zustimmend)), die wollte das irgendwie so haben. Also im Vorraum von ihrem Mann (I: Mh-h (zustimmend)), das sind zwei Büros, die durch eine Tür verbunden sind (I: Okay.). In den einem ist der Seniorchef und im anderen war ich mit ihr. Da kam [ANONYMISIERT, NAME DES VORGESETZTEN DER INTERVIEWTEN PERSON], und da hat er gesagt: Frau [ANONYMISIERT, NAME DER INTERVIEWTEN PERSON], er braucht das Büro, ich müsste jetzt ins Großraumbüro. Und da habe ich zum Glück gleich geschaltet und habe ihm gesagt: Das kann ich nicht. Wenn ich da nur reingehe zum Kopieren, und da sind immer zwei, die telefonieren (I: Mh (zustimmend)) und nochmal zwei, die irgendwas besprechen, irgendeine Arbeit besprechen (I: Mh (zustimmend)). Es ist ein wahnsinniger Geräuschpegel (I: Mh (zustimmend)). Und dann wird- und dann habe ich zu ihm gesagt: Ich kann mich da- ich kann das nicht, mich dann auf die Arbeit konzentrieren. Und das ist auch wirklich nicht gelogen (I: Ja.). Und dann hat er gesagt, er überlegt sich etwas für mich. Und jetzt habe ich das Glück gehabt, dass ich da mit der Kollegin aus der Buchhaltung zusammen oben sitze (I: Okay.), wo es auch so heiß war. |
| 404 | **I:** Okay, ja, das haben Sie erzählt. Und da ist es dann ruhig? |
| 405 | **IH12:** Da ist es ruhig. Die Kollegin ist total ruhig. Die pff also die ist total nett, da habe ich Glück gehabt (I: Okay.). Die bastelt auch so gerne und dann zeigt sie mir, was sie gemalt hat [LACHT]. |
| 406 | **I:** [LACHT]. Schön. Das heißt, wenn viele ähm vor allem, was Geräusche betrifft (IH12: Geräusche, ja), viele Geräusche gleichzeitig sind, dann können Sie sich auf das, was Sie machen, nicht konzentrieren? |
| 407 | **IH12:** Ja, oder heute in der S-Bahn saß ich auf dem Platz und plötzlich sehe ich, dass da oben so ein Licht flackert (I: Mh-h (zustimmend)). Da habe ich mich auch weggesetzt (I: Okay.), weil es mich so gestört hat (I: Okay.). Ich habe dann die neben mich gefragt: Stört Sie das nicht? [/Und sie:/] Habe das noch gar nicht gemerkt [LACHT]. |
| 408 | **I:** Alles klar, okay. Verstehe. Gut. Und Gedächtnis hatten wir vorhin kurz angesprochen. Sie haben gesagt, wenn Sie- Sie lesen langsam, weil es schwierig ist, sich ähm zu erinnern an den Anfang, den Sie gerade gelesen habe, oder? |
| 409 | **IH12:** Nee, weil ich einfach langsam lese. Weil ich Buchstabe für Buchstabe lese. |
| 410 | **I:** Okay. Aber Sie können sich äh Sie können- also das Kurzzeitgedächtnis funktioniert gut? Sie können sich erinnern? |
| 411 | **IH12:** Also, wenn ich etwas erlebt habe, erinnere ich mich sehr gut daran (I: Mh-h (zustimmend)). An Zahlen erinnere ich mich überhaupt nicht (I: Mh-h (zustimmend)). Ich wüsste jetzt nicht mehr, wie viel Geld ich heute gezählt [LACHT] habe (I: Okay, okay). Und dann habe ich auch echt Lernprobleme. Zum Beispiel Vokabeln, kann ich gar nicht. |
| 412 | **I:** Können Sie sich nicht merken? |
| 413 | **IH12:** Kann ich mir nicht merken (I: Mh-h (zustimmend) ähm-). Das war in der Schule halt auch das Problem. |
| 414 | **I:** Mh-h. Das ist ähm eine allgemeine Sache, die ist (Gegenstand auf Teller klappert) auch diagnostiziert worden, dass Sie Lernschwierigkeiten haben? Oder das war einfach so- ist Ihre eigene Wahrnehmung? |
| 415 | **IH12:** Das ist meine- das war so. |
| 416 | **I:** Ja, also für Sie selber, sage ich. |
| 417 | **IH12:** Das fing ja schon im Kindergarten an. Da konnte ich mir die Farben nicht merken [LACHT] (I: Okay, okay). Da war ich in der zweiten Gruppe, also war ich vier (I: Mh-h (zustimmend)). Und da sind wir immer auf dem Weg an Autos vorbeigelaufen, meine Mutter bei jedem Auto: [ANONYMISIERT, NAME DER INTERVIEWTEN PERSON], was ist das für eine Farbe? [LACHT] Ich wusste es nicht (I: Ja.). Ich seh- ich habe die zwar gesehen, die Farben, aber wie die jetzt heißen? Gut, ich habe es dann irgendwann doch noch gelernt [LACHT] (I: Ja.). Oder auch Buchstaben merken. (I: Hm (zustimmend)) Meine Mutter hat dann mir die Buchststaben aufgeschrieben und an die Pinnwand gehängt, dass ich das immer sehe und so. Ich meine, ich habe es dann irgendwann doch gelernt. |
| 418 | **I:** Aber es war schwierig für Sie? |
| 419 | **IH12:** Meine Mutter hat dann viel mit mir gelernt. Und ich hatte auch das Glück, dass meine Mutter tatsächlich Englischlehrerin für Realschule ist (I: Mh-h (zustimmend)). Und die hatte da auch ihre Arbeit. Anfangsunterricht in der fünften Klasse in Englisch und da hat sie das natürlich toll gewusst, ne? Da mit mir zusammen gelernt. |
| 420 | **I:** Da war sie pädagogisch sozusagen geschult? |
| 421 | **IH12:** Die hat dann mir eine Box hingestellt für Box [LACHT] oder einen Vogel für bird [LACHT] (I: Okay, schön). Und haben gelernt und gelernt und dann kam die erste- das erste Diktat. Und ich hatte eine Fünf (I: Ah). Und ich war so verzweifelt. Ich habe gedacht: Wie soll denn das jetzt werden? Jetzt bin ich mal gerade in der fünften Klasse (I: Mh (zustimmend)), bis zur zehnten Klasse? |
| 422 | **I:** Mh. Aber es hatte irgendwie geklappt? |
| 423 | **IH12:** Ja, in der achten, neuten, plötzlich. Ich habe- ich war natürlich auch fleißig. Meine Mutter hat dann Vokabelkärtchen gemacht, und die habe ich jeden Tag wiederholt und wiederholt. Und irgendwann habe ich es dann doch noch gelernt (I: Mh (zustimmend)). Mathematik habe ich eigentlich keine Probleme gehabt. |
| 424 | **I:** Okay, super. Das heißt ähm aber jetzt, wenn Sie einen Film schauen, oder so- |
| 425 | **IH12:** Das weiß ich, was drin vorkommt. |
| 426 | **I:** Dann- dann können Sie sich gut erinnern, was am Anfang war und (IH12: ja) das ist alles kein Problem? |
| 427 | **IH12:** Das ist kein Problem. Es ist wirklich nur das Lernen (I: Hm (zustimmend)). Und das habe ich auch in der [ANONYMISIERT, ANGABE DER BERUFSAUSBILDUNG]- ach Gott, ach Gott, ach Gott. Das war schon schlimm. |
| 428 | **I:** Das war schwierig, ja? |
| 429 | **IH12:** Ja [LACHT]. |
| 430 | **I:** Das kann ich mir vorstellen. Wie ist es, wenn Sie Ihr eigenes Zeiterleben angucken? Kommt es manchmal da zu Veränderungen? Dass die Zeit schneller oder langsamer läuft? |
| 431 | **IH12:** Also mir kommt- es ist Wahnsinn, dass es Wahnsinn, dass es Mai ist, mir kommt es vor, als hätte das Jahr gerade angefangen. |
| 432 | **I:** Okay. Das heißt, Sie (IH12: geht schnell) haben so viel zu tun gehabt, oder? |
| 433 | **IH12:** Die Zeit geht so schnell herum. |
| 434 | **I:** Okay. Das ist jetzt auf die- die großere Zeit, sozusagen, auf Monate und Jahre und so bezogen. Und jetzt im Moment? Kann es sein, dass es wie plötzlich im Zeitraffer stattfindet? Oder verlangsamt? |
| 435 | **IH12:** Das habe ich schon erzählt, wo ich da gestürzt bin, das habe ich im Zeitraffer erlebt. |
| 436 | **I:** Okay. Sch- langsamer? |
| 437 | **IH12:** Langsamer. |
| 438 | **I:** Langsamer, okay. |
| 439 | **IH12:** Langsam (I: Ja.). Oder wenn ich irgendwo total plötzlich ein Auto auf mich zukommen sehe: Ganz langsam (I: Mh-h (zustimmend)), ganz langsam. |
| 440 | **I:** Das sind Stresssituationen, oder? |
| 441 | **IH12:** Wahrscheinlich. |
| 442 | **I:** Und das mit dem Auto, das auf Sie zugekommen ist, das war- da waren Sie auf der Straße, oder? |
| 443 | **IH12:** Wahrscheinlich, ja. |
| 444 | **I:** Ja? Da können Sie sich nicht mehr genau erinnern an die Situation? |
| 445 | **IH12:** Doch, ich weiß, dass- dass- dass ein Auto kommt auf mich zu und total langsam. Ah ja, da bin ich ja hingeflogen. Da bin ich ja bei der [ANONYMISIERT, ORT DES INTERVIEWS] hingeflogen. |
| 446 | **I:** Mh-h und das war auf der Straße? |
| 447 | **IH12:** Da musste ich noch mit meinem Seniorchef über die Straße rübergehen zum Bäcker (I: Mh-h (zustimmend)). Und ich liege da und sehe dieses Auto dann auf mich ganz langsam auf mich zukommen. Aber es ist ja nichts passiert. Der hat mich ja auch gesehen (I: Ja.). Aber es hätte schon passieren können. |
| 448 | **I:** Ja, okay. Das heißt, das sind immer gefährliche Situationen gewesen. Okay, verstehe, mh. |
| 449 | **IH12:** Oder wenn plötzlich- habe ich plötzlich mal, dass ich einen Schreck kriege, oder so (I: Mh-h (zustimmend)), wenn ich bei irgendetwas erwischt werde. Dann ist es auch ganz langsam. |
| 450 | **I:** Was heißt erwischt werde? |
| 451 | **IH12:** [LACHT]. Ich muss jetzt gerade mal überlegen. Mh. Fällt mir jetzt auch gerade nichts ein. Vielleicht mit einem Spickzettel mal [LACHT]. |
| 452 | **I:** Okay. Okay. Okay. Früher schön? |
| 453 | **IH12:** Früher. |
| 454 | **I:** Ja alles klar. Und- |
| 455 | **IH12:** Und dann gibt es aber auch Situationen, wo ich mein ganzes Leben sehe. Innerhalb von einer Sekunde. |
| 456 | **I:** Okay? Was sind das für Situationen? |
| 457 | **IH12:** Das war zum Beispiel, als ich ins Wasser gesprungen bin mit einem Köpfer. Wo mein Kopf aufs Wasser aufkam, habe ich in- innerhalb einer Sekunde mein Leben ganzes gesehen. |
| 458 | **I:** Das war- wo war das und wann? |
| 459 | **IH12:** Im- im Schwimmbad. |
| 460 | **I:** Das war keine gefährliche Situation? |
| 461 | **IH12:** Nee, es war nichts. Ich bin da nur mit einem Köpfer reingesprungen. |
| 462 | **I:** Und dann hatten Sie plötzlich so einen Flash (IH12: Ja) über Ihr ganzes Leben? Mh-h. Und war das bedrohlich? |
| 463 | **IH12:** Nö. |
| 464 | **I:** Nö? Gut, schön? Eine schöne Erfahrung, oder? |
| 465 | **IH12:** Vielleicht war es auch jetzt nicht das ganze Leben, vielleicht war es auch nur eine Woche, die ich gesehen habe, aber auf jeden Fall- ja, mein Onkel hat das auch erzählt, der hat- der ist tatsächlich mal von einem Blitz getroffen worden. |
| 466 | **I:** Wirklich? Wow. Okay. |
| 467 | **IH12:** Und da hat er erzählt, er hätte ganz hell gesehen. Und da hatte er sein ganzes Leben gesehen. Und dann war er weg gewesen. Und dann hat er das Glück gehabt, das war auf einem Feld in- in [ANONYMISIERT, ORT DES INTERVIEWS] (I: Mh-h (zustimmend)), dass der Blitz nochmal woanders eingeschlagen hat und dadurch ist er wieder zu sich gekommen. Und da war alles so taub gewesen (I: Mh-h (zustimmend)) und dann ist er in die Klinik gegangen und hat hier einen richtigen Blitz gehabt. |
| 468 | **I:** Wirklich? |
| 469 | **IH12:** Und dann hat- und dann hat der Arzt die Studenten [/gefragt/]: Was glauben Sie, was dieser Mann hat? [LACHT] |
| 470 | **I:** Da war er ein Versuchsobjekt, sozusagen, oder- oder Vorzeigeobjekt. Subjekt. So, ja. Wow. |
| 471 | **IH12:** [LACHT]. Und da hat er- und man sagt ja auch, wenn jemand stirbt, dass man dann nochmal sein ganzes Leben sieht. Also habe ich mal gelesen. |
| 472 | **I:** Ja, ja, ja. Das hört man manchmal, ne? Ja. |
| 473 | **IH12:** Deswegen- und das ist ja auch nicht schlimm (I: Okay.). Auch nicht schlimm. |
| 474 | **I:** Ich weiß nicht, ob das schlimm ist dann. Ich habe es selber noch nie erlebt (IH12: [LACHT]). Ich glaube, es kommt auch auf das Leben drauf an, oder? Das man dann sieht, vielleicht? |
| 475 | **IH12:** Ja? (I: Ob einen-) Achso. Ob es gut war? |
| 476 | **I:** Mh, genau. Ob es einem- ob man da- damit okay ist, oder so. |
| 477 | **IH12:** Mh, das weiß ich jetzt auch nicht. |
| 478 | **I:** Ja. Ähm. Okay, und Sie haben aber jetzt nicht manchmal das Gefühl, dass es nur noch das Hier und Jetzt gibt, und keine Vergangenheit und keine (IH12: Mh (verneinend)) Zukunft? Okay. Gibt es Situationen, in denen es kleine Lücken oder Brüche oder Aussetzer gibt im Bewusstsein? Dass Sie plötzlich nicht mehr wissen, wie Sie irgendwo hingekommen sind, zum Beispiel? |
| 479 | **IH12:** Nee, das wüsste- weiß ich eigentlich schon immer. |
| 480 | **I:** Okay. Gut. Und ähm wenn Sie darüber nachdenken, was Sie so sagen und ausdrücken, haben Sie immer das Gefühl, das passt zu dem, was Sie eigentlich sagen wollen und ausdrücken wollen? Oder ähm kommt es manchmal falsch oder anders heraus, so wie Sie es gar nicht sagen wollten? |
| 481 | **IH12:** Es passieren immer Missverständnisse. |
| 482 | **I:** Aber das liegt nicht daran, dass Sie sich jetzt- |
| 483 | **IH12:** Wahrscheinlich habe ich mich falsch ausgedrückt, ich weiß es nicht. (Glas wird auf Tisch abgestellt) Ähm wegen dem Tennis, da hatte ich ja letztens ja- ich war ja in der Mannschaft (I: Mh-h (zustimmend)) und hatte Mannschaftstraining (I: Mh-h (zustimmend)) [LACHT]. Und das Mannschaftsspielen liegt mir ja überhaupt nicht [LACHT]. |
| 484 | **I:** Okay, okay. Sie schwingen lieber alleine? Also- im- also eins zu eins, sozusagen? Oder? |
| 485 | **IH12:** Ich spiele lieber miteinander, als gegeneinander (I: okay, verstehe). Weil dann fährt man dann irgendwohin, es ist zwar immer ganz nett, da gibt es gutes Essen und so (I: Hm (zustimmend)), aber ich rege mich da so auf und kann überhaupt nicht mehr Tennis spielen [LACHT]. |
| 486 | **I:** [LACHT] Alles klar. |
| 487 | **IH12:** Und dann war das halt mit dem Training, dass ich dann da nicht mehr mitmachen wollte, und so. Und dann habe ich das beim- im Frühjahr erzählt, und dann haben die das so aufgefasst, dass ich da nicht mehr mitmachen darf. Aber das stimmt ja gar nicht. Ich habe wegen mir nicht mehr mitmachen wollen (I: Okay, okay.). Und dann habe ich das wahrscheinlich falsch rübergebracht. |
| 488 | **I:** Okay. Gut, so Missverständnisse passieren oft, ne? Dass man etwas sagen will. Aber haben Sie selber das Gefühl, dass Sie es nicht richtig ausgedrückt haben? |
| 489 | **IH12:** Nö. |
| 490 | **I:** Nee, okay. Darum geht es, um Ihr eigenes (IH12: achso) Gefühl (IH12: Ja). Ähm und das heißt, Sie- Sie haben auch das Gefühl, Sie können meistens die Worte für das finden, was Sie- was Sie sagen wollen und da gehen nicht plötzlich die Worte verloren? Oder so. |
| 491 | **IH12:** Nee. |
| 492 | **I:** Okay. Gut, dann kommen wir zu einem Bereich, der mehr so ähm Ihr grundlegendes Gefühl mit sich selber betrifft. Eigene Wahrnehmungen, selber da sein, präsent sein (IH12: Mh-h (zustimmend)). Gibt es da Besonderheiten? Veränderungen bei Ihnen? |
| 493 | **IH12:** Jetzt von der Krankheit und jetzt? |
| 494 | **I:** Zum Beispiel, oder auch vor der Krankheit schon? |
| 495 | **IH12:** Habe es jetzt gar nicht verstanden, also. |
| 496 | **I:** Ähm ich kann auch spezifischer fragen. Ich wollte Ihnen erstmal so, was Ihnen so einfällt, Platz dafür lassen, sozusagen. Ähm es geht um das Gefühl, wie- wie bin ich da in der Welt? Präsent in der Welt? Ähm bin ich ich selbst? Oder vielleicht jemand anders? Oder ähm bin ich anwesend im Hier und Jetzt? |
| 497 | **IH12:** Also, als ich das erste Mal krank war, hatte ich irgendwie die Ein- bei uns ?(daheim)?, da waren ja sechs- waren ja vier Kinder (I: Hm (zustimmend)) und es hatte immer jeder seinen Platz (I: Mh-h (zustimmend)) am Tisch. Und in meiner ersten Psychose habe ich gedacht, wenn ich mich jetzt an den Platz, dann bin ich die eine Schwester (I: Mh-h (zustimmend)). Und wenn ich mich hier hinsetze bin ich die andere [LACHT VERLEGEN] Schwe- weil ich weiß ja nicht, ob Sie es- |
| 498 | **I:** Das- das wäre so etwas, zum Beispiel, da hatten Sie- |
| 499 | **IH12:** Das habe ich, aber das war nicht lang. |
| 500 | **I:** Okay. Nur einmal? Das ist nur einmal vorgekommen? |
| 501 | **IH12:** Das war eigentlich nur bei der ersten Psychose. |
| 502 | **I:** Okay. Da hatten Sie das Gefühl, wenn Sie etwas Bestimmtes machen, wenn Sie sich auf einen bestimmten Platz setzen, dann bin ich jemand anders. Und denke und fühle und handle wie die andere Person? |
| 503 | **IH12:** Aber was ich als Kind auch immer gemacht habe, und es ist vielleicht auch ein Auslöser für die Psychose gewesen, ich weiß es ja nicht (I: Mh-h (zustimmend)): Ich habe mir immer Geschichten überlegt. |
| 504 | **I:** Okay. Was für Geschichten? |
| 505 | **IH12:** [LACHT] ich hatte da eine ganze Familie mir zusammemgesponnen. |
| 506 | **I:** Die um Sie herum existiert? |
| 507 | **IH12:** Ja |
| 508 | **I:** Mh-h. Die in Wirklichkeit sozusagen nicht- |
| 509 | **IH12:** Ich hab es immer gewusst, dass es nur Geschichten sind (I: Ja.), die ich mir abends im Bett überlege (I: Ja, okay). Und da habe ich mir halt immer so ganz intensiv diese Geschichten vorstellt. Da hatte ich, was weiß ich, eine Zwillingsschwester, einen kleinen Bruder und noch eine andere Schwester und so. Und die hatten alle irgendwie eine Besonderheit. Alsp sprich die Zwillingsschwester war Gehörlos (I: Mh-h (zustimmend)), die eine Schwester saß im Rollstuhl (I: Mh-h (zustimmend)), der eine Sohn, Bruder konnte nicht richtig sprechen (I: Mh-h (zustimmend)). Und als ich dann in der Psychose war, habe ich dann wirklich gedacht, dass ich diese verschiedenen Personen bin. Und vorher waren es ja nur Geschichten. Das wusste ich genau, dass es alles nur erfunden ist (I: Ja.), aber in der Psychose habe ich das wirklich- habe ich dann plötzlich gedacht, ich kann nicht mehr laufen (I: ah) oder ich kann nicht mehr hören. |
| 510 | **I:** Mh. Und Sie waren alle diese Personen gleichzeitig (IH12: ja) oder zu unterschiedlichen Zeiten? |
| 511 | **IH12:** Also wie es halt gerade war. Als ich nicht laufen konnte- obwohl, da war es dann alles ich. In der Psychose war alles ich (I: Mh-h (zustimmend)). Vorher waren es die verschiedenen Personen, in der Psychose war es dann ich, wo ich nicht mehr laufen konnte. |
| 512 | **I:** Das heißt, es gab Momente, wo Sie keine Kontrolle mehr über Ihren eigenen Körper hatten? Also plötzlich konnten Sie nicht mehr laufen sozusagen? |
| 513 | **IH12:** Ja. Also, ich- ich die haben mich dann da gebadet und dann haben Sie mich da raus und dann musste ich in das ähm Krankenzimmer hinterlaufen, das war so ein Sechsbettzimmer, den Gang entlang. Und da habe ich gesagt: Ich kann nicht laufen! Und dann haben Sie gesagt: Ach was, natürlich können Sie laufen. Und dann die Ärzt- die Schwester gesagt: Jetzt treten Sie mal fest auf. Und so. Und mein Vater hat mich da laufen gesehen, der hat gesagt: Das war sein schlimmster Moment seines Lebens gewesen, mich da laufen sehen. |
| 514 | **I:** Mh-h. Das ist dann- hat sich aber wieder verändert? Also Sie konnten dann- |
| 515 | **IH12:** Ich konnte dann laufen aber ich- ich- ich hatte wirklich- (I: das Gefühl) wirklich das Gefühl, ich kann jetzt nicht laufen (I: Okay, und-). Ich habe dann auch ganz fest aufgestampft [MACHT STAMPENDE GERÄUSCHE AUF DEM BODEN] und habe dann gesagt: Ich kann doch laufen. Und so. |
| 516 | **I:** Okay. Und ähm das war das einzige Mal, dass diese- diese- diese mh diese Verbindung zum Körper verloren gegangen ist? Oder gab es andere Situationen, in denen Sie auch das Gefühl hatten, Ihr Körper macht jetzt nicht mehr, was (IH12: in der Psychose?) eigentlich geht. Äh in der Psychose, aber auch gerne danach oder davor. |
| 517 | **IH12:** [ATMET EIN UND AUS]. Das war das einzige. |
| 518 | **I:** Das einzige Mal. Mh-h. |
| 519 | **IH12:** Mh, obwohl, ja. Ja. Einmal ist es mir passiert, beim Bratschespielen. Da habe ich ein Stück gespielt, das ich früher gespielt habe, als ich so- ich äh als ich psychotisch war, habe ich ja so Bratsche gespielt. Und da habe ich das total intensiv geübt und gemacht und getan und da habe ich das noch Jahre immer wieder gespielt, das Stück. Und dann kam es mir plötzlich vor, als wäre da eine Wand. Ich konnte meine Hände nicht mehr- |
| 520 | **I:** bewegen? |
| 521 | **IH12:** Nee, ich konnte sie schon bewegen, aber ich- ich bin mit meinen Gedanken nicht mehr hingekommen [LACHT] (I: Okay.). Ich habe plötzlich einen Blackout gehabt. |
| 522 | **I:** Die haben sich dann auch nicht mehr bewegt, die Hände? Oder ging das noch, ohne dass Sie sozusagen Einfluss nehmen? |
| 523 | **IH12:** Ich habe halt dann- ich habe halt dann pausiert [LACHT] (I: Okay.) Ich habe- ich habe- (I: Ja.) ich habe aber- |
| 524 | **I:** Das ging plötzlich nicht mehr? |
| 525 | **IH12:** Ja und dann- und dann, das war aber nur bei der einen Stelle und auch nur beim ersten Mal, wo ich plötzlich- (I: Mh-h (zustimmend)) weil ich halt das so oft gespielt hatte und so geprobt- geübt hatte. Und so. Und da- da war ich halt schon, ja. Der Stress wahrscheinlich (I: Hm (zustimmend)). Habe ich das doch Jahre noch mal gespielt und dann hatte ich plötzlich dieses- |
| 526 | **I:** Dieses Blackout, dieses körperliche Blackout? |
| 527 | **IH12:** Wenn das- wenn das damit gemeint ist? Weiß es ja nicht. |
| 528 | **I:** Ja, zum Beispiel, ja. Das ist damit gemeint. Und ähm dieses Gefühl, keine Identität zu haben? Keinen Kern, anonym, nicht existent zu sein? Das hatten Sie gesagt, gibt es eher nicht? Oder gab es nie? |
| 529 | **IH12:** Nee. |
| 530 | **I:** Nee? |
| 531 | **IH12:** Mh-h (verneinend). |
| 532 | **I:** Okay. Und ähm Gedanken, Handlungen und Gefühle gehörten immer zu Ihnen selbst? Oder gab es Momente, wo die nicht zu Ihnen gehören- gehörten? Vorhin hatten- in- in der Psychose, haben Sie gesagt? (IH12: [KLAPPERT MIT DEM TELLER UND MACHT KAUENDE GERÄUSCHE] Hm). Oder wo Sie das Gefühl hatten, da ist eine Distanz zu meinen Gedanken? In der Psychose hatten wir das schon. Gab es da andere Momente? |
| 533 | **IH12:** Hm. Hm. Weiß es nicht. |
| 534 | **I:** Ja, wenn Sie so lange überlegen müssen, höchstwahrscheinlich (IH12: [LACHT]) [crosstalk]. Ja. Ähm. Ist es so, dass Sie häufig beobachten, so ein bisschen wie von außen, was Sie tun? Und wie Sie es tun? |
| 535 | **IH12:** Nee. |
| 536 | **I:** Nee? Sie denken nur hinterher darüber nach, wenn es passiert ist. Aber im Moment sind Sie voll mit drin und- und beobachten nicht von außen oder denken- |
| 537 | **IH12:** Ich habe mich noch nie von außen beobachtet. |
| 538 | **I:** Okay. Okay. Mh. Genau. Und Sie hatten auch nicht manchmal das Gefühl, sich selbst fremd zu sein? Nicht so richtig Sie selbst zu sein? |
| 539 | **IH12:** Nee, auch nicht (Wasser gießt ein). Mh. |
| 540 | **I:** Oder weniger zu fühlen, weniger zu empfinden als vorher? Weniger involviert zu sein in die Welt? |
| 541 | **IH12:** Ja, ich meine, ich konnte ja auch- das war ja auch, wo ich bei der ersten Psychose- ich saß ja nur im Gang und [LACHT VERLEGEN] ja, das war ja auch furchtbar. Dann war da so eine Putzfrau, ich weiß ja auch nicht, die ist die ganze Zeit immer nur hin und her gelaufen [LACHT] mit so einem Besen (I: Hm (zustimmend)). Ich weiß auch nicht, was sie so lange an einer Stelle immer geputzt hat. |
| 542 | **I:** [LACHT] Und Sie saßen da und haben zugeguckt, ja? |
| 543 | **IH12:** [LACHT] und dann saß neben mir irgendeiner und der konnte nur Englisch. Und in meiner Psychose habe ich ja gedacht, ich könnte perfekt alle Sprachen sprechen (I: Mh-h (zustimmend)) [LACHT]. Und dann- und da habe ich es tatsächlich- in [ANONYMISIERT, ORT IN DEUTSCHLAND] habe ich das auch gedacht, ja, dass jemand anderes meine Gedanken lesen kann (I: Mh-h, mh-h (zustimmend)). Da saßen wir dann im Raucherraum. Ich weiß auch nicht, wieso ich da reingegangen bin, weil ich ja gar nicht rauche. Und da war halt so ein Mann und ich habe die ganze Zeit gedacht, dass ich mich mit dem unterhalte. Also meine Gedanken sich mit dem seinen Gedanken unterhält [/unterhalten/]. |
| 544 | **I:** Obwohl Sie nicht gesprochen (IH12: nee) haben? |
| 545 | **IH12:** Nicht gesprochen (I: Hm (zustimmend), okay). Ich weiß auch nicht, wieso ich da im Raucherraum saß [LACHT]. Komisch. |
| 546 | **I:** Sie rauchen selbst gar nicht? |
| 547 | **IH12:** Nee. |
| 548 | **I:** Sie haben nie geraucht? |
| 549 | **IH12:** Mh-h (verneinend). |
| 550 | **I:** Okay. Ähm [GLAS WIRD AUF TISCH ABGESTELLT]. Also dieses Gefühl, nicht so richtig anwesend zu sein, nicht so richtig in der Welt zu sein- |
| 551 | **IH12:** Ja gut, in der Psychose ist man natürlich dann schon auch bisschen lahm gestellt. |
| 552 | **I:** Ja, klar, durch die Medikamente. Aber mehr so- ich meine das eher so ein generelles, grundlegenderes Gefühl ähm dass die Welt nicht so richtig zu Ihnen durchdringt. Oder dass da ein Schleier ist. Oder so eine Wand zwischen Ihnen und der Welt? Das gibt es nicht? |
| 553 | **IH12:** Mh-mh (verneinend). |
| 554 | **I:** Okay. Ähm dann hatten Sie gesagt, dass manchmal Sie Dinge anders wahrnehmen. Heller (IH12: heller), intensivere Farben (IH12: langsamer), langsamer, schneller. Ähm- |
| 555 | **IH12:** Aber das ist eigentlich, wenn dann etwas Bedrohliches (I: genau, das sind-), Schock (I: ganz bestimmte Situationen). Ich glaube, das ist tatsächlich der Schock (I: Mh-h (zustimmend)), also der Schockmoment. Wo das Adrenalin oder was weiß ich. |
| 556 | **I:** Ja, ja. Das sind- das ist kein grundle- keine grundlegende (IH12: nee) Geschichte (IH12: nee), wo das immer mal wieder kommt und geht oder so für einen längeren Zeitraum? Nee. Sondern nur in diesen Momenten. Mh-h. Okay. Und Sie hatten auch gesagt, dieses Beobachten, was tue ich gerade? Mache ich das richtig? Mache ich es ähm sollte ich es anders machen vielleicht? Oder ähm das ist bei Ihnen nicht so, sondern nur am Ende des Tages, dass Sie darüber nachdenken, was ist so gelaufen am Tag? |
| 557 | **IH12:** Ja ich mache mir natürlich ständig Gedanken (I: Ja.), das fängt schon damit an, welchen Stempel ich jetzt auf den Brief machen soll. |
| 558 | **I:** Okay, das sind wieder die Entscheidungen. |
| 559 | **IH12:** Achso, das sind Entscheidungen. |
| 560 | **I:** Ja, genau, was mache ich jetzt? |
| 561 | **IH12:** Aber mein Seniorchef, der ist ja so nett zu mir (I: Hm (zustimmend)). Der ist auch ganz auf mich fixiert, wenn der irgendetwas hat, muss ich immer kommen. Und dann zeigt er mir immer ganz genau: Jetzt machen Sie davon drei Kopien, wenn ich das gemacht habe: Jetzt scannen Sie das ein. Jetzt habe ich da einen Brief, da kleben Sie eine Briefmarke drauf, machen den [ANONYMISIERT, NAME DES STEMPELS DES UNTERNEHMENS, IN DEM DIE INTERVIEWTE PERSON ERWERBSTÄTIG IST] drauf. Jetzt machen Sie mir einen Briefumschlag mit dem und- und der sagt mir wirklich alles, was ich- und das ist genau das, was ich brauche. |
| 562 | **I:** Mh-h. Schön. Das ist gut, dass Sie so eine Arbeitsstelle gefunden haben? |
| 563 | **IH12:** Ja. |
| 564 | **I:** Ja. |
| 565 | **IH12:** Und- und- und der Juniorchef- der Seniorchef ist ja jetzt schon 89 (I: Mh-h (zustimmend)), der Leiter ist ja schon so alt. |
| 566 | **I:** Wow. Arbeitet noch mit mit 89? |
| 567 | **IH12:** Ja, Montag, Mittwoch, freitags. |
| 568 | **I:** Wahnsinn. |
| 569 | **IH12:** Und ähm der Juniorchef ist aber auch total nett zu mir. Da kann ich jederzeit anrufen, wenn irgendetwas ist. |
| 570 | **I:** Schön. Gut, das heißt aber, Sie haben, das hatten wir ja, Sie hatten die Entscheidungsschwierigkeiten (IH12: Mh-h (zustimmend) ähm aber in dem Moment, wenn Sie etwas machen (IH12: achso), da sind Sie sozusagen- da denken Sie nicht darüber nach: Wie mache ich es das jetzt gerade? |
| 571 | **IH12:** Nee. |
| 572 | **I:** Und- sondern- wenn dann, vielleicht- |
| 573 | **IH12:** Wenn ich da sitze und mein Geld zähle, dann zähle ich mein Geld und- und- und- |
| 574 | **I:** Gut, okay. Okay, verstehe. Ähm wie erleben Sie Ihr eigenes Alter? |
| 575 | **IH12:** Alter? Mh-h. Also ich fühle mich eigentlich noch nicht wie [ANONYMISIERT, ALTER DER INTERVIEWTEN PERSON]. |
| 576 | **I:** Nee? Sondern jünger? |
| 577 | **IH12:** Ja [LACHT]. |
| 578 | **I:** Ja? Ja, Sie wirken auch jung. |
| 579 | **IH12:** Jung, aber weiß auch nicht. ANONYMISIERT, ALTER DER INTERVIEWTEN PERSON]. |
| 580 | **I:** Aber es ist jetzt nicht so, dass Sie- dass Sie das Gefühl haben, mein- mein Alter passt überhaupt nicht zu mir? |
| 581 | **IH12:** Nö, das passt schon. Ich war heute in der S-Bahn gesessen und habe lauter so Jugendliche gesehen, und habe gesagt: Naja, also aus dem Alter bin ich jetzt schon längst raus. (I: Mh (zustimmend), okay). Also habe ich auch gedacht: Das- das könnten ja alles meine Kinder sein. |
| 582 | **I:** Ja, ja. Und das eigene Geschlecht? |
| 583 | **IH12:** Gut. Da habe ich Glück. Oh Gott (I: Ja?). Das gibt es ja tatsächlich, dass sich jemand nicht in seinem Körper wohl fühlt (I: Ja, genau.). Das ist furchtbar |
| 584 | **I:** Ja. Das ist bei Ihnen nicht so? |
| 585 | **IH12:** Habe ich zum Glück nicht. |
| 586 | **I:** Und auch was die sexuelle Orientierung angeht? Das ist auch ähm völlig und wird nicht infrage gestellt? Oder fühlt sich manchmal falsch an? |
| 587 | **IH12:** Nee. |
| 588 | **I:** Nee, okay. Mh. Hatten Sie schon mal Zeiten, in denen Sie das Gefühl hatten, Ihnen geht so ein bisschen die Selbstverständlichkeit im Alltag verloren? Also, dass- dass Sie das Gefühl haben, Sie wissen nicht mehr so genau, warum laufen die Dinge eigentlich so und nicht anders? Und wieso machen wir das so? Wieso schütteln wir uns zum Beispiel die Hand, wenn wir Hallo sagen? Oder, also nur als Beispiel, ne? Solche Erlebnisse? |
| 589 | **IH12:** Achso, dass es sich falsch anfühlt? |
| 590 | **I:** Mh. Oder dass Sie hinterfragen? |
| 591 | **IH12:** Warum man sich die Hände schüttelt? |
| 592 | **I:** Ja, das ist nur ein Beispiel. Grundsätzlich so, wie die Welt läuft. Ob das- ob das so richtig ist, oder ob es eigentlich anders laufen könnte, auch. |
| 593 | **IH12:** [LACHT] in meiner Psychose habe ich gedacht, es gibt kein Geld mehr (I: Ah, okay). Ich habe gedacht, jeder arbeitet und nimmt sich das, was er braucht. |
| 594 | **I:** Das wäre ein Vorschlag gewesen, oder das- (IH12: nee) das wäre, das war so? In der- in der Psychose? |
| 595 | **IH12:** Das- ich weiß es nicht, ob es ein Vorschlag war, oder, das war so. Dann kam noch mein Onkel, der hat uns immer Münzen geschenkt. Und dann habe ich ihm gesagt: Ach, das sind jetzt die letzten Münzen. Hat er gelacht. So kann man es auch sehen [LACHT]. |
| 596 | **I:** Okay. Grundsätzlich fragen Sie sich aber nicht, wieso haben wir Geld und bezahlen Sachen damit? Oder wieso laufen die Dinge so und nicht anders? Also grundsätzlich. |
| 597 | **IH12:** Grundsätzlich nicht (I: Hm (zustimmend)). Es gibt ja auch verschiedene Kulturen. |
| 598 | **I:** Ja, das stimmt. Und da werden die Dinge unterschiedlich gemacht. |
| 599 | **IH12:** So ist es [LACHT] (I: ja, ja, okay). So ist es, ja, ja. |
| 600 | **I:** Was- was- was heißt es für Sie, dass es unterschiedliche Kulturen gibt? Was meinen Sie damit? |
| 601 | **IH12:** Es gibt zum Beispiel eine Kultur, da sagt man sich guten Appetit, indem man (Klopft auf Tisch) so macht. |
| 602 | **I:** Okay (IH12: [LACHT]). Okay. Also man- das bedeutet, man kann die Dinge auch anders sehen, für Sie? Oder anders machen? |
| 603 | **IH12:** Ja. |
| 604 | **I:** Okay. |
| 605 | **IH12:** Mh-h. |
| 606 | **I:** Und dann hatten Sie vorhin erzählt, dass Sie Angst haben, Auto zu fahren? |
| 607 | **IH12:** Ach, lieber Gott, Auto fahre (I: Ja.) ich überhaupt nicht. |
| 608 | **I:** Nee? Okay. |
| 609 | **IH12:** Ich habe zwar einen Führerschein [LACHT]. |
| 610 | **I:** Ja? Davor- also ich wollte Sie jetzt nach Ängsten- gibt es bestimmte (IH12: Ja) Ängste, die Sie haben? |
| 611 | **IH12:** Ich habe im Auto Ängste (I: Mh-h (zustimmend)). Ich habe wirklich Ängste. Wenn ich da mitfahre nur, dann denke ich, wenn jetzt der entge- uns passiert ja auch oft, wenn ich in der Zeitung lese, fast (I: Hm (zustimmend)) jeden Tag ist jemand von der Fahrbahn abgekommen. |
| 612 | **I:** Ja? Okay. Also Sie haben Angst vor dem Autofahren? |
| 613 | **IH12:** Ja. |
| 614 | **I:** Okay. Und ähm- |
| 615 | **IH12:** Ich bin sogar mal aus dem Bus ausgestiegen, weil der so schnell gefahren ist. Und jetzt sage ich mir, wenn ich im Bus sitze und plötzlich- jetzt fahre ich ja schon so lange Bus (I: Hm (zustimmend)). Jetzt mache ich mir auch nicht mehr die Gedanken (I: Hm (zustimmend)). Weil dann denke ich mir: Das ist ja dem sein Beruf (I: Ja, ja.). Ich mache ja auch meinen Beruf (I: Ja.), mache ja auch das Geld richtig (I: Ja.) und dann wird der auch fahren können. |
| 616 | **I:** Okay. Aber im Bus- also früher hatten Sie auch beim Busfahren Angst? |
| 617 | **IH12:** Ja. |
| 618 | **I:** Gibt es noch andere Sachen, vor denen Sie Angst haben? |
| 619 | **IH12:** Über schmale Brücken laufen. Ich war in [ANONYMISIERT, ORT IN DEUTSCHLAND] und konnte nicht übers Blau runterlaufen (I: Mh-h (zustimmend)). Bin nicht rübergekommen. |
| 620 | **I:** Okay. Das war zu viel? |
| 621 | **IH12:** Das war- da hatte man auch so durchsehen können. |
| 622 | **I:** Ah, verstehe, okay. Mh-h. |
| 623 | **IH12:** Das und- und- und war ich ja auch- das habe ich früher nicht gehabt: Ich kann auch nicht enge, also so Bergwanderungen, so- so- so- so- so Gratwanderungen (I: Mh-h (zustimmend)) kann ich nicht. |
| 624 | **I:** Also Angst vor Höhen auch? |
| 625 | **IH12:** [LACHT] der Seniorchef ist mit mir nach [ANONYMISIERT, ORT IN DEUTSCHLAND] gefahren. Wir haben Inventur gemacht. Und der hatte da so ein- so ein Fahrzeug gehabt, das war vielleicht gerade mal so groß, wie der Tisch, wo man da saß (I: Mh-h (zustimmend)) und der hatte da so eine Kette da vorne gehabt. Und dann ging das erst die unteren Paletten, haben wir alles aufgeschrieben. Dann ging es zur nächst höheren Palette, hat er wieder aufgeschrieben, ich habe alles aufgeschrieben. Dann ging es nochmal höher und dann ist der [ANONYMISIERT, NAME DES VORGESETZTEN DER INTERVIEWTEN PERSON] so: So mal da gucken. Und ich dann so: Bitte, [ANONYMISIERT, NAME DES VORGESETZTEN DER INTERVIEWTEN PERSON], tun Sie mir einen gefallen (I: Ja.), bleiben Sie jetzt mal da sitzen. Und da hat er mich angeguckt: Was haben Sie denn Frau [ANONYMISIERT, NAME DER INTERVIEWTEN PERSON] [LACHT]. Ich habe solche Angst gehabt. Und dann hat er gesagt: Wissen Sie, Frau [ANONYMISIERT, NAME DER INTERVIEWTEN PERSON], ich mache das mit jemand anderem [LACHT]. |
| 626 | **I:** [LACHT]. Okay, das heißt ähm? |
| 627 | **IH12:** Höhenangst habe ich. |
| 628 | **I:** Höhenangst, okay. |
| 629 | **IH12:** Ich kann auch nicht vom Fünfmeterbrett springen. |
| 630 | **I:** Ja, da- okay. Das- das kenne ich auch. |
| 631 | **IH12:** [LACHT] Ich bin einmal hoch und bin die Treppen wieder runter. |
| 632 | **I:** Mh. Und wenn Sie dann so Ängste, also zum Beispiel auch beim Autofahren, oder beim Busfahren haben, spüren Sie das dann auch richtig körperlich? |
| 633 | **IH12:** Ich habe schweißnasse Hände. |
| 634 | **I:** Hm. Und vielleicht einen schnelleren Herzschlag? |
| 635 | **IH12:** Ja. |
| 636 | **I:** Oder so etwas. Ja? |
| 637 | **IH12:** Ich schwitz dann sofort. Und einmal bin ich mit einer gefahren, die hat so gesagt- ach Gott, war die goldig, die hat bei der Tankstelle eine Pause eingelegt [LACHT] damit ich mich wieder erholen kann [LACHT]. |
| 638 | **I:** Ja? Das war eine Mitfahrgelegenheit oder? |
| 639 | **IH12:** Da war- nee, da war ich bei einer Freundin und wir haben uns da getroffen. Irgendwie Geburtstag oder was weiß ich. Und da hat die mich mit Auto mitgenommen. |
| 640 | **I:** Okay, okay |
| 641 | **IH12:** Und dann hat die das auch gemerkt, dass ich solche Ängste habe. Und dann hat sie gesagt: Wir machen jetzt hier mal Zwischenpause. Und da kam aber noch erschwerend hinzu, dass die ihr Hündchen auch noch auf dem Schoß hatte. |
| 642 | **I:** Beim Autofahren? |
| 643 | **IH12:** Ja. Und dann hat sie gesagt: Ich mache das immer, auf jeder Autobahn. Brauchst keine Angst zu haben. |
| 644 | **I:** Okay. Das heißt, es sind bestimmte Dinge, bestimmte ähm Ativitäten, vor denen Sie Angst haben? |
| 645 | **IH12:** Ja. |
| 646 | **I:** Aber keine sozialen Situationen? Keine anderen Menschen? |
| 647 | **IH12:** Oh, da habe ich auch manchmal Angst. |
| 648 | **I:** Können Sie da ein Beispiel sagen? |
| 649 | **IH12:** Oh. Ja, ich hatte schon vor meiner Chefin schon ein bisschen Angst, weil die hat schon auch bisschen gefordert hat immer. |
| 650 | **I:** Mh-h. Die Chefin, die jetzige Chefin? |
| 651 | **IH12:** Mh-h. Ja, ja, die ist- die ist ja die Frau vom Seniorchef. |
| 652 | **I:** Okay. Okay. Und- |
| 653 | **IH12:** Und- und- und das war halt auch schwierig. Die hat gesagt. Frau [ANONYMISIERT, NAME DER INTERVIEWTEN PERSON], schreiben Sie jetzt mal den Brief. Schreiben Sie das und das und das und das und das. Und ich wollte da mitschreiben. Und dann hat Sie- hat Sie- und dann habe ich gesagt: Können Sie es nochmal sagen? Und dann hat sie dasgleiche nochmal völlig anders ausgedrückt. |
| 654 | **I:** Mh. |
| 655 | **IH12:** Und dann saß ich da. Ja, da hatte ich schon ein bisschen Angst, ja. (I: Achso) Vielleicht war es das auch, ich weiß es nicht, vielleicht die Angst vorm Fa- Falschmachen. |
| 656 | **I:** Hm. Also, Sie haben Angst dann etwas falsch zu machen? |
| 657 | **IH12:** Ja. |
| 658 | **I:** Okay. Und, und- |
| 659 | **IH12:** Dabei brauche ich vor Sie- Ihr gar keine Angst zu haben. Ich habe das erst jetzt erfahren, von einer ehemaligen Kollegin, dass ihr Sohn mich eigentlich gar nicht mehr nehmen wollte nach der Psychose. Ich war ja fast ein dreiviertel Jahr krank (I: Ja, ja). Nach fünf Jahren, wo ich dort angefangen hatte. Und dann hat sich die [ANONYMISIERT; FRAU DES VORGESETZTEN DER INTERVIEWTEN PERSON] eingesetzt und hat gesagt: Die Frau [ANONYMISIERT, NAME DER INTERVIEWTEN PERSON], die wollen wir behalten. |
| 660 | **I:** Schön, ja. |
| 661 | **IH12:** Also im Grunde ist die mir nur wohlgesonnen. Und die hat sich mich ausgesucht, als wir umgezogen sind, dass ich bei ihr im Büro sitze (I: Okay.). Die hätte sich jeden anderen Kollegen aussuchen können (I: Okay, okay.). Und deswegen brauche ich da echt keine Angst haben. |
| 662 | **I:** Okay. Und ähm aber Sie fürchten jetzt nicht bedroht zu werden, oder verfolgt, oder beobachtet? |
| 663 | **IH12:** Also ich habe- muss ehrlich sagen, mir ist- ich habe schon kein gutes Gefühl, wenn ich da nachts ähm durch die Straßen laufe, aber das ist vielleicht auch berechtigt. |
| 664 | **I:** Mh-h. Was könnte passieren? |
| 665 | **IH12:** Dass Plötzlich mich jemand angreift? |
| 666 | **I:** Mh. Okay. Dass Sie jemand verfolgt, denken Sie auch manchmal? |
| 667 | **IH12:** Verfo- ja, nee, einfach, dass jemand- |
| 668 | **I:** Bedroht? |
| 669 | **IH12:** Ja, oder- oder vergewaltigt, oder so (I: Hm (zustimmend)). Also da habe ich schon ein bisschen Angst. |
| 670 | **I:** Okay. Okay. Verstehe. Ähm das- aber ist auch eine ganz spezifische Angst, also (IH12: Mh-h (zustimmend)) es ist nicht einfach eine- so ein ungutes Gefühl, die ganze Zeit, das da ist, sondern (IH12: nee) da geht es um- es geht um bestimm- bestimmte Situationen? |
| 671 | **IH12:** Ja, also ich habe nicht das Gefühl, dass jemand mich [/mir/] hinterherläuft. |
| 672 | **I:** Mh-h. Okay. Und das ist, wenn es dunkel ist draußen? |
| 673 | **IH12:** Und was ich natürlich- wo ich besonders viel Angst habe, ist vor Gewitter. |
| 674 | **I:** Mh-h. Okay. |
| 675 | **IH12:** Da war ich ja auch letztes Jahr im Urlaub und dann habe ich so Angst gehabt, dass bei dem Ausflug da ein Gewitter ist. Aber kam zum Glück bei dem Ausflug kein Gewitter. Jeden Abend war ein Gewitter, nur bei dem Ausflug nicht (I: [LACHT]). Und als es dann geregnet hat, hat mich die [ANONYMISIERT, NAME EINER FREUNDIN DER INTERVIEWTEN PERSON] gefragt: Und [ANONYMISIERT, NAME DER INTERVIEWTEN PERSON], hast du Angst? Also am anderen Abend (I: Mh-h (zustimmend)). Da habe ich gesagt: Nee, heute habe ich keine Angst. Heute ist auch kein Gewitter. Und die konnten das ja nicht wissen, ob Gewitter ist, weil die das ja nicht gehört hat. |
| 676 | **I:** Sie hat nur den Regen gehört? Oder- |
| 677 | **IH12:** Nein, die hatte- hörte ja gar nichts. |
| 678 | **I:** Wer- wer hört nichts? (IH12: [LACHT]) Jetzt bin ich gerade- jetzt bin ich gerade nicht mitgekommen. Wie- wer hört nichts? |
| 679 | **IH12:** Ich- ich war- ich fahre- mache schon seit Jahren so ähm Urlaub in [ANONYMISIERT, ORT IN DEUTSCHLAND] und das ist so ähm das wird von so einer Gebärdensprachschule organisert (I: Okay, okay) und die zwei, die das organisieren, sind beide gehörlos. |
| 680 | **I:** Verstehe, okay (IH12: und deswegen-). Das- den Kontext hatte ich nicht mehr- |
| 681 | **IH12:** [LACHT] genau, das habe ich auch nicht erzählt [LACHT]. |
| 682 | **I:** Ja, okay. Und sie konnte nichts hören, ob Gewitter ist oder nicht. Sie hat nur den Regen gesehen (IH12: genau), sozusagen. Okay, verstehe. |
| 683 | **IH12:** Und dann habe ich gesagt: Oh Gott, es re- es ist Gewitter, ich habe schon ein bisschen Angst. Sie hat ja auch gesehen, dass ich total Angst habe (I: Hm (zustimmend)). Und dann am nächsten Tag hat es ja dann nur geregnet und dann hat sie mich gefragt: Und [ANONYMISIERT, NAME DER INTERVIEWTEN PERSON], hast du wieder Angst? Nee, heute ist ja kein Gewitter. (I: [LACHT], okay). Also vor Regen habe ich keine Angst. |
| 684 | **I:** Gut. Okay. Ähm. Und, das ist so ein bisschen ähnlich, wie was ich vorhin schon gefragt habe. Das Gefühl, bei vollem Bewusstsein zu sein und äh und trotzdem in Watte eingepackt zu sein? Nicht so richtig durchdringen zu können zu anderen oder zur Welt? Ohne dass man Alkohol getrunken hat, ohne dass man irgendwas- |
| 685 | **IH12:** Ja also ich habe das ja, wenn ich aufgeregt bin, dass ich dann so eine pelzige Zunge habe (I: Mh-h (zustimmend)), denke, ich falle gleich in Ohnmacht. Das ist halt dann bei so Konzerten. |
| 686 | **I:** Wenn Sie im Chor vorne singen sollen? |
| 687 | **IH12:** Aber es ist viel besser geworden im Gottesdienst. Singen wir jetzt auch meistens hinten und wenn wir hinten sind, rege ich mich gar nicht auf. Und bei den jungen Konfirmationen habe ich kurzfris- kurz vorher erfahren, dass wir jetzt doch vorne singen und dann gings- dann ging natürlich los die Angst und (I: Hm (zustimmend)) das Herzklopfen (I: Hm (zustimmend)) und dann stand ich da vorne. Und da dachte ich mir, wenn ich mich jetzt schon bei dem einen Lied im Gottesdienst so aufrege, wie soll ich dann so ein Konzert überstehen? |
| 688 | **I:** Okay. Und dann haben Sie sich jetzt dagegen entschieden? |
| 689 | **IH12:** Dagegen entschieden. Ich habe es aber noch niemandem gesagt. Aber so ist es jetzt halt. |
| 690 | **I:** Okay. Und das ist erleichternd für Sie? |
| 691 | **IH12:** Ja. |
| 692 | **I:** Ja? Gut. |
| 693 | **IH12:** Da ist mir jetzt echt ein Fels vom Herzen gefallen, dass ich da nicht mitsinge. |
| 694 | **I:** Ein ganzer Fels gleich? |
| 695 | **IH12:** Ich bin auch keine tragende Stimme, ich kann das überhaupt noch nicht (I: Okay, okay). Und wir sind so viele im Alt. |
| 696 | **I:** Da werden sie das bestimmt verstehen. |
| 697 | **IH12:** Ja. |
| 698 | **I:** Ja. |
| 699 | **IH12:** Ich kann das einfach nicht. |
| 700 | **I:** Okay. Ähm gibt es Zeiten, in denen Sie das Gefühl hatten oder gab es Zeiten, dass Sie Dinge, die Sie normalerweise äh die Ihnen normalerweise Spaß machen plötzlich nicht mehr interessieren? Oder keinen Spaß mehr machen? Essen, Hobbies, Sport, soziale Interaktionen, Sex vielleicht? |
| 701 | **IH12:** Naja das ist halt, wenn ich morgens dann aufwache, habe ich im ersten Moment überhaupt keine Lust zu nichts (I: Okay.). Oder auch nachmittags, wenn ich mich hinlege, und dann, null Lust. Und wenn ich dann aufstehe, dann fällt mir wieder ein, was ich machen könnte. |
| 702 | **I:** Mh-h. Aber kurz ist das Gefühl einmal weg. Ja? Die- die (IH12: morgens, ja) die Motivation, sozusagen. |
| 703 | **IH12:** Sonst habe ich ja eigentlich schon viel Interessen. |
| 704 | **I:** Gut. Also viele Hobbies, wie das Singen und- |
| 705 | **IH12:** Singen habe ich, Bratschespielen habe ich (I: Mh (zustimmend)), Gebärdensprache habe ich (I: genau), Tennis, Schwimmen (I: Ja, alles klar), malen. |
| 706 | **I:** Und das machen Sie auch alles? |
| 707 | **IH12:** Hm (zustimmend). |
| 708 | **I:** Ja? Okay. |
| 709 | **IH12:** Also im So- im Winter natürlich kein Tennis (I: Hm (zustimmend)), das habe ich ja schon erzählt, dass ich da nicht gespielt habe. |
| 710 | **I:** Ja, ja, das haben Sie erzählt. |
| 711 | **IH12:** Schwimmen ist auch eher- aber ich möchte mir jetzt dieses Jahr, das fand die [ANONYMISIERT, NAME EINER FRAU] auch so toll, Schwimmbadkarte kaufen (I: Okay.). Weil ich- dass ich mich einfach öfters auch dann bewege (I: Ja.). Dann kann ich morgens, wenn ich es schaffe- |
| 712 | **I:** Wenn Sie rauskommen (IH12: wenn ich es schaffe [LACHT]), ja. Ja. Ist denn ähm das Gefühl, wenn Sie morgens nicht aufstehen können, ist es dann so ein grundsätzliches Keine-Energie-, Keinen-Elan-Haben? |
| 713 | **IH12:** Och einfach kein- kein Interesse aufzustehen. Einfach keine Lust, einfach. |
| 714 | **I:** Das geht aber dann wieder weg? Sobald Sie aufgestanden sind? |
| 715 | **IH12:** Ja, ich brauche dann so- eine Viertelstunde brauche ich schon. |
| 716 | **I:** Hm. Okay. Und das bleibt nicht den ganzen Tag? |
| 717 | **IH12:** Nee. |
| 718 | **I:** Aber ist jeden Tag so? |
| 719 | **IH12:** Ja. |
| 720 | **I:** Mh-h. |
| 721 | **IH12:** Und dann wenn ich morgens aufwache- |
| 722 | **I:** Durchgängig? |
| 723 | **IH12:** Ja. Ich habe dann immer irgendwas Schönes geträumt und bis ich mich wieder in der Wirklichkeit zurecht finde, dann schießt es mir so hoch und dann die Angst, dass- weil irgendwann sind meine Eltern nicht mehr da. Und im Moment bin ich halt doch schon ziemlich [MACHT SCHMATZENDES GERÄUSCH MIT DEM MUND] eng noch. |
| 724 | **I:** Die passen auf Sie auf, sozusagen? |
| 725 | **IH12:** Nee ja, die, was weiß ich, wenn ich halt so doofe Fragen habe: Was soll ich denn jetzt machen? Dann fra- gehe ich halt doch zur [LACHT] Mutter. |
| 726 | **I:** Ja, ja. Ja. Und plötzlich haben Sie dann morgens Angst, dass sich das verändern könnte? |
| 727 | **IH12:** Das, ja. Meine Eltern sind jetzt auch bald 80. |
| 728 | **I:** Okay. Das ist eine Angst, die Sie schon länger haben? Oder die jetzt einfach mit der Zeit gekommen ist? |
| 729 | **IH12:** Die habe ich schon ganz lange. |
| 730 | **I:** Ganz lange, okay. Also, so eine Verlustangst (gleichzeitig IH12: könnte man so sagen), hm. Okay. |
| 731 | **IH12:** Ich habe auch immer- ich saß auch mit meinem Seniorchef, nur durch die Tür verbunden, noch im alten Büro- ich habe immer Angst um den gehabt. Schnauft dann noch, der sitzt dann auch manchmal so [MACHT KLAPPERNDES GERÄUSCH]. Ist ja halt schon- der ist auch schon 89 inzwischen (I: Ja.). Der liest da dann immer seine Zeitschriften. Und dann sitzt er manchmal so und dann habe ich schon immer einen Schreck gekriegt, wenn ich dann so um die Ecke geguckt habe, ob er noch schnauft [SPRICHT SEHR VORSICHTIG UND LEISE]. |
| 732 | **I:** Sie machen sich Sorgen um andere Leute auch? |
| 733 | **IH12:** Ja, ich habe dann gedacht: Was soll ich denn machen? 112 wählen? Und- und- und- und- und- und- und dann habe ich mir irgendwas überlegt, was ich ihn jetzt fragen könnte und so. Dann ist er so aufgewacht (I: [LACHT] ja). Und jetzt, wo ich jetzt oben bin- jetzt habe ich dieses- jetzt bin ich da ganz froh, dass ich sozusagen die Verantwortung auch jetzt nicht habe (I: Okay, also-). Weil ich habe schon gedacht, wenn der jetzt- wenn mit ihm etwas ist, dann muss ich dann- meine Mutter zieht mich da immer auf, die sagt dann immer: Ja ja, du weißt schon, 112 wählen (I: [LACHT]). Und einmal, ich bin da schon ein bisschen hysterisch, könnte man schon sagen (I: Mh-h (zustimmend)), da war irgendwie mein Vater auch irgendwie so dagesessen und da habe ich auch gedacht, der hätte irgendwas. Da habe ich dann auch 112 gewählt. [LACHT VERLEGEN] |
| 734 | **I:** Ah Sie haben tatsächlich die Polizei oder den Krankenwagen gerufen? |
| 735 | **IH12:** Ja (I: Mh (zustimmend)). Und dann haben die im Krankenwagen gefragt: Ja ähm ist es wirklich jetzt so schlimm? Weil ich ja ziemlich hysterisch wahrscheinlich geklungen habe (I: Okay.). Und so. Und dann kam mein Vater und: Nee, es ist doch gar nichts! Und so. Habe ich mir gedacht: Naja, okay, dann ist nichts [LACHT]. |
| 736 | **I:** Und dann sind die auch nicht gekommen? |
| 737 | **IH12:** Mh-mh (verneinend), die sind nicht gekommen. Aber wo ich da gestürzt bin, habe ich dann tatsächlich bei mir selber gedacht- ich habe dann auch plötzlich keine Luft gekriegt und dann habe ich die auch angerufen. Und die sind dann auch gekommen [LACHT VERLEGEN] (I: Ja, ja.). Aber das war dann auch, weil ich da so starke Antibiotika nehmen musste (I: Okay, okay). So ein starkes Antibiotikum nehmen musste. |
| 738 | **I:** Okay. Okay. Gut, also wenn man zusammenfassen würde, das heißt (IH12: Ja), Sie machen sich Sorgen um Ihre eigene körperliche Versehrtheit, sozusagen, in solchen bestimmten Situationen (IH12: Hm (zustimmend)), klar, wo man stürzt, natürlich. Aber auch- |
| 739 | **IH12:** Auch um andere, ja. |
| 740 | **I:** Um andere [RÄUSPERT SICH], und- |
| 741 | **IH12:** Auch um meine Schwester habe ich mir Sorgen jetzt Sorgen gemacht, die jetzt am Montag ihr Kind bekommt. |
| 742 | **I:** Hm, ob es Ihr gut geht, sozusagen? |
| 743 | **IH12:** Ob die das- ja. |
| 744 | **I:** Und das ist ein Verantwortungsgefühl, was Sie da empfinden? Belastet Sie das Gefühl? Oder, dass Sie- |
| 745 | **IH12:** Das ist ein Verlustgefühl. |
| 746 | **I:** Das sind einfach Ängste vor dem Verlust? Aber nicht so etwas wie: Ich müsste jetzt besser aufpassen oder ähm ähm könnte etwas falsch machen, in dem Sinne, dass ich nicht 112 wähle? Oder so etawas. |
| 747 | **IH12:** Ja, gut, da muss man schon schnell handeln (I: Ja.), wenn man irgendwas, einen Herzinfarkt hat oder so. |
| 748 | **I:** Ja. Aber es geht bei diesen- bei diesen Ängsten geht es eher um die Angst, die Leute zu verlieren? |
| 749 | **IH12:** Hm (zustimmend) (I: Ja, okay). Ja. Ja. |
| 750 | **I:** Okay. Wie sieht es zeit- wie sieht es bei Ihnen aus? Wie geht es Ihnen? |
| 751 | **IH12:** Gut. |
| 752 | **I:** Ja? Also wir haben noch- |
| 753 | **IH12:** Wie viel ist es denn noch? |
| 754 | **I:** Ähm einiges haben wir schon besprochen, also- |
| 755 | **IH12:** Ja, dann machen wir das lieber, weil sonst muss ich ja noch mal kommen, oder? |
| 756 | **I:** Machen wir es zu Ende? |
| 757 | **IH12:** Ja (I: Ja.), würde ich schon sagen. |
| 758 | **I:** Okay, dann kommen wir- |
| 759 | **IH12:** Wenn Sie noch können? |
| 760 | **I:** Ich kann äh ich kann. |
| 761 | **IH12:** Sie haben bestimmt ja auch einen langen Arbeitstag. |
| 762 | **I:** Ja, ja, ja. Seit heute morgen um halb neun. |
| 763 | **IH12:** Jetzt ist es halb acht (überrascht). |
| 764 | **I:** Ja. Wir machen gleich zu Ende. Ja? Also ich habe auch eine Pause zwischendrin machen können, also, kein Problem. |
| 765 | **IH12:** Fange erst um zwölf an, also. |
| 766 | **I:** Ja, das ist gut. Das ist schön. |
| 767 | **IH12:** Habe ich Glück. |
| 768 | **I:** Also der Bereich, der jetzt kommt, da geht es um Ihren Körper und wie sich Ihr Körper anfühlt (IH12: Mh-h (zustimmend)) ähm zum Beispiel ähm fühlt sich der Körper wie eine Einheit an? Oder haben Sie auch manchmal das Gefühl äh, dass er vielleicht sich ein bisschen fremd anfühlt? Oder sich Körperteile fremd anfühlen? Gibt es da besondere Ereignisse, an die Sie sich erinnern können? Oder Veränderungen vor oder nach der Psychose? |
| 769 | **IH12:** Eigentlich nicht. |
| 770 | **I:** Spontan (IH12: mh-mh (verneinend)) fällt nichts ein, okay. Und gab es schon mal die Situation, dass sich Ihre Gliedmaßen zum Beispel zu groß oder zu klein angefühlt haben? |
| 771 | **IH12:** Mh-mh (verneinend). |
| 772 | **I:** Nein. Ähm oder dass Sie vielleicht größer oder kleiner ausgesehen haben? Nein? |
| 773 | **IH12:** Hm. Bei meiner ersten Psychose war das so. Da wusste ich wirklich nicht mehr, wie groß ich bin. Da hatte ich so ein Plakat in meinem Zimmer hängen, vom Mädchen, mit dem Gebüsch außenrum. Da war ich so mit den Kindern im [ANONYMISIERT, AUSÜBUNGSSTÄTTE DES BERUFS]- gut ich war damals 19 (I: Hm (zustimmend)), mit ähm dass man da schon auch die Autoritätsperson ist. (IH12: ja?) In der Psychose wusste ich dann auch nicht mehr: Wie groß bin ich? Das Mädchen auf dem Poster, da habe ich meinen Vater gefragt: Wenn du das Poster anguckst, wie würdest du sagen, wie hoch sind die Büsche? (I: Mh-h (zustimmend)) Er hat dann gesagt: So ungefähr. Und ich wusste es dann damals auch nicht: Sind die jetzt so hoch oder so hoch? |
| 774 | **I:** Im Vergleich zu Ihnen selbst, sozusagen? |
| 775 | **IH12:** Hm (zustimmend). |
| 776 | **I:** Mh-h, okay. Das war aber nur in der Psychose? Das ist nicht außerhalb der Psychose auch mal (IH12: Nee, würde ich nicht sagen) vorgekommen? Okay. Und wenn Sie in den Spiegel gucken? Machen Sie das oft? |
| 777 | **IH12:** Ja [LACHT]. |
| 778 | **I:** Ja? Ähm haben Sie sich da schon mal angeschaut und das Gefühl gehabt, das bin gar nicht ich? Oder da sehe ich jemand anders? |
| 779 | **IH12:** In meiner Psychose bin ich ja auch irgendwie geröntgt worden. Und da haben Sie auch gesagt, was sie diese- was Sie heutzutage alles mit dem Messias machen? [LACHT] |
| 780 | **I:** [LACHT] Das haben Sie zu den Leuten gesagt? |
| 781 | **IH12:** Nee, das habe ich mir gedacht [LACHT]. |
| 782 | **I:** Okay. Okay. Inwiefern hängt das mit dem Spiegel zusammen? |
| 783 | **IH12:** Einfach, weil ich mich dort da auch angucke. |
| 784 | **I:** Im Spiegel gucken Sie sich oft an? Oder- oder wo- wo- wo gucken Sie sich an? |
| 785 | **IH12:** Sie haben doch irgendwie gesagt, ob man sich als jemand anders gefühlt hat, wenn man sich im Spiegel anguckt. |
| 786 | **I:** Achso, genau, mh-h. Und da- da haben Sie sich im Spiegel angeguckt und den- den Messias gesehen, sozusagen? |
| 787 | **IH12:** Ja, ich habe schon mich gesehen, aber ja. Ja. (I: Mh-h (zustimmend), okay). Weiß auch nicht. Ist auch schon so lange her. |
| 788 | **I:** Ja, ja. Und gibt es Phasen, in denen Sie immer wieder in den Spiegel schauen müssen, um nachzuprüfen zum Beispiel, ob sich etwas verändert hat oder ob alles noch gleich ist? |
| 789 | **IH12:** Äh ich habe manchmal, wenn mir da irgendwas auffällt, irgendwas, was ich schon immer habe, denke ich plötzlich: Ach Gott, habe ich das schon immer gehabt? Ach Gott, ist es jetzt vielleicht ein Tumor? (I: Mh-h (zustimmend)) Oder- |
| 790 | **I:** Das heißt, Sie untersuchen Ihren Körper? |
| 791 | **IH12:** Ja, es sind eigentlich- plötzlich fällt mir irgendwann mal etwas auf und dann denke ich: Ach Gott, ach Gott, was habe ich denn jetzt? Und dann mache ich tatsächlich dann auch einen Arzttermin aus [LACHT]. |
| 792 | **I:** Ja? |
| 793 | **IH12:** Und dann- aber dann- es war noch nie etwas. |
| 794 | **I:** Okay. Okay. Das hat aber nichts mit dem Spiegel zu tun? |
| 795 | **IH12:** Nee. |
| 796 | **I:** Also Sie gucken jetzt nicht im Spiegel nach Veränderungen? Oder ob sich irgendwie Ihr Gesicht- ob das anders aussieht? Oder ob sich da etwas verändert hat? |
| 797 | **IH12:** Nö, eigentlich nicht. |
| 798 | **I:** Okay. Mh. Hatten Sie schon mal die Erfahrung, oder das Gefühl, dass Ihr Körper oder Teile des Körper taub oder fremd oder weit weg? |
| 799 | **IH12:** Taub habe ich schon gehabt. |
| 800 | **I:** Ja? Können Sie da ein Beispiel- |
| 801 | **IH12:** Das war aber, glaube ich, wirklich so. Da war ich- das war letztes Jahr, das war in [ANONYMISIERT, ORT IN DEUTSCHLAND], in der Pension, da gehe ich dieses Jahr auch wieder hin (I: Mh-h (zustimmend)). Und da bin ich morgens aufgewacht und habe kein Gefühl gehabt, ganzen Arm, Hände. Habe ich dann so geklopft. Und dann bin ich auf die Toilette und dann war da ein Pensionsgast und dann habe ich gesagt: Ach Gott, ich habe gar kein Gefühl mehr in den Händen und kann sie gar nicht mehr richtig bewegen. Und dann ähm hat er gesagt: Ach, gehen Sie einfach wieder ins Bett. Das- das- schlafen Sie mal noch eine Stunde. Und dann habe ich mich wieder ins Bett gelegt und dann kam zum Glück die Pensionswirtin, hat bei mir geklopft: Frau [ANONYMISIERT, NAME DER INTERVIEWTEN PERSON], ich habe eben gehört, mit Ihrer Hand ist irgendwas nicht in Ordnung? (I: Mh-h (zustimmend)) Aber das war wirklich so. (I: Mh-h (zustimmend) Und-) Und dann- und dann war die so lieb zu mir, hat gesagt: Wissen Sie was? Tr- dann, in der Nach- in der- also drei Häuser weiter, ist ein Internist. Gehen Sie doch mal zu dem hin. Man weiß ja nie, was im Urlaub ist, und so. (I: Hm (zustimmend)) Und dann habe ich dem, der dann auch die Reflexe gemacht und das war alles in Ordnung. Und dann war auch schon alles gut. |
| 802 | **I:** Okay. Kam zurück? |
| 803 | **IH12:** Kam zurück. Und dann habe ich dem gesagt, ob das vielleicht daher kam, dass ich im [ANONYMISIERT, SEE IN DEUTSCHLAND] geschwommen bin. Und das war Juni (I: Mh-h (zustimmend)). Also Anfang Juni. Ende Mai war das (I: Mh-h (zustimmend)). Und dann hat er gesagt: Wie lange waren Sie denn drin? Und dann habe ich gesagt: Naja, so eine Viertelstunde bis 20 Minuten. Hat er gesagt: Uh, hat er gesagt, uh Gott. Er war ja gestern auch am [ANONYMISIERT, SEE IN DEUTSCHLAND] und der ist ja eiskalt. Dürfen Sie doch nicht länger als drei Minuten reingehen. |
| 804 | **I:** Okay. Und das war kurz davor? |
| 805 | **IH12:** Das- das war am Tag vorher. |
| 806 | **I:** Am Tag vorher. Mh-h. |
| 807 | **IH12:** Und dann hat er gemeint, da kommt das vielleicht daher (I: Mh-h (zustimmend), okay). Und dann ist auch oft so, dass ich tatsächlich aufwache und die Hände sind eingeschafen (I: Mh-h (zustimmend), dann kribbeln-) oder- oder die Füße sind eingeschlafen. Das kribbelt dann (I: Hm (zustimmend)), deswegen habe ich auch immer mein Handy neben dem Bett liegen. |
| 808 | **I:** Damit Sie? |
| 809 | **IH12:** Damit ich jemand anrufen kann, wenn etwas ist. Wenn ich nicht mehr aufstehen kann. |
| 810 | **I:** Okay. Okay. Kam das schon mal vor, dass Sie dann wirklich die Hand auch nicht mehr bewegen konnten? Oder die Füße? |
| 811 | **IH12:** Naja, wenn die eingeschlafen sind, dann habe ich ja erstmal gar kein Gefühl. |
| 812 | **I:** Ja. Dann muss es zurückkommen genau. |
| 813 | **IH12:** Genau, und dann kann ich sie erstmal nicht richtig bewegen (I: Mh-h (zustimmend)). Und ich wache auch oft nachts auf und kriege keine Luft. |
| 814 | **I:** Okay. |
| 815 | **IH12:** Dass ist so aus dem Schlaf hochschrecke. Aber das kommt wahrscheinlich daher, weil- dass ich so tief schlafe. |
| 816 | **I:** Mh-h. Und dann wachen Sie auf und können erstmal kurz nicht mehr atmen? |
| 817 | **IH12:** Mh-h (zustimmend). |
| 818 | **I:** Sie haben aber dann das Gefühl, Sie können es- also Sie haben Einfluss darauf? Also Sie- Sie können dann- |
| 819 | **IH12:** Ja, ich- ich hole dann Luft [HOLT TIEF LUFT] und dann geht es ja auch wieder (I: Mh-h (zustimmend), okay). Also es nicht so, dass ich da keine ?(Enge)? habe. Das ist- es ist- ich denke, das kommt daher, weil ich so tief schlafe. |
| 820 | **I:** Mh-h. Alles klar. Und hatten Sie schon mal das Gefühl, dass Ihr Körper [GLAS WIRD AUF TISCH ABGESTELLT] mh verschwinden, oder, das klingt jetzt vielleicht komisch, verschwinden, sich auflösen, auseinanderfallen könnte? Dass die Teile des Körpers nicht mehr so richtig zusammengehören? |
| 821 | **IH12:** Habe ich noch nie gehabt. |
| 822 | **I:** Nee. Alles klar. Oder Körperteile werden wie so Dinge im Raum? |
| 823 | **IH12:** [HUSTET] mh-mh (verneinend). |
| 824 | **I:** Nein? |
| 825 | **IH12:** Mh-mh (verneinend). |
| 826 | **I:** Dann gibt es außer diesem, dass manchmal etwas taub wird oder dass es kribbelt- (IH12: [HUSTET]) wollen Sie einen Schluck wasser? |
| 827 | **IH12:** Ich habe mich gerade verschluckt. |
| 828 | **I:** Oh. |
| 829 | **IH12:** [HUSTET] Ja. Geht schon wieder. |
| 830 | **I:** Ja? Ähm andere sondere- sonderbare, rätselhafte Körperempfindungen? Reizen, Drücken, Vibrieren, Schwebegefühl? |
| 831 | **IH12:** Nee [LACHT]. |
| 832 | **I:** Nee? |
| 833 | **IH12:** Nee. |
| 834 | **I:** Gut. Und die eigenen Bewegungen? Wie nehmen Sie die wahr? Fällt Ihnen da irgendwas? |
| 835 | **IH12:** Also in der Psychose bin ich natürlich so gelaufen. Da hatte ich ja dieses furchtbare Medikament. Hatte ich erst [ANONYMISIERT, NAME EINES MEDIKAMENTS] (I: Mh-h (zustimmend)) und dann [ANONYMISIERT, NAME EINES MEDIKAMENTS]. Das [ANONYMISIERT, NAME EINES MEDIKAMENTS] habe ich auch nicht vertragen, da bin ich echt so gelaufen (IH12: ALso mit dem-). Da war ich dann auch- da war ich dann auch echt- da bin ich richtig in die Depression reingerutscht. |
| 836 | **I:** Deshalb, weil Sie sich nicht bewegen konnten, oder? |
| 837 | **IH12:** Ich habe gesagt: Oh Gott, es will keiner, mein Leben lang in der Psychiatrie sein. |
| 838 | **I:** Mh. Also Sie sagen mit den Fingern gespreizt? Und (IH12: also ich bin so gelaufen.) und nach unten guckend (IH12: so bin ich-) ohne die Arme zu bewegen? |
| 839 | **IH12:** Ja (I: Ja.), so bin ich gelaufen. |
| 840 | **I:** Okay. |
| 841 | **IH12:** Mein Vater, der kannte sich dann ja auch in meiner ersten Psychose aus, dass ich [ANONYMISIERT, NAME EINES MEDIKAMENTS] brauche (I: Mh-h (zustimmend)), weil das [ANONYMISIERT, NAME EINES MEDIKAMENTS] ja nicht geklappt hat. Und dann hat er das in [ANONYMISIERT, ORT IN DEUTSCHLAND] den Ärzten, sie sollen [ANONYMISIERT, NAME EINES MEDIKAMENTS] benu- versuchen. Haben sie zu ihm gesagt: Das ist ja auch besser, als sie. [LACHT] Und letztendlich war es dann doch [ANONYMISIERT, NAME EINES MEDIKAMENTS], was ich gebraucht habe. |
| 842 | **I:** War es dann richtig. Okay. Okay. Wieso kannte er sich da aus? |
| 843 | **IH12:** Weil er halt mich kennt und meine erste Psychose schon mitgekriegt hat. |
| 844 | **I:** Achso, das war die zweite (gleichzeitig IH12: das war die dritte) Psychose? Die dritte. Okay. Verstehe. Und das war sozusagen nachdem Sie die Medikamente genommen haen? Oder war es auch schon vor der Psychose so? Hat sich das angekündigt, dass sich der Körper verändert hat? |
| 845 | **IH12:** Nee. |
| 846 | **I:** Nee? |
| 847 | **IH12:** Das war ja- das kam ja durch das [ANONYMISIERT, NAME EINES MEDIKAMENTS] (I: Mh (zustimmend), okay.). Das kam ja durch die Tabletten (I: Alles klar). Davor war es ja nicht. |
| 848 | **I:** Und, also, gab es schon mal Momente, wo Sie das Gefühl hatten, Ihr Körper bewegt sich ohne dass es wirklich so war? Oder ohne, dass es andere Leute gesehen haben? |
| 849 | **IH12:** Nee. Also ich hatte dann so- ich saß dann so da, auch durch die Tabletten, aber das habe ich gemerkt (I: Hm (zustimmend)) und das haben die anderen auch gesehen. |
| 850 | **I:** Das war wirklich? |
| 851 | **IH12:** Das war so. |
| 852 | **I:** Ja. Ja. Und dann hatten Sie vorhin beschrieben- also die nächste Frage ist, ob es Bewegungen gab, die Sie ausführen woll- ähm die Sie ausführen wollten und dann ging das nicht? Da hatten wir vorhin, da beim Bratschenspielen einmal. |
| 853 | **IH12:** Achso, das, ja. |
| 854 | **I:** Diese Situation. Das war nicht innerhalb der Psychose? |
| 855 | **IH12:** Nee. |
| 856 | **I:** Ne? Genau. |
| 857 | **IH12:** Aber das war wirklich nur pf ein Tag lang. |
| 858 | **I:** Einmal? |
| 859 | **IH12:** Einmal. |
| 860 | **I:** Nie wieder, nicht nochmal. Okay. Oder dass Sie Bewegungen ausführen mussten, obwohl Sie das gar nicht wollten? |
| 861 | **IH12:** Naja, das zieht dann hier (I: Mh-h (zustimmend), das-). Wollte ich- wollte ich ja auch nicht unbedingt so dasitzen. |
| 862 | **I:** Das konnten Sie nicht stoppen? |
| 863 | **IH12:** Nee. |
| 864 | **I:** Mh. Wissen wir nicht genau, waren das Medikamente? |
| 865 | **IH12:** Das können gut die Tabletten sein. |
| 866 | **I:** Ja. Das war auch nur nach der zwei- dritten Psychose so? |
| 867 | **IH12:** Nee, das war bei der ersten. |
| 868 | **I:** Bei der ersten. Und da haben Sie [ANONYMISIERT, NAME EINES MEDIKAMENTS] auch genommen? |
| 869 | **IH12:** Ja. |
| 870 | **I:** Ja? Und danach nicht nochmal, nach den anderen Psychosen? Da kam es nicht nochmal? |
| 871 | **IH12:** Ich glaube nicht, dass ich es da nochmal hatte. Das erinnere ich mich jetzt nur an das ersten Mal. |
| 872 | **I:** Okay. Okay. Ähm und wie ist es bei alltäglichen Bewegungen? So etwas wie ähm so Sachen, die automatisch ablaufen? Zähneputzen ähm fahrradfahren, anziehen, Schuhe binden? Gibt es da manchmal Probleme im automatischen Ablauf von solchen- dass man sich irgendwie- dass man viel Konzentration braucht und sich überlegen muss, wie ging das jetzt eigentlich nochmal? Nie? Okay. |
| 873 | **IH12:** Also Fahrrad fahre ich jetzt auch gar nicht mehr. |
| 874 | **I:** Weil Sie Angst davor haben? Ja? Okay. |
| 875 | **IH12:** Ich habe auch keins. Besser gesagt, ich habe noch eins, aber das ist nicht mehr wirklich mein- nicht mehr fahrfähig. |
| 876 | **I:** Okay. Das müsste man nochmal restaurieren? |
| 877 | **IH12:** Ja, so ist es [LACHT]. |
| 878 | **I:** Ja? [LACHT] Gut. Ja. Okay, aber das liegt nicht daran, dass Sie nicht mehr wissen, (gleichzeitg IH12: nee, ich weiß schon-) überlegen müssen- |
| 879 | **IH12:** Nee, nee, (I: wie geht das-) das geht schon (I: Hm (zustimmend)). Ich weiß schon, wie die Sachen gehen. |
| 880 | **I:** Okay. Und haben Sie manchmal das Gefühl, oder gab es so Situationen schon, in denen ähm andere Menschen plötzlich komischerweise dasgleiche gemacht haben, wie Sie? Sich genauso bewegt haben? Sie gedacht haben: Huh, warum bewegen (IH12: [LACHT]) die sich jetzt genauso wie ich? |
| 881 | **IH12:** Mh. Nee, eigentlich nicht. |
| 882 | **I:** Ja, wenn Ihnen das jetzt nicht sofort einfällt, dann war das wahrscheinlich (IH12: [LACHT]) auch nicht so. Weil so etwas merkt man sich meistens, ne? |
| 883 | **IH12:** Ja, nee, das habe ich eigentlich jetzt noch nie erlebt. |
| 884 | **I:** Okay. Wie ist es, wenn Sie andere Menschen- gab es Situationen, wenn Sie andere Menschen berührt haben, dass Sie dann das Gefühl hatten, das ist bedrohlicher Körperkontakt? Oder die Leute gehen- gehen irgendwie in mich rein, wenn sie mich berühren?Oder- |
| 885 | **IH12:** Mh-mh (verneinend). |
| 886 | **I:** Nee? |
| 887 | **IH12:** Eigentlich gar nicht. |
| 888 | **I:** Ähm grundsätzlich, wie ist es mit der eigenen Grenze zwischen Ihnen und anderen Leuten? Ähm haben Sie da manchmal Schwierigkeiten, sich abzugrenzen? |
| 889 | **IH12:** Mh-mh (verneinend). |
| 890 | **I:** Nee? Okay. Also nicht das Gefühl, andere durchdringen Sie? Dringen in Sie ein? |
| 891 | **IH12:** Mh-mh (verneinend). |
| 892 | **I:** Oder ähnliche unangenehme Gefühle (IH12: nee, habe ich nicht), wenn Sie sie angucken (IH12: nee, habe ich nicht), oder so etwas? Nie, okay. Oder wenn Sie in den Spiegel gucken, dass Sie dann das Gefühl haben: Ich weiß nicht mehr so genau, stehe ich eigentlich im Spiegel oder draußen? |
| 893 | **IH12:** Nee, (gleichzeitig I: Dass es so verschmilzt) das habe ich nicht. Ich ziehe mir jetzt nochmal eine Jacke an. |
| 894 | **I:** Ja, wir können auch das Fenster zumachen, wenn Sie wollen. (Geräusche im Raum) |
| 895 | **IH12:** Ist es okay? |
| 896 | **I:** Drücken Sie es ruhig ran. Kein Problem. Ja. Mh-h. Oder das Gefühl, dass Sie so ein bisschen der Welt ausgeliefert sind und das ungute (schleifendes Geräusch) Gefühl, dass die Welt Sie beeinflussen könnte? Dass Sie- das etwas Schreckliches passieren könnte, ohne dass Sie genau sagen können, was? |
| 897 | **IH12:** Mh-mh (verneinend). |
| 898 | **I:** Nee? |
| 899 | **IH12:** Mh-mh (verneinend). |
| 900 | **I:** Okay. Ähm letzter Bereich. |
| 901 | **IH12:** Mh (erleichtert). |
| 902 | **I:** [LACHT] jetzt haben wir auch echt lange gesprochen. (IH12: Mh (zustimmend)). Es geht um Weltanschauung. Um religiöse, philosophische Themen. Ähm gibt es da Zeiten oder gab es Zeiten, wo Sie solche Themen besonders oder solche Fragen besonders intensiv beschäftigt haben? Vielleicht mehr als vorher? Oder? Ja? |
| 903 | **IH12:** Naja, schon [LACHT]. |
| 904 | **I:** Wollen Sie da mal kurz- oder was fällt Ihnen so als erstes ein? |
| 905 | **IH12:** Mit ähm Religion. |
| 906 | **I:** Okay, das war- da gab es einen Zeitpunkt, wo Sie angefangen haben, sich intensiv damit zu beschäftigen? Oder ist das schon immer so? |
| 907 | **IH12:** Also Religion- bin ja auch religiös erzogen worden. |
| 908 | **I:** Mh-h. Beschäftigt Sie schon immer, seit Sie Kind sind? |
| 909 | **IH12:** Ja, schon. Also schon- meine Mutter hat uns immer aus der Bibel vorgelesen. |
| 910 | **I:** Mh-h. Und- und das hat Sie- hat sich intensiviert über die Zeit? Oder gab es da Zeiten, wo sich das intensiviert hat? Oder ähm- |
| 911 | **IH12:** Ja, gut. Meine Psychose war ja auch so ein bisschen religiös. |
| 912 | **I:** Mh-h, mh-h. Also gut, da war- da war es- in der Psychose war es sehr intensiv. Aber das hat nicht nach- das hat nicht nachhaltig auch danach noch diese Beschäftigung mit religiösen Fragen sich intensiviert? |
| 913 | **IH12:** Also- also man beschäftigt sich ja schon damit immer. |
| 914 | **I:** Okay. Ja. Okay. Ich frage nur, ob es da Veränderungen gab über den Zeitverlauf der Intensität, wissen Sie? Dass Sie jetzt nur noch das beschäftigt hat oder ganz viel, bestimmte Fragen Sie ganz viel beschäftigt haben. |
| 915 | **IH12:** Ja, mich hat das halt schon auch immer beschäftigt mit diesen Wunderheilungen (I: Mh-h, mh (zustimmend)) von Jesus (I: Okay.). Ja. |
| 916 | **I:** Und war das dann so, dass Sie da ganz intensiv und viel darüber nachgelesen und nachgedacht haben? |
| 917 | **IH12:** Ja, ich habe mir da sogar so ein Buch gekauft. Das war in der [ANONYMISIERT, ANGABE DER BERUFSAUSBILDUNG] hatte ich so ein Glück, da hatte ich so einen guten Religionslehrer (I: Ja.), der war echt gut (I: Okay.). Und da habe ich gemerkt, da auch sehr inten- habe ich mich halt auch dafür interessiert und da habe ich mir ein Buch gekauft: Die gute Nachricht erklärt (I: Mh-h (zustimmend)) [LACHT]. Und da habe ich auch tatsächlich drin gelesen. |
| 918 | **I:** Mh-h. Vor der Psychose? |
| 919 | **IH12:** Vor der Psychose war das. |
| 920 | **I:** Mh-h. Und da- da stand viel- da stand erklärt- da war erklärt wie die Wunderheilungen- |
| 921 | **IH12:** Ich weiß es nicht mehr, was da genau (I: nee) drin steht. |
| 922 | **I:** Okay. |
| 923 | **IH12:** Das habe ich jetzt vergessen [LACHT]. |
| 924 | **I:** Kein Problem. Mh. Gab es Situationen, wo Sie das Gefühl hatten, äußere Ereignisse oder Menschen ste- stünden in Beziehungen zu Ihnen auf eine gewisse Art und Weise? |
| 925 | **IH12:** Ja, ja, ja, ja (I: Ja?). In meiner Psychose habe ich dann zum Beispiel gedacht, ähm dass meine Geschwister nicht mehr Geschwister sind (I: Mh-h (zustimmend)), also irgendwie schon aber nicht wirklich. |
| 926 | **I:** Sondern? |
| 927 | **IH12:** Ich hatte gedacht, ich bin die Tochter von meinem Onkel, also vom Bruder meiner Mutter (I: Mh-h (zustimmend)). Und die- meine Schwester ist die Tochter von meiner Tante, weil die vom Pfarrer, der jetzt- also es ist total- total identisch, mein Onkel war halt so wie ich. Und meine anderen Geschwister sind wiederum die Kinder meiner Eltern und dass wir irgendwie nur Cousinen sind und so. |
| 928 | **I:** Okay. Also hatten Sie das Gefühl jemand anders zu sein in Wirklichkeit? |
| 929 | **IH12:** Nee. Ich habe schon gewusst, was ich bin. Nur halt nicht, dass wir so Geschwister sind, sondern halt anders miteinander verwandt. |
| 930 | **I:** Ah ja, okay. Okay. Und Sie haben auch gesagt, Sie hatten das Gefühl, Zentrum der Welt, sozusagen, zu sein? Messias zu sein (IH12: [LACHT]) in Ihrer- in Ihrer Psychose? |
| 931 | **IH12:** Ja. |
| 932 | **I:** Aber hatten Sie- haben Sie auch darüber hinaus mal das Gefühl gehabt, alles bezieht sich auf Sie auf eine gewisse Art und Weise? Steht in Verbindung mit Ihren Handlungen oder mit Ihnen generell? |
| 933 | **IH12:** Ja, ich- in [ANONYMISIERT, ORT IN DEUTSCHLAND] weiß ich auch nicht, was das war. Da habe ich plötzlich gedacht, ich würde in einer- im Theaterstück mitmachen. |
| 934 | **I:** Ah die Welt war- war nicht wirklich die Welt, sondern- |
| 935 | **IH12:** Da waren dann die ganzen Patienten. Das war in der Geschlossenen. Und dann bin ich halt- hatte ich gedacht, jetzt muss ich diese Handlungen machen und das so machen. Und dann kam dann die Schwester zu mir: Frau [ANONYMISIERT, NAME DER INTERVIEWTEN PERSON], jetzt lassen Sie die doch mal da in Ruhe sitzen und so. |
| 936 | **I:** Okay. Also das war wie auf einer Bühne? |
| 937 | **IH12:** Ja (I: wie eine Bühne). Ich habe gedacht, das wäre irgendwie ein Buch, das schon geschrieben ist (I: Mh-h (zustimmend)). Und- und ich müsste das dann irgendwie- ein Theaterstück (I: Hm (zustimmend)) aufführen. |
| 938 | **I:** Und sich auf gewisser Art und Weise verhalten, wie man sich eben (IH12: Ja.) im Theaterstück (IH12: Ja.) verhält? Okay. Verstehe. Mh. (IH12: Mh). Das war einmalig aber auch nur? |
| 939 | **IH12:** Das war da in der Klinik. |
| 940 | **I:** Ja. Haben Sie schon mal das Gefühl gehabt, dass nur Sie oder nur das existiert, was Sie gerade erleben? |
| 941 | **IH12:** Nee, das hatte ich noch nicht. |
| 942 | **I:** Nee? Oder außergewöhnliche Kräfte? Eigenschaften, das hatten wir schon. Heilungen? |
| 943 | **IH12:** Heilungen konnte ich. Ich konnte alle Sprachen sprechen. Ich konnte Gedanken lesen (I: Hm (zustimmend)). Oder ich habe gedacht, andere können meine Gedanken lesen. |
| 944 | **I:** Ja? Wie- das hatten Sie vorhin nicht erwähnt. Sie haben nur gesagt, Sie konnten sozusagen telepathisch auch mit den Mitpatienten kommunizieren (IH12: ja), ne? Aber Sie hatten nicht gesagt, dass- dass andere Leute sozusagen Ihre Gedanken lesen könnten. |
| 945 | **IH12:** Ja doch, sonst hätte ich ja mich mit dem gar nicht unterhalten können. |
| 946 | **I:** Ah der hat dann auch sozusagen in Ihren Kopf gucken können? Mh. |
| 947 | **IH12:** Ja, ja. Ich habe mich ja mit dem unterhalten. Dabei saß der auch nur so da [LACHT]. |
| 948 | **I:** [LACHT] Ja. Okay. War das aber dann bedrohlich, oder war- |
| 949 | **IH12:** Nö. |
| 950 | **I:** Nee? Das war okay. Sie haben einfach telepathisch sich unterhalten? Mh-h. |
| 951 | **IH12:** [LACHT] Ja dann- wir haben da so Mandala angemalt. Und da haben wir Schuldkröten gemalt. Und eine Schildkröte sah anders aus (I: Mh-h (zustimmend)). Und dann hat diese Kunsttherapie- oder was weiß ich, Thera- Therapeut: Ach Gott, hat er gesagt, das bin ich, und das andere sind meine Jünger [LACHT]. |
| 952 | **I:** Okay. Okay. |
| 953 | **IH12:** Der wird sich ja auch etwas gedacht haben. |
| 954 | **I:** Ja. Also, ich meine, es ist ja auch eine sehr kreative Geschichte, so eine- so eine- so ein psychotisches Erleben? Oder? Was- was da alles- |
| 955 | **IH12:** Es ist alles logisch (I: Ja.). Und wenn eine Sache gerückt wird, dann ist es sofort auf einer anderen Weise wieder logisch. |
| 956 | **I:** Okay. Ja. Und ist es manchmal davor oder danach oder auch jetzt noch ähm so, dass Sie das Gefühl haben, die Dinge hängen von Ihnen ab? Was in der Welt geschä- geschieht, (gleichzeitig IH12: Achso, nee, das habe ich nicht) hängt von Ihnen ab? |
| 957 | **IH12:** Das habe ich zum Glück nicht. |
| 958 | **I:** Sie sind die Ursache? |
| 959 | **IH12:** Nee. |
| 960 | **I:** Nee? Nicht mehr? |
| 961 | **IH12:** Das habe ich zum Glück nicht, nee. |
| 962 | **I**: Und in der Psychose war es aber schon da, ne? Dass Sie- dass Sie, wie- |
| 963 | **IH12:** Da habe ich ja gepredigt. |
| 964 | **I:** Ja, genau. Äh genau. Okay. Gut. Letzte- vorletzte Frage. Hatten Sie schon mal das Gefühl, dass ähm Sie Zusammenhänge besser verstehen können als (IH12: Ja) andere Leute? Grundsätzlich- |
| 965 | **IH12:** Nee, nicht als andere Leute, aber ich habe plötzlich- in meiner Psychose habe ich gedacht: Oh, ich kann plötzlich so gut denken. Ich kann mir alles so gut merken. Kann alle Sprachen (I: Ja.). Ich habe immer Probleme mit dem Lernen gehabt. |
| 966 | **I:** Ja, ja. Das haben Sie gesagt, ja. |
| 967 | **IH12:** Und dann- und dann haben Sie mir diese Tabletten gegeben und da habe ich echt gedacht, diese Tabletten, die machen mich krank (I: Hm (zustimmend)). Immer, wenn ich diese Tabletten genommen habe, konnte ich plötzlich nicht mehr denken. |
| 968 | **I:** Mh-h. Ja. Klar. Man sich erstmal an die Tabletten gewöhnen. Wie ist es jetzt? Also wie ist das Gefühl jetzt? Mit- |
| 969 | **IH12:** Normal. Ich nehme ja schon so lange Tabletten (I: Okay.). Nee das war ja- das war ja dann tatsächlich- aus der Psy- Psychose rauszukommen ist ja nicht so einfach (I: Ja. Ja, ja.). Das hat vom vierten November bis März gedauert. März habe ich noch falsche Gedanken gehabt. |
| 970 | **I:** Falsche Gedanken. Was meinen Sie mit falsch? Also die- die Gedanken mit Messias (IH12: Ja) und solche Sachen? Okay. Okay. Ja, na dann stelle ich meine letzte Frage. Das ist einfach- wenn Sie die Forschungsfrage angucken- also es geht um Bewegung und Schizophrenie. Und die Frage, hängt das zusammen? Als Expertin (IH12: Oh Gott) für Schizophrenie, für Ihre eigenen Erfahrungen, mit (IH12: Mh-h) der- mit der Psychose. Was würden Sie sagen? Gibt kein richtig, kein falsch (IH12: [LACHT]), nur die eigene- nur die eigene Erfahrung. |
| 971 | **IH12:** Also. Ich glaube Schizophrenie- ähm ich glaube, dass man sich dann besser bewegen kann (I: Okay?), wenn man krank ist. |
| 972 | **I:** Okay? Und wollen Sie kurz erklären? |
| 973 | **IH12:** Ich weiß auch nicht. Ich- ich- also in- in der Psychose habe ich echt gedacht, ich kann alles total super und- und auch mich total super bewegen. Ich habe dann da- ich war ja da- habe ja da eine Freundin besucht und wir sind abends in die Disko gegangen. Ich habe da in der Psychose total- mich total bewegt und getanzt und gemacht. |
| 974 | **I:** Okay. Also es war ein positives Erlebnis. Das war- das war, bevor Sie zum zweiten Mal, zum dritten Mal? |
| 975 | **IH12:** Das erste Mal. |
| 976 | **I:** Das erste Mal, okay (IH12: war das). Weil Sie gesagt hatten, das kam so plötzlich, die- die Psychose. |
| 977 | **IH12:** Ja (I: Ja?), naja, das Wochenende, da war ich eigentlich auch schon krank. |
| 978 | **I:** Okay. Okay. Und da war alles intensiver? |
| 979 | **IH12:** Intensiver (I: Ja?) und- und ich habe die Welt viel intensiver erlebt. Und habe mh und habe auch gedacht, ich kann viel besser denken und mich auch super gut bewegen einfach. |
| 980 | **I:** Ja. Okay. Alles klar (IH12: [LACHT]). Gut, danke schön. Gibt es noch was Ihnen- was Sie noch erwähnen wollen? Was wir vergessen haben? Was wir noch nicht besprochen haben? |
| 981 | **IH12:** Eigentlich haben wir ja viel besprochen [LACHT]. |
| 982 | **I:** Richtig viel, genau. Sehr, sehr viel. Auf jeden Fall. Okay. Schön. Dann würde ich mit Ihnen jetzt- dann mache ich das hier mal aus. |

SF03a_EASE_anonymized

| 1 | Interview SF03a |
| --- | --- |
| 2 | **I:** So, Sie haben zugestimmt, dass wir Sie aufnehmen dürfen? |
| 3 | **SF03a:** Ja. |
| 4 | **I:** Ja? Okay. Gut. Was wir jetzt machen, ist ein phänomeologisches Interview (SF03a: Okay.). Das heißt, ein Interview, in dem es um Ihre Erfa- Erfahrungen, Ihre ganz persönliche Erfahrung geht. |
| 5 | **SF03a:** (unverständlich) |
| 6 | **I:** (unverständlich) (SF03a: [LACHT]) Genau. Sie- Sie wissen Bescheid, ja genau. Ähm das heißt, wenn wir das beziehen auf die- auf die ähm Schizophrenie oder auf die Erkrankung, dass es nicht so sehr darum geht, was die- was die Symptome sind, die Sie schon kennen, wo Sie die Namen kennen (SF03a: Ja.), sondern Ihre ganz persönliche Erfahrung (SF03a: Okay.). Sprich: Ich kann Sie [/Ihnen/] fragen stellen, da denken Sie, das sind komische Fragen. Was (SF03a: Hm (zustimmend)) meinen Sie damit? Und wenn ähm Sie das noch nie erlebt haben, es kein- kein Glöckchenklingeln irgendwo im Kopf, dann (SF03a: Ja.) ähm sagen Sie einfach: Nö, war bei mir nicht so. Und wir machen weiter im Text (SF03a: Ja.). Und wenn Sie sich an etwas erinnern können, was so ähnlich war bei Ihnen, dann können Sie es gerne in eigenen Worten beschreiben. Ich würde Sie dann nach Beispielen fragen (SF03a: Ja.). Und so weiter, ja? |
| 7 | **SF03a:** Okay. |
| 8 | **I:** Genau. Und das ganze dauert so lange, wie Sie wollen (SF03a: Okay.). Wenn Sie keine Lust mehr haben, wenn Sie eine Pause brauchen, wenn Sie auf Toilette müssen, wenn wir an einem anderen Termin weitermachen sollen: Alles gar kein Problem, ja? |
| 9 | **SF03a:** Okay. |
| 10 | **I:** Und Sie erzählen, was Sie erzählen wollen. Sie müssen nichts erzählen (SF03a: Ja.), was Sie nicht erzählen möchten. |
| 11 | **SF03a:** Gut. |
| 12 | **I:** Okay. Dann würde ich Sie gerne als allererstes fragen: Wenn ich sage: Wer sind Sie? Was würden Sie sagen? |
| 13 | **SF03a:** Meinen Namen? Weil der ja als Bezeichnung für eine Person verwendet wird? Aber das ist natürlich eine sehr schwierige und philosophische Frage, die Frage nach der Identität. Ähm. Ich würde sagen, ich bin ein interessierter Mensch. Interessiert an einigen Dingen und ja ich finde auch, dass Freunde und Familie mich definieren, sozusagen. Und mh die Gegenwart ist ja auch irgendwo gezeitigt von der Vergangenheit, von den Erfahrungen. Also würde ich sagen, ich bin [ANONYMISIERT, NAME DER INTERVIEWTEN PERSON], ein interessierter Mensch, der schon diverse Sachen erlebt hat. Der in einem bestimmten sozialen Rahmen lebt. |
| 14 | **I:** Okay. Und Sie sagen: Familie definiert Sie auf eine gewisse Art und Weise. Wie sind Sie aufgewachsen? |
| 15 | **SF03a:** Also ähm. In meiner frühen Kindheit wurde ähm in meiner frühen Kindheit bin ich beruflich äh bedingt äh also beruflich durch meinen Vater bedingt sehr oft umgezogen. Aber meine Mutter hat mich dann bemüht, dass ich zumindest zu einem guten Freund noch den Kontakt gehalten habe, auch wenn er dann in [ANONYMISIERT, ORT IN DEUTSCHLAND] gewohnt hat und ich in [ANONYMISIERT, ORT IN DEUTSCHLAND] (I: Hm (zustimmend)) und ahm oder sonst irgendwo. Also ich bin sehr oft umgezogen und dann zu meiner Einschulung ziemlich genau haben meine Eltern sich getrennt (I: Hm (zustimmend)). Ich habe aber nicht so viel Erinnerungen an die Zeit vor meiner Einschulung. Vor allem habe ich irgendwo gar keine Erinnerung an meinen Vater vor der Einschulung. Also als Familie, meine beiden leiblichen Eltern, kann ich mich gar nicht so richtig erinnern. Dann bin ich eben bei meiner Mutter aufgewachsen, in [ANONYMISIERT, ORT IN DEUTSCHLAND]. Ähm und da sind wir dann aber auch im Ort geblieben. Von meiner Einschulung bis jetzt. Meine Mutter wohnt noch da. Ich bin da noch öfter am Wochenende. Und dann hatte ich ein bisschen Schwierigkeiten in der Schule, weil ich mich sehr auf die Schule gefreut habe. Ich habe mir schon selbst Buchstaben beigebracht, als ich noch recht klein war. Und habe dann gedacht: Jetzt lernt man. Und dann wurde mir irgendwann gesagt: Lesen, Schreiben, das lernt man aber in der Schule. Das darfst du jetzt noch gar nicht können (I: Mh-h (zustimmend)). Ich ?(erfuhr)? von meiner Mutter aber von so anderen Eltern von gleichaltrigen Kindern, die das nicht so gut fanden, dass ich da schon so weit war. Und dann habe ich mich sehr auf die Schule gefreut. Hatte sehr hohe Erwartungshaltung. Wurde dann sehr enttäuscht, weil ich es sehr langweilig fand. Weil ich es sehr äh blöd fand, dass man das da so sehr viel ausmalt und sauber ausschneiden muss (I: Mh-h (zustimmend)). Das habe ich in dem Kindergarten nie gemacht (I: Mh-h (zustimmend)). Ich habe mir lieber irgendwas mit Lego gebaut oder habe mir irgendwelche ähm Bücher angeguckt. Und ausmalen und ausschneiden, sauber und dann sauber ins Heft kleben war nicht so meine Stärken (I: Mh-h (zustimmend)). Deswegen bin ich dann auch nach der Grundschule auf die Realschule gekommen. War man, also wenn ich es salopp sagt, hat man aus den inhaltlichen- aus der inhaltlichen Gymnasialreife und der- den formalen, ordentlichkeitsbezogen Hauptschulniveau den Mittelwert genommen und hat mich dann auf die Realschule geschickt (I: Okay.). Ähm da war ich dann relativ unterfordert und wollte auch gerne aufs Gynmasium. Ich habe das überhaupt nicht eingesehen, die langweiligen Hausaufgaben zu machen, weil die nur Freizeit rauben und ich sowieso gute Noten schreiben konnte (I: Mh-h (zustimmend)) ohne Hausaufgaben zu machen (I: Mh-h (zustimmend)). Und das fanden die Lehrer nicht sehr gut. Die haben äh mir dann lauter Klassenbucheinträge gese- gegeben. Wollten mich schon alleine wegen Hausaufgaben-Nichtmachen von der Schule verweisen. Das stand auch zur Debatte, ob ich auf eine Sonderschule kommen soll. Ich hatte dann eine intensive Auseinandersetzung mit einer Schulpsychologen, die mir erzählt hat, dass sie selbst auch auf der Realschule war und dann danach ihr Abitur gemacht hat. Und dass das doch nicht so schlimm sei. Und ich soll mich nicht so anstellen. Und dann bin ich aber zu mehreren Schulpsychologen, den zuständigen Ämtern in [ANONYMISIERT, ORT IN DEUTSCHLAND] gegangen. Habe dann mal eine Testung machen lassen. So eine Mischung aus Intelligenz und Bildung. Auch so Sachen wie Wortschatz, oder so. Und da kam dann raus, dass ich auf jeden Fall aufs Gymnasium soll. Und dann bin ich nach der achten Klasse Realschule in die achte Klasse gymnasial- Gymnasium. Habe die sozusagen wiederholt auf einer höheren Schulform. Ähm. Allerdings da gab es da das Problem, dass ich in der Realschule keine Fremdsprache gewählt habe. Französi- nicht Französisch gewählt habe, sondern Technik. Und mh dann musste ich innerhalb einmal Sommerferien eben drei Jahre Latein beibringen. Weil die Klasse, in die ich gekommen wurde [/bin/], wurde nach dem Biberacher Modell unterrichtet. Das heißt, man hat Latein als erste Fremdsprache (I: Hm (zustimmend)). Damals gab es noch kein Englisch auf einer Grundschule (I: Hm (zustimmend)). Also Latein in der fünften ganz intensiv und Englisch nur so ein bisschen. Und dann musste ich da irgendwie Latein nachholen. Da habe ich dann ganz viel mit meiner Mutter Latein gelernt. Das fand meine Schwester bisschen [LACHT] nicht so gut. Die hat sich dann vernachlässigt gefühlt, dass meine Mutter so viel Latein mit mir gelernt. Aber das ging dann irgendwie mit dem Latein. Mache ich bis heute noch sehr gerne. Ich schreibe auch manchmal lateinische Postkarten an einen Freund oder spiele lateinisch mit ihm Scrabble. Ähm ah und nach der achten Klasse Gymnasium hat man dann gemerkt, ich war dann ein paar Wochen in der neunten Klasse und das war irgendwie- hat mir nicht so gefallen. Das war irgendwie zu einfach. Die Lehrer haben mir nicht gepasst. Dann hat man gesagt: Ja, dann soll ich halt überspringen. In die Zehnte. Dann gab es das Problem: Die einzige zehnte Klasse mit Latein hatte da schon in mein- Englisch meine Mutter. Und die darf mich nicht unterrichten. Die war Lehrerin an derselben Schule (I: Mh-h (zustimmend)) und dann hat man gesagt: Ja, dann überspringe ich halt zwei Klassen. Und dann bin ich quasi nach der achten Klasse in die Oberstufe gegangen. In die elfte Klasse. Und das war das erste Jahr mit G8, also das war der berüchtigte Doppeljahrgang (I: kurz, ja genau, hm.). Das heißt, das war eh so eine Durchmischung. Da habe ich- bin ich auch nicht so weiter aufgefallen. Nicht so sehr. Und dann ja es war schon komisch dann irgendwie mit fünfzehn in der Oberstufe zu sein, aber das hat dann auch eigentlich vom Sozialen her ganz gut geklappt. Achso, ich habe noch vergessen, was natürlich sehr wichtig ist: Als ich dann in der achten Klasse auf dem Gymnasium war, ging es mir besser als auf der Realschule. Viel besser auch von den Mitschülern her. Das war eine sehr strebsame und äh soziale Klasse. Aber da hatte ich die erste Psychiatrieerfahrung auch gemacht und was (I: Mh-h (zustimmend)) ich damals auch gesagt habe, ist: Davor konnte ich alles darauf schieben, dass ich unterfordert bin. Und wenn ich jetzt nicht glücklich bin, jetzt auf dem Gymnasium, dann muss es an mir selbst liegen. Das war so ein bisschen die- meine Begründung warum ich dann so etwas entwickelt habe. Dann ware ich da zum ersten mal in der Psychiatrie in [ANONYMISIERT, ORT IN DEUTSCHLAND] (I: Mh-h (zustimmend)). Und damals war die Diag- achso, Diagnosen darf ich nicht sagen, weil es ja phänomenologisch ist, ähm- |
| 16 | **I:** Sie dürfen gerne die Diagnose sagen. |
| 17 | **SF03a:** Das war Sozialphobie. |
| 18 | **I:** Sozialphobie, okay. |
| 19 | **SF03a:** Die habe ich aber seitdem nicht mehr bekommen, also. |
| 20 | **I:** Was können Sie- können Sie erklären, als Sie da ins (SF03a: Ja, also-) also, was so die Auslöser (SF03a: Ja, genau) waren? Wie Sie sich gefühlt haben? |
| 21 | **SF03a:** Also. Es- man könnte es auch- es war- also Soziaphobie klingt jetzt nach Angststörung. Aber es war mehr so etwas Depressives, was vielleicht in der Pubertät gar nicht so ungewöhnlich ist [LACHT]. So: Die anderen mögen mich nicht wirklich. Ich habe keine Freunde. Und so. Das stimmte zwar nicht, aber es war halt schwierig auch für mich in die Klasse zu kommen, wo sich viele seit dem Kindergarten kannten (I: Mh-h (zustimmend)) und ich bin dann einfach so als Exot, der sich selbst Latein beigebracht hat von der Realschule gekommen (I: Mh-h (zustimmend)). Das war nicht ganz so einfach. Und ich habe aber eigentlich ganz gut sozialen Anschluss gefunden, aber irgendwie hat es sich noch nicht so echt angefühlt. Und- |
| 22 | **I:** Also Sie hatten jetzt mal so von außen betrachtet: Wenn- wenn (SF03a: Ja.) damals jemand gesehen hätte, in der Klasse, der Lehrer oder die Lehrerin (SF03a: Ja.), dann hatten Sie- dann hatten Sie F- Freunde (SF03a: Ja.) und Kontakte (SF03a: Ja.) und Sie selbst hatten nicht das Gefühl, dass es so ist? |
| 23 | **SF03a:** Ja. Also. Die waren mir wichtig. Aber ich hatte- also ich hatte schon das Gefühl, dass ich Freundschaft für sie sozusagen empfinde, aber ich war mir halt nicht sicher, ob sie mich auch als Freund sehen (I: Mh-h (zustimmend)). Und heute weiß ich, dass es so ist. Und heute weiß ich auch, dass ich dem einen Freund, dem [ANONYMISIERT, NAME EINES FREUNDES DER INTERVIEWTEN PERSON], mit dem ich immernoch Kontakt habe, der studiert jetzt Physik hier in [ANONYMISIERT, ORT IN DEUTSCHLAND], dass ich ihm sogar ganz gut getan habe. Dass er, dav- bevor er mich getroffen hat, nicht so richtig aus sich herausgekommen ist (I: Mh-h (zustimmend)) und eher so verschüchtert war. Und ich ihm dann so ein bisschen, bisschen geholfen habe, aus sich herauszukommen (I: Mh-h (zustimmend)). Und, also, ich war, wie- wie hat er es gesagt? Der Psychiater in der [ANONYMISIERT, ORT IN DEUTSCHLAND], nee der Psychologe, das war der führende Psychotherapeut. Da gab es so eine duale Leitung (I: Mh-h (zustimmend)) mit Psychotherapie- peut und Chefärztin (I: Mh-h (zustimmend)) und ähm der hat gemeint, ich bin eigentlich sozial sehr attraktiv, also nicht äußerlich, sondern ich- mit mir wollen Leute eigentlich etwas machen. Insofern ist es eigentlich unverständlich, dass ich [LACHT] Sozialphobie entwickelt habe (I: Mh-h (zustimmend)). Aber genau, also ich würde sagen, das war halt so etwas Depressives und dem wurde da dann irgendwie der Stempel Sozialphobie aufgedrückt. |
| 24 | **I:** Hm. Hm. Weil sich Ihre Gedanken auf andere Leute (SF03a: Ja, genau), die Ängste, dass andere Sie nicht mögen und so? |
| 25 | **SF03a:** Ja, genau, und eben auch Sozialphobie und keine soziale Angst (I: Mh-h (zustimmend)), weil ja es sich auf Leute bezogen hat, die ich eher näher kenne (I: Mh-h (zustimmend)). Soziale Angst hatte ich dann später auch mal als Diagnose (I: Mh-h (zustimmend)), da, ja. Gut. Dann- jetzt würde ich wieder zurück zur Oberstufe bringen- springen. Dann [QUITSCHENDES GERÄUSCH] habe ich da eben- da ging es mir dann auch nicht immer so gut, aber ich fand das ganz angenehm unter so bisschen reiferen jungen Menschen zu sein. Habe mich da auch gut zurecht gefunden so vom Sozialen her, obwohl die mich nicht kannten. Ich war natürlich auch so ein bisschen so ein bunter Hund. So ein Phänomen, weil ich dann zwei Klassen übersprungen hatte. Und das haben mich auch so Fünftklässler, so kleine, dann gefragt: Ja, bist du der, von (I: [LACHT]) dem ich gehört habe (I: [LACHT]). Und dann habe ich gesagt: Ja, der bin ich. Und ähm ja. Und genau, dann war ich da in der Oberstufe, habe Abitur gemacht. Ich habe mir nicht wirklich Mühe gegeben. Hatte einen eins neuner Schnitt, weil ich mir auch gesagt habe: Ich strenge mich nicht wirklich an. Ich will eben [ANONYMISIERT, STUDIENFACH] studieren, das will kein Mensch. Da gibt es keinen NC. Können Sie als Psychologin nicht so teilen (I: Kann ich nicht, ja), diesen Ansatz. |
| 26 | **I:** Ich brauchte dann auch einen NC, ja. |
| 27 | **SF03a:** Ja. Also ich habe da nicht wirklich etwas gelernt. In Chemie hat es nicht so gut funktioniert, weil man da viel auswendig lernen muss. In Phsyik hat es besser funktioniert, weil alles eher logisch ist (I: Mh-h (zustimmend)) und man sich die Sachen aus anderen herleiten kann, ja. Und dann habe ich ja da eben Abitur gemacht und dann wollte ich in [ANONYMISIERT, ORT IN DEUTSCHLAND] studieren. Mit 17 damals noch, ich war gerade 17 geworden. Äh mit 16 habe ich Abitur geschrieben. 17 war ich, als ich das Zeugnis bekommen habe. Und dann bin ich da nach [ANONYMISIERT, ORT IN DEUTSCHLAND] gezogen in diese riesige Großstadt aus der [ANONYMISIERT, ANGABE DER EINWOHNERZAHL] Kleinstadt [ANONYMISIERT, ORT IN DEUTSCHLAND] (I: Hm (zustimmend)). Und habe da [ANONYMISIERT, STUDIENFÄCHER] studiert. Habe noch mein Graecum angefangen und am Anfang lief das auch sehr gut. Aber ich hatte einen sehr großen Erwartungsdruck. Also das, was sich aus einer Erwartungshaltung, würde ich sagen, ergibt, weil ich bin ja- wurde dann ja auch so mit so Titeln belegt wie Genie oder Wunderkind oder so etwas (I: Hm (zustimmend)) und dann muss man ja auch gut sein. Und das war ich eigentlich auch. Aber irgendwie hat mich die Situation alleine in der Großstadt, in einer WG mit Leuten, die kein Deutsch sprechen, die zwar Französisch und Englisch sprechen, das war schon nicht das Problem aber irgendwie hat mich die Situation sehr überfordert (I: Hm (zustimmend)). Und es gab da wenig Kontrollinstanzen. Wenig Leute, die gefragt haben, was eigentlich mit mir los ist. Dann habe ich mich- hatte ich erst so depressive Sachen. Oder halt, dass ich zum Beispiel zum- zum- zur Immatrikulation einen Rucksack geschenkt bekommen habe. Den habe ich dann verloren. Und dann habe ich gesagt: Ja, jetzt kann ich das Studium auch gleich abbrechen, wenn ich diesen Rucksack verloren habe. Also so ein sehr, sehr strenges Umgehen mit sich selbst und sehr nachtragend mir selbst gegenüber (I: Hm (zustimmend)) war ich da. Und das würde ich auch in die depressive Ecke schieben. War aber eher auf mich bezogen, also nicht so sehr auf andere. Und dann habe ich mich immer mehr zurückgezogen. Und dann hat sich so- so ein Verfolgungswahn, so Verfolgungsideen haben sich dann entwickelt. Und da es lange Zeit unbehandelt und unbemerkt und so weiter war, ist es dann auch ziemlich schlimm geworden. Bis dann irgendwie, nachdem ich ziemlich erratisch und ziemlich verschwörungstheoretisch mit meiner Mutter mal telefoniert habe, sie gesagt hat: So, das geht nicht. Ich fahre jetzt nach [ANONYMISIERT, ORT IN DEUTSCHLAND]. Ich hole dich ab. Ich bringe dich nach [ANONYMISIERT, ORT IN DEUTSCHLAND]. Das hat sie da nicht gesagt, aber das hat sie dann gemacht (I: Hm (zustimmend)). Und dann war ich da erstmal aber- das war eine sehr schwierige Phase mit 17. Da- das war der längste Aufenthalt oder die längsten Aufenthalte in Psychiatrien, die ich bis da hatte. Ich glaube, das war über ein halbes Jahr, war ich immer wieder in der Psychiatrie. Am Anfang war auch das Problem, dass ich nicht einsichtig war. Sogar so sehr nicht einsichtig, dass ich überhaupt die Existenz von psychischen Krankheiten infrage gestellt habe (I: Ja.). Ich habe dann gesagt, ähm es gibt keine psychisch kranken Menschen, sondern nur Menschen mit geringer Willensstärke. War aber selber in einer akuten Psychose. Also habe, wenn die Pizza zu salzig geschmeckt hat, gedacht, dass mich jemand vergiften will und ja, das war sehr schwierig. Irgendwann habe ich dann gedacht, dass mich irgendein Auto, das vorbei gefahren ist, dass da gleich jemand aussteigt, um mich zu entführen. Und da hat man die Polizei angerufen. Ähm und das war dann der Punkt, wo ich einsichtig wurde und gedacht habe: Ja, ich muss mir jetzt helfen lassen. Und dann habe ich denen auch nicht mehr so von der Psychiatriekritik von Faucoult vorgetragen (I: [LACHT]), wenn ich mit Psychiatern geredet habe. Sondern, habe mich dann bisschen darauf eingelassen. Dann war ich da sehr lange in der Kinder- und Jugendpsychiatrie damals noch. Hatte dann in der Kinder- und Jugendpsychiatrie meinen 18. Geburtstag, was dazu geführt hat, dass ich dann mit 18 noch in der Kinder- und Jugendpsychiatrie (I: Hm (zustimmend)) war, was auch nicht so oft vorkommt. Ja. Und als jemand, der schon angefangen hat, zu studieren, das kann sowieso überhaupt (I: Ja.) nicht in der Kinder- und Jugendpsychiatrie (I: Ja.). Aber das war ganz- war eigentlich- eigentlich war es auch teilweise eine schöne Zeit, als es mir dann besser ging. Weil da ist man halt sehr intensiv auf engen Raum mit jungen Leuten zusammen und- in der Sch- ich durfte dann auch in die Klinikschule gehen, obwohl ich schon Abitur hatte und habe dann mit dem Englischlehrer irgendwas über Shakespeare gemacht und so. Und habe irgendwie Lateinvokabeln gelernt oder so etwas (I: Hm (zustimmend)). Das war eigentlich ganz gut da dann als es mir ein bisschen besser ging. Aber es hat sehr lange gedauert. Was auch sehr schlecht war, war der Schlaf. Ich konnte nächtelang nicht schlafen (I: Okay.). Äh- ich- ich bin immer noch bei der ersten Frage. |
| 28 | **I:** Alles klar, gar kein problem. |
| 29 | **SF03a:** Aber ich bin jetzt ja schon so ungefähr bei meinem 18. Geburtstag (SF03a und I: [LACHEN]), also bin ich jetzt an- und dann wurde ich eben da entlassen und als es mir wieder besser ging und damals hatte ich auch wirklich Verfolgungswahn. Damals gab es dann auch die Diagnose soziale Angst (I: Mh-h (zustimmend)) und paranoide Schizophrenie. |
| 30 | **I:** Mh-h, okay, zusammen, sozussagen? |
| 31 | **SF03a:** Zusammen. |
| 32 | **I:** Ja. Ähm. Dieses- dieses- diese Ereignisse, dass Sie schon mal eine Diagnose ganz früh bekommen haben und dann ähm mit dem 17. Lebensjahr die schizophrene Diagnose (SF03a: Ja.). Könnten Sie das nochmal zeitlich ein bisschen einordnen? Wie alt waren Sie ungefähr bei der ersten- bei dem ersten (SF03a: 14) Gefühl- 14 waren Sie da? Da hatten Sie dieses Gefühl, andere Leute könnten Sie nicht als Ihre Freunde sehen, zum (SF03a: Ja.) Beispiel, mh-h. Oder Angst, nicht richtig anz- angenommen zu werden (SF03a: Ja.), oder so? |
| 33 | **SF03a:** Genau. |
| 34 | **I:** Könnte man sagen, dass Sie sich einfach auch ein bisschen anders gefühlt haben? Als wären Sie nicht so richtig Teil der Welt? |
| 35 | **SF03a:** Ja. |
| 36 | **I:** Teil? |
| 37 | **SF03a:** Also vielleicht [crosstalk] als Teil der sozialen Gruppe oder so (I: Mh-h (zustimmend)). Ähm und nicht Teil der Gesellschaft. So, gleich so metaphysisch Teil- nicht Teil der Welt. Würde ich es da noch nicht sagen (I: nee, okay) an der Stelle. |
| 38 | **I:** Und nicht Teil der Gesellschaft- wenn Sie sagen: Nicht Teil der Gesellschaft (SF03a: Ja.). Was meinen Sie damit? |
| 39 | **SF03a:** Naja, ich habe mich dann irgendwie auch mit diesem Hikikomori-Trend in Japan beschäftigt, kennen Sie das? |
| 40 | **I:** Das müssen Sie mir erklären. |
| 41 | **SF03a:** Achso. Ja, die kriegen oft auch die Diagnose Sozialphobie. Also Hikikomori ist Japanisch und das ist jemand halt so jemand, der nicht arbeitet, keine Ausbildung hat und einfach nicht sich nicht mehr aus dem Zimmer traut. |
| 42 | **I:** Mh-h, ah, okay, mh-h. |
| 43 | **SF03a:** Und das ist irgendwie teilweise fast schon ein Massenphänomen in Japan so ein bisschen (I: Ja.) und deswegen gibt es da einen japanischen Begriff dafür. Und ich hatte mich bisschen damit beschäftigt und dann habe ich mir irgendwie gedacht: Ja so werde ich bestimmt auch. Was irgendwie ziemlich seltsam ist, mit- weil ich ja schon wusste, dass ich nicht ganz blöd bin. Ja. Und ja, also auch das Gefühl, wertlos zu sein. Das war nicht nur auf andere bezogen, sondern auch also die anderen sehen mich nicht als Freund und das- sie sind auch richtig in der Annahme, weil (I: Hm (zustimmend)) ich es nicht wert bin, ihr Freund (I: Okay.) zu sein. |
| 44 | **I:** Okay. Mh-h. Also kann man sagen- könnte man sagen, das war wie ein bisschen wie so eine innere Leere? Weshalb waren Sie nicht wertlos? Äh weshalb waren Sie wertlos? Entschuldigung. |
| 45 | **SF03a:** Dafür habe ich nicht wirklich eine Begründung. |
| 46 | **I:** Für dieses Gefühl? |
| 47 | **SF03a:** Nein (I: Nein), das war mehr einfach da. Ich weiß nicht, durch was das gekommen ist (I: Hm (zustimmend)) oder wie ich das gerechtfertigt habe (I: Ja), dass ich wer- dass ich wertlos bin, also kann ich- kann ich- |
| 48 | **I:** War einfach so ein Gefühl. |
| 49 | **SF03a:** Ja. |
| 50 | **I:** War einfach da. |
| 51 | **SF03a:** Mh-h. |
| 52 | **I:** Okay. Und ist das- ist das plötzlich aufgetreten oder kam das einfach so langsam? Oder können Sie sich erinnern, ob das schon immer da war? |
| 53 | **SF03a:** Das war schon immer so ein bisschen da. Weil ich mir irgendwie schwergeta- ich glaube, ich habe einfach auch sehr, sehr viel Erwartungen an Freundschaft gehabt, die da auch teilweise bestätigt wurden. Also ich hatte ja in der frühen Kindheit von Freunden aus [ANONYMISIERT, ORT IN DEUTSCHLAND] erzählt (I: Hm (zustimmend)), die in der Klasse, zu der ich dann auch Kontakt hatte. Ich habe mir halt immer so eine enge, gute Freundschaft gewünscht und hatte die dann auch und war dann irgendwie dann unzufrieden oder so, oder habe gedacht, ich mache da irgendwas falsch, und ja. Also so ein bisschen war das schon immer da. |
| 54 | **I:** Mh. Okay. Okay. Und hatten Sie in Bezug auf dieses Gefühl auch manchmal das Gefühl sich selbst irgendwie fremd zu sein? Nicht so richtig Sie selbst zu sein? |
| 55 | **SF03a:** Würde ich nicht sagen, weil ich doch recht stur war. Was man auch merkt daran, dass ich dann in der Schule nie Hausaufgaben [LACHT] gemacht habe, zum Beispiel (I: Ja.), also. Ich nicht ich selbst zu sein, hatte ich zu dem Zeitpunkt nicht das Gefühl. |
| 56 | **I:** Okay. Gab es das irgendwann schon mal? |
| 57 | **SF03a:** Ja. |
| 58 | **I:** Ja? |
| 59 | **SF03a:** Definitiv. Also auch letzten Freitag [LACHT] als ich- ich habe jetzt durch das Duloxetin kann man sagen, oder vielleicht [AUTO HUPT] einfach durch das Überwindenhaben- Überwundenhaben der depressiven Phase so ein bisschen eine Hypomanie entwickelt (I: Mh-h (zustimmend)) gehabt. Und die war dann- die hat dann- die war ha- war aber nur sehr schwach ausgeprägt. Also Herr [ANONYMISIERT, NAME EINES ARZTES] hat gesagt: So drüber war ich nur Mitte letzter Woche bis (I: Hm (zustimmend)) Freitag (I: Hm (zustimmend)). Und die hat dann am Freitag aber geendet, die Hypomane Phase, und dann gab es so bisschen diese postmanische Depression wieder. Und die habe ich dann aber auch überwunden, so ein bisschen. Ich habe mir dann etwas Gutes getan. Und dann ging es mir wieder besser. Und als es mir dann wieder besser ging nach der kl- kurzen Depression, nach der hypomanen Phase, hatte ich kurz das Gefühl einfach, schon bevor ich in den Spiegel geschaut habe, wer bin ich eigentlich? Weil wer steckt da in meinem Körper so? (I: Hm (zustimmend)) Oder da hatte ich so, so ganz kartesisch gedacht, irgendwie, Körper und Geist, das ist jetzt total getrennt und das war sehr seltsames Körpergefühl. Und dann, als ich auch in Spiegel geschaut habe, kam ich mir sehr fremd vor. Das hat sich dann aber auch wieder relativ schnell, nachdem ich nochmal geschlafen hatte, das war irgendwie nachts, und dann habe ich nochmal geschlafen und dann hat sich das am nächsten Morgen auch wieder gelegt gehabt. |
| 60 | **I:** Okay. War das die einzige Situation, in der Sie dieses Gefühl erlebt haben oder gab es schon andere Situationen, wo Sie das auch schon mal hatten? |
| 61 | **SF03a:** In dieser Ausgeprägtheit hatte ich das sonst nicht. Aber so ein bisschen hatte ich es dann schon, wenn ich eben- wenn ich eben so psychotisch war so im- im Wahn, so ein bisschen (I: Hm (zustimmend)). Ähm. Dann so dieses Gefühl, nicht ich selbst zu sein (I: Ja. ). Das war dann schon manchmal auch da, dass ich mir auch so auf die Hand geguckt habe und gedacht, was ist das für eine Hand? So. |
| 62 | **I:** Alles klar. Hm. Und wie sieht es mit Gedanken und Gefühlen oder Handlungen aus in solchen verschiedenen Situationen? Sind die immer Ihre eigenen? Oder kann es auch mal sein, dass Sie das Gefühl haben, das sind gar nicht meine eigenen Gedanken? |
| 63 | **SF03a:** Also Gedanken, Handlungen. Nicht- nicht, also da habe ich nicht das Gefühl, dass die irgendwie fremd sind. Aber was ich teilweise hatte, was stark in die Richtung geht, ist beim Bedienen von elektronischen Geräten. Laptop, Handy. Hatte ich, wenn ich- wenn es mir schlecht ging, wenn ich psychotische Symptome hatte, hatte ich manchmal den Eindruck, dass da irgendwas pa- dass ich irgendwas am Handy gemacht habe oder am Laptop. Und dann gedacht habe, da passie- die Maus bewegt sich gerade und ich habe das gar nicht selbst gemacht (I: Mh-h (zustimmend)). Oder am Handy, da öffnet sich eine App und ich habe da gar nicht drauf gedrückt (I: Okay.), das hatte ich dann. Aber irgendwie so spezifisch auf elektronische Geräte (I: Ja, ja.) und nicht auf Gedanken oder so, andere Handlungen. |
| 64 | **I:** Also, abgesehen von der Situation, wo Sie jetzt wirklich das Gefühl hatten: Ich und mein Körper, wir gehören gar nicht mehr so richtig zusammen (SF03a: Ja.), da- da ha- da grundsätzlich haben Sie schon eher das Gefühl, Gedanken, Handlungen Gefühle gehören zu Ihnen selbst? |
| 65 | **SF03a:** Ja. |
| 66 | **I:** Und bleiben (SF03a: Ja.) bei Ihnen selbst? |
| 67 | **SF03a:** Ja. Ja, also gehören zu mir selbst: Ja. Bleiben bei mir selbst: Nicht immer (I: Okay.). Also ich habe mal ein Gedicht geschrieben über eine Psychose. Ah, das kann ich- können Sie das haben. Das sind acht Zeilen. Äh da habe ich dann als letzte zwei Verse geschrieben: Was da tief in dir schlummert, ist jetzt in den Dingen erwacht. Und das beschreibt auch so ein bisschen mein Erleben. Und was ich manchmal das Gefühl habe, wenn ich im Wahn bin, im Verfolgungswahn oder so, dass ich durchschaubar bin. Also nicht, dass mir jemand direkt die Gedanken lesen kann, Wort für Wort, sondern dass jemand mich einfach durchschauen kann wie so einen gläsernen Menschen. Dass jemand mich anguckt und sofort weiß: Ich war schon in der Psychiatrie, ich habe Diagnose paranoide Schizophrenie (I: Hm (zustimmend)), ich- und alles, sozusagen. |
| 68 | **I:** Sie sind wie durchsichtig? |
| 69 | **SF03a:** Ich bin durchsichtig, ja (I: Ja), das Gefühl hatte ich manchmal |
| 70 | **I:** Okay. Okay. Das belastet Sie, das Gefühl? |
| 71 | **SF03a:** Es ist nicht angenehm (I: Ja, ja.), so durchschaubar zu sein. Aber ich meine, so psychotische Symptome in dem Sinne, habe ich jetzt- jetzt akut jetzt in dem Moment schon seit ein paar Wochen nicht mehr, das (I: Okay) belastet mich gerade nicht. |
| 72 | **I:** Okay. Also das geht auch wieder weg, dieses (SF03a: Ja.) Gefühl? |
| 73 | **SF03a:** Das war immer nur einzelne Momente, wenn ich alleine irgendwo an einem- rumgelaufen bin (I: Hm (zustimmend)) oder so. Kurzzeitig, jetzt, dass ich dann durchschaubar bin, ja. |
| 74 | **I:** Okay. Meistens dann vor krankheitsepisoden? Oder während Krankheits-? |
| 75 | **SF03a:** Während. |
| 76 | **I:** Währenddessen. Okay. Ähm. Wie ist es, wenn Sie in so Momenten, wo Sie das Gefühl haben, nicht so richtig in sich oder nicht so richtig anwesend zu sein (SF03a: Ja.). Müssen Sie dann viel darüber nachdenken, was Sie gerade tun? Wie Sie es tun? Ob Sie es richtig tun, oder nicht so richtig? Und- |
| 77 | **SF03a:** Also, als ich da letzten Freitag das ganz extrem habe, war ich dann so mit- so damit beschäftigt, die Situation zu erfassen oder nachzudenken, einfach darüber, dass ich eben doch im eigenen Körper stecke und dass das jetzt ziemlich seltsames Gefühl ist, dass ich eigentlich kaum irgendwas getan habe [LACHT] (I: Hm (zustimmend)), bis es wieder ein bisschen besser wurde (I: Okay.). Also ich stand ?(nunmehr)? in Gedanken verloren mitten in der Nacht im Bad (I: Ja, ja.) und habe eben dieses Gefühl gehabt (I: ja) nicht im richtigen Körper zu stecken. |
| 78 | **I:** Okay. Und so im Alltag? Wenn Sie in Interaktion mit anderen Leuten oder wenn Sie einkaufen gehen oder so (SF03a: Ja.), gibt es da Situationen, wo Sie besonders darüber nachdenken: Was tue ich gerade? Tue ich das richtig? Was sage ich (SF03a: Ja.) gerade? Sage ich das Richtige? |
| 79 | **SF03a:** Ja, schon bisschen. Ich weiche dann immer Leuten aus, mach mir ganz viele Sorgen, dass ich im Weg stehe oder so. Bin da sehr vorsichtig. Das würde ich schon sagen, aber denke nicht in einem krankhaften Maße [LACHT], wenn ich jetzt- wenn es mir gut geht, einkaufen (I: Ja) gehe. |
| 80 | **I:** Ja. Also ich will das auch nicht bewerten, ne? (SF03a: Ja) Das ähm hängt ja davon ab (SF03a: Ja), ob es Ihnen- Sie belastet oder (SF03a: mich) nicht. |
| 81 | **SF03a:** Also da gibt es jetzt keinen Leidensdruck, aber ich bin da (I: Hm (zustimmend)) schon irgendwie schüchtern [LACHT], beim einkaufen oder so, könnte man sagen. Oder weich dann eben Leuten viel aus. Guck, dass ich niemandem im Weg stehe und so. |
| 82 | **I:** Okay. Also diese Gedanken, die Sie dann- dieses Beobachten von den eigenen Handlungen, das (SF03a: Hm) beeinflusst schon auch so ein bisschen, wie Sie sich verhalten? |
| 83 | **SF03a:** Ja. Ja, also ich habe das Gefühl, dass ich mich selbst viel beobachte. Dass ich viel reflektiere, was positive und negative- also es kann eben auch gut sein, viel zu reflektieren und dann sich selbst gut zu kennen. Aber es kann auch manchmal in Grübeleien ausarten (I: Mh-h (zustimmend)). Aber generell würde ich sagen, bin ich sehr relativ reflektiert und relativ selbstbeobachtend. |
| 84 | **I:** Okay. Sie haben gesagt, Sie grübeln? Jetzt gerade haben Sie gesagt, Sie grübeln manchmal? |
| 85 | **SF03a:** Ja, wenn es mir halt schlechter geht (I: Ja?). Also die Depression ist dann manchmal eben auch mit innerer Leere verbunden. Aber manchmal auch mit ganz vielen Gedanken. Oder wenn es mir eben- Verstimmung vielleicht besser als Depression, Depress- wenn ich eine depressive Verstimmung habe, dann manchmal fühle ich eine innere Leere. Und manchmal fühle ich- habe ich dann dann auch ganz viele negative Gedanken. Und dass die wie vielen negativen Gedanken würde ich dann eben als Grübelei bezeichnen. Weil sie nicht zielführen. Weil sie nirgends hinführen. |
| 86 | **I:** Okay. Können Sie ein Beispiel nennen, was Sie da so denken? |
| 87 | **SF03a:** Ja, ich- teilweise denke ich an total unwichtige Situationen von vor acht Jahren, wo ich mich irgendwie komisch verhalten habe (I: Hm (zustimmend)) und gedacht habe, ja, da hätte ich vielleicht irgendwie mich anders verabschieden sollen oder so. Also wirklich sehr, sehr banal auch oft (I: Okay.). Aber ich mache mir dann eben Vorwürfe und gehe hart mit mir ins Gericht, sozusagen (I: Hm (zustimmend)). Aber jetzt nicht in dem Sinne, dass ich sage, ich muss es jetzt besser machen, sondern ich muss mich dann [LACHT] selbst bestrafen irgendwie, oder so. |
| 88 | **I:** Tun Sie das dann? |
| 89 | **SF03a:** Nein, also nicht das- nicht falsch verstehen, also ich- selbstverletzendes Verhalten, Ritzen oder Selbstmord oder so etwas (I: Hm (zustimmend)) habe ich nie gemacht. Also. Eher bestrafen in dem Sinne, dass ich mich nicht wertschätze, so war das |
| 90 | **I:** Okay. Das ist eine gedankliche Sache? |
| 91 | **SF03a:** Ja. |
| 92 | **I:** Also. Aber gibt es Situationen, wo Sie dann auch eine Handlung anschließen an solche Grübelein? Wo es irgendwas gibt, was Sie dann machen müssen? |
| 93 | **SF03a:** Grübeleien habe ich eher, wenn ich im Bett liege und nicht schlafen kann (I: Okay.) und dann mache ich dann eh nicht so viel (I: Okay.). Also eher nicht, ja. |
| 94 | **I:** Also keine Rituale? Keine Handlungen, die dann zusammenhängen? |
| 95 | **SF03a:** Vielleicht ein Rückzug, dass ich weniger rausgehe? (I: Ja, okay.) Oder eher das Ausbleiben von Handlungen. |
| 96 | **I:** Okay. Also das Nicht- Nichtmachen von (SF03a: Ja) Handlungen, ja. Alles klar. Hm. Jetzt haben Sie schon Gedanken angesprochen (SF03a: Ja.). Gibt es Situationen, in denen Sie Ihre Gedanken nicht so richtig gut nachvollziehen können? |
| 97 | **SF03a:** Ja, (I: Wo so ein-) definitiv. |
| 98 | **I:** Wo so ein Gedanke einschießt? |
| 99 | **SF03a:** Ja, also (I: Unterbricht?). Jetzt auch hier vor ein paar Wochen, vor der Remittierung der Psychose hatte ich dann eben- also manchmal war das so emotionaler Paranoia, dass ich einfach ein vages und diffuses Verfolgungsgefühl hatte. Aber es gab auch die Paranoia dann teilweise auf der kognitiven Ebene, dass ich einfach plötzlich die fixe Idee hatte: Aus diesem Auto, dass da gerade in der [ANONYMISIERT, ORT IN DEUTSCHLAND] steht, steigt gleich jemand aus, der mir etwas Böses will (I: Mh-h (zustimmend)). Und äh mir wird dann auch relativ schnell klar, dass es wahrscheinlich nicht so ganz zutreffend ist, aber das hat den- hat den Gedanken nicht unwirksam machen können [LACHT] (I: Okay.). Und dann bin ich halt eher geflüchtet vor dem Auto, was jetzt (I: Ja) nicht so, dann auch- nicht so ungewöhnlich war, weil ich bin dann einfach weggegangen. Das Auto stand ja da (I: Ja). Aber so Idee- also Gedanken, die einfach da sind, ohne dass sie wirklich einen Zusamm- einen logischen Zusammenhang oder wenigstens einen assoziaten Zusammenhang irgendwie entspringen, das kenne ich, ja. |
| 100 | **I:** Mh. Und das sind solche angstauslösenden Gedanken? |
| 101 | **SF03a:** Ja. |
| 102 | **I:** Jetzt in dem Fall? Gibt es das auch mit ganz banalen Gedanken? Dass Sie irgendetwas denken und plötzlich schießt ein ganz banaler Gedanke rein? |
| 103 | **SF03a:** Also. Grundsätzlich würde ich sagen, es gibt so eine Assoziations- ähm Toleranzgrenze. Also, weil wenn man Dinge assoziiert, dann haben sie irgendwelche Gemeinsamkeiten. Und die führen dann eben, wenn eine gewisse Schwelle über ähm schritten ist dazu, dass man eine Assoziation hat (I: Mh-h (zustimmend)). Und ich würde sagen, wenn es mir schlechter geht, wenn ich wahnhaftes Erleben habe, psychotisches Erleben, dann ist diese Schwelle sehr niedrig (I: Mh-h (zustimmend)). Dann kann ich auch sehr gut Gedichte schreiben zum Beispiel (I: Mh-h (zustimmend)), weil man da ja auch viel assoziieren muss (I: Mh-h (zustimmend)), viele Metaphern und Bilder und so. Aber- also grundsätzlich ist dann die Assoziationsschwelle sehr niedrig. Das heißt, man schie- es kommt schon mal irgendein Gedanke. Aber wenn es jetzt nicht um angstmachende Gedanken geht, würde ich nicht sagen, dass es komplett ohne Assoziation irgendwas kommt. |
| 104 | **I:** Okay, also die haben irgendwas mit dem Gedanken (SF03a: Ja) Zug, den Sie gerade haben (SF03a: Ja), sowieso zu tun? |
| 105 | **SF03a:** Genau. |
| 106 | **I:** Okay, mh-h. Und mh haben Sie dann auch manchmal das Gefühl, da drängen ganz viele Gedanken auf einmal? |
| 107 | **SF03a:** Das kenne ich, ja. |
| 108 | **I:** Oder wie so ein Salat im Kopf? |
| 109 | **SF03a:** Das kenne ich schon aus der frühen Kindheit, so (I: Okay.). Meine Mutter hat mir erzählt, als ich noch in einem Gitterbett geschlafen habe, also ja ich weiß nicht, wie das heißt, aber halt so ein Gitter (I: Ja, so ein, so ein), wo man dann drin ist (I: Mh (zustimmend)), dass ich dann irgendwie in meinem Bettchen nachts stand am Gitter und gesagt habe: Ich kann nicht schlafen. Die Gedanken hören nicht auf. Ich kann nicht aufhören zu denken (I: Okay.). Also da kann ich mich nicht wirklich erinnern, es gibt nur diesen- dieses Phänomen, dass wenn man es so oft erzählt bekommen hat, dass man dann irgendwann eine Erinnerung aus dem Nichts entwickelt (I: Ja), aber das kenne ich ganz gut, ja. Schon sehr lange. |
| 110 | **I:** Also das kennen Sie auch wo- von eigenen Erinnerungen? |
| 111 | **SF03a:** Ja. |
| 112 | **I:** Ja? [SIGNALHORN ERTÖNT] Und könnten Sie einmal beschreiben, wie sich das dann für Sie anfühlt? Ich habe jetzt da so ein paar Worte genannt, vielleicht finden Sie noch Ihre eigenen? |
| 113 | **SF03a:** Ja, also, es ist so ein Rauschen von Gedanken, so ein Sturm, ich weiß nicht, mir fallen jetzt nur Metaphern ein. |
| 114 | **I:** Das ist völlig in Ordnung. |
| 115 | **SF03a:** Also, es ist- man kann nicht mehr richtig filtern. Mann kann nicht- ich kann nicht mehr richtig filtern. Ich kann nicht mehr richtig die Gedanken einordnen in die richtige Bedeutung beimessen. Man hat ja sehr viele Gedanken oder Gedankenansätze, würde ich sagen. Wenn man irgendwas sieht, da gehört ja immer ein kognitives Element schon zur Wahrnehmung dazu (I: Mh-h (zustimmend)). Aber wenn ich- wenn es dann- wenn dann die Gedanken auf einen einströmen, dann würde ich sagen, ist der Filter kaputt, ist der- der äh am Computer gibt es so einen Prozessor [LACHT], so einen Task Scheduler, der äh der-der verschieden- den verschiedenen Prozessen zuordnet, was wichtiger ist (I: Ja), was zuerst (I: Ja) ausgeführt (I: Ja) werden muss. Das ist dann kaputt (I: Okay.) im Gehirn [LACHT]. |
| 116 | **I:** Also die- die- der Verteiler sozusagen (SF03a: Ja, genau). Der Ressourcenverteiler? |
| 117 | **SF03a:** Ja. |
| 118 | **I:** Ja? Mh-h. Alles klar. Und diese Gedanken, diese vielen Gedanken oder auch ein- einschießende Gedanken: Sind das immer ihre eigenen Gedanken? |
| 119 | **SF03a:** Ja, das sind meine Gedanken (I: Okay, also), das ist nichts Fremdes. |
| 120 | **I:** Alles klar. Okay. Und gab es auch schon Situationen, wo Gedanken einfach plötzlich wie weg waren? Sie haben angefangen, die zu denken, und plötzlich konnten Sie sie nicht mehr greifen? |
| 121 | **SF03a:** Also- das einem irgendwas auf der Zunge liegt, was man gerade sagen wollte, kennt glaube ich jeder so (I: Genau) ein bisschen [LACHT]. |
| 122 | **I:** Nee, das meine ich jetzt eher nicht, sonde- genau, sondern eher so, dass Sie einen Gedanken haben und der ist einfach plötzlich: Der verschwindet, oder löst sich langsam auf. |
| 123 | **SF03a:** Kenne ich nicht. |
| 124 | **I:** Kennen Sie nicht. Okay. Und ähm so dass sie das Gefühl haben, Ihre eigenen Gedanken hallen im Kopf wider, hallen nach? |
| 125 | **SF03a:** Das kenne ich so ein bisschen, ja. Also ich hatte schon mal einen Ohrwurm von einem Gedicht [LACHT]. Was eigentlich auch nicht so häufig ist (I: Mh-h (zustimmend)). Und den habe ich immer noch so ein [LACHT] bisschen. Das ist nicht von mir, das Gedicht (I: Ich wollte gerade fragen), sondern von Wilke. Ich mag das Gedicht auch gerne, aber es ist halt irgendwie ein bisschen komisch, weil es mir so oft in den Sinn kommt. Und dass Gedanken so nachhallen, widerhallen dann, öfter (I: [HUSTET]) wiederkehren, vielleicht ein bisschen schwächer, wie so ein Echo, das langsam, sich verliert (I: Mh-h (zustimmend)), das kenne ich, ja. |
| 126 | **I:** Okay. Haben Sie das öfter? |
| 127 | **SF03a:** Nee, eher nicht so. |
| 128 | **I:** Eher nicht so oft. Okay. Ist jetzt auch nichts, was Sie besonders (SF03a: belastet mich auch nicht sehr) belastet, okay. Ähm wie ist es mit dem Gefühl ähm dass sich Gedanken wie laut gesprochen im Kopf anhören? Haben Sie das schon mal erlebt? |
| 129 | **SF03a:** Mh, nein. |
| 130 | **I:** Nein? Oder so, dass Sie Ihre eigenen Gedanken wie im Film oder Video sehen können? |
| 131 | **SF03a:** Also so, dass man ein bisschen von außen auf die eigenen Gedanken blickt, oder so? |
| 132 | **I:** Ja? |
| 133 | **SF03a:** Eher nicht. |
| 134 | **I:** Nee? |
| 135 | **SF03a:** Nee, also das sind meine Gedanken und ich- ich identifiziere mich mit dem Gedanken (I: Ja?), also. |
| 136 | **I:** Okay. Und das ist auch nicht- also dass andere Leute- dass sie sich irgendwie ausdehnen könnten? Und vorhin haben Sie beschrieben, ähm manchmal fühlen Sie sich wie gläsern? |
| 137 | **SF03a:** Ja. |
| 138 | **I:** Ähm. Ge- gehört da vielleicht auch ein Gefühl dazu, dass sich die Gedanken ausdehnen räumlich? Und dann aus Ihnen rausgehen und zu anderen Leuten gehen? |
| 139 | **SF03a:** Nee (I: Nee), solche Metaphern habe ich jetzt noch nicht gehabt (I: das nicht), nee. |
| 140 | **I:** Okay. Gut. Wie ist es mit- mit Entscheidungen? Alltäglichen Entscheidungen? |
| 141 | **SF03a:** Dass ich viel mit mir hadere, oder meinen Sie so etwas? |
| 142 | **I:** Zum Beispiel. Also, mein Beispiel, was ich immer nehme, ist, wenn Sie einkaufen gehen und dann auswählen müssen: Was nehme ich? Oder wenn Sie sich morgens entscheiden müssen: Was ziehe ich an? Ist das leicht? Fällt Ihnen das schwer? |
| 143 | **SF03a:** Pff, das ist nicht so schwer, nee (I: Nö). Aber manchmal denke ich dann, wenn ich was größere, eine Anschaffung gemacht habe: Oh, war das jetzt so richtig? Oder so. Aber das ist denke ich auch normal [LACHT]. Also nee, ist jetzt nichts Problematisches. |
| 144 | **I:** Okay. Okay. Und wenn Sie sagen, ich- dass Sie viel mit sich hadern, dann meinen Sie, das was Sie vorhin beschrieben haben? Oder? |
| 145 | **SF03a:** Was genau? |
| 146 | **I:** Dass Sie- dass Sie zurückdenken und fragen (SF03a: Ja), habe ich mich richtig verhalten (I: ja, genau, das habe ich), ja? Okay. |
| 147 | **SF03a:** Aber das ist dann eher vergangenheitsbezogen (I: Mh-h (zustimmend)) als zukunftsbezogen (I: Mh-h (zustimmend)), sage ich mal. |
| 148 | **I:** Und was ist mit gegenwartsbezogen? Ist es auch gegenwartsbezogen manchmal? |
| 149 | **SF03a:** Ja, also, Zukunft im Sinne von auch naher Zukunft- also, ich meine die Zukunft wird dann ja sehr schnell Gegenwart, wenn sie noch nicht so weit vor- (I: Ja) voran- also, "was soll ich jetzt machen?" wäre für mich auch zukunftsbezogen (I: Okay.), weil ich muss mich ja erst entscheiden und dann das machen |
| 150 | **I:** Und da hadern Sie auch? |
| 151 | **SF03a:** Da hadere ich nicht. |
| 152 | **I:** Da hadern Sie nicht? Sondern- |
| 153 | **SF03a:** Ich hadere mehr so vergangenheitsbezogen. |
| 154 | **I:** Alles klar. Alles klar. Okay. Wie ist es mit Ihrer Aufmerksamkeit? Wenn wir jetzt zum Beispiel uns unterhalten und (SF03a: Ja) nebenan würde ein Radio laufen. Würde das stören? |
| 155 | **SF03a:** Kommt darauf an, wie laut das ist (I: [LACHT]) und was so läuft. So ein bisschen- bisschen Klaviermusik, hätte ich [LACHT] nichts dagegen (I: Okay, okay), nein. |
| 156 | **I:** Ja. Also das ist kein Problem, dass Sie sich sozusagen das ausblenden können? |
| 157 | **SF03a:** Nein, ich meine, draußen ist jetzt ja auch ein bisschen Verkehr (I: Hm (zustimmend)) oder Vögel. Das stört mich nicht. |
| 158 | **I:** Okay. Alles klar. Oder die Situation, dass irgendwelche ähm Dinge in Ihrer Umwelt so besonders Ihre Aufmerksamkeit auf sich ziehen. Gibt es so etwas? |
| 159 | **SF03a:** Fällt mir jetzt nichts ein. |
| 160 | **I:** Alles klar. Und ähm das Kurzzeitgedächtnis? |
| 161 | **SF03a:** Normal, würde ich sagen. |
| 162 | **I:** Ja? Wenn Sie ein Buch lesen, klappt einwandfrei? |
| 163 | **SF03a:** Ja. |
| 164 | **I:** Ja? Oder einen Film gucken? Dann wissen Sie auch noch, was- oder eine Serie, wissen Sie auch noch, was bei der letzten Serie passiert ist? |
| 165 | **SF03a:** Ja. |
| 166 | **I:** So? Okay. Und ähm. Das Erleben von Zeit. Wenn Sie sagen- die- die Zeit, wie erleben Sie die Zeit? |
| 167 | **SF03a:** Buff (I: Schw- große Frage, ich weiß). Soll ich jetzt mit Heidegger anfangen? (I und SF03a: [LACHEN]), (I: ähm) das wär nicht so (I: das können Sie machen-), das wär nicht im Sinne von der Epoche. |
| 168 | **I:** Es geht eher um Ihre persönliche Erfahrung. |
| 169 | **SF03a:** Ja. Ahm. Wie erlebe ich die Zeit? Also ich finde, Zeiterleben hat ein paradoxes Moment, und zwar, wenn man viele Dinge tut, vergeht die Zeit in dem Moment schnell. Und im Nachhinein kommt es einem so, als wäre sehr viel Zeit vergangen. Und wenn man hingegen wenig tut oder nur im Bett liegt, sich zurückzieht, dann vergeht die Zeit im Moment sehr langsam und im Nachhinein hat man das Gefühl, dass eigentlich gar nichts passiert ist (I: Hm (zustimmend)) und nicht viel Zeit vergangen ist (I: Hm (zustimmend)). Also das ist so, würde ich sagen, die Paradoxie vom Zeitempfinden. |
| 170 | **I:** Das ist bei Ihnen auch so? |
| 171 | **SF03a:** Das ist bei mir was- auch so. |
| 172 | **I:** Das- also so erleben Sie selbst? Weil Sie (I: Ja) haben jetzt man gesagt, die ganze Zeit. |
| 173 | **SF03a:** Ja, ja, achso. Ja, ich habe auch schon mal was in die Richtung in einem Roman gelesen. Kennen Sie (unverständlich) äh nee Quatsch äh "Herr Jensen steigt aus"? |
| 174 | **I:** Ah, von? |
| 175 | **SF03a:** Ich weiß nicht, wie der heißt. Das haben wir in der Schule gelesen. Da gibt es ein Kapitel (I: Ja (erstaunt)), das beschreibt genau diese Sache, die ich gerade gesagt habe. |
| 176 | **I:** Ja. Ich- also es ring- it rings a bell. |
| 177 | **SF03a:** Ja? (I: Ja), okay (I: Also, so). "Herr Jensen steigt aus" ist ein sehr guter Roman (I: Mh-h (zustimmend)), sehr interessant, kann ich empfehlen (I: Okay.) Aber, ja. |
| 178 | **I:** Ähm, und, das heißt, wenn Sie selbst- wenn Sie wenig machen (I: Ja), und es passiert wenig, dann vergeht die Zeit langsam, und wenn Sie viel machen, dann vergeht sie schnell? |
| 179 | **SF03a:** Ja, aber im Moment (I: In dem Moment), aber in der Retrospektive ist es dann genau umgekehrt. |
| 180 | **I:** Okay. Und dieses Schema, sozusagen, das wurde auch noch nie aufgebrochen. Das ist bei Ihnen schon immer so gewesen? Es ist nicht so gewesen, dass die Zeit mal, seltsamerweise, schnell, superschnell ablief, oder mal seltsamerweise superlangsam, obwohl es gerade nicht passte? In Ihrer Wahrnehmung? |
| 181 | **SF03a:** Nee, ich würde sagen, das Schema mit dem Gegenwartsbez- das ist genau- sich einen Widerspruch gibt zwischen dem momentanen Zeiterleben und dem retrospektiven Zeiterleben. Das ist konstant so. |
| 182 | **I:** Okay. Mh-h. Und ähm gab es schon mal Situationen, wo Sie das Gef- wo Sie so Lücken, oder hm ja Lücken im Bewusstsein hatten? Wo Sie zum Beispiel irgendwo hingegangen sind und plötzlich nicht mehr so genau wussten, wie bin ich jetzt eigentlich gerade da hingekommen? |
| 183 | **SF03a:** Also mir wird immer berichtet, dass ich manchmal recht gedankenverloren durch die Gegend spaziere. Das stimmt auch. Also manchmal kriege ich nicht mit, wenn Leute mich grüßen [LACHT] (I: Mh-h (zustimmend)), weil ich so in Gedanken bin. Die Leute wissen das auch und finden es eher lustig als dass sie sich dann angegriffen fühlen. Ähm also ich denke sehr gerne nach, wenn ich laufe, und dann kann es auch mal passieren, dass ich einfach irgendwo hinlaufe. Aber dann weiß ich, dass das halt war, weil ich gerade so intensiv in Gedanken gegangen bin (I: ja) und an dem tatsächlichen Gang nicht so viel Bedeutung beigemessen habe. Also (I: Okay.) würde ich es nicht als so belastend beschreiben. |
| 184 | **I:** So da- da waren Sie auch dann bewusst im Kopf (SF03a: Ja) in dem Moment? Also Sie wissen (SF03a: Ja) Ihre Gedanken auch noch, die Sie dann (SF03a: Ja) hatten in dem Zeitpunkt? |
| 185 | **SF03a:** Ja, (I: Okay.) genau. |
| 186 | **I:** Weil es nicht wie so eine- wie so ausgeschnitten, aus dem (SF03a: nein) Kurzzeit- |
| 187 | **SF03a:** Das ist nicht so ein (I: Okay) Filmriss, oder wie man sagt. |
| 188 | **I:** Genau, aber ohne, dass Sie irgendwelche- (SF03a: nein) dass Sie irgendwas getrunkten hätten (SF03a: ja) oder so, ne? |
| 189 | **SF03a:** Ja, genau, nee, so etwas hatte ich nicht. Habe ich nicht. |
| 190 | **I:** Okay. Wie ist es, wenn wir uns unterhalten? Haben Sie dann das Gefühl, das was innen in Ihnen vorgeht, können Sie so ausdrücken (SF03a: Ja), wie Sie möchten? Ja? |
| 191 | **SF03a:** Also ich schreibe ja auch kreati- so Gedichte, oder Kurzgeschichten, und so. Also mich zu artikulieren, ist jetzt nicht problematisch (I: Okay.), würde ich sagen. |
| 192 | **I:** Das passt auch zu dem (SF03a: Ja), was Sie empfinden? |
| 193 | **SF03a:** Ja. |
| 194 | **I:** Okay. Gut. Ähm. Lassen Sie uns nochmal über dieses Gefühl reden, nicht so richtig Teil der Gesellschaft, haben Sie gesagt- |
| 195 | **SF03a:** Ja (I: zu sein), das war schon ziemlich lange her. Mit 14, aber ja (I: Mh-h (zustimmend)), könnte man. |
| 196 | **I:** Das ist heute nicht mehr so? |
| 197 | **SF03a:** Nein (I: Okay.). Also soziale Phobie wurde nach der Episode mit 14 auch nie wieder als Diagnose gestellt. |
| 198 | **I:** Ja. Ja. Und was würden Sie sagen, wie lange Sie dieses- jetzt gar nicht die Diagnose, sondern Ihr Gefühl, wie lange Sie das hatten? |
| 199 | **SF03a:** Ah [ATMET AUS]. Ich würde sagen, seit ich jetzt hier in [ANONYMISIERT, ORT DES INTERVIEWS] studiere, hatte ich das nicht mehr. Weil ich da so gut, so gut- mh mit den sozialen Kontakten es alles so gut läuft. In der Oberstufe, da nachdem ich zwei Klassen übersprungen hatte, hatte ich es schon noch so ein bisschen. Was ja auch an der Situation nicht so fern liegt. Weil ich dann als ganz neu dazu gekommen bin (I: Hm (zustimmend)). Aber so richtig akut hatte ich es eigentlich wirklich mehr mit 14. |
| 200 | **I:** Okay. Also in der Jugend? |
| 201 | **SF03a:** In der (I: Mh-h (zustimmend)), ja. |
| 202 | **I:** Haben Sie schon mal die Erfahrung gemacht, ähm vielleicht zu dem Zeitpunkt, oder auch zu einem späteren Zeitpunkt, nicht so richtig an der Welt teilhaben zu können? Sich nicht so richtig berührt zu fühlen von der Sonne, von den Dingen, vom Essen vielleicht? |
| 203 | **SF03a:** [PAUSE] Nein, also, ich habe mich schon in der Welt gefühlt. Es gab dann schon ein In-Der-Welt-Sein. (I: Mh-h (zustimmend)), würde ich sagen. Ich war nicht außerhalb der Welt, sondern schon in der Welt. |
| 204 | **I:** Okay. Okay. [PAUSE] Mh (überlegend). Das Gefühl, sich selbst zu teilen haben Sie vorhin einmal kurz beschrieben. Das war so eine isolierte Situation, wo Sie das einmal so extrem hatten. |
| 205 | **SF03a:** Mich zu teilen? |
| 206 | **I:** So- Sie hatten gesagt, Ihr Körper und Ihr Geist (SF03a: Achso, ja) hat sich (SF03a: ja) geteilt? |
| 207 | **SF03a:** Ja (I: Mh-h (zustimmend)), das hat nicht mehr zusammengepasst. |
| 208 | **I:** Ja, hat nicht mehr zusammengepasst plötzlich? |
| 209 | **SF03a:** Ja. |
| 210 | **I:** Woll- könnten Sie das nochmal (SF03a: Ja) bezogen auf das Körpergefühl ein bisschen beschreiben? |
| 211 | **SF03a:** Ja also das war g-ganz ungewohnt, wie mh wie wenn man neue Schuhe hat, an die sich die Füße noch nicht gewöhnt haben (I: Mh-h (zustimmend)), aber bezogen auf den ganzen Körper. |
| 212 | **I:** Hm (zustimmend). Der war ja nicht neu, der Körper (SF03a: nein), eigentlich ne? |
| 213 | **SF03a:** Ja. Das hat sich- hat sich sehr ungewohnt, seltsam und falsch angefühlt. |
| 214 | **I:** Ja. Ja. Hatten Sie da auch das Gefühl, Sie können sich von außen sehen? |
| 215 | **SF03a:** Nein. |
| 216 | **I:** Nein. Das war nur so ein Fehlende-Puzzle-Gefühl? |
| 217 | **SF03a:** Ja. |
| 218 | **I:** Ja. |
| 219 | **SF03a:** Ich war schon in dem Körper, aber irgendwie halt nicht- fälschlicherweise [LACHT] (I: Hm (zustimmend)), so hat es sich angefühlt. |
| 220 | **I:** Mh (zustimmend). Also i- ich ver- ich versuche es einfach (SF03a: Ja) zu verstehen, deswegen (SF03a: ja) frage ich so viel nach (SF03a: natürlich), also. Der Körper ist auch nicht auseinandergefallen (SF03a: nein), sondern Körper und Geist sind so ein bisschen auseinander (SF03a: getrennt, ja) getrennt gewesen. Okay. |
| 221 | **SF03a:** So also der Geist war im Körper, aber es hat nicht so ganz gepasst (I: Mh (zustimmend), okay). Wie wenn man versucht, ich weiß es nicht, Windows auf einen Applerechner aufzuspielen oder so. |
| 222 | **I:** Ja? [LACHT] Ja? Gut, aber gleich. Und- und ähm ga- ging es mit einer- Sie haben gesagt, Sie haben sich da auch Ihre Hände angeguckt (SF03a: Mh-h (zustimmend)) und so? Ging das- |
| 223 | **SF03a:** In den- in den Spiegel geguckt (I: Mh-h (zustimmend)), auch. |
| 224 | **I:** Okay. Ging das mit einer bestimmten Wahrnehmung einher? Dass Sie etwas Bestimmtes gesehen haben, auch? |
| 225 | **SF03a:** Also als ich in den Spiegel geguckt habe, kam- kam ich mir einfach wie eine unbekannte Person vor. Also das Gesicht, das ich gesehen habe, dachte: "Mh, wer ist das? Den kenne ich gar nicht", so. Und die Hände- auf die Hände zu gucken, ich weiß nicht, warum ich das genau gemacht hatte, also liegt ja irgendwie nah am Sichtfeld, die Hände. |
| 226 | **I:** Mh. Ja klar, das macht- das ist die erste Reaktion, die man hat, oder? |
| 227 | **SF03a:** Ja |
| 228 | **I:** Ähm. Gehört das noch zu mir? Könn- (SF03a: Ja, genau) kann ich nachvollziehen. Ähm die Frage ist aber auch, ob sich- ob die Hände- sahen die dann anders aus, oder? |
| 229 | **SF03a:** Ja, die sahen auch irgendwie fremd aus. Ich meine, man kennt ja irgendwie die Hände ein bisschen (I: ja) von sich so (I: Ja.) und die Linien und die äh da gibt es ja sehr feine Rillen dadrauf (I: Mh (zustimmend)) und das kam mir alles irgendwie neu vor. |
| 230 | **I:** Okay. Okay. Okay. Also Das war dann- dann in dem Moment haben Sie dann auch in den Spiegel geguckt und geguckt, sin- bin ich noch ich selbst (SF03a: Ja). Gab es denn früher auch schon mal Phasen, wo Sie das Gefühl hatten, ich muss in den Spiegel gucken, um zu gucken: Bin ich noch ich selbst? |
| 231 | **SF03a:** Also dass ich auf die Hände geguckt hatte, hatte ich auch da mit 17 als es mir so richtig schlecht ging [LACHT VERLEGEN] (I: Mh-h (zustimmend)). Also als [/ich/] die schlimmste Erkrankungsphase hatte, würde ich sagen, habe ich mir auch manchmal auf die Hände geguckt, einfach mehr um mich zu vergewissern, dass ich noch ich selbst bin. Aber dann war es meistens so, dass ich gesehen habe: Okay, ah das sind meine Hände (I: Ja.) und das ist okay. Aber ich habe es dann schon gemacht um (I: Ja.) um mich zu- um sicher zu gehen. |
| 232 | **I:** Ja. Also dann gab es so Anflüge (SF03a: Ja) von- von diesem Gefühl auch schon mal? |
| 233 | **SF03a:** Ja. |
| 234 | **I:** So ich könnte irgendwie meinen Geist und mein Körper könnten sich irgendwie trennen voneinander? |
| 235 | **SF03a:** Ja |
| 236 | **I:** Ja. Mh. |
| 237 | **SF03a:** So, Uneigentlichkeit. |
| 238 | **I:** Uneigentlich? |
| 239 | **SF03a:** Keit |
| 240 | **I:** Dann erklären Sie es mir. Was meinen Sie damit? |
| 241 | **SF03a:** Also ich wollte- also, das ist jetzt irgendwie, ich bin sehr Heidegger-Fan, kann man nicht sagen, aber seine Zeit finde ich sehr interessant. Und da gibt es eben zwei Seinsmodi und (I: Mh-h (zustimmend)) die heißen Eigentlichkeit und Uneigentlichkeit (I: Ja, ja). Und ähm in der Eigentlichkeit nimmt man sich selbst wirklich als- als Teil der Welt wahr. Und da ist der Bezug zu den Dingen auch ein praktischer, kein rein beobachtender. Da ist die Welt- erscheint einem die einzelnen Bestandteile der Welt als Zeug und nicht als Dinge, weil man damit etwas machen kann wie mit Werkzeug (I: Ja, ah). Und in der Uneigentlichkeit, das sind entspricht dann so ein bisschen dem kartesischem Menschenbild, von Descartes, dann ist- ist man- nimmt man sich selbst als losgelöst von der Welt wahr (I: Mh-h (zustimmend)). Und bloß beobachtendes- bloß beobachten. Und da sind die- besteht die Welt auch nur aus Dingen (I: Mh-h (zustimmend)) und nicht aus Zeug. |
| 242 | **I:** Also Dinge, die keine (SF03a: Ja), keine Handlungs- [Crosstalk] |
| 243 | **SF03a:** Dinge sind, die nur von dem beobachtenden Subjekt wahrgenommen werden (I: Mh-h (zustimmend)) und darin liegt ihre ganze Existenz (I: Mh-h (zustimmend)), sozusagen. (I: Und) Und das ist mir halt gerade gekommen (I: Okay.), diese heidegge'sche Terminologie [LACHT]. |
| 244 | **I:** Ja? Ja, die passt gut. Ähm. Wo würden Sie sich verorten, in diesen Terminologien? |
| 245 | **SF03a:** Also ich bin ein relativ vergeistigter Mensch [LACHT], also bin ich- bin ich schon öfter mal im Modus [LACHT] der Uneigentlichkeit. Aber das ist auch ganz normal laut Heidegger. Dass man in diesen Modus verfällt. Dass man der Uneigentlichkeit verfällt (I: Mh-h (zustimmend)). Aber wenn ich dann wieder so etwas mache wie Ergotherapie oder- oder Gedichte schreiben, obwohl, das ist auch etwas Geistiges, aber trotzdem. Ähm. Oder- oder Joggen gehen, oder Bouldern oder so etwas. Dann bin ich auch im Modus der Ei- dann fühle ich mich auch selbst mehr als Teil der Welt und weniger als [LACHT] beobachtendes Subjekt. |
| 246 | **I:** Mh-h. Okay. Also es gibt Aktivitäten, die Sie sozusagen in den anderen Modus (SF03a: ja) bringen? Mh. |
| 247 | **SF03a:** Also im Sinne (I: Aber) von Heidegger (I: Ja.), ja. |
| 248 | **I:** Ja, ja, in dieser Terminologie (SF03a: ja) gesprochen, ja (SF03a: ja). Und äh und in diesen Situationen, die S- die Ihnen- die belastend waren, die auch Angst gemacht haben (SF03a: ja) bestimmt, ne? Da waren Sie massiv in der Un- in der Uneinigkeit? |
| 249 | **SF03a:** Uneigentlichkeit. |
| 250 | **I:** Uneigentlichkeit (SF03a: Ja.). In diesem Modus? Oder hat das damit nichts zu tun, diese Erfahrung? |
| 251 | **SF03a:** Also die Erfahrung, wie ich sie letzten Freitag am stärksten hatte, und wie sie in Anflügen schon vorher da war, hat denke ich schon stark etwas mit der Uneigentlichkeit zu tun (I: Okay.). Aber Angstempfinden im Sinne von Verfolgtsein wär wieder die andere Ecke. Weil, wenn man nicht in der Welt ist, kann man auch nicht verfolgt sein (I: Hm (zustimmend)), so. |
| 252 | **I:** Okay. Okay. Also das ist noch einmal eine andere Geschichte? |
| 253 | **SF03a:** Ja. |
| 254 | **I:** Über Angst sprechen wir gleich (SF03a: ja) nochmal detailliert (SF03a: okay, ja.). Okay. Mh (zustimmend). Okay. Gab es irgendwelche anderen vom Verlauf der Zeit bis heute jetzt (SF03a: ja), irgendwelche anderen ähm seltsamen Körperempfindungen, die Sie- an die Sie sich erinnern können? Die Sie sich nicht erklären konnten? Schmerzen, die Sie sich nicht erklären könnten? |
| 255 | **SF03a:** Zählt ein Tinnitus [LACHT] dazu? |
| 256 | **I:** Den hm (überlegend) Tinnitus? Okay. Den haben Sie? |
| 257 | **SF03a:** Hm gerade nicht. |
| 258 | **I:** Ist der diagnostiziert? |
| 259 | **SF03a:** Als ich mit 17 in der Psychiatrie war, stand der Tinnitus auris als Diagnose mit auf dem Entlassbrief (I: Ja.). Aber gerade habe ich den nicht. |
| 260 | **I:** Okay, der ist weggegangen? |
| 261 | **SF03a:** Ja. |
| 262 | **I:** Alles klar. Okay. Tinnitus. Alles klar, eine Sache. Irgendwelche anderen Körperempfindungen? So etwas wie ein Kribbeln, Schweregefühl, Schmerzen, die sich nicht- die Sie sich nicht erklären können? |
| 263 | **SF03a:** Ich hab- wackel manchmal mit den Beinen so (I: Okay.), auch gerade, ja [SCHNAUFT LACHEND]. |
| 264 | **I:** Okay. Gut, aber das ist ja alles, da- das können Sie beeinflussen? Da haben Sie (SF03a: ja), ja? |
| 265 | **SF03a:** Manchmal mache ich es auch ohne nachzudenken, wenn ich irgendwie (undeutlich) (I: Ja.), das ist denke ich jetzt nicht so unüblich. |
| 266 | **I:** Ja. Aber wenn Sie dann darauf schauen, dann? |
| 267 | **SF03a:** Dann kann ich auch aufhören, ja. |
| 268 | **I:** Genau, alles klar. Grundsätzlich: Wie ist es mit Bewegungen? Wie nehmen Sie Ihre eigenen Bewegungen wahr? |
| 269 | **SF03a:** Mh wenn ich in Gedanken vertieft bin, eher ni- gar nicht [LACHT]. Dann bewege ich mich halt intuitiv. Und äh dann bringt dir das nicht so viel. Ansonsten habe ich- irgendwie war ich jetzt auch schon beim Orthopäden und in der- in der Physiotherapie und als ich einmal- ah, die- die- die Phase hatte ich vergessen. Ich war auch mal in der Tagesklinik in [ANONYMISIERT, ORT IN DEUTSCHLAND] (I: Mh-h (zustimmend)) und die haben mich dann irgendwie auch, weil ich so unaufrecht gehe und so, haben die mich auch zum Orthopäden geschickt (I: Okay.). Da war Bewegung irgendwie ein wichtiges Thema. Aber das ist auch schon ziemlich lange her ist, erinnere ich mich gar nicht so dran. Wenn Sie wollen, können Sie aber den Brief von der Tagesklinik anfordern. Ich entbinde Sie hiermit von der Schweigepflicht. |
| 270 | **I:** [LACHT]. Es geht mir gar nicht so sehr [/darum/], was da wirklich war, sondern (SF03a: ja) um Ihre eigene Erfahrung (SF03a: achso). Mh-h. (SF03a: Ja ich-) Wie erleben Sie Ihre eigenen Bewegungen? |
| 271 | **SF03a:** Wie erlebe ich meine Bewegungen? Ja, manchmal etwas unbeholfen. Dass ich nicht weiß, wie ich mich richtig bewegen soll, so. Ich hatte mal mir den großen Zeh gebrochen. Bin halbes Jahr mit Krücken durch die Gegend gelaufen. Und der Orthopäde meinte: Könnte auch davon kommen. Dass ich irgendwie teilweise nicht richtig gehe. Also mich nicht richtig abrolle, wie man das macht (I: Mh-h (zustimmend)), sondern irgendwie so platt auftrete (I: Mh-h (zustimmend)). Ahm, aber da habe ich jetzt- war ich schon beim Orthopäden, habe mir Einlagen geben lassen und war dann in der Physiotherapie. Seitdem ist das auch besser. Da also da konnte man dann auch etwas machen. |
| 272 | **I:** Okay. Und ähm wie ist es so mit alltäglichen Bewegungen oder Handlungen? So etwas, was ganz automatisch abläuft eigentlich? Schuhe binden, ähm Mantel anziehen, T-Shirt anziehen? Ähm gab es Situationen, wo Sie das Gefühl hatten: Oh, jetzt weiß ich gerade gar nicht mehr genau, wie das geht und ich muss darüber nachdenken? |
| 273 | **SF03a:** Nein. |
| 274 | **I:** Nein. Okay. Und gab es Situationen, wo Sie Bewegungen gemacht haben, oder das Gefühl hatten, Bewegungen gemacht zu haben und andere haben die gar nicht sehen können? |
| 275 | **SF03a:** Nein. |
| 276 | **I:** Auch nicht. Oder so was wie so eine Lähmung, Sie wollten sich bewegen, aber das ging nicht? |
| 277 | **SF03a:** Nein. |
| 278 | **I:** Alles klar. Und so eine Situation, wo Sie das Gefühl hatten, jemand anderes macht das gleiche, was ich mache und ich weiß nicht, warum? Imitiert mich? Spiegelt mich? |
| 279 | **SF03a:** Mh, (I: [RÄUSPERT SICH)] nein. |
| 280 | **I:** Mh-h. Wie empfinden Sie Ihr eigenes Alter? |
| 281 | **SF03a:** Ja, so alt bin ich ja noch nicht [LACHT]. |
| 282 | **I:** Wie alt sind Sie jetzt? |
| 283 | **SF03a:** [ANONYMISIERT, ALTER DER PERSON]. Also. Keine Ahnung [LACHT]. |
| 284 | **I:** Passt die? Die [ANONYMISIERT, ALTER DER PERSON]? Zu wie Sie sich fühlen [RÄUSPERT SICH]? |
| 285 | **SF03a:** Ja. |
| 286 | **I:** Ja? |
| 287 | **SF03a:** Ich bin kein Teenager mehr. Und schon- bin ein bisschen nicht mehr- bin jetzt auch nicht, wie soll man sagen? |
| 288 | **I:** Okay, passt also so. Die Zahl (SF03a: Ja) passt zu dem Gefühl, was Sie haben. Und wie sieht es da in Bezug auf Ihre Geschlechtsidentität oder Ihre sexuelle Identität aus? Passt das auch? |
| 289 | **SF03a:** Mh da möchte ich nicht darüber reden. |
| 290 | **I:** Alles klar. Kein Problem. Mh. Hatten Sie schon mal für eine längere Zeit das Gefühl, Ihnen geht so die Sicherheit, die Selbstverständlichkeit verloren, mit der Sie Dinge tun? Dass Sie sich fragen: Warum machen eigentlich Leute- warum halten alle Leute an, wenn die Ampel rot ist? Warum ist sie überhaupt rot? Warum nehmen wir nicht eine andere Farbe? Solche, dass Sie so ganz selbstverständliche Dinge, für alle anderen selbstverständliche Dinge infragegestellt (SF03a: mh) haben? |
| 291 | **SF03a:** Nein. |
| 292 | **I:** Okay. [PAUSE] Mh. Ich habe Sie so etwas Ähnliches vorhin schon mal gefragt (SF03a: Okay). Ähm. Gab es Zeiten, wo Sie Schwierigkeiten hatten, Lust oder Spaß zu empfinden? |
| 293 | **SF03a:** Oh ja. Jetzt hier, bevor ich auf [ANONYMISIERT, NAME EINER ÖRTLICHEN PSYCHIATRISCHEN STATION] gekommen bin, da hatte ich auch- hatte ich an nichts Interesse, an nichts Freude. Da wollte ich einfach gar nichts machen. Habe ich nur im Bett gelegen um (I: Okay.) nichts zu tun. |
| 294 | **I:** Und das ging über einen längeren Zeitraum? |
| 295 | **SF03a:** Ja, das ging ein paar Wochen, ja. Und irgendwann ist dann halt meine Mutter gekommen und hat mir geholfen, ja. |
| 296 | **I:** Also Dinge, die Ihnen vorher Spaß gemacht haben (SF03a: ja), waren dann plötzlich- |
| 297 | **SF03a:** Ja, also ich war komplett interessen- und antriebslos. |
| 298 | **I:** Mh-h. Alles klar. Und mh gab es in diesen Sit- in dieser Phase auch das Gefühl so, keine Energie mehr zu haben? Die Dinge- (SF03a: Mh nee) die Dinge sind irgendwie schwer, ich kann mich nicht dazu aufraffen, sie zu tun? |
| 299 | **SF03a:** Ja. |
| 300 | **I:** Ja? |
| 301 | **SF03a:** Also ich fand es dann sogar schwierig, an das klingelnde Telefon, das neben dem Bett stand, zu gehen, von dem ich wusste, dass meine Mutter anruft, um mir zu helfen (I: Okay.). Aber selbst das Telefon abzunehmen und dann zu reden äh da habe ich irgendwie die Energie dafür nicht aufgebracht (I: Ja, ja), auch nicht aufbringen können. |
| 302 | **I:** Und das war jetzt- war das vereinzelt, also jetzt vor dieser Zeit so? Oder kam- gab es so etwas schon mal? |
| 303 | **SF03a:** Also so eine schwere Depression hatte ich noch nicht, nee. Also so eine schw- Antriebslosigkeit und innere Leere in dem Maß (I: Mh-h (zustimmend)) hatte ich vorher nicht. |
| 304 | **I:** Mh-h. Und in fänge- verringertem Maß? |
| 305 | **SF03a:** Ja, innere Leere. Schon manchmal. Aber so Energielosigkeit nicht so sehr. Also vielleicht, dass ich nicht so viel Lust auf andere Sachen habe. Aber eigentlich hatte ich dann auch immer viel Interessen. Selbst, wenn es mir nicht so gut ging. (I: Ja.). Also ich habe dann auch in der Klinik ganz viele Romane gelesen, und, Umberto Eco, und äh (unverständlich) |
| 306 | **I:** Und Latein haben Sie gelernt, haben Sie gesagt. |
| 307 | **SF03a:** Ja (I: ja, solche Sache) genau, und halt Griechisch. |
| 308 | **I:** Ja. Okay. Also. Eigentlich ging es immer- ging es immer- |
| 309 | **SF03a:** Vielleicht we- wenn- wenn es wirklich akut war, bevor es behandelt wurde, bevor ich in die Klinik dann gekommen bin, dann schon so ein bisschen (I: Mh-h (zustimmend)), dass ich mich- es nicht mehr interessiert hat. Aber dann so ein bisschen die Tagesstruktur wieder da war (I: Mh-h (zustimmend)), ich wieder regelmäßig geschlafen und gegessen habe (I: Mh-h (zustimmend)), dann kam auch eigentlich immer relativ schnell die Interessen wieder. |
| 310 | **I:** Mh-h, okay. Okay. Okay. Dann kommen wir zu unserem letzten Bereich (SF03a: Mh-h (zustimmend)) vom Interview. Da geht es um Weltanschauung, philosophische Themen (SF03a: Ohje [LACHT SCHNAUBEND]). Ähm haben wir schon ein bissches angeschnitten, alles (SF03a: Ja), aber um ja, vielleicht auch religiöse Themen (SF03a: Okay). Was fällt Ihnen da spontan dazu ein? |
| 311 | **SF03a:** Ja. Also ich habe da schon auch mit dem katholischen Seelsorger hier auf Station [LACHT] darüber geredet. Also ich glaube an Gott und ich habe einen sehr phänomenologischen [LACHT] Gotteszugang (I: Mh-h (zustimmend)). Und zwar empfinde ich manchmal, egal ob es mir jetzt gerade besonders gut geht oder besonders schlecht geht, eine Dankbarkeit, die ich nicht richtig zuordnen kann. Obwohl ich auch sagen würde, dass ist eine metaphysische Angst, so wie Dürrenmatt über die Existentialisten schreibst, sie hatten metaph- äh metaphysische Dankbarkeit, ja. Dürrenmatt schreibt über die Existentialisten, die hatten metaphysische Angst (I: Mh-h (zustimmend)). Ich würde sagen, ich empfinde irgendwie auch so eine metaphysische Dankbarkeit. Ich bin einfach froh und dankbar, dafür, dass ich existiere. Dafür, dass es mir wie auch immer ausgeprägt gut geht und ja. Also diese Dankbarkeit, dieses Dankenwollen konnte ich dann nicht richtig zuordnen. Bis ich es dann irgendwie so theologisch gedeutet habe. Also am Anfang von Sein und Zeit [LACHT] schreibt Heidegger über die Struktur der Frage. Und er schreibt zur Frage, gehören drei- zum Fragen gehören drei Elemente (I: Mh-h (zustimmend)): Das Erfragte, das Befragte und das Gefragte (I: Mh-h (zustimmend)). Und ich würde sagen, zum Denk- zum Danken gehören auch mehre Elemente [HANDY ERTÖNT]. Es gehört dazu das Bedankte, und das würde ich dann als Gottheit definieren, weil Dankbarkeit auf einer metaphysischen Ebene so sublimiert ist [LACHT], kann es irgendwie nicht- sich auf nichts Weltliches beziehen. Und das Bedank- äh das wäre das Bedankte, das Gedanke, das wofür ich mich bedanke (I: Mh-h (zustimmend)), würde ich dann im äh theologischen Tradition Gnade nennen (I: Mh-h (zustimmend)) und das Erdankte, das was aus dem Danken erwächst, würde ich dann Glaube nennen [LACHT]. |
| 312 | **I:** Ah, okay. Und diese Sicht, haben Sie die entwickelt über die Zeit? Oder sind Sie schon- |
| 313 | **SF03a:** Ja, auch in Auseinandersetzung mit Heidegger eben [LACHT]. |
| 314 | **I:** Okay. Und das heißt, früher waren Sie nicht gläubig? |
| 315 | **SF03a:** Nein, ich habe Nietzsche und Schopenhauer gelesen [LACHT] und war ganz antireligiös (I: Okay.) eine Zeit lang. |
| 316 | **I:** Okay, okay. Und wie- wie kam dieser- dieser Wechsel? Weil Sie angefangen haben, Heidegger zu lesen? |
| 317 | **SF03a:** Ja, das auch. Dann habe ich so diese- so über diese Dankbarkeit, die ich empfinden [/empfunden/] habe, nachgedacht, und habe dann so aus den- aus den Strukturmomenten der Dankbarkeit ge- des Dankens gedacht (I: Mh-h (zustimmend)). Da komme ich irgendwie zu Gott. Und auch weil ich jetzt einen Freund habe, der Philosophie und Theologie studiert und der mich dann so ein bisschen da wieder hingeführt hat. |
| 318 | **I:** Der hat sie best- der hat Sie sozusagen auf diese Themen gebracht? |
| 319 | **SF03a:** Ja. |
| 320 | **I:** Mh. Und das heißt, an- am Anfang dieser- dieser Entwicklung der Religiösität stand ein Gefühl? |
| 321 | **SF03a:** Ja, also (I: das metaphysisch) ich wurde eigentlich- ich wurde- ich wurde auch getauft und komuniert (I: Hm (zustimmend)), und so. Und habe dann auch als Kind so geglaubt, bin dann aber auch relativ früh ausgetreten. Und habe eben, wie gesagt, Nietzsche und Heidegger (I: Hm (zustimmend)) äh Nietzsche und Schopenhauer gelesen, die jetzt nicht so viel mit Christentum anfangen konnten (I: Mh-h (zustimmend)). Und habe dann gedacht, ja ich war sehr antireligiös. Und dann- |
| 322 | **I:** Und dieses metaphysische Gefühl der Dankbarkeit hat Sie dazu gebracht, sich nochmal neu Gedanken zu machen, sozusagen? |
| 323 | **SF03a:** Ja, ja. |
| 324 | **I:** Kann man so sagen, ja? |
| 325 | **SF03a:** Ja, kann man (I: okay) so sagen. |
| 326 | **I:** Okay. Und jetzt haben Sie das im Ansatz schon gesagt, aber ähm gibt es Gefühle, dass äußere Ereignisse oder Situationen irgendwie in Beziehung zu Ihnen stehen? |
| 327 | **SF03a:** Beziehungsideen, ja. Also vor allem, wenn es mir halt schlecht ging als psychotisches Symptom. Dass ich irgendwie Gelächter von irgendwelchen Leuten oder Gespräch, das wo ich nicht genau verstehen- das ich nicht verstehen- genau verstehen konnte, gedacht habe, es- die lachen über mich. Die reden (I: Hm (zustimmend)) über mich (I: Hm (zustimmend)) et cetera. Solche Gedanken hatte ich. Oder ein Auto fährt vorbei: Ah, das hat bestimmt auch etwas mit mir zu tun (I: Mh-h (zustimmend)), so (I: Mh-h (zustimmend), okay). Aber jetzt auf einer religiösen Ebene war das nicht. |
| 328 | **I:** Das war nicht, okay. Und wie ähm wie diese- diese Bezugsgedanken (SF03a: Ja) ähm entwickeln die sich? Verändern die sich auch? Sind die- werden die mal stärker, mal weniger stark? |
| 329 | **SF03a:** Also, die sind dann auch wieder komplett weg, wenn eben die (I: Ja.) Psychose remittiert ist, (I: Okay.) wie jetzt seit zwei Wochen (I: Okay.). Oder (I: Dann-) seit zwei, drei Wochen jetzt mittlerweile. |
| 330 | **I:** Ja, ja. Also das heißt, in diesen Situationen ähm haben Sie einfach das Gefühl, manche Dinge beziehen sich auf Sie (SF03a: Ja). Aber nicht das Gefühl, Sie sind irgendwie Zentrum der Welt? |
| 331 | **SF03a:** Nein, das nicht. |
| 332 | **I:** Zentrum der Welt? |
| 333 | **SF03a:** So eine Egomanie habe ich nicht. |
| 334 | **I:** [LACHT] jetzt ohne das zu werten, ne? Das ist- (SF03a: ja) kann auch ein Gefühl sein, was einen- was einen belastet, was man gar nicht gut findet, ne? |
| 335 | **SF03a:** Ja, natürlich. |
| 336 | **I:** Muss ja nicht unbedingt sein, dass man denkt, man ist dadurch irgendwie besser als andere Leute oder so. |
| 337 | **SF03a:** Nein, das habe ich nicht. |
| 338 | **I:** Okay. Und ähm aber dieses Gefühl, Sie würden die Dinge besser verstehen? Oder könn- könnten- wissen mehr, als andere Leute? Haben Sie das manchmal? |
| 339 | **SF03a:** Das glauben auch manchmal andere Leute [LACHT]. |
| 340 | **I:** Ja, klar, wenn Sie denken, Sie sind Genie, oder- |
| 341 | **SF03a:** Nein, da- nein (I: Das-), Genie habe ich nie auf mich selbst angewendet. Sondern, das haben immer nur andere gesagt. Den Begriff finde ich auch ganz schwierig (I: Hm (zustimmend)). Weil so die Vorstellung damit verbunden ist, dass man, wenn man begabt ist, gar nichts tun muss und einem alles zufällt (I: ja). Aber oft sind die Leute, die Genies genannt werden, auch- arbeiten sehr hart an irgendwas. |
| 342 | **I:** Ja, natürlich (SF03a: Also [LACHT]). Naja, äh für mich bedeutet Genie ähm auch einfach diese- diese Flexibilität im Kopf, dass man es hinbekommt, einen Gedanken zu haben, den noch keiner vorher hatte. Oder (SF03a: mh) andere, dass dann damit harte Arbeit verbunden ist- |
| 343 | **SF03a:** Also so, ja, also. Ich würde schon sagen, dass ich mental, kognitiv und von der Bildung überdurchschnittlich bin (I: Mh-h (zustimmend)). Aber- ich meine, ich studiere relativ erfolgreich [ANONYMISIERT, STUDIENFACH] [LACHT] (I: Ja.). Habe ein bisschen Wissen über Philosophie und so. Also ich glaube jetzt nicht, dass das ganz unberechtigt ist [LACHT] (I: Ja.), dieser Glaube. |
| 344 | **I:** Das würde ich auch nicht sagen. Okay. Und das ist- Sie machen das fest, an- an Ihren- an den Dingen, die Sie- die Sie schon getan haben, ne? |
| 345 | **SF03a:** Die ich wissen, wie andere auf mich reagieren (I: Ja, ja). Also man bekommt dann ja auch Feedback (I: Ja, ja), sei es von Professoren oder von Kommilitonen (I: Ja.) und oder von Mitpatienten [LACHT]. |
| 346 | **I:** Okay. Das heißt, damit geht nicht einher, das Gefühl, man hätte irgendwie besondere Kräfte, besondere- |
| 347 | **SF03a:** Nein, also ich habe keine psych- keine telepathischen Kräfte (I: Okay.) oder Telekinese oder so etwas kann ich nicht [LACHT]. |
| 348 | **I:** Okay [LACHT]. Okay noch ganz kurz zu diesem Gefühl des Gläsernen (SF03a: Ja), das hatte ich vergessen. Da würde (SF03a: okay) ich gleich nochmal kurz darüber sprechen. Ähm. Kann man sagen, dass da das Gefühl auch beinhaltet, dass Ihre Grenze so ein bisschen verloren geht? |
| 349 | **SF03a:** Ja. Also, (I: in dem Moment?) die Grenze zwischen Ich und Welt. Die verschwimmt dann so (I: Mh-h (zustimmend)). Also, wie ich es in dem Gedicht geschrieben habe über die Psychose, was da tief in mir schlummert ist jetzt in den Dingen erwacht [LACHT]. Also das ist natürlich sehr poetisch zugespitzt (I: genau) aber (I: das) so ein bisschen verschwimmt dann die Grenze, schon, ja. |
| 350 | **I:** Ja. Ich interpretiere das jetzt mal. Ich sage einfach (SF03a: ja) mal, was ich aus diesen Zeilen lese, (SF03a: ja), ja? Ähm. Das heißt das Innerste in Ihnen, was Sie eigentlich gar nicht nach außen bringen wollen (SF03a: Ja), vielleicht auch, ist in den Dingen erwacht, das heißt, auch nicht unbedingt in anderen Menschen, sondern in den- in der Umwelt? In den Dingen? |
| 351 | **SF03a:** Ja. |
| 352 | **I:** Können Sie mir erklären, was "die Dinge" (SF03a: [LACHT LEISE]) bedeutet? Oder ist es einfach eine Metapher, die passt für Sie? |
| 353 | **SF03a:** Das hat auch ins [LACHT] Versmaß gepasst. |
| 354 | **I:** Das hat gut ins Versmaß- okay. (SF03a und I: [LACHEN]). Also man kann es nicht so eins zu eins verstehen? |
| 355 | **SF03a:** Nein, also. Also, ja, ich würde schon sagen, das einfach so dann- die Gedanken, sie haben sich nicht ausgedehnt, wie Sie vorher gesagt haben, aber sie sind irgendwie auch also- in der Welt so ein bisschen. Ich weiß nicht, wie ich das beschreiben soll, das ist jetzt schwierig, das zu artikulieren. Aber ich hatte ja schon über die Assoziation- den Assoziationsgrenzwert (I: Hm (zustimmend)) gesprochen, oder so. Aber da ist dann auch manchmal so, dass eben- dass einem Dinge in der Welt dann wie eine- einen anderen Gedanken hervorruft, wie es viell- einen Gedanken hervorruft, wie er eigentlich vielleicht nur im größeren Gedankengang kommen könnte. Also dass so ein bisschen die Grenze vielleicht zwischen sinnlicher Wahrnehmung und Kognition auch verschwimmt. |
| 356 | **I:** Mh. Das heißt, plötzlich treten innere Gedanken und äußere Ereignisse in Beziehung (SF03a: Ja) miteinander. Mh-h. Oder ist das zu vereinfacht? |
| 357 | **SF03a:** Nö, das ist nicht vereinfacht. Das trifft es eigentlich ganz gut (I: Ja? Hm). Eine andere Zeile aus dem Gedicht- das kann ich Ihnen auch noch geben, ich habe es jetzt nur nicht im Kopf, "irgendwie ist ahm die Welt nur ein Spiegel für eigenes Denken". |
| 358 | **I:** Okay. Okay. Mh. Das heißt, Ihre Gedanken beeinflussen die Welt auch? |
| 359 | **SF03a:** Was ich von der- die beeinflussen, was ich von der Welt wahrnehme. Also ich würde jetzt nicht- was ich so- was ich so wahrnehme, unbedingt gleich als das Ding an sich, dass da hinter (I: Mh (zustimmend)) der Wahrnehmung (I: Mh (zustimmend)) ist, würde ich nicht identifizieren. |
| 360 | **I:** Okay. Okay, verstehe. Mh. Und bezieht sich dieses- dieses Gefühl der fehlenden Grenze, der Durchsichtigkeit (SF03a: Ja), bezieht sich das nur auf die Welt? Also auf eigentlich allgemein die Umgebung (SF03a: Ja) oder auch auf soziale Interaktionen? |
| 361 | **SF03a:** Also erstmal auf die Welt. Also einfach nicht unbedingt die Tatsache, dass ich durchschaubar- äh dass mich jemand durchschaut, sondern dass ich durchschaubar bin. (I: Grundsätzlich) weil das Gefühl- aber natürlich, wenn man durchschaubar ist, dann kann man auch durchschaut werden. Also- aber das war dann nur in als Folge daraus (I: Okay.), würde ich sagen. |
| 362 | **I:** Also das- das Zentrale für Sie ist das Gefühl (SF03a: Ja, das-), das grundsätzliche? |
| 363 | **SF03a:** Ja, das Gefühl durchschaubar sein, also die Möglichkeit durchschaut zu werden, und nicht unbedingt tatsächlich durchschaut zu werden. |
| 364 | **I:** Mh-h. Also bezieht sich jetzt nicht auf bestimmte Personen (SF03a: nein), sondern es ist ein grundsätzliches (SF03a: ja), grund- okay. Und mh. Kann damit auch einhergehen, dass Sie mal das Gefühl haben irgendwie zu verschmelzen mit einer anderen Person und dann nicht so richtig sich abgrenzen zu können? |
| 365 | **SF03a:** Nein, das kenne ich nicht. |
| 366 | **I:** Nein. Und ähm wie ist es mit Körperkontakt? |
| 367 | **SF03a:** Was ist damit? |
| 368 | **I:** Ähm. Kann der unangenehm sein? Gab es Situationen, wo der unangenehm war? Weil- |
| 369 | **SF03a:** Vielleicht in einem überfüllten Bus [LACHT]. |
| 370 | **I:** Ja. Ähm, ja. Wenn es heiß ist und (SF03a: ja) andere schwitzen und man berührt sich (SF03a: ja). Ähm. Ich meine eher so vielleicht auch im Zusammenhang mit diesem Fehlende-Grenze-Gefühl? (SF03a: Mh) Dass- dass man das Gefühl hat, wenn jemanden- einen berü- einer jemanden berü- berührt einen? |
| 371 | **SF03a:** Achso, dass man dann verschmilzt? |
| 372 | **I:** Dass man- ja. Oder dass dann einem das unangenehm ist, weil die Leute in einen Eindringen, irgendwie nicht (SF03a: nein, also) die Grenzen wahren. |
| 373 | **SF03a:** Also dieses Gefühl des Gläsernseins ist dann eher vereinzelt und eher, wenn ich alleine unterwegs bin und (I: Okay.) da berührt einen dann auch niemand (I: Okay.), also. Kann ich schlecht sagen. Also ich weiß nicht, was ich- wie sich das anfühlen würde, wenn ich im Moment des Gläsernen- im Moment des Gläsernsichfühlen, mich jemand berühren würde. Das kann ich jetzt nicht sagen, weil es irgendwie noch nicht vorgekommen ist. |
| 374 | **I:** Mh. Okay. Und dass Sie mal das Gefühl hatten, unabhängig von dem Gläsernsein (SF03a: ja), dass Körperkontakt unangenehm war, weil Sie das Gefühl hatten, da löst sich irgendeine Grenze auf? Oder jemand überschreitet die Grenze in dem Moment? Das ist auch nicht vorgekommen? |
| 375 | **SF03a:** Nee. |
| 376 | **I:** Okay. Gut, ich denke ähm. Damit haben wir alle meine Fragen geklärt. Ich habe eine Abschlussfrage. Aber erst wüsste ich gerne, ob Sie noch Fragen haben. Oder vielleicht auch irgendwas, was Sie noch nicht gesagt haben? |
| 377 | **SF03a:** Wollen Sie das Gedicht alle acht Zeilen hören [LACHT]? |
| 378 | **I:** Sehr gern. |
| 379 | **SF03a:** Ich habe es auf dem Handy. |
| 380 | **I:** Ja. Lesen Sie es vor. |
| 381 | **SF03a:** Moment ich habe die Systemsprache auf Italienisch umgestellt (I: [LACHT]). Hoffentlich finde ich es jetzt [PAUSE] (undeutlich) suchen. Muss sagen, dass ich auch in dem Gedicht nicht nur eigenes Erleben beschrieben habe, sondern auch so, was ich so über das Symptom über Psychose gehört habe. Einfach, weil es mir um das- die Psychose ging. Also da kommen jetzt auch Stimmenhören vor, obwohl ich nie Halluzinationen hatte. |
| 382 | **I:** Sie haben nie fremde Stimmen gehört? |
| 383 | **SF03a:** Nee. |
| 384 | **I:** Mh. |
| 385 | **SF03a:** Entschildigung, das dauert jetzt irgendwie ein bisschen. |
| 386 | **I:** Das ist kein Problem. [PAUSE] |
| 387 | **SF03a:** Wo ist es denn? Schon so lange her, dass ich das Gedicht habe jemanden? Okay. Ich weiß nicht, ob ich es- ich habe es auf Station, auf dem Laptop, da habe ich es sicher. |
| 388 | **I:** Können Sie mir auch per E-Mail schicken. |
| 389 | **SF03a:** Ich schicke es Ihnen per Mail (I: genau), einfach die Datei. |
| 390 | **I:** Können Sie auch machen. Wenn Sie es- wenn Sie es rezitieren wollen, dann gerne. Aber wenn es nicht geht, auch kein Problem. |
| 391 | **SF03a:** Ähm. Ich kann es jetzt nicht auswendig. |
| 392 | **I:** Okay. Okay. Dann stelle ich meine letzte Frage? |
| 393 | **SF03a:** Ja. |
| 394 | **I:** Außer, Sie haben noch andere Sachen, die Sie gerne? |
| 395 | **SF03a:** Nee. |
| 396 | **I:** Nee? Und das ist auch jederzeit, wenn Ihnen etwas einfällt, können Sie sich melden, können wir schreiben (SF03a: okay), ne? |
| 397 | **SF03a:** Ja. |
| 398 | **I:** Und wir sehen uns ja eh nochmal (SF03a: ja) zum Bewegungslabortermin und so (SF03a: ja). Also, als allerletztes frage ich immer: Sie als Experte für Ihre Erfahrung und Ihre Erkrankung (SF03a: ja), ne? Ähm. Ich würde Ihnen gerne die Forschungsfrage sagen und Sie darauf eine Antwort geben lassen? |
| 399 | **SF03a:** Ja. |
| 400 | **I:** Ja? |
| 401 | **SF03a:** Okay. |
| 402 | **I:** Ähm. Die Forschungsfrage bezieht sich auf Schizophrenie und Bewegung (SF03a: Mh-h (zustimmend)). Ähm die Frage ist: Wirkt sich die Erkrankung auf die Bewegung aus und wenn ja, in welcher Form? |
| 403 | **SF03a:** Mh. Ob es sich immer auswirkt, also auch wenn es remittiert ist, weiß ich nicht, kann ich nichts dazu sagen. In der akuten Erkrankung würde ich sagen, wirkt es sich auf die Bewegungen aus. Weil man- aber ich [LACHT] dann eben auch so- weil irgendwie das Körperempfinden eben so gestört ist. Also ich habe ja schon gesagt, die Asssoziationsschwelle ist so niedrig und man sagt ja auch manchmal: Schizophrenie ist eine Wahrnehmungsstörung (I: Mh-h (zustimmend)). Und zur Wahrnehmung gehört natürlich auch die Selbstwahrnehmungen. Aber wenn man den Körper selbst irgendwie nicht normal wahrnimmt, dann denke ich, bewegt man sich nicht normal. So würd- und das heißt, ich würde sagen vielleicht, wenn es remittiert ist: Keine Ahnung. In der akuten Phase: Ja. |
| 404 | **I:** Und wenn Sie sagen: Nicht normal wahrnehmen (SF03a: Ja). Den eigenen Körper nicht normal wahrnehmen (SF03a: Mh). Was- was assoziieren Sie damit? Was- |
| 405 | **SF03a:** Ja also entweder, dass man den Kö- eigenen Körper gar nicht wahrnimmt, dass man einfach nur bei irgendwelchen Fantasien ist (I: Mh (zustimmend)). Und so- so auf der Hut vor Sachen, dass man gar nicht mehr den Körper wahrnimmt. Oder eben dieses Sichvergewissern, dass man noch im richtigen Körper steckt (I: Mh (zustimmend)). Dass man sich nicht ganz sicher ist, ob das passt (I: Okay.). Oder ich sage immer: Man- ersetze man durch ich. |
| 406 | **I:** Okay. Dass man so, wie aus- was rausfällt (SF03a: ja), aus dem Körper? Mh. Okay. Dankeschön. Vielen Dank. Dann mache ich das jetzt erstmal wieder aus. |

3 **Final categories of the qualitative analysis by second author DM**

**Table 2:** *Overview of the major dimensions of self-experience and body experience*

| **Categories** | **Brief definition** | **Detailed description/coding examples** | **Authors** | **Examplary quotes from the test subjects** |
| --- | --- | --- | --- | --- |
| *Major dimensions of self-experience and possible aberrations* | | | | |
| **Feeling of self-security** | A usual sense of being (existence). It involves automatic unreflected self-presence and immersion in the world (natural, automatic, self-evident). | “The inherent ‘mineness’ of all my experiences, i.e. the structure of experiencing is subjective. This phenomenological concept of presence implies that in our everyday transactions with the world, the sense of self and sense of immersion in the world are inseparable” (de Haan & Fuchs, 2010).“In a normal experience, there is no experiential distance between the sense of self and the experiencing. The basic self-presence is normally presupposed in experience; in itself, it does not possess specific experiential qualities” (Parnas et al., 2005). This unreflected immersion consists of two interdependent components:   (a) Unreflected self-presence; self-awareness; intact first-person perspective; ‘transparency’ or ‘clarity’ of consciousness, intact ‘mineness’ of experience. (b) Unreflected presence/immersion/embeddedness in the world.   Coding examples:   1. The person never questions the ipseity of own thoughts, perceptions, feelings, or pains. 2. The person is directly (noninferentially) conscious of own thoughts, perceptions, feelings, or pains. These appear in a first-person mode of givenness that immediately reveals them as their own. 3. The person feels that she/he is part of the world. 4. The person denies to think a lot about her/himself. 5. The person feels fully alert, fully awake, fully conscious (clarity and transparency of consciousness). | (de Haan & Fuchs, 2021; Parnas et al., 2005) | - “I always knew that I was myself.” - “I would say I am an interested person. Interested in some things and I also find that friends and family define me, so to speak. […] So I would say, I am SF03a [anonymized], an interested person who has already experienced various things. Who lives in a certain social framework.” |
| **Feeling of self-insecurity** | Worrying about being yourself and being a person. | “A pervasive sense of inner void, lack of inner nucleus, a pervasive lack of identity, feelings of being anonymous, as if non-existent or profoundly different from other people” (Parnas et al., 2005). The loss of self-coherence cannot be suppressed; rather, it leads to a constant effort to somehow collect oneself and to form a meaningful whole from the individual parts.  This category also includes a subjective feeling of ‘overadaptation’.  Lack of basic self may also be associated with a pervasively negative self-image.  Coding examples:   1. The person experiences her/himself as being fragmented into parts; He/she does not experience her/himself as a whole. 2. The person describes differences in the worldview, being linked to an existential orientation that is fundamentally different than that of fellow humans. 3. Outwardly, the person describes difficulties in coming into contact with others; she/he feels fundamentally “different“ as if being an observer or outsider in the world. 4. The person describes a necessity to accommodate to the others’ opinion or their point of view, linked to a dominating feeling of not having one’s own inner standpoint (‘innere Haltung’; ‘Haltlosigkeit’). 5. The person describes a negative self-image, e.g. as a sort of eternal ‘shame’, ‘sense of inferiority’ (i.e. devoid of a comprehensible relation to concrete contexts). 6. The person describes feelings of being somehow too open to the world, of having a too ‘thin skin’. 7. The person expresses that they do no longer know who they are, that they doubt if they are the same person, or even a person at all. | (de Haan & Fuchs, 2010, 2021; Parnas et al., 2005) | - “Because I’ve been [taking] Leponex since I was 19 […] I don’t know what I am like in reality.” - “My thinking and my behavior are very different from people who are not so sick. […] I can’t at all say whether I feel this [the thoughts] normally, or whether it is already, uh, abnormal, because it was so long ago that I wasn’t sick that I forgot how it should be. And now I don’t really know whether that’s right, or isn’t that right?” - “Yeah, that [the alienation] sounds weird. You’re right. Now that I hear it like that myself, I’ve never seen it like that. So that sounds really weird. So my thoughts are clearly mine, because I think them. But I don’t have the feeling that it’s just mine. So, strange, kind of weird. […] Something is added to the normal structure of thoughts.” |
| **Feeling of anxiety** | It includes panic attacks, psychic-mental anxiety, phobic anxiety, social anxiety, diffuse anxiety, and paranoid anxiety. | - Panic attacks include severe anxiety with autonomous symptoms. - Psychic-mental anxiety is a strictly mental feeling of anxiety without autonomous symptoms. - Phobic anxiety is provoked by specific stimuli excluding social stimuli. - Social anxiety, provoked by social encounters. - Diffuse anxiety is an inner tension which is provoked by a multitude of stimuli and arises for no reason. - Paranoid anxiety is linked to paranoid ideation.   Coding examples:   1. The person describes fear of height. 2. The person experiences fear due to the presence of other people. | (Parnas et al., 2005) | - “I’m a little afraid of it, of everything. Because when I think about where it all began, slowly creeping up over the years, it got worse and worse. And I’m afraid that at 40, 50 or so I’ll be totally crazy or something. Or say loudly strange things on the train, completely being out of it. That’s what I’m afraid of.” - “I just watch television and suddenly I think: A grenade is about to come in. And then I think again: What nonsense. And then I keep watching television.” |
| **Distorted first-person perspective** | Thoughts, perceptions, or feelings appear as deprived of the tag of mineness. | The distorted first-person perspective comprises:   (a) A decreased or temporally delayed sense of mineness or decreased sense of subjecthood (of being a human subject). (b) A pervasive phenomenological distance between the self and experiencing.   Coding examples:   1. The person describes that their thoughts, perceptions, or feelings are not always their own, anonymous, or mechanically performed. 2. The person describes that they perceive the ipseity with a time delay. 3. The person describes experiences of a lacking relation between themselves and what they are thinking. | (Parnas et al., 2005) | - “Well, for me it is like that when I think, then I hear a voice inside of me. So not acoustically loud, but, even when I read, it is as if someone reads something to me.” - “For example, what I said when I stood at the door on the train, and someone behind me laughed [and I thought] he laughed at me then that’s a thought that belongs to the disease, which is just not mine.” |
| **Feeling of being sovereign** | The feeling of being free or acting freely, autonomously, and independently. | Coding examples:   1. The person describes themselves to be acting freely without being controlled by any external or internal circumstances, e.g. other persons or cognitions. |  | - “I have to say that I’m proud that I can talk like that today. Because two years ago, if you had asked: Are you sick? I would have said: no?” - “I can do everything outside.” |
| **Feeling of loss of control** | The feeling of being dependent or to lose control. | Coding examples:   1. The person describes that she/he said something completely different from what was originally intended. |  | - “Well, actually, I just want the normal thing, but I always fail. Family, work, being reasonably healthy, no drugs, everything just normal. Yes, but hold on trying to achieve that, I have failed time and again for years.” - “The thoughts don’t stop. I can’t stop thinking […] It is such a rush of thoughts, such a storm. […] I can no longer filter properly. I can no longer properly classify the thoughts in the right meaning. […] When the thoughts flow in on you, then I would say the filter is broken. There is a processor on the computer, a task scheduler, which assigns to the various processes what is more important, what has to be carried out first. This is then broken in the brain.” |
| **Feeling of being controlled** | The feeling to be controlled by others. | A more severe form of the feeling of being controlled that includes externalization.  Coding examples:   1. The person describes the feeling that somebody or something else controls their actions, thoughts and/or emotions. |  |  |
| **Feeling of being present** | The feeling of being part of the world or being present. | The ability to become affected, incited, moved, motivated, drawn, influenced, touched, attracted or stimulated by objects, people, events and states of affairs.  Note: While the categories “Bodily estrangement” and “Morbid objectivization” are related to the person’s experience towards themselves, this one is related to the person’s experience towards the world or their surroundings.  Coding examples:   1. The person reports to feel present in the world. 2. The person reports a sense of being affected by the external world. | (Parnas et al., 2005) | - “I’ve already felt myself in the world. There was a being-in-the-world.” |
| **Feeling of being absent/not present** | The feeling of not fully participating in the world or not being entirely present in the world. | A decreased ability to become affected, incited, moved, motivated, drawn, influenced, touched, attracted or stimulated by objects, people, events and states of affairs.  Note: While the categories “bodily estrangement” and “Thingness/mechanization” are related to the person’s experience towards themselves, this one is related to the person’s experience towards the world or their surroundings.  Coding examples:   1. The person perceives a distance to the world. 2. The person complains to not be properly present in the world. 3. The person perceives a sense of not being affected by the external world, a lack of resonance, lack of natural and spontaneous engagement, impossibility of immersion. 4. The person senses that everything is or seems meaningless. 5. The person describes a feeling of being enclosed in a ‘glass case’ or being behind a glass. 6. The Person describes that colors are faded. | (Parnas et al., 2005) | - “Everything works, but it works like a movie, where it’s totally passed out, somehow totally second row, like that.” - “That corresponds a bit to the Cartesian image of man, from Descartes, where you perceive yourself as detached from the world. And just watch. And then the world consists only of things and not of tools.” |
| **Feeling of being lively/vital** | Feeling of vitality, initiative and hedony. | (a) *Vitality.*   A feeling of liveliness, energy, spontaneity, ‘élan’.   (b) *Initiative.*   A feeling that activities can be carried out easily and without great effort. There is no difficulty in initiating actions.   (c) *Hedonia.*   The sensation of pleasure. This includes the ability to experience pleasure in relation to immediately surrounding ‘physical’ perceptual or intellectual stimulation (note: social hedonia is related to the category “Feeling of being present”).  Coding examples:   1. The person perceives her/himself as vital. 2. The person reports activity, energy, or drive. 3. The person reports that they can initiate actions easily. 4. The person describes pleasure (e.g. when tasting food, taking a hot bath, desire for sex, sports, reading books). | (Parnas et al., 2005) | - “I already have a lot of interests. [...] Singing, playing the viola, sign language, tennis, swimming, painting.” - “I am already diligent about some things. So hard work, for example when I’m extremely interested in something. For example, I’m extremely interested in my current job.” |
| **Feeling of being unlively/devitalized** | A diminished vitality, diminished initiative, hypohedonia or (in extreme cases) the feeling of being a liveless, dead object/body. | (a) *Diminished Vitality.*   A pervasive or frequently recurrent sense of inexplicable mental or physical fatigue, dampening of immediate aliveness, diminished energy/spontaneity/élan’.   (b) *Diminished Initiative.*   A pervasive sense that all activity is requiring effort, difficulty in initiating action. In other words, it is not sufficient to score this category on the basis of inactivity, or apathy. The patient must describe his inability to initiate action.   (c) *Hypohedonia.*   Pervasively or recurrently occurring diminished ability to experience pleasure in relation to immediately surrounding ‘physical’ perceptual or intellectual stimulation (note: social anhedonia is related to the category “Feeling of being absent/not present”).   (d) *Feeling of being a liveless, dead body/object.*   A more extreme form of decreased vitality.  Coding examples:   1. The person reports apathy, inactivity or staying in bed. 2. The person describes a feeling of emptiness or a loss of feelings. 3. The person describes a feeling of being tired and exhausted. 4. The person reports excessive preparation times for actions (e.g. sits three hours, preparing himself for going to the post office). 5. The person describes a loss of pleasure (e.g. in tasting food, taking a hot bath, no pleasure from sex, sport, reading books). 6. The person perceives themselves as liveless and in extreme cases as a dead object or body. | (Parnas et al., 2005) | - “I used to postpone a lot. […] I’ve also had phases where I really only spent three weeks in bed and stared at the ceiling.” - “Sometimes I feel an emptiness inside. Before I got to [name of psychiatric department], I wasn’t interested in anything, enjoyed nothing. I just didn’t want to do anything. I just lay in bed doing nothing. […] I was completely disinterested and lacking in drive. […] I even found it difficult to answer the ringing phone next to the bed, which I knew my mother would call to help me. But to pick up the phone myself and then talk, somehow I couldn’t muster the energy for it.” |
| **Hyperreflexivity** | Hyperreflexivity refers to the tendency to exaggeratedly monitor one’s own **sensations, emotions and thoughts**. Patients try to volitionally steer the otherwise tacit processes. | Mild to“occasionally excessive or frequent, even chronic, tendency to take oneself or parts of oneself or aspects of the environment as objects of reflection. The person typically suffers from a loss of naïveté, leniency, and ease. There is an increase in the tendency to reflect about their own thinking or feelings, and inability to react and behave spontaneously and carefree; a tendency to excessively monitoring inner life, while at the same time interacting in the world (‘simultaneous introspection’)” (Parnas et al., 2005).  Coding examples:   1. The person continuously or repetitively questions their own thoughts and what she/he said and did. She/he intensely reviews conversations and own reactions after they happened for example at the end of the day. 2. The person has to think about what to think. 3. The person has to observe social interactions, e.g. whether words or sentences are correct while talking. | (de Haan & Fuchs, 2010, 2021; Parnas et al., 2005) | - “I have no rest with my thoughts. Many people, I think, can sometimes relax, just think about nothing for a moment or only simple things that sometimes fly by a little. And with me it’s always [makes loud electric noises].” - “I find that fascinating, somehow. That I forgot whether this is normal thinking or not. I’m thinking about that right now.” |
| **Hyperautomaticity** | It is the opposite of hyperreflectivity. If the patient succeeds in not to think about what she/he is doing, she/he can surrender to the bodily memory that is still available. | The loss of natural, automatic bodily behaviors can also lead to the opposite of hyperreflectivity. Instead of consciously controlling their own actions, the person then finds that their body performs the movements as if“by itself“. The person itself is no longer involved, not present in their actions. It is not necessarily uncomfortable for those affected to let the body do its thing. Turning off observation and thinking can be a relief, especially if you suffer from hyperreflectivity.  Sometimes used as a coping strategy in order to prevent psychotic breakdowns, patients distract themselves by handing themselves over to manual automatic jobs like cleaning the house, for instance. Hence, there is no modulation, no flexible switching between controlled and uncontrolled action. The observation that ‘almost nothing works naturally’ applies to both hyperreflectivity as well as to hyperautomaticity.  Coding examples:   1. The person experiences his/her body like a machine or a robot. 2. The person describes that she/he does things without even noticing. 3. The person describes that she/he switches off and that she/he does actions automatically. | (de Haan & Fuchs, 2010) |  |
| **Fragmentation of experience** | Stimuli and experiences, which usually tacitly appear to the subject as a unity, are perceived as fragmented. | While reading, a voice is perceived that reads the sentences to the reading person like in an audio book. Due to this parallel perception of reading, the act of reading is perceived by the person much more slowly.  In another case, only the meaning of individual words was correctly and easily recognized, while more complex sentences could no longer be grasped in the sense due to the diverse stimuli. |  | - “There are people who read when they see the letters. And for me it is the case that I actually hear it that way. And not loud, but just as I think. […] When I read something, it goes very slowly for me because I practically hear everything in audio books as if someone were reading everything to me.” - “[To understand] the whole sentence, the context, and then the next one after it, this mass, or something like that, that’s too much.” |
| Mirror-related phenomena | An unusually frequent, and intense looking in the mirror or avoiding one’s specular image or looking only occasionally but perceiving a facial or bodily change. | The patients either perceive changes of their own face or they look for such changes, and therefore examine themselves in the mirror often and/or intensely. They may become surprised or frightened by what they see, and even tend to avoid mirrors because of what they see. Sometimes they look in the mirror to assure themselves of their very existence. They might also look at photos of themselves to find out about their own identity.  Coding examples:   1. The person searches for change or frequently looks in the mirror for a non-specified reason. 2. The person perceives his own face as somehow changed or deformed when looking in the mirror. | (Parnas et al., 2005) | - “And when I got better after the short depression, after the hypomanic period, I had the feeling for a short time, even before I looked in the mirror, who am I actually? Who is there in my body?” Even looking in the mirror could not restore self-coherence: “And then, when I also looked in the mirror, I felt very strange. So when I looked in the mirror, I just felt like an unfamiliar person. So the face that I saw, I thought, who is that? I don’t know him.” - And then, when I also looked in the mirror, I felt very strange. So when I looked in the mirror, I just felt like an unfamiliar person. So the face that I saw, I thought, who is that? I don’t know him.” |
| **Sense of change in relation to chronological age** | A fundamental feeling as if being considerably older or younger than the actual chronological age, not clearly understandable because of social relations or interactions. | Coding examples:   1. The person describes that they feel like a little kid and thus much younger than the actual age. | (Parnas et al., 2005) | - “I have to be honest, I’m 25 and it doesn’t feel like 25 years. [But] like 14 somehow.” |
| **Sense of change in relation to sexual identity and gender** | Occasional fear of being homosexual or that others consider one as such.  A feeling as if being of the opposite sex or a confusion of one’s own sex. | Coding examples:   1. The person reports that they are occasionally unsure of their own gender. 2. The person reports fear of suddenly being perceived as homosexual by other people. | (Parnas et al., 2005) |  |
| *Major dimensions of body experience and possible aberrations* | | | | |
| **Feeling of body agency** | The sense of controlling one’s own body movement. | Body agency is (as well as body ownership) part of a pre-reflective experience of embodied experience. It does not have to be a complex or reflective cognition; it could be simply a matter of a very brief and minimal awareness of initiating and performing a voluntary movement or causing a certain effect in the world.  Coding examples:   1. The person attributes their movement to themselves. 2. The person senses that they are the cause (or ‘author’) of the movement. 3. The person experiences that they are in control of their body or action. | (Tsakiris et al., 2007) |  |
| **Somatic depersonalization/bodily estrangement** | A milder form of estrangement to the body and the movement in comparison to mechanization. | The body or some of its parts are perceived as strange, alien, lifeless, isolated, separated from each other, dislocated or not existing. The person may here experience a kind of unusual introspective access to normally mute body parts or physiological processes.  Note: If the person perceives the body or parts of it as objects or things, the category “Morbid objectivization” must be coded.  Coding examples:   1. The person experiences his internal organs or physiological processes. 2. The person perceives his arms as if they are separated from the body. 3. The person perceives parts of the body as if they do not belong to them. | (Parnas et al., 2005) |  |
| **Morbid objectivization** | A severe estrangement to the body. Experiencing the body or its parts as a physical object, at the expense of the lived bodily experiences. | Morbid objectivization stands for the experiences of an increased degree of “thingness” in the body. The body appears as an object-like entity, as mechanical or even as a robot. Parts of one’s body that are usually silently and implicitly present and at work become spatialized and explicitly experienced.  The estrangement also encompassed a distance between the patients and their own perception. Whereas normally the subject and his experience coincide, here there is no direct perception of and no direct contact with the world. On the contrary, instead of directly seeing something, patients look at images transmitted to them by a camera (not even by their own eyes), and they themselves are far away from the world where these images originated.  Coding examples:   1. The person describes sensations that their body or its parts are experienced as mere things, rather than as living parts of their body, as devoid of life and/or substituted by some kind of mechanism. 2. The person describes that an image of a camera transmits the perceptual data of body parts to them. | (de Haan & Fuchs, 2010; Parnas et al., 2005; Röhricht et al., 2020; Stanghellini et al., 2012, 2014) |  |
| **Loss of body agency** | The sense of losing control of one’s own body movement. | Loss of body agency is the loss of the minimal awareness of initiating and performing a voluntary movement or causing a certain effect in the world.  Coding examples:   1. The person describes that she/he moves unintendedly. 2. The person describes that someone or something else controls her/his movements. 3. The person does not know she/he is controlling their own body. 4. The person does not sense that she/he is the cause (or ‘author’) of the movement. 5. The person experiences that she/he is not in control of their actions. | (Tsakiris et al., 2007) | - “I just have twitching during the day. So there is the bone in the middle and then there are muscles on the outside. And, for example, they twitch briefly and then relax again or something. And something like that on the whole body. Well, it wasn’t always like this, now and then there, then again for half an hour nothing at all. It twitches again briefly, and then again nothing at all. That’s how I have it.” - “If these are my feet now [taps with feet on the floor] and I watch television, for example, then both move at once, like that, over and over again [moves feet]. And then I think again: Huh, why are you doing this? And then I do it again.” |
| **Pseudo-movements of the body** | The experience of pseudo-movements (i.e. not real movements) of the body or parts of it. | Note: Pseudo-movements of the body are not to be confused with motor interference, where real movements occur.  Coding example:   1. The person feels that their legs are moving, but upon closer inspection, they notice that they are not moving. 2. The person describes how they felt parts of their body move, but another person did not notice these movements. | (Parnas et al., 2005) |  |
| **Motor slowdown** | The movement is perceived as being slowed down. | Compared to other people, the person perceives their movements to be significantly slower. |  | - “I move very leisurely.” |
| **Thoughts linked to movements** | Single or repetitive movements related to thoughts. | The person must involuntarily and unwillingly perform a movement as soon as a certain cue appears (i.e. a specific sentence). |  | - “For example, I sit and watch TV, then I think something to myself, I don’t know, “the door is gray“, and then my finger does this [moves finger]” |
| **Motor interferences** | Motor derailments that occur without or against the patient’s intention and typically interfere with intended motor actions or speech. | Such derailments are part of usually intended behavior (pseudo-spontaneous movements, e.g. gaze spasm, movement stereotypes, automatosis syndrome) and are not regarded by the patient as being made or influenced by external forces.  Coding example:   1. The person describes bodily changes (e.g. in posture) that interfere (cave: not prevent) them from moving as intended. 2. The person reports of repeatedly having to perform certain movements, e.g. shaking their head or pulling their shoulders up. These movements take place by themselves without the person doing anything. 3. The person reports to have left the room through a different door than the originally planned one. She/he is not sure where her/his legs are going. | (Gross et al., 1987; Parnas et al., 2005) |  |
| **Motor blocking** | Sudden weakness (paresis), impediment or complete blockage of intended motor actions. | Sensations of paresis might lead to limping or to things slipping out of the hand, to not being able to hold tools so that work has to be stopped.  Motor blocking occurs as a paroxysm. Complete blockages (‘Bannungszustände’) may appear suddenly, like an attack or paroxysm and disappear quickly. The patient is fully conscious, but is unable to move or speak.  Note: These blockages can be regarded as counterparts of the automatosis syndrome.  Coding example:   1. The person describes how suddenly they were paralyzed in the morning and could only move after a few minutes. 2. The person sometimes describes being downright blocked, unable to speak or move. The condition usually lasts a few minutes. | (Gross et al., 1987; Parnas et al., 2005) | - “And then it is often the case that I actually wake up and my hands are asleep, then it tingles or my feet have fallen asleep. [...] and then I can’t move it properly at first.” - “I often wake up at night and can’t breathe.” |
| **Disautomation** | Disautomation refers to the tendency to exaggeratedly monitor one’s own **actions**. Patients try to volitionally steer the otherwise tacit actions. | In action, alienation manifests itself in an increasing disintegration of bodily habits and automatic processes. Common everyday, habitual actions (such as getting dressed, climbing stairs, washing, shaving, brushing the hair) that have been performed more or less automatically in the past cannot be performed anymore or only with great effort of will power great expenditure of time. In many situations the patient is no longer able to carry out a fluid story arc and to use their body in a natural and self-evident way.  Coding examples:   1. The person describes how the daily work no longer goes as usual. They have to think about every step first. 2. The person describes that their daily routine is often interrupted because they have forgotten their habits. The person describes everyday actions require conscious attention. 3. The person has to observe his/her movements. | (de Haan & Fuchs, 2021; Gross et al., 1987; Parnas et al., 2005) | - “Sometimes I found myself having to think about how to tie the knot [from the shoe] now. That I thought to myself: Okay, now you have to think somehow, even though you really don’t have to.” - “The stairs seem different heights to me. As if the levels are different, as if I had to climb higher to overcome the next stair than the other. Strange. And yet, it is the same [height] everywhere. […] All over the world it is the same [the height of the stairs], if you consider how close we go over them. It’s the same everywhere, stairs are always the same, otherwise you would stumble.” |
| **Fluidity of movements** | Fluid movements. | The feeling of fluidity of movements can be perceived as follows:   - Smooth. - Harmonious. - Flawless. - Consistent. - Changing shape easily. |  |  |
| **Rigidness/choppiness** | Rigid and choppy movements. | The feeling of rigidness or choppiness can be perceived as follows:   - Clumsy, cumbersome, inharmonious. - Mechanical, robotic-like. - Choppy or fragmented. - Movements that do not reach the goal correctly. |  | - “How do I experience my movements? Yes, a little awkward at times. That I don’t know how to move properly, like that.” |
| **Feeling of body ownership** | The sense of being the subject of the movement or the body. | Body ownership is (as well as body agency) part of a pre-reflective experience of embodied experience. It is the sense that I am the subject of the movement, that I am the one moving, whether voluntarily or not or that I am experiencing a certain sensation.  Coding examples:   1. The person describes a kinaesthetic experience of movement. 2. The person describes a sensation of a touch. | (Tsakiris et al., 2007) |  |
| **Feeling of lost body ownership** | Disturbances in the sense of being the subject of the movement or the body. | The loss of body ownership is expressed in the sense that I am not the one moving or that I am not the one experiencing a certain sensation. There is no self-ascription that I am the one who is undergoing an experience.  Coding examples:   1. The person describes that they notice a movement, but it is not they who experience that movement. 2. The person describes that they notice a sensation of a touch, but it is not they who experience that touch. | (Gallagher, 2000; Tsakiris et al., 2007) | - “I had thought so completely Cartesian, somehow, body and mind are now totally separate and that was a very strange bodily feeling. […] That was very unusual, like having new shoes that your feet have not yet got used to but related to the whole body. […] That felt very unfamiliar, strange, and wrong. […] I was yet in the body, but yet not. Wrongly, that’s how it felt. […] So the spirit was in the body, but it didn’t quite fit. Like when you try, I don’t know, to put Windows on an Apple computer or something.” |
| **Dysmorphic-like experiences** | Patients experience the form or appearance of their body in transformation. | The dysmorphic-like experiences entail puzzling phenomena of an ongoing change or destructuring in parts of one’s body, especially its form or appearance, or in the body as a whole. The experience may involve the entire organism, or components.  They are usually characterized by:   (a) *Dynamization of bodily boundaries.*   An intrusion or incorporation of extra-personal things, forces and events. Violation typically entails dynamism, that is experiencing something moving into oneself, not merely the static presence in oneself of something that should occupy a position external to the self.   (b) *Dynamization of body construction.*   An experience of body disintegration which involves the shifting around of the usual spatial relationships between body parts, or a dynamic distortion of body Gestalt, i.e. of one’s body as a unitary and integrated structure.   (c) *Externalization.*   The feeling that bodily components, vital energies or biological activities are projected beyond one’s somatic boundaries. Externalization is not a static experience, but it implies movement. Ego and corporeal boundaries, so to say, are violated from within by parts of the body that are felt as expelled into outer space.  Coding examples:   1. The person complains that their body boundaries are being violated by entities or forces coming from without their own bodily boundaries. 2. The person complains that part of their body is moving away from its usual position. 3. The person perceives their body structure being disintegrated. Parts of their body are felt as moving away from their usual position. 4. The person perceives their body as falling apart or going into pieces or disappearing. 5. The person reports perplexing phenomena of unusual, strange movements or forces acting inside one’s body. 6. The person experiences bodily components as moving away from their usual position or shifting around the usual spatial relationships. E.g. their mouth was where hair should be. 7. The person experiences that some of their intestines are half outside. | (Parnas et al., 2005; Stanghellini et al., 2012, 2014) |  |
| **Dysmorphophobia** | Patients experience the form or appearance of their body as ugly or having some physical defect - although their appearance is within normal limit. | Patients are puzzled about/afraid of the loss of the form/appearance of their body because they experience it as ugly or having some physical defect (especially asymmetry or change in proportion) although they appear to others within normal limits. | (Stanghellini et al., 2012, 2014) |  |
| **Psychophysical misfit and psychophysical split** | The body or individual parts of the body are experienced as being isolated and separated from one another or as not being present at all. | The body feels as if not really fitting, as either too small, too big, or otherwise uncomfortable or somehow changed.  Coding examples:   1. The person describes a feeling that their mind and body do not fit or belong together, as if the body does not belong to them. 2. The person describes that sometimes they cannot feel their body. 3. The person describes feeling a void in their head. | (Gross et al., 1987; Parnas et al., 2005) | - “I had thought so completely Cartesian, somehow, body and mind, that are now totally separate and that was a very strange bodily feeling. […] That was very unusual, like having new shoes that your feet have not yet got used to but related to the whole body. […] That felt very unfamiliar, strange, and wrong. […] I was yet in the body, but yet not. Wrongly, that’s how it felt. […] So the spirit was in the body, but it didn’t quite fit. Like when you try, I don’t know, to put Windows on an Apple computer or something.” |
| **Coenesthesias** | Coenesthesias are unusual bodily feelings, which cannot be explained medically. They are also termed body hallucinations. | Coenesthesias can manifest itself in the following sensations:   - Unusual bodily sensations of numbness and stiffness. - Migrating bodily sensations wandering through the body. - Electric bodily sensations, feelings of being electrified. - Bodily sensations of movement, pulling or pressure inside the body or on its surface. - Sensations of abnormal heaviness, lightness or emptiness, of falling or sinking, levitation or elevation affecting the whole body or just parts of it. - Vestibular sensations.   Coding examples:   1. The person describes that their hands, feet or other parts of the body are numb. 2. The person describes that they experience a vibrating or electrical feeling in the head. 3. The person describes a feeling as if her/his legs or the whole body are/is floating. | (de Haan & Fuchs, 2021; Parnas et al., 2005) | - “I have such a strange pressure on my head and it’s kind of weird. That doesn’t hurt or anything! […] You can live with it, but it’s like being mad all the time, yes. […] Just like when you take two plastic bowls and do something like that [makes vibrating noise].” - Sometimes I have, for example, I sit and watch TV, then I think something to myself, I don’t know, the door is gray, and then my finger goes so [moves finger]. |
| **Pain-like experiences** | These are sine materia painful experiences that are lacking a cause ascertainable by means of standard diagnostic procedures. | The core phenomenon in this category is that the patients report unpleasant or painful bodily feelings not substantiated by any medical evaluation; experiences may present themselves in the form of paroxysms or persistent sensations; they are characterized by feelings of strangeness and uncanniness. Sometimes, they are conceived as inflicted.  This category contains the feeling of:   - Heat. - Pain. - Stings. - Being cut. - Dysesthesias provoked by sensory or tactile stimulations.   Coding examples:   1. The person describes inconveniences, i.e. pain that is provoked by an acoustic stimulus or a touch, which feels unpleasant and painful. 2. The person describes unexplainable pain, which occurs without any stimulus. | (Parnas et al., 2005; Stanghellini et al., 2012, 2014) |  |

*Note.* Brief definitions and detailed descriptions are taken from the publications in the authors column. The green font color indicates descriptions of deductively formed categories, while the orange font color indicates descriptions of categories that were set up deductively but were not confirmed in the interviews. The blue font color indicates descriptions that were formed inductively.

5 **Case vignettes of subgroup participants taken from the qualitative analysis by second author DM**

### RM05

#### Self-experience.

RM05 shows signs of *self-security* when he said: “I always knew that I was myself”. However, his predominant *self-insecurity* leads him to wonder for a long time when the changes that he perceives in himself began. To this day he has not found a satisfactory answer. He suspects, however, that his self-doubts marked the beginning of his illness. His self-questioning leads to a feeling of otherness and loneliness [#1]: “I feel clearly different from other people”. Above all, sinking into daydreams for him is a sign of his self-insecurity. In his first job, confronted with stressful situations and work pressure, he specifically remarked [#2]: “Something is wrong with you.” This insecurity existed alongside a conviction that persisted for several years that people in his work environment would speak badly of him and that he would therefore never find a job again.

He perceives *anxiety* when he thinks about the fact that his previous employer would sue him for having worked for a long time with negative symptoms. After the employer had already assured him several times that he had no such ambitions, RM05 describes [#3]: “The thought is completely nonsense, but it comes, and it makes me panic.”

Although he experiences a *feeling of being sovereign* when he described to generally find the right words for what he wants to express and be able to deliberately stop lines of text that he has to repeat over and over again in his mind, the *feeling of loss of control* predominates. His thoughts disappear, fade, and suddenly dissipate. He describes phases in which he could not grasp the content of even a few sentences due to “concentration problems” and “memory difficulties”. This leads to the fact that he sometimes does not know what he was doing shortly before. Then he thinks [#4]: “Oops, how did you get there now [to a certain place]?”. He sometimes has to repeat lines of lyrics of songs over and over again, 20 or 30 times. The thought of being sued by the previous employer persists [#5]: “I can’t get that away [the thought]. […] Come what may.” His sensitivity to environmental stimuli is also a great distraction and prevents him from concentrating. Even the faint squeaking he hears in the background leads to a strong feeling of loss of control [#6]: “[It] sometimes drives me completely crazy and makes me unable to act to a certain extent because I feel too distracted.” Ambivalence in decision-making is also linked to his loss of control. Everyday shopping is very difficult for him as he spends hours choosing what to buy. [#7] “Shopping with me is the killer! […] I find it extremely difficult to make decisions.” He also describes a situation in a schnitzel restaurant where he was overwhelmed by the offer. [#8] “That was hell for me.” In the end, after 30 minutes and out of sheer desperation, he chose a standard schnitzel. This phenomenon, referred to by the patient as “decision poverty”, also happens when choosing and putting on clothes.

RM05 experiences a severe *feeling of being absent/not present* in everyday life. By sinking into thoughts that come automatically and become very lively for him, he appears absent-minded and subsequently has to be actively brought back into the conversation by other people who snap their fingers and say his name several times.

He reports a *feeling of being lively/vital* when he states that he was very interested in search engine optimization and that he had a lot of fun with it. He is also very interested in politics and English. On the other hand, the *feeling of being unlively/devitalized* seems to be more pronounced when he speaks of a lack of initiative [#9]: “I used to postpone a lot. […] I’ve also had phases where I really only spent three weeks in bed and stared at the ceiling.” He says that he has been wanting to buy a new bed for months, keeps looking at brochures, is indecisive and cannot mobilize enough energy to drive to a furniture store. Such decisions cost him a lot of energy. When asked, he estimates that he feels moderately exhausted, burned out and lacking energy.

RM05 experiences *hyperreflexivity* in the form of thoughts to which he is very attached, and which are difficult to resolve. He often worries that his e-mail account could be hacked and often wonders after leaving his apartment whether he has properly closed his windows and doors beforehand. He often asks himself who a certain person was after they looked at him outside.

#### Body experience.

In the domain of *disautomation*, he describes that he experienced the loss of the natural self-givenness in routine actions several times [#10]: “Sometimes I found myself having to think about how to tie the knot [from the shoe]. That I thought to myself: Okay, now you have to think about it somehow, even though you really don’t have to.”

After he was asked how he basically perceives his movements, he states that he moves much more slowly than his fellow human beings [#11]: “I move very leisurely.” Due to this statement, we inductively formed the category *motor slowdown*.

***Unmentioned categories of self-experience.*** RM05 did not describe phenomena addressing the following categories of self-experience: distorted first-person perspective, feeling of being controlled, feeling of being present, hyperautomaticity, mirror-related phenomena, sense of change in relation to chronological age, sense of change in relation to sexual identity and gender, and fragmentation of experience.

#### Unmentioned categories of body-experience.

RM05 did not describe phenomena addressing the following categories of body experience: feeling of body agency, somatic depersonalization/bodily estrangement, morbid objectifization, loss of body agency, pseudo-movements of the body, motor interferences, motor blocking, fluidity of movements, rigidness/choppiness, feeling of body ownership, feeling of lost body ownership, dysmorphic-like experiences, dysmorphophobia, psychophysical misfit and psychophysical split, coenesthesias, pain-like experiences and thought linked to movements.

**IH12**

#### Self-experience.

While IH12 gives no indications of *self-security*, she perceives several aspects of *self-insecurity*. She links her longer illness and, at the time of the interview, 27 years of medication to her self-insecurity [#12]: “Because I’ve been [taking] Leponex since I was 19 […] I don’t know what I am like in reality.” Especially in social situations and during work, she experiences a multitude of problems that have a common basis of insecurity [#13]: “I think I did something wrong at work. And then I keep thinking about it. […] Still, there is something I do wrong every day. […] At least one thing.” From her point of view, her problem is that, due to her insecurity, she too often has to reinsure herself with other people by inquiries. Even with simple decisions, such as which company stamp to use, she is insecure and therefore asks other people for advice. She also experiences insecurity in her leisure activities with her friends. There, she often thinks that she is not good enough at exercising or singing [#14]: “I’m not a leading voice either, I can’t do that at all yet.” As a result, IH12 felt so overwhelmed that she canceled a planned church event and quit her tennis group. Her insecurities also address worries about health: after reading an article in a newspaper that recommended carrying a small jar with you in the event of a fall and a tooth fracture, she immediately bought one. IH12 perceives going to church weekly to be a successful strategy for distancing herself from uncomfortable feelings of insecurity [#15]: “That’s why I always go to church on Sundays, simply because I keep making mistakes, and just to let go of that and know that I’m going to start all over again.”

Her self-insecurity seems to lead to a diverse *feeling of anxiety*. IH12 reports severe anxieties when singing in the church choir in front of people [#16]: “I get so upset when I sing there. I think all the time, I’m going to faint.” Her anxieties also relate to heights, thunderstorms and traveling by bus and train. She once had to get out of the bus when the driver was driving too fast, and she was too afraid of the speed. In another situation, a friend of hers had to take a break while driving to allow IH12 to recover. In these situations, she experiences vegetative symptoms of fear such as increased perspiration, palpitations, and a furry tongue. Even when she walks the streets at night, she feels afraid that someone could attack, threaten or rape her. Her anxieties also relate to close relatives and close work colleagues. Sometimes she suddenly becomes afraid that at some point her parents will no longer be there for her to help with her questions or insecurities.

Furthermore, due to the older age of her boss, she was often worried about him and checked whether he was still alive by listening to his breathing from the next room. She had to ask herself how she should act in a case of emergency [#17]: “What should I do? Dial 112? […] I’m a bit hysterical, you could say.” When her father once sat suspiciously for her, she worried so much that she dialed the emergency number. However, the father was in good health. She describes this herself as“being fearful of loss”. In addition, she is also very concerned about her own health. She controls her body and, because of her concern, goes to the doctor if there are tiny abnormalities, such as a small red dot on the skin.

IH12 reports a *distorted first-person perspective* when she perceives her thoughts as if her mother were reading to her. She also has this feeling when reading herself. She describes [#18]: “For me it is like that when I think, then I hear a voice inside of me. Not acoustically loud, but, even when I read, it is as if someone is reading something to me.”

A *feeling of being sovereign* arises from the various strategies that she uses to reduce insecurities. At the time of the interview, she reports that she can drive the bus again without any major fear, because she always says to herself [#19]: “That’s his job. I also do my job […] and then he will also be able to drive.” Besides, she describes feeling better since she decided to sing in the back of the choir.

Indications for a *feeling of a loss of control* are present in IH12 as short blackouts of a few seconds that she experienced as a teenager in her job as a kindergarten teacher when telling stories [#20]: “[The thoughts were] just gone […] it only lasted for three seconds, then I just looked at the children, or so, and then it came back.” Her ambivalence in decision-making also shows signs of a loss of control [#21]: “Oh, I can’t make up my mind at all. […] Always. The decision of whether to come back from vacation a day earlier or stay a day longer. Or deciding what to wear in the morning. Or at work. Actually, all the time. […] What I should buy, too. […] Should I take a window seat or an aisle seat? […] It’s just too much.” In addition, she experiences some fears so strongly that she has the feeling of losing consciousness. She anticipates this for future events such as in quote #16 and said [#22]: “And then I thought to myself, if I get so upset about one song in the service, how am I supposed to survive a whole concert?” She also reports loss of control over her sensitivity to stimuli. If she perceives stimuli too intense, she can no longer concentrate [#23]: “It starts when I’m on the bus and there are two people sitting behind me talking. I usually do something on the bus. And then I usually sit down somewhere else.” At work, she has to shield herself from stimuli in order to be able to concentrate. She describes her sensitivity to stimuli as follows [#24]: “I was sitting in the train today and suddenly I saw that a light was flickering up there. I sat down somewhere else because it bothered me so much. I then asked the one next to me: Doesn’t that bother you?” For IH12, feelings of a loss of control also result from learning problems, as she has difficulty remembering vocabulary, for example.

In the domain of *feeling of being lively/vital*, IH12 reports diverse interests such as singing, playing the violin, learning sign language, tennis, swimming, painting. In addition, she plans to buy a swimming pool card. Besides, she reports *a feeling of being unlively/devitalized* when she talks about her lack of initiative in the morning [#25]: “When I wake up in the morning, at first I don’t feel like doing anything at all […] I have quite a problem waking up. […] Then I always look forward to the weekend, for example, and then I cannot get out of bed! […] Or in the afternoon, after I lie down, I’ve got no pleasure at all.” She perceives this lack of initiative every day.

IH12 experiences *hyperreflexivity* as a tendency to exaggeratedly monitor own thoughts as follows [#26]: “When I lie in bed at night, the thoughts rattle and rattle through my head. And then I say to myself: You thought that now, this is gone now. And then the next thought comes, and then I think like this: You have now thought that too, get rid of it.” When asked about the content of the thoughts, she replies [#27]: “What I have to do the next day. Or that I think I did something wrong at work. And then I keep thinking about whether that was really wrong.” She also ruminates whether other people are mad at her. A next indication of hyperreflexivity arises from the fact that IH12 needs checklists for both less and more complex workflows. Even if she has gone through all the steps, for example the routine before leaving the office, she often has to re-enter the office to make sure that she has really thought of all the steps. She describes this hyperreflexivity as compulsive checking. Additionally, switching trains is extremely stressful for her because she is afraid of forgetting something. That is why she has to check several times whether she is carrying everything with her. She also asks other fellow travelers several times to reassure herself that she has taken all her luggage with her.

A *fragmentation of experience* results from the fact that IH12 perceives her mother’s voice while reading and thinking [#28]: “There are people who read when they see the letters. And for me it is the case that I actually hear it that way. Not loud, but just as I think. […] When I read something, it goes very slowly for me because I practically hear everything in audio books as if someone were reading everything to me.”

#### Body experience.

IH12 describes body experiences in the domain of *motor blocking*. In the morning after waking up, she often experiences that her hands and feet have fallen asleep, a tingling and that she can no longer move them. That is why a cell phone is always ready by the bed to call for help if necessary. Additionally, she often wakes up at night and then cannot breathe properly. She then takes a deep breath, after which the condition improves.

In the area of *disautomation*, she once experienced a physical blackout while playing a viola that she had practiced for many years [#29]: “I couldn’t get on with my thoughts”.

Some of the experiences described in the section of motor blocking also meet the criteria for *coenesthesias* when she sometimes wakes up and feels a numbness in her arms, hands, and legs.

#### Unmentioned categories of self-experience.

IH12 did not describe phenomena addressing the following categories of self-experience: feeling of self-security, feeling of being controlled, feeling of being present, feeling of being absent/not present, hyperautomaticity, mirror-related phenomena, sense of change in relation to chronological age, sense of change in relation to sexual identity and gender.

#### Unmentioned categories of body-experience.

IH12 did not describe phenomena addressing the following categories of body experience: feeling of body agency, somatic depersonalization/bodily estrangement, morbid objectifization, loss of body agency, pseudo-movements of the body, motor interferences, fluidity of movements, rigidness/choppiness, feeling of body ownership, feeling of lost body ownership, dysmorphic-like experiences, dysmorphophobia, psychophysical misfit and psychophysical split, pain-like experience, motor slowdown, and thought linked to movements.

### AB04

#### Self-experience.

AB04 shows evidence of *self-security* when he reports that he can do everything outside in social contact. He identifies parts of his beliefs and thoughts as normal and denies the question of whether he is insecure of his identity. When asked who he is, AB04 replies [#30]: “I am, uh, a person” and thus shows a self-concept that tends to be less specific. When asked who this person is, he replies that it is a really tough question. This *self-insecurity* presents itself in the course of the conversation as deep uncertainty about what is right or wrong, whether he is right or wrong as a person and which parts of him as a person, including thoughts and perceptions, are healthy or sick [#31]: “My thinking and my behavior are very different from people who are not so sick. […] I can’t at all say whether I feel this [the thoughts] normally, or whether it is already, uh, abnormal, because it was so long ago that I wasn’t sick that I forgot how it should be. And now I don’t really know whether that’s right, or isn’t that right?” The *loss of self-coherence*, about which he seems to gain more experiential insight in the course of the interview, manifested itself in a feeling of an alienation of his own thoughts and a failure to attribute experiences to himself [#32]: “Yeah, that [the alienation] sounds weird. You’re right. Now that I hear it like that myself, I’ve never seen it like that. That sounds really weird. My thoughts are clearly mine because I think them. But I don’t have the feeling that it’s just mine. […] Something is added to the normal structure of thoughts.” He continues [#33]: “I have no rest with my thoughts. Many people, I think, can sometimes relax, just think about nothing for a moment or only think about simple things […] And with me it’s always [makes loud electric noises].” Furthermore, the *loss of the self* presents itself as a profound feeling of worthlessness, which leads to insecurity in behavior and even to a questioning of his right to exist [#34]: “For years I thought I had to die. […] You just have the feeling yourself that you are worth nothing if you are supposed to be killed. […] And then I always thought: May I do that [say something] at all?” This feeling ultimately leads to the feeling of being defenseless when coming into social contact. He explains that he used to be unsettled by stimuli caused by noises and people, for example when he is in the city. Honking of cars is especially aggressive to him and he is often irritated by conversations in his environment (not directed towards him) that force themselves on him [#35]: “I perceive everything”. AB04 sums up being constantly separated from putting his wishes into practice because of his illness [#36]: “There are always plans. For years. Normal life. But I always fail. Hopefully not again this time.”

AB04 reports *feelings of anxiety* when he is afraid that his illness will progressively increase [#36]: “I’m a little afraid of it, of everything. Because when I think about where it all began, slowly creeping up over the years, it got worse and worse. And I’m afraid that at 40, 50 or so I’ll be totally crazy or something. Or say strange things loudly on the train, completely being out of it. That’s what I’m afraid of.” AB04 also describes constant paranoid anxieties [#37]: “I have often asked myself, if I am really kidnapped, what do I do then? Do I defend myself or do I fight? Do I just let it happen? I honestly don’t know it at all.” He perceives these anxieties as life-threatening, but can distance himself from them [#38]: “I just watch television and suddenly I think: A grenade is about to come in. And then I think again: What nonsense. And then I keep watching television.”

He shows evidence of a *distorted first-person perspective* when he describes the lack of ipseity in some thoughts [#39]: “For example, what I said when I stood at the door on the train, and someone behind me laughed [and I thought] he laughed at me then that’s a thought that belongs to the disease. The thought is just not mine.” Additionally, he agreed on sometimes perceiving his thoughts, actions, and feelings as strange and doubting that they belong to him (see quote #39).

Regarding the *feeling of being sovereign*, he reports being proud of having quickly overcome both his drug addiction and his recent psychosis. He feels able to express himself well and also reflects on his introspective insight into illness [#40]: “I have to say that I’m proud that I can talk like that today. Because two years ago, if you had asked: Are you sick? I would have said: no?” Making everyday decisions, such as choosing clothes or shopping, is easy for him. He was even able to cut his daughter’s umbilical cord. His feeling of being sovereign is contrasted with a *feeling of loss of control*. Despite his efforts, he describes that the psychosis had recurred. He also experiences strong concentration and memory problems in everyday life [#41]: “I used to like to play chess. I was also a very good chess player. […] And then we wanted to play chess and he [a friend] beat me, beat me, beat me. I couldn’t concentrate at all. I mean, my concentration has only gone downhill since I was 17 or 18 years old.” When reading texts, he finds it very difficult to grasp the meaning of the content [#42]: “I read a sentence, the second sentence, then I have already forgotten the first one, then I skip one because it is so exhausting for me and so I go through the whole text.” He also experiences a loss of control during conversations due to concentration and memory problems: “I talk to my mom, and in the middle of a sentence it suddenly disappears and then I no longer know what to say.” Loss of control also manifests itself in the domain of thought. He can neither elude nor distance himself from recurring pressured thinking [#43]: “It’s more of a kind that you don’t stop. Thinking, thinking, and thinking. […] That doesn’t necessarily happen quickly, but it never ends.” Several times in the past, he suffered from audible thoughts. The loss of control appeared to be sometimes connected to paranoid anxieties when he thinks that he is more likely going to be killed after talked about the fear of being killed [#44]: “When it comes to topics like this [his anxiety to be kidnapped and killed], I actually never talk about this because I am somehow convinced that things will get worse then. Because when I tell other people how I am feeling and what I am thinking and how it works, so to speak, that it will get worse, because other people will know it then.” He also experiences a loss of control when he does not achieve goals he has set himself [#45]: “Well, actually, I just want the normal thing, but I always fail. Family, work, being reasonably healthy, no drugs, everything just normal. But I have failed time and again for years.”

In addition to a general *feeling of being present* in the world, AB04 also describes the *feeling of being absent/not present*. He confirms the question of whether the world seemed dead and gray to him and added [#46]: “Everything works, but it works like a movie, where it’s totally passed out, somehow totally second row, like that.”

With regard to the *feeling of being lively/vital*, AB04 confirms the question of whether he could easily make everyday decisions when shopping or choosing clothes. This contrasts with a *feeling of being unlively/devitalized:* According to his perception, AB04 has always failed to achieve longer-term goals and confirmed the question of whether he perceived himself to be separated from his goals and wishes. In addition, he described a strong anhedonia [#47]: “The whole time you just had the thought: I have to die, I have to die, I have to die. And then everything somehow passed you by, the whole rest. […] You couldn’t enjoy anything anymore, and so on. […] I didn’t take a shower for weeks, I didn’t go out, and everything. Nothing at all.” His previously presented feeling of lack of presence in quote #46 nevertheless leads to a state of anhedonia.

*Hyperreflexivity* as a tendency to exaggeratedly monitor own sensations, emotions and thoughts initially presents itself as a compulsion to brood over the course of the day and in the course of increasing exposure to stimuli [#48]: “I can concentrate best in the morning. […] And when I’ve experienced the entire day, then it’s always worst in the evening.” In the evenings he lies in bed and thinks about situations in which, for example, someone laughed behind him and he thought that person was laughing at him. This increased brooding happens unintentionally [#49]: “I don’t go to bed and think: now you’re thinking about today again. Not like that, it comes automatically.” The pressure he experiences through his thoughts is even evident as an electrical feeling or pressure that impacts on his head as mentioned in quote #33. He explains that he first thought every patient with schizophrenia experiences this sort of pressure. After reporting his sensation to another patient, he was surprised that he did not experience this phenomenon. He also reflects intensively on various environmental problems and in this process gains a high level of abstraction, which, however, sometimes appears qualitatively alienated [#50]: “That [the plate, as an example] is the earth, our planet, and the cookies [on it] are resources for our lives […] and we take ourselves from them and at some point we fall down [from the plate].” In addition to pondering, he also tries to steer otherwise tacit processes. He asks himself [#51]: “Why do we have money and not exchange [things]? […] With the carbon dioxide [in mineral water]: why does it bubble?” AB04 also took the height of the stairs as an object of reflection [#52]: “All over the world it is the same [the height of the stairs], if you consider how close we go over them. It’s the same everywhere, stairs are always the same, otherwise you would stumble.” Besides, when standing at traffic lights, he reflects [#53]: “I stand there and then I don’t know how to stand. And then someone starts walking and then I start walking too and then I think: Mh, the other person is long gone while I still think about the traffic lights.” In the course of the conversation, this reflective meta-perspective itself becomes an object of reflection [#54]: “I find that fascinating, somehow. That I forgot whether this is normal thinking or not. I’m thinking about that right now.”

Furthermore, he experiences *changes in relation to chronological age*. He is 25 years old at the time of the interview but feels more like 14 years old.

AB04 describes that he understands individual words easily and without problems, but with regard to longer sentences we inductively found a *fragmentation of experiences* [#55]: “[To understand] the whole sentence, the context, and then the next one after it, this mass, or something like that, that’s too much.”

#### Body experience.

In the domain of *loss of body agency*, he experienced muscle twitches downwards the neck [#56]: “I have twitching during the day. So there is the bone in the middle and then there are muscles on the outside. And, for example, they twitch briefly and then relax again. And something like that on the whole body. Well, it wasn’t always like this, now and then there, then again for half an hour nothing at all. It twitches again briefly, and then again nothing at all. That’s how I have it.” Besides, he experiences sudden involuntary movements of limbs [#57]: “If these are my feet now [taps with feet on the floor] and I watch television, for example, then both move at once, like that, over and over again [moves feet]. And then I think again: Huh, why are you doing this? And then I do it again.” These movements sometimes have a meaning of are connected to thoughts as described in the respective category.

The loss of the tacit dimensions with the tendency to exaggeratedly monitor own actions leads to *disautomation*. He explains that he is no longer able to climb stairs automatically as indicated above [#58]: “The stairs seem different heights to me. As if the levels are different, as if I had to climb higher to overcome the next stair than the other. Strange. And yet, it is the same [height] everywhere. […] All over the world it is the same [the height of the stairs], if you consider how close we go over them. It’s the same everywhere, stairs are always the same, otherwise you would stumble.” He also experiences disautomation of movements in other states of hyperreflexivity, for example when standing at traffic lights as indicated in quote #53.

AB04 experiences *coenesthesias* in the form of a dull feeling of pressure on the head [#59]: “I have such a strange pressure on my head and it’s kind of weird. That doesn’t hurt or anything! […] You can live with it, but it’s like being mad all the time, yes. […] Just like when you take two plastic bowls and do something like that [makes vibrating noise].” He has even asked other patients about this and found that they do not have the feeling.

Lastly, he described *thoughts linked to movements* [#60]: “Sometimes, for example, I sit and watch TV, then I think something to myself, I don’t know, the door is gray, and then my finger goes so [moves finger].”

#### Unmentioned categories of self-experience.

AB04 did not describe phenomena addressing the following categories of self-experience: feeling of being controlled, hyperautomaticity, mirror-related phenomena, sense of change in relation to sexual identity and gender.

#### Unmentioned categories of body-experience.

AB04 did not describe phenomena addressing the following categories of body experience: feeling of body agency, somatic depersonalization/bodily estrangement, morbid objectifization, pseudo-movements of the body, motor interferences, motor blocking, fluidity of movements, rigidness/choppiness, feeling of body ownership, feeling of lost body ownership, dysmorphic-like experiences, dysmorphophobia, psychophysical misfit and psychophysical split, pain-like experiences, and motor slowdown.

### SF03a

#### Self-experience.

When asked who he is, SF03a responds with an established self-concept and thus shows signs of *self-security*. He answers [#61]: “I would say I am an interested person. Interested in some things and I also find that friends and family define me, so to speak. […] So I would say, I am SF03a [anonymized], an interested person who has already experienced various things. Who lives in a certain social framework.” In addition, he perceives his thoughts, actions and feelings as belonging to him and not alien. He confirms that he identifies with his thoughts. This is contrasted by a *feeling of self-insecurity*, which he describes mainly through phenomena during his school days. After he managed to switch from Realschule to Gymnasium, which he wished for a long time but remained unexpectedly unhappy, he realized for the first time that something was wrong with himself [#62]: “Before that, I could blame everything on the fact that I wasn’t challenged enough. And when I’m not happy now in high school, then it [the problem] must be myself. That was a bit of my reason why I then developed something like this [the disease].” Subsequently, in his youth, he had the feeling that others did not really like him and that he had no friends. He describes that he had friends objectively, but he felt“exotic”. The social contact felt strange to him [#63]: “I actually made quite good social connections, but somehow it didn’t feel that real.” Basic self-doubts revealed themselves in the social contact too [#64]: “I already had the feeling that I felt friendship for them, so to speak, but I wasn’t sure whether they also see me as a friend.” These self-doubts manifest themselves in reports of being very strict and resentful towards himself. He thought to himself [#65]: “You are correct in assuming that you are not worthy of being their friend.” In the course of his youth, he wished for a very close friendship, but he always had the feeling that he was doing something wrong. As a result of the increased self-doubts and the feeling of not being part of a social network, he has withdrawn greatly. Over a longer period of time, he then dealt with the hikikomori trend, in which young people do not leave their own rooms for years and do not have any employment. He had anticipated that he would become that way and subsequently developed persecutory delusions. Even today he still suffers from the self-doubt as part of self-insecurity [#66]: “Sometimes I think of totally unimportant situations from eight years ago, where I behaved strangely and thought maybe I should have said goodbye differently or something. So often very, very banal. Then I just reproach myself and take myself to court, so to speak. But not in the way that I say I have to do better now, but more like I have to punish myself somehow. […] Punish in the sense that I don’t appreciate myself.”

SF03a reports [#67]: “I would say that I am mentally, cognitively and educated above average” which helped him to successfully study mathematics and to gain an understanding of philosophy and thus showed indications for a *feeling of being sovereign*. He states that shopping or dressing choices are easy to make. In addition, he finds it easy to articulate himself as intended. These perceptions were contrasted with a *feeling of loss of control*. During his youth, he became withdrawn socially and as a result a paranoia developed [#68]: “And since it was left untreated and unnoticed for a long time, it got pretty bad too.” He also experiences the exposure of his thoughts as a loss of control when they seem to impact him. SF03a describes the feeling that they all at once are pushing on him. As a child he said to his mother [#69]: “The thoughts don’t stop. I can’t stop thinking” which he experienced as an adult as well [#70]: “It is such a rush of thoughts, such a storm. […] I can no longer filter properly. I can no longer properly classify the thoughts in the right meaning. […] When the thoughts flow in on you, then I would say the filter is broken. There is a processor on the computer, a task scheduler, which assigns to the various processes what is more important, what has to be carried out first. This is then broken in the brain.”

SF03a has *feelings of being present* that derive from a feeling of participating in the social and physical environment [#71]: “I’ve already felt myself in the world. There was a being-in-the-world”, but also describes a *feeling of being absent/not present*, which results from an increasing objectification of the environment. SF03a reports that he usually does not perceive lively objects in the environment with which he can interact. Instead, he feels as an observing subject detached from the world [#72]: “That corresponds a bit to the Cartesian image of man, from Descartes, where you perceive yourself as detached from the world. And just watch. And then the world consists only of things and not of tools.” As a result, he describes that he is increasingly withdrawing and lacking initiative.

In the domain of the *feeling of being unlively/devitalized,* besides described above in quote #72, SF03a reports further phenomena addressing anhedonia, lack of initiative and reduced vitality [#73]: “Sometimes I feel an emptiness inside. Before I got to [name of psychiatric department], I wasn’t interested in anything, enjoyed nothing. I just didn’t want to do anything. I just lay in bed and did nothing. […] I was completely disinterested and lacking in drive. […] I even found it difficult to answer the ringing phone next to the bed, which I knew my mother would call to help me. But to pick up the phone myself and then talk, somehow I couldn’t muster the energy for it.”

In the area of *hyperreflexivity,* SF03a describes to be frequently thinking about events that have occurred long ago as described in quote #66: These self-esteem-related insecurities also address his everyday life [#74]: “I always avoid people, worry a lot about being in the way or something. I am very careful” During the increased reflection, he experiences to no longer participating properly in the environment [#75]: “I am always told that I sometimes stroll through the area, lost in thought. That is true. Sometimes I don’t notice when people greet me because I’m so in my thoughts. […] I really like to think when I walk, and then it can happen that I just walk somewhere [without noticing]. But then I know that it was because I was just thinking so intensely and didn’t attach so much importance to the actual walk.” In addition, SF03a already experienced severe hyperreflexivity that resulted from a psychophysiological misfit [#76]: “When I got that extreme last Friday, I was so busy grasping the situation or thinking about it, just about the fact that I’m in my own body after all and that this is a rather strange feeling that I actually hardly did anything until it got a little better. So I was lost in thought in the middle of the night in the bathroom and had this feeling that I wasn’t in the right body.”

SF03a describes *mirror-related phenomena* as a far-reaching questioning of self-coherence [#77]: “I had the feeling for a short time, even before I looked in the mirror, who am I actually? Who is there in my body?” Even looking in the mirror could not restore self-coherence [#78]: “And then, when I looked in the mirror, I felt very strange. When I looked in the mirror, I just felt like an unfamiliar person. [Noticing] the face that I saw, I thought, who is that? I don’t know him.”

#### Body experience.

When asked about his perception of the body, SF03a expresses a feeling of *rigidness/choppiness* [#79]: “How do I experience my movements? Yes, a little awkward at times. That I don’t know how to move properly, like that.”

Besides, SF03a reports a *psychophysical misfit and psychophysical split* that also meets the criteria of *somatic depersonalization/bodily estrangement* as well as the *feeling of lost body ownership* which we already described in the section above [#80]. “I had thought so completely Cartesian, somehow, body and mind are now totally separate and that was a very strange bodily feeling. […] That was very unusual, like having new shoes that your feet have not yet got used to but related to the whole body. […] That felt very unfamiliar, strange, and wrong. […] I was yet in the body, but yet not. Wrongly, that’s how it felt. […] So the spirit was in the body, but it didn’t quite fit. Like when you try, I don’t know, to put Windows on an Apple computer or something.” The look in the mirror was then made as an attempt to restore the lack of self-coherence as described above in quote #78.

#### Unmentioned categories of self-experience.

SF03a did not describe phenomena addressing the following categories of self-experience: feeling of anxiety, distorted first-person perspective, feeling of being controlled, feeling of being lively/vital, hyperautomaticity, mirror-related phenomena, sense of change in relation to chronological age, sense of change in relation to sexual identity and gender, and fragmentation of experience.

#### Unmentioned categories of body experience.

SF03a did not describe phenomena addressing the following categories of body experience: feeling of body agency, morbid objectifization, loss of body agency, pseudo-movements of the body, motor interferences, motor blocking, disautomation, fluidity of movements, feeling of body ownership, dysmorphic-like experiences, dysmorphophobia, coenesthesias, pain-like experiences, motor slowdown, and thought linked to movements.
